# *N*‑Heterocyclic Olefins of Pyrazole and Indazole

**DOI:** 10.1021/acs.orglett.5c00775

**Published:** 2025-05-28

**Authors:** Bolin Zhu, Rouven Woyciechowski, Eike G. Hübner, Felix Lederle, Andreas Schmidt

**Affiliations:** † Institute of Organic Chemistry, 26534 Clausthal University of Technology, Leibnizstraße 6, D-38678 Clausthal-Zellerfeld, Germany; ‡ Fiber Optical Sensor Systems, Fraunhofer Heinrich Hertz Institute (HHI), Am Stollen 19H, D-38640 Goslar, Germany

## Abstract

Deprotonation of 3-methylpyrazolium and 3-methylindazolium salts yielded *N*-heterocyclic olefins (NHOs) in excellent yields, which reacted with isocyanates, halogens, and carbon disulfide. Calculated proton affinities are 261 kcal/mol (indazole NHOs) and 272 kcal/mol (pyrazole NHOs). The calculated p*K*_a_ values are between 14.8 and 25.2, and bond lengths of the exocyclic double bond are slightly shorter than those of imidazole NHOs. As expected, the highest occupied molecular orbitals show significant atomic orbital coefficients at the exocyclic carbon atom.

## Graphical abstract

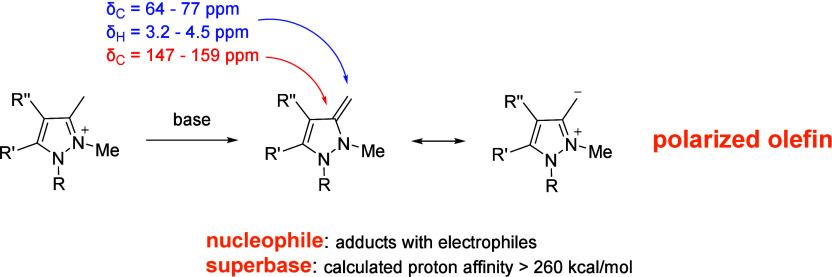

In recent decades, the chemistry of *N*-heterocyclic carbenes (NHCs) has developed impressively,^1^ and there has been a particular focus on their ligand properties for catalysis.^2^ Today, a wide range of different structural types with customized properties are available. *N*-Heterocyclic olefins (NHOs) are formally the methylene adducts of NHCs (Scheme 1). The investigation of their properties and potential applications is currently the focus of interest.

**Scheme 1:**
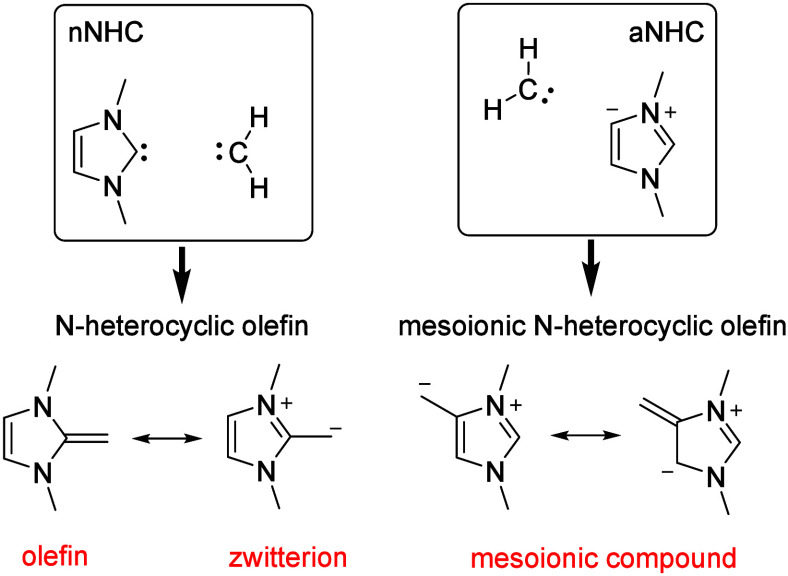
Mesomeric Structures of the NHOs of Imidazole

The formal CH_2_ adducts of the normal NHC (nNHC) imidazol-2-ylidene have an ene-1,1-diamine structural increment that can be described by a zwitterionic mesomeric structure, in which the exocyclic carbon atom carries a negative charge.^3^ The formal adduct of methylene with the abnormal NHC (aNHC) imidazol-4-ylidene, imidazolium-4-methide, is a mesoionic compound^4^ and, therefore, a member of a subclass of mesomeric betaines.^5^ In the course of the development of NHOs, these hetarenium methides are now often referred to as mesoionic *N*-heterocyclic olefins (mNHOs).^6^ The history of NHOs can be traced back several decades. The reaction of ene-1,1-diamine with Zeise’s dimer to form a platinum complex was described in 1979.^7^ The first reports of imidazole-based NHOs date from the 1990s.^8^ NHOs of other ring systems were described (benzimidazoles,^9^ 1,2,3-triazoles,^10^ sydnones,^11^ pyridines,^12,13^ imidazol­[1,5-*a*]­pyridines^14^), and subsequently, adducts with Au­(I),^12^ Rh­(I),^12^ W,^15^ Ir,^16^ Pd,^17^ Pb,^18^ and others followed. Nucleophilicities,^19^ Lewis basicities,^19^ buried volumes,^19^ proton affinities,^20^ Brønsted basicities in DMSO,^10,21^ and their donor strength^22^ were examined. Catalytic reactions of NHOs described include sequestration of CO_2_,^23^ hydroborylation,^24^ hydrosilylation,^25^ transesterifications,^26^ and polymerization of poly­(propylene oxide),^27^ MMA,^28^ and DMMA.^28^ Although the chemistry of NHCs of pyrazole^29^ and indazole^30^ has been investigated, NHOs based on these ring systems are unknown. They are, therefore, the subject of this work.

In this study, indazolium and pyrazolium salts were used as NHO precursors. The indazolium salt **1a** was prepared in good yields by two consecutive methylations with iodomethane^31^ and Meerwein’s reagent,^29^ respectively (Scheme 3). The corresponding 1-phenylindazole derivatives **1b** and **1c** were easily obtained by copper-catalyzed N-phenylations^30,32^ and subsequent methylations, with reaction with iodomethane instead of Meerwein’s reagent yielding better yields in the case of compound **1c**, when nitrobenzene was added as a catalyst and higher temperatures (80 °C) were applied. The 4-methoxyindazole derivative **1d** was synthesized from 2-chloro-6-methoxyacetophenone with methylhydrazine under copper catalysis with subsequent methylation by iodomethane according to modified literature procedures.^33^ All of the indazolium salts were obtained as colorless solids. The deprotonation of the precursor salts **1a**–**1d** in THF using strong bases yielded the desired NHOs **2a**–**2d** in very good yields as yellow–orange to yellow–brownish oils, with potassium hydride proving to be the ideal base for NHO synthesis in all cases, except for the deprotonation of compound **1b** (Scheme 2). The byproducts KI or KBF_4_ could be easily filtered off. Compound **2b** could only be formed without decomposition when LiHMDS was used; however, the byproducts such as suspected LiBF_4_ and hexamethyldisilane could not be completely separated, despite many attempts. The yield therefore refers to the crude product containing LiBF_4_.

**Scheme 2:**
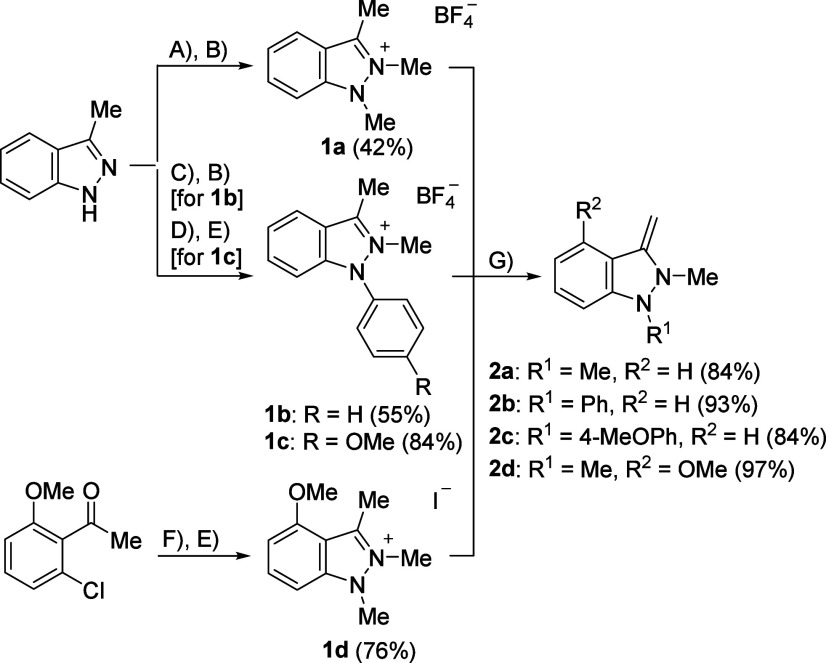
Synthetic Routes of Precursor Indazolium Salts

The yellow-colored pyrazolium salt **3a** was prepared starting from 3-methylpentane-2,4-dione and methylhydrazine under montmorillonite catalysis,^34^ followed by methylation using iodomethane in the presence of catalytic amounts of nitrobenzene (Scheme 3). The reaction of 2-methyl-1-phenylbutane-1,3-dione with phenylhydrazine under copper catalysis^35^ gave a pyrazole, which was subsequently methylated to give compound **3b** as an orange solid. Deprotonation with KH, respectively, gave pyrazole NHOs **4a** and **4b**. All indazole and pyrazole NHOs described here are yellow to orange oils or solids. With the exception of compound **4a**, which decomposed immediately during the drying process, the NHOs are stable in the absence of moisture and can be stored under an inert atmosphere at −20 °C for several days without significant changes in the NMR spectra. Whereas the indazole NHOs **2a**–**2d** are not soluble in nonpolar solvents, the pyrazole NHOs are highly soluble and could be easily extracted with pentane or toluene from the crude reaction mixture.

**Scheme 3:**
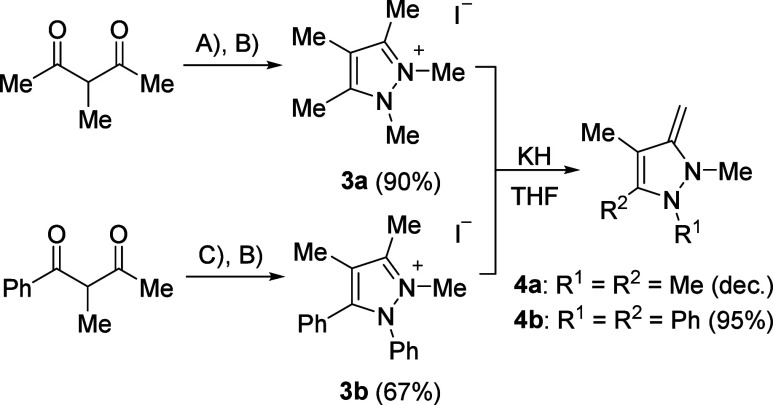
Synthetic Routes of Precursor Pyrazolium Salts

The polarization of the olefinic double bond is evident from the chemical shifts in the NMR spectra. The exocyclic carbons of the pyrazole and indazole NHOs appear between 64.1 and 76.2 ppm, and the corresponding ^1^H NMR spectra shift between 3.2 and 4.0 ppm. Table 1 shows the comparison of chemical shifts between compounds **5**, **6**, **7**, and **8** (Figure 1) and the herein described pyrazole and indazole NHOs.

**Table 1:** Relevant Chemical Shifts of the Double Bonds of NHOs

NHO	solvent	^1^H δ	^13^C δ (C_endo_)	^13^C δ (C_exo_)
**5** ^8^	C_6_D_6_	2.77	153.6	40.2
**6** ^9^	C_6_D_6_	3.12	152.7	47.0
**7** ^12^	C_6_D_6_	3.34, 3.60		70.4
**8** ^10^	tol-*d*_8_	2.69, 3.47		49.7
**2a**	DMSO-*d*_6_	3.78, 4.23	151.5	71.8
**2b**	DMSO-*d*_6_	3.99, 4.42	150.9	73.2
**2c**	DMSO-*d*_6_	3.92, 4.36	157.7	72.5
**2d**	DMSO-*d*_6_	3.86, 4.51	155.6	76.2
**4a**	THF-*d*_8_	3.19	158.8	64.1
**4b**	DMSO-*d*_6_	3.49, 3.51	157.5	67.0
	C_6_D_6_	3.94, 3.85	158.6	67.5
	THF-*d*_8_	3.52, 3.48	158.8	66.7
	tol-*d*_8_	3.85, 3.76	158.5	67.5
**11a**	DMSO-*d*_6_		147.8	73.5
**11b**	DMSO-*d*_6_		147.1	73.4
**11c**	DMSO-*d*_6_		148.0	73.5
**11d**	DMSO-*d*_6_		148.1	73.5
**11e**	DMSO-*d*_6_		151.1	72.5

**Figure 1:**
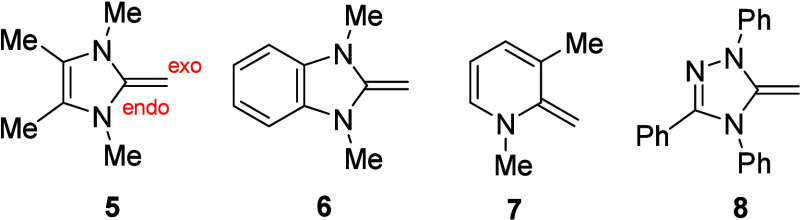
Reference NHOs from the literature.

The endo carbons of all listed NHOs are detectable in the ^13^C NMR spectra between 150.9 and 158.8 ppm. When the chemical shifts of the terminal olefinic carbons are taken as a reference, a rough comparison of the degree of polarization between the different ring systems can be made, yielding the following ranking: imidazole > benzimidazole > triazole > pyrazole > pyridine > indazole. A significant solvent dependence of the ^1^H NMR resonance frequencies was demonstrated using NHO **4b** as an example. Replacing the solvent DMSO-*d*_6_ with toluene-*d*_8_, for example, resulted in a shift of the ^1^H NMR resonance frequencies by up to Δδ = 0.45 ppm. It is also evident that, in both the pyrazole/indazole and imidazole/benzimidazole systems, phenyl substituents and fused benzene rings lead to a deshielding of the exocyclic CH_2_ protons, while the carbon atoms are minimally shielded. The indazole NHOs react with elemental iodine or bromine analogous to imidazole NHOs^8^ to give the corresponding iodo- and bromomethyl indazoles and pyrazoles, respectively (Scheme 4 and Table 2). Thus, methylated indazole NHO **2a** reacts with elemental iodine and bromine to give adducts **9a** and **9b** as pure and stable yellow solids. However, the decomposable phenyl derivative **9b** could not be separated from the reaction mixture as it already decomposed during filtration and formed a dark oil. The pyrazole NHOs **4a** and **4b** show different reactivities compared to the indazole NHOs. Only the bromination product **4b** was obtained, which yielded compound **9c** as a yellow solid. Carbon disulfide reacted with compounds **2a**, **4a**, and **4b** in THF to give red–orange adducts **10a**–**10c** that spontaneously precipitated from the reaction solution in sufficient yields. The phenyl derivative does not react under the same conditions.

**Scheme 4:**
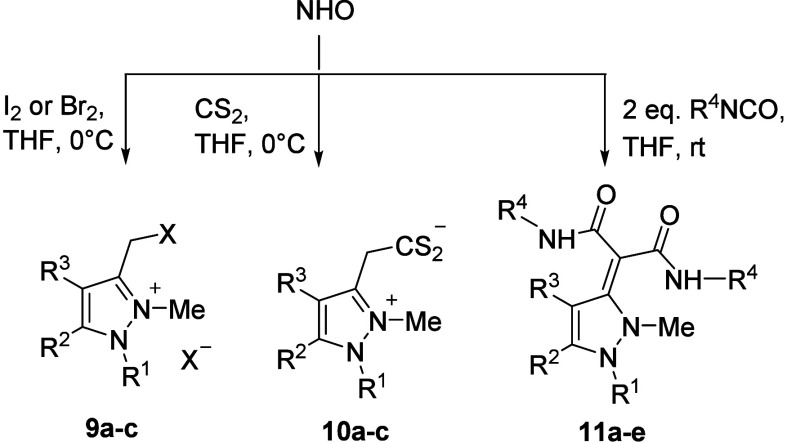
Reaction of NHOs with Electrophiles

**Table 2:** Substitution Patterns and Yields of the Adducts

reaction	R^1^	R^2^	R^3^	R^4^	X	yield (%)
**2a** → **9a**	Me	–CH=CH–CH=CH–			I	40
**2b** → **9b**	Me	–CH=CH–CH=CH–			Br	70
**4b** → **9c**	Ph	Ph	Me		Br	92
**2a** → **10a**	Me	–CH=CH–CH=CH–				77
**4a** → **10b**	Me	Me	Me			50
**4b** → **10c**	Ph	Ph	Me			50
**2a** → **11a**	Me	–CH=CH–CH=CH–		Ph		41
**2a** → **11b**	Me	–CH=CH–CH=CH–		4-Cl-C_6_H_4_		46
**2a** → **11c**	Me	–CH=CH–CH=CH–		2-MeO-C_6_H_4_		82
**2a** → **11d**	Me	–CH=CH–CH=CH–		4-Me-C_6_H_4_		75
**4a** → **11e**	Ph	Ph	Me	4-Cl-C_6_H_4_		48

The reaction of the indazole NHOs with various aryl isocyanates in THF gave the products **11a**–**11d** as yellow to orange precipitates in pentane. However, the adduct of compound **2b** with phenyl isocyanate could not be purified due to rapid decomposition. No product was obtained when alkyl isocyanates were employed. Pyrazol NHOs reacted with aryl isocyanates and yielded yellow adducts with poor stability. The only characterizable product proved to be compound **11e**, although the exocyclic double bond is part of the β-enaminocarbonyl chromophore, which is known to be a stabilizing push–pull group.^36^ All of these products with isocyanates decompose at their melting point, indicating less stabilities than comparable adducts of other ring systems. The ^13^C NMR signals of the double bonds, summarized in Table 1, appear at around 73 and 148 ppm, indicating highly polarized bonds. However, the adducts **11a**–**11e** apparently have a lower degree of polarization than the pyrazole, indazole, and imidazoline NHOs, whose ^13^C NMR resonance frequencies appear between 71 and 166 ppm. Table 3 shows the calculated^37^ [6-311++G­(2df,2p)/M06-2X//6-31G­(d)/PBE0-D3] proton affinities of the pyrazole and indazole NHOs, which range from 260.7 kcal/mol (**2a**) to 273.4 kcal/mol (**4b**). For the sake of comparability, the values of imidazole NHO **5** and benzimidazole NHO **6** were also calculated. Their values are in the same range. Proton affinities of imidazole and triazole NHO superbases were also calculated earlier to range from 262 to 296 kcal/mol.^20^ Table 3 also shows p*K*_a_ values calculated in DMSO via the indirect method [PCM/6-311++G­(2df,2p)/M06-2X//6-31G­(d)/PBE0-D3] and referenced to the value of 17.2 for compound **6**. The p*K*_a_ values are between 17.6 (**2a**) and 25.2 (**4a**), and for the compounds based on imidazole and benzimidazole, they are between 17.2 and 25.7. The literature values [SMD/6-311++G­(2df,2p)/M06-2X//6-31+G­(d)/B3LYP-D3] are also given for comparison. Calculated p*K*_a_ values of imidazole, triazole, and thiazole NHOs in DMSO are available in the literature.^21^ Table 3 also presents calculated bond lengths *in vacuo* [6-31G­(d)/PBE0-D3]. In line with the formulation of mesomeric structures, they are slightly longer than, for example, the C_sp^2^_–C_sp^2^_ bond length of ethene (132 pm), but they are far from reaching the values of a C_sp^2^_–C_sp^2^_ or C_sp^3^_–C_sp^2^_ single bond, like that in butadiene (148 pm) or toluene (151 pm). In comparison to the imidazole and benzimidazole NHOs, the indazole and pyrazole NHOs have slightly shorter calculated exocyclic C=C bond lengths, indicating less polarization than in the imidazole and benzimidazole NHOs. This is also reflected in the NMR values, as mentioned before. Notably, annulated phenyl rings attached to both the imidazole and pyrazole backbone reduce the polarization of the exocyclic double bond.

**Table 3:** Calculated Proton Affinities (PAs), p*K*_a_ Values, and Bond Lengths of the NHOs **2a**, **2b**, **4a**, and **4b** as Well as Reference NHOs

NHO	PA	PA^20^	p*K*_a_	p*K*_a_ ^21^	bond length (pm)	orbital contribution of C_exo_ to the HOMO (%)
**2a**	260.7		17.6		134.5	34
**2b**	262.1		14.8		134.4	31
**4a**	271.0		25.2		134.8	40
**4b**	273.4		21.8		134.9	41
**5**	273.2	273.9	25.7	24.5	135.7	42
**6**	260.6	262.4	17.2*	17.2	134.9	39

The highest occupied and lowest unoccupied molecular orbitals [6-31G­(d)/PBE0-D3] of the NHOs **2a** and **4a** are shown in Figure 2, and those of the hypothetical examples **12a** and **12b** are presented in the Supporting Information. Compounds **12a** and **12b** are the methylisocyanate adducts of the methyl-substituted pyrazole and indazole NHOs, respectively. The highest occupied molecular orbitals (HOMOs) of compounds **2a** and **4a** both show pronounced atomic orbital coefficients at the exocyclic olefinic carbon, around 10 percentage points higher for the pyrazole NHOs.

**Figure 2:**
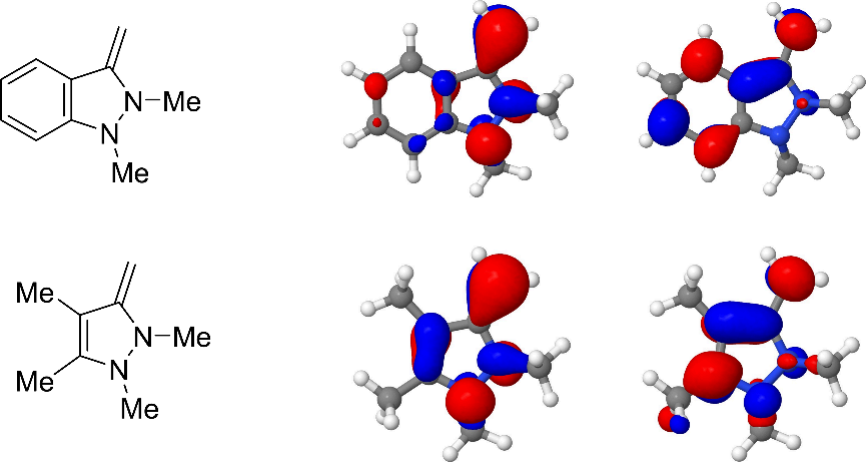
HOMO/LUMO profile of compounds **2a** (above) and **4a** (below).

Their energies are summarized in Table S4 of the Supporting Information and shown graphically in Figure 3 as a comparison with imidazole and benzimidiazole NHOs **5** and **6**. It is evident that indazole and pyrazole NHOs have lower HOMO energies and smaller HOMO/LUMO gaps than imidazole NHOs, indicating less nucleophilicity and reactivities that correspond to the experimental results. Among the calculated pyrazole and indazole NHOs, the pyrazole NHO **4a** has the highest HOMO/LUMO gap. The extended π systems of the hypothetical methylisocyanate adducts **12a** and **12b** cause a further reduction in the frontier orbital energies.

**Figure 3:**
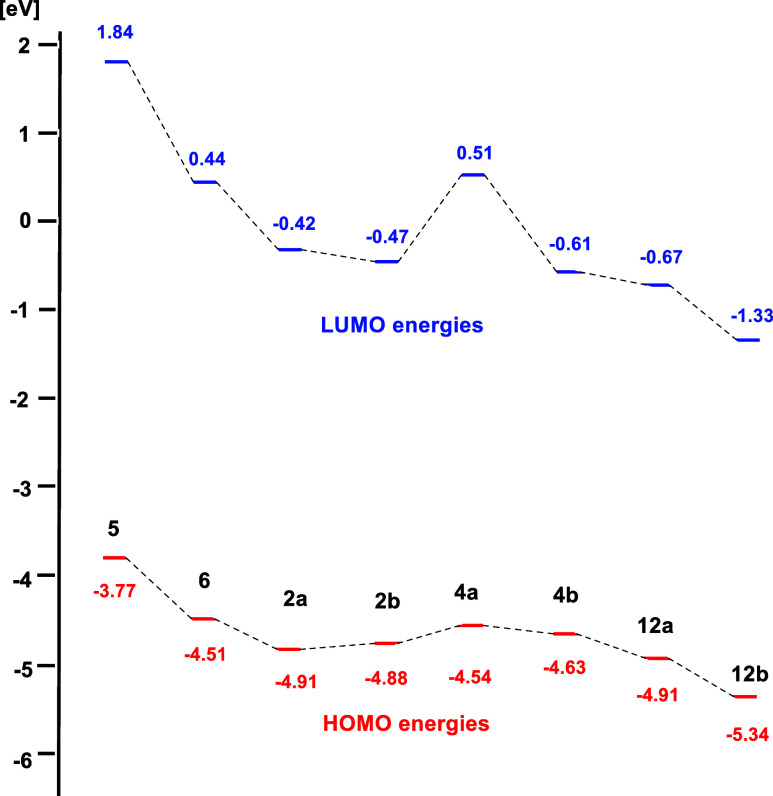
Comparison of frontier orbital energies.

In summary, a total of six NHOs were synthesized, including four indazole NHOs and two pyrazole NHOs. These have an exocyclic double bond whose polarity is translated into nucleophilic properties against electrophiles, such as halogens, isocyanates, and carbon disulfide, as well as considerable calculated basicities, proton affinities, and characteristic NMR shifts. Among these NHOs and their reaction products with electrophiles, *N*,*N*′-dimethylindazole NHO **2a** and 2,4-dimethyl-1,5-diphenylpyrazole NHO **4b** exhibit optimal stability and reactivity. In comparison to the imidazole and benzimidazole NHOs **5** and **6**, the calculated bond lengths are slightly shorter and the frontier orbital energies, with the exception of pyrazole NHO **4a**, are on average smaller. Overall, the pyrazole and indazole NHOs and their properties are in line with those of the previously known NHOs of other ring systems but show characteristic differences to those, which encourage further work.

## Supplementary Material



## Data Availability

The data underlying this study are available in the published article and its Supporting Information.

## Notes

Experimental procedures, characterization data, copies of ^1^H and ^13^C NMR spectra, HOMO–LUMO profiles, and details of calculations (PDF)
