# Supplementary material for: N‑Heterocyclic Olefins of Pyrazole and Indazole
Source: Org Lett. 2025 May 28;27(22):5572–7. doi: 10.1021/acs.orglett.5c00775 (PMC12150313; doi:10.1021/acs.orglett.5c00775)
Supplement: Supplementary file 1 [file ol5c00775_si_001.pdf]

N-Heterocyclic olefins of pyrazole and indazole

Bolin Zhu,[a] Rouven Woyciechowski,[a] Eike G. Hübner,[a,b] Felix Lederle,[a,b] and Andreas Schmidt[a]*

[a] Clausthal University of Technology, Institute of Organic Chemistry, Leibnizstraße 6, 38678 Clausthal-Zellerfeld, Germany. E-mail: schmidt@ioc.tu-clausthal.de

[b] Fraunhofer Heinrich Hertz Institute, HHI, Fiber Optical Sensor Systems, Am Stollen 19 H, D-38640 Goslar, Germany.

Contents

Experimental part S6

General procedures of *N*-quaternization reaction S6

Synthesis of 1,3-dimethyl-1*H*-indazole S7

Synthesis of 1,2,3-trimethyl-1*H*-indazolium tetrafluoroborate **1a** S9

Synthesis of 3-methyl-1-phenyl-1H-indazole S14

Synthesis of 2,3-dimethyl-1-phenyl-1*H*-indazolium tetrafluoro-borate **1b** S16

Synthesis of 1-(4-methoxylphenyl)-3-methyl-1*H*-indazole S21

Synthesis of 2,3-dimethyl-1-(4-methoxylphenyl)-1*H*-indazolium iodide **1c** S23

Synthesis of 1,3-dimethyl-4-methoxyl-1*H*-indazole S28

Synthesis of 1,2,3-trimethyl-4-methoxyl-1*H*-indazolium iodide **1d** S33

Synthesis of 1,3,4,5-tetramethyl-1*H*-pyrazole S38

Synthesis of 1,2,3,4,5-pentamethyl-1*H*-pyrazolium iodide **3a** S40

Synthesis of 3,4-dimethyl-1,5-diphenyl-1*H*-pyrazole S41

Synthesis of 1,5-Diphenyl-2,3,4-trimethyl-1*H*-pyrazolium iodide **3b** S43

General procedure of NHO synthesis S50

Synthesis of 2,3-dihydro-1,2-dimethyl-3-methylene-1*H*-indazole **2a** S50

Synthesis of 2,3-dihydro-2-methyl-3-methylene-1-phenyl-1*H*-indazole **2b** S55

Synthesis of 1-(4-methoxyphenyl)-2-methyl-3-methylene-2,3-dihydro-1*H*-indazole **2c** S60

Synthesis of 2,3-dihydro-1,2-dimethyl-3-methylene-4-methoxyl-1*H*-indazole **2d** S65

Synthesis of 2,4-dimethyl-3-methylene-1,5-diphenyl-2,3-dihydro-1*H*-pyrazole **4a** S70

Synthesis of 2,4-dimethyl-3-methylene-1,5-diphenyl-2,3-dihydro-1*H*-pyrazole **4b** S74

General procedure of NHO adduct synthesis S80

Synthesis of 3-iodomethyl-1,2-dimethyl-1*H*-indazol-2-ium iodide **9a** S80

Synthesis of 3-bromomethyl-1,2-dimethyl-1*H*-indazol-2-ium bromide **9b** S85

Synthesis of 3-bromomethyl-1,2-dimethyl-1*H*-indazol-2-ium bromide **9c**............ S90

Synthesis of 2-(1,2-dimethyl-1*H*-indazol-2-ium-3-yl)ethanedithioate **10a**............ S94

Synthesis of 2-(1,2,4,5-tetramethyl-1*H*-pyrazol-2-ium-3-yl)ethanedithioate **10b**. S99

Synthesis of 2-(1,2,4,5-tetramethyl-1*H*-pyrazol-2-ium-3-yl)ethanedithioate **10c**S103

Synthesis of N^1,N^3-bichlorophenyl-2-(1,2-dimethyl-2,3-dihydro-1*H*-indazol-3-ylidene)malonamide **11a**. S107

Synthesis of N^1,N^3-bis(4-chlorophenyl)-2-(1,2-dimethyl-2,3-dihydro-1*H*-indazol-3-ylidene)malonamide **11b** S112

Synthesis of N^1,N^3-bis(2-methoxylphenyl)-2-(1,2-dimethyl-2,3-dihydro-1*H*-indazol-3-ylidene)malonamide **11c** S117

Synthesis of N^1,N^3-bis(4-methylphenyl)-2-(1,2-dimethyl-2,3-dihydro-1*H*-indazol-3-ylidene)malonamide **11d** S121

Synthesis of N^1,N^3-bis(4-chlorophenyl)-2-(2,4-dimethyl-1,5-diphenyl-1,2-dihydro-3*H*-pyrazol-3-ylidene)malonamide **11e**.. S126

.... S128

DFT calculated structures S131

1,2-Dimethyl-3-methylene-2,3-dihydro-1*H*-indazole **2a** S131

vacuum S131

DMSO S133

1,2,3-Trimethyl-1H-indazol-2-ium **1a** S134

vacuum S134

DMSO S135

2-Methyl-3-methylene-1-phenyl-2,3-dihydro-1*H*-indazole **2b** S136

vacuum S136

DMSO S137

2,3-Dimethyl-1-phenyl-1H-indazol-2-ium**1b** S139

vacuum S139

vacuum S142

DMSO S143

1,2,3,4,5-Pentamethyl-1*H*-pyrazol-2-ium **3a** S144

vacuum S144

DMSO S145

2,4-Dimethyl-3-methylene-1,5-diphenyl-2,3-dihydro-1*H*-pyrazole **4b** S146

vacuum S146

DMSO S148

2,3,4-Trimethyl-1,5-diphenyl-1H-pyrazol-2-ium **3b** S150

vacuum S150

DMSO S152

1,3,4,5-Tetramethyl-2-methylene-2,3-dihydro-1H-imidazole **5** S154

vacuum S154

DMSO S155

1,2,3,4,5-Pentamethyl-1H-imidazol-3-ium **5 cation** S156

vacuum S156

DMSO S157

1,3-Dimethyl-2-methylene-2,3-dihydro-1H-benzo[*d*]imidazole **6** S158

vacuum S158

DMSO S159

1,2,3-Trimethyl-1H-benzo[d]imidazol-3-ium **6 cation** S160

vacuum S160

DMSO S161

1,3-Dimethyl-2-methylene-1,2-dihydropyridine **7** S162

vacuum S162

DMSO S163

1,2,3-Trimethylpyridin-1-ium **7 cation** S164

vacuum S164

DMSO S165

2-(1,2-Dimethyl-1,2-dihydro-3*H*-indazol-3-ylidene)-N^1,N^3-dimethylmalonamide **12b** S166

vacuum S166

DMSO S167

3-(1,3-Bis(methylamino)-1,3-dioxopropan-2-yl)-1,2-dimethyl-1*H*-indazol-2-ium **12b cation** S168

vacuum S168

DMSO S170

N^1,N^3-Dimethyl-2-(1,2,4,5-tetramethyl-1,2-dihydro-3*H*-pyrazol-3-ylidene)malonamide **12a** S171

vacuum S171

DMSO S174

3-(1,3-Bis(methylamino)-1,3-dioxopropan-2-yl)-1,2,4,5-tetramethyl-1*H*-pyrazol-2-ium **12a cation** S176

vacuum S176

DMSO S179

Experimental part

General procedures of *N*-quaternization reaction

Procedure A: The quaternization is carried out using Meerwein´s reagent. In an oven-dried Schlenk flask was added 1-substituted 1*H*-indazole or 1*H*-pyrazole (1 Eq) under nitrogen atmosphere in anhydrous DCM. Meerwein's reagent trimethyloxonium tetrafluoroborate (1.2 Eq) was added to and the mixture was stirred at room temperature for 24 h. The solvent was evaporated in vacuum and the residue was washed with diethyl ether. The crude product was added to 10 mL of water and the mixture was heated to boiling with a heat gun. A small amount of water was added and heated to boiling until all solids were dissolved. The saturated solution was allowed to cool to room temperature and the precipitated solid was filtered off, collected and dried in vacuum to afford the final product.

Procedure B: The quaternization is carried out using iodomethane. In a sealed tube was added 1-substituted 1*H*-indazole or 1*H*-pyrazole (1.0 Eq), acetonitrile and iodomethane (2.0 Eq). To the mixture was added 0.05 mL nitrobenzene as catalyst. The sealed tube was closed and stirred magnetically at 80 °C in an oil bath for 24 h. The solvent was evaporated in vacuum and the residue was washed three times with ethyl acetate to afford the product.

Synthesis of 1,3-dimethyl-1*H*-indazole

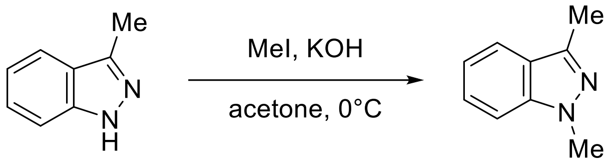

The methylation of 1*H*-indazole is prepared according to the literature: Ben-Yahia, A.; Naas, M.; El Kazzouli, S.; Essassi, E.M.; Guillaumet, G., Direct C-3-arylations of 1*H*-indazoles. *Eur. J. Org. Chem.* **2012**, 2012, 7075–7081. DOI: 10.1002/ejoc.201200860.

1*H*-Indazole (1.0 Eq, 500 mg, 3.78 mmol) was dissolved in 20 mL acetone at 0 °C. KOH (3.0 Eq, 637 mg, 11.3 mmol) was added to the mixture, followed by dropwise addition of iodomethane (1.5 Eq, 805 mg, 5.67 mmol). The reaction mixture was filtered and the solvent evaporated in vacuo. The product was further purified by flash chromatography on silica gel and afforded as light-yellow oil (385 mg, 69%).

^{1}H-NMR ($CDCl_3$, 400 MHz): δ = 7.65 (dt, J = 8.0, 1.0 Hz, 1H), 7.38 (td, J = 8.0, 1.0 Hz, 1H), 7.32 (dt, J = 8.0, 1.0 Hz, 1H), 7.12 (td, J = 8.0, 1.0 Hz, 1H), 4.01 (s, 3H), 2.58 (s, 3H) ppm.

The NMR spectrum corresponds to the literature: Viña, D.; del Olmo, E.; López-Pérez, J. L.; Feliciano, A. S., One-Pot Cu-Catalyzed Synthesis of 3-Substituted Indazoles, *Org. Lett.* **2007**, 9 (3), 525-528. DOI: 10.1055/s-2007-968580.

^{1}H-NMR

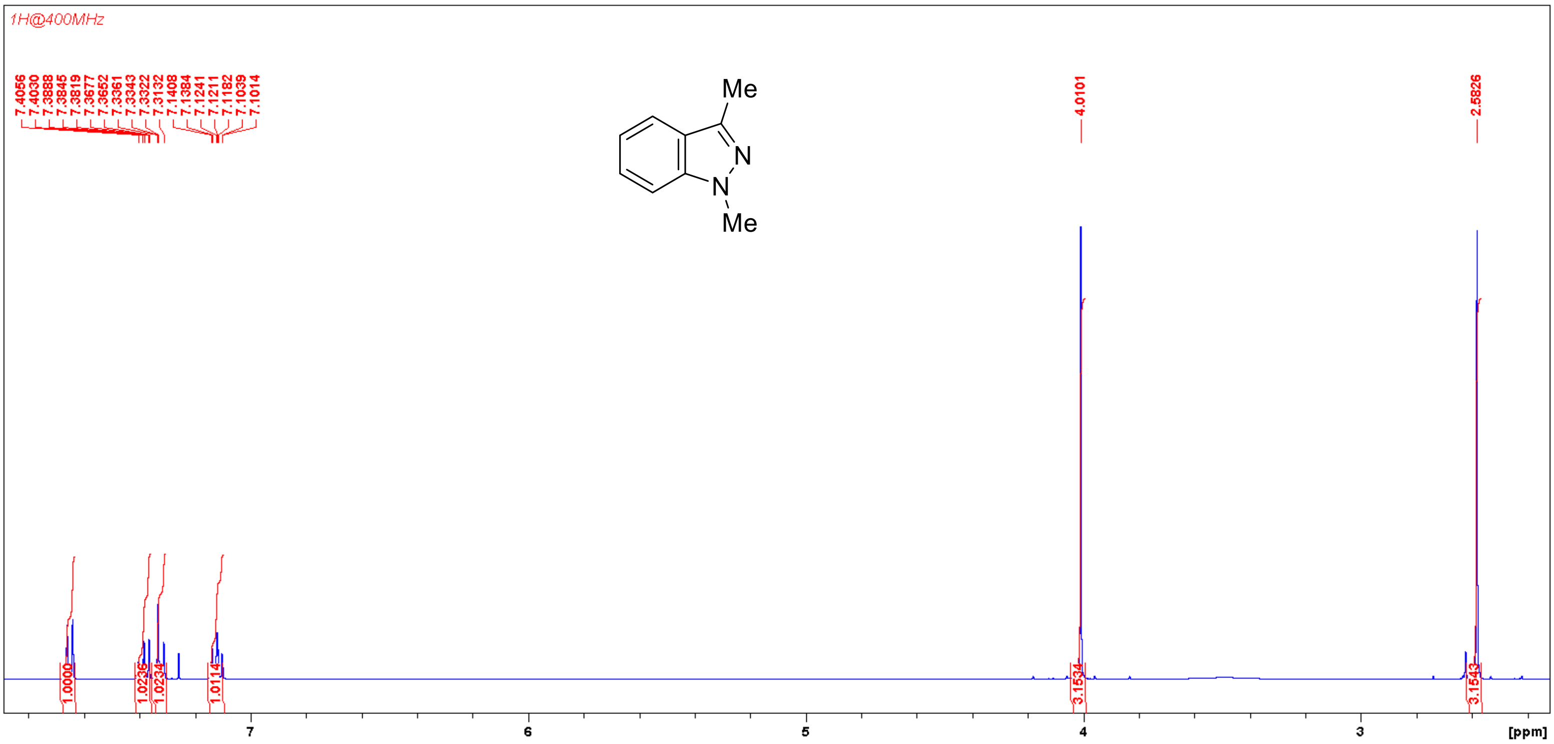

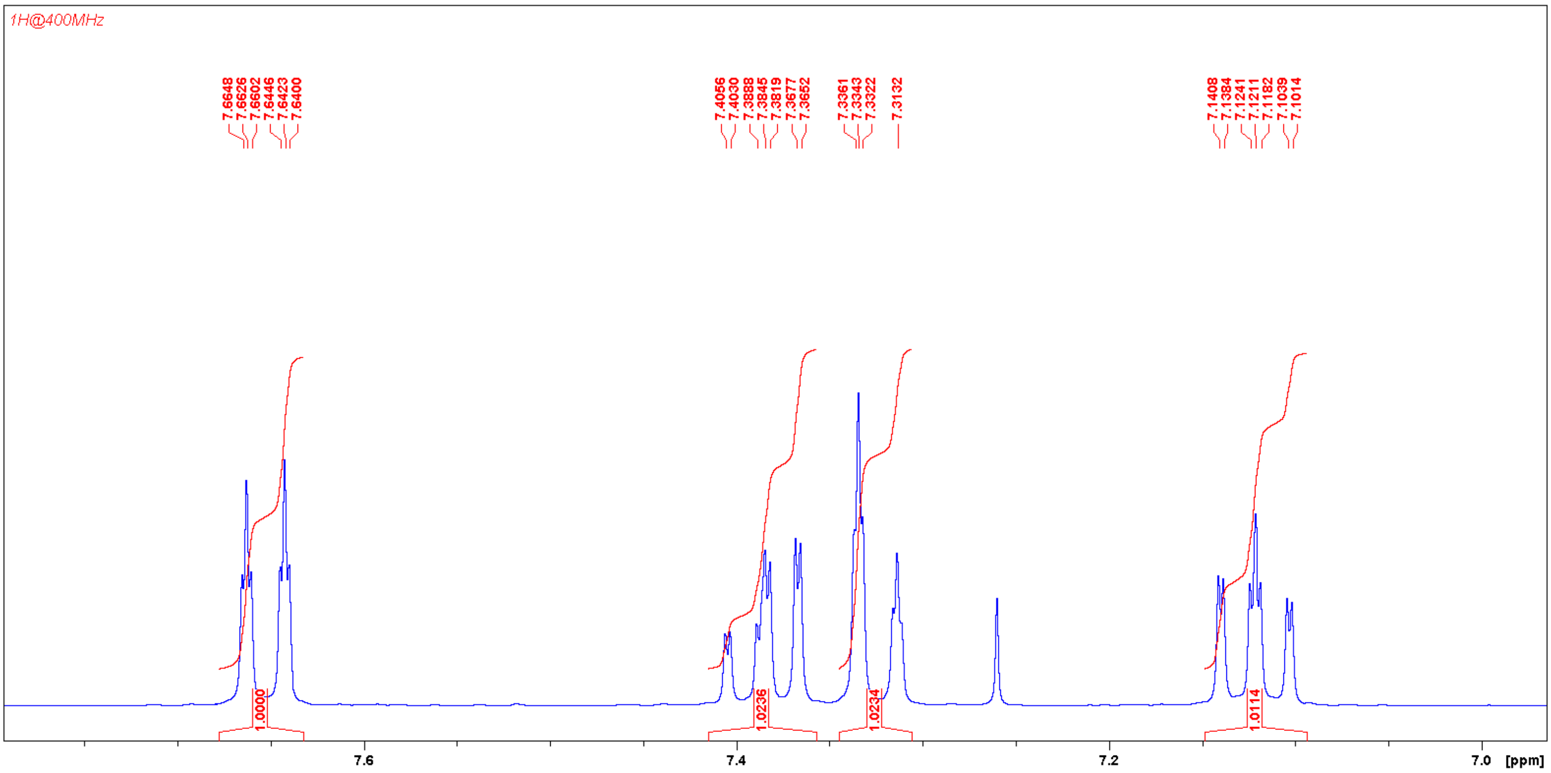

Synthesis of 1,2,3-trimethyl-1*H*-indazolium tetrafluoroborate **1a**

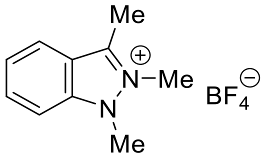

Followed by quaternization procedure A, **1a** was synthesized from 1,3-dimethyl-1*H*-indazole (1.993 g, 13.63 mmol) in 50 mL anhydrous DCM as white solid (1.411 g, 42%).

^{1}H-NMR (DMSO-d$_6$, 600 MHz): δ = 8.14 (dt, J = 8.0, 1.0 Hz, 1H, Ar-H), 7.95 (dt, J = 8.0, 1.0 Hz, 1H, Ar-H), 7.86 (td, J = 8.0, 1.0 Hz, 1H, Ar-H) 7.48 (td, J = 8.0, 1.0 Hz, 1H, Ar-H), 4.23 (s, 3H, N-Me), 4.22 (s, 3H, N-Me), 2.85 (s, 3H, Me) ppm.

^{13}C{^{1}H}-NMR (DMSO-d$_6$, 150 MHz): δ = 142.1 (o, Ar-C), 139.1 (o, Ar-C), 133.0 (+, Ar-C), 123.9 (+, Ar-C), 122.2 (+, Ar-C), 118.9 (o, Ar-C), 111.8 (+, Ar-C), 34.4 (+, N-Me), 33.2 (+, N-Me), 10.3 (+, Me) ppm.

ESI-MS (m/z): calculated for $[C_{10}H_{13}N_2]^+$: 161.1073, found 161.1073.

IR (ATR): $\tilde{\nu}$ = 1023 (indazole ring vibration), 775 (BF_4^-) cm^{-1}.

Melting point: 192-195 °C.

^{1}H-NMR

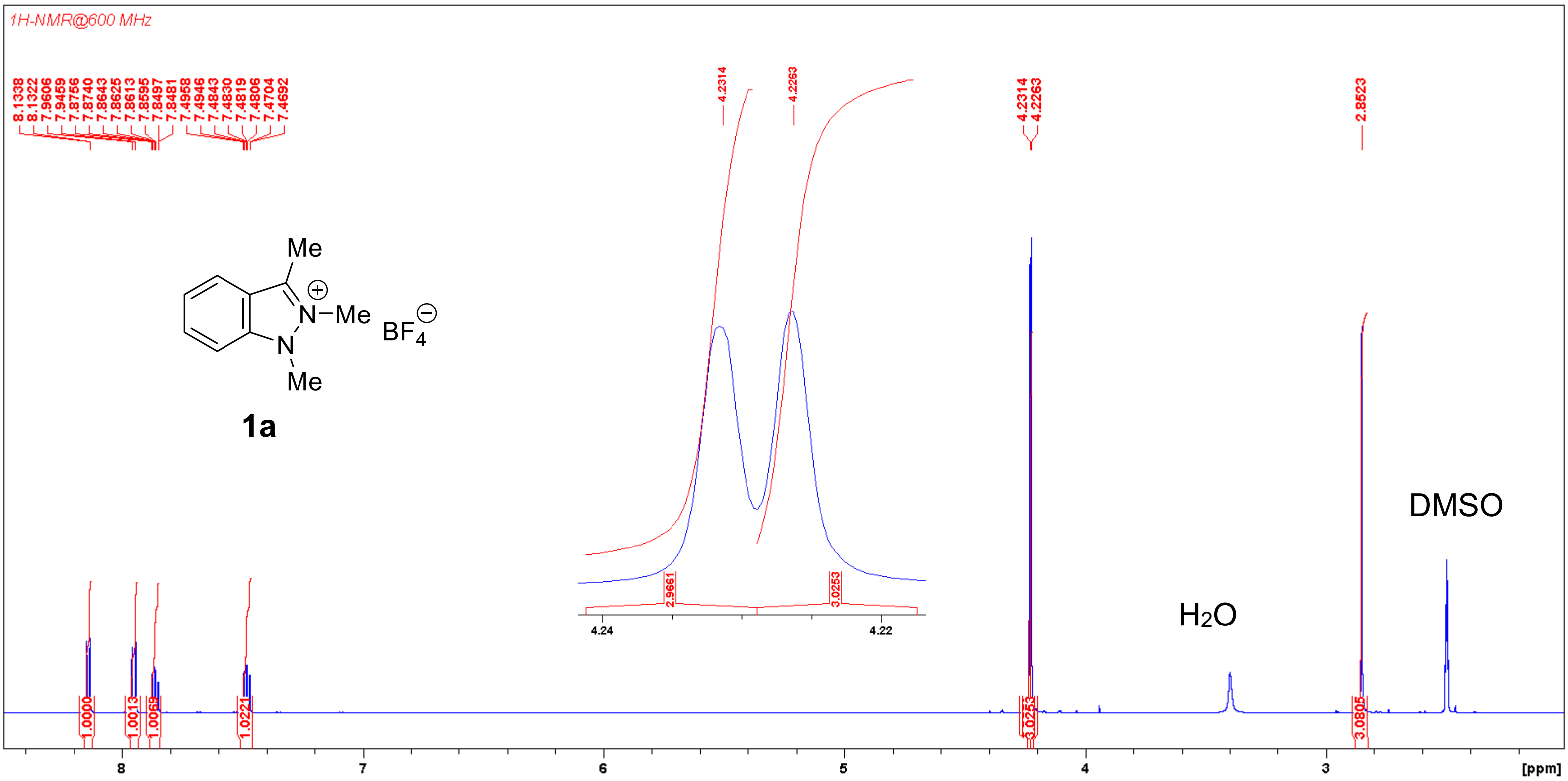
1H-NMR@600 MHz
8.1338 8.1322 7.9606 7.9459 7.8756 7.8740 7.8643 7.8625 7.8613 7.8595 7.8497 7.8481 7.4958 7.4946 7.4843 7.4830 7.4819 7.4806 7.4704 7.4692
4.2314 4.2263
2.8523
Me
N−Me
BF4
N
Me
1a
2.9661
3.0253
4.24
4.22
H2O
DMSO
1.0000
1.0013
1.0069
1.0221
3.0253
3.0805
8
7
6
5
4
3
[ppm]

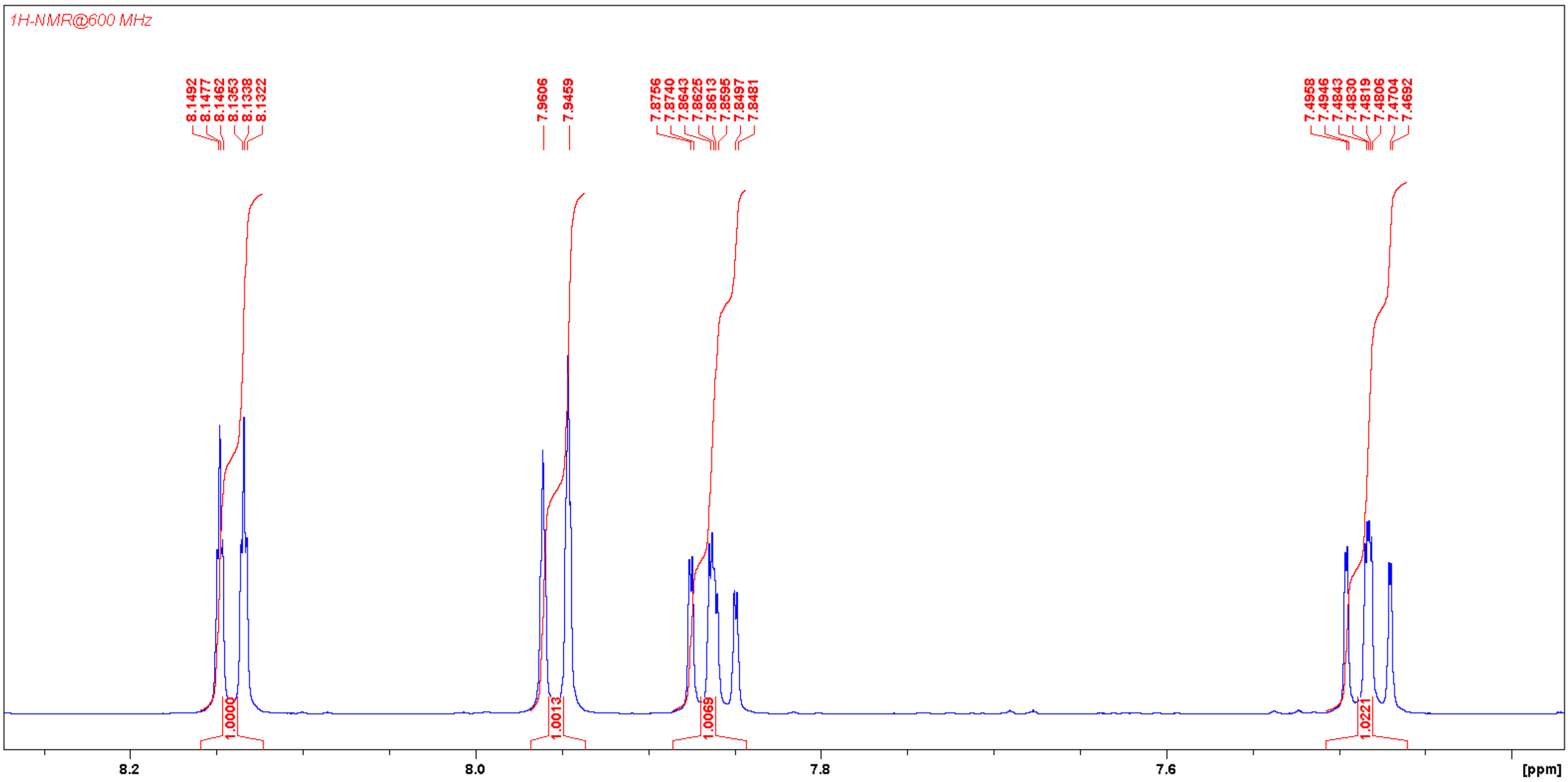
1H-NMR@600 MHz
8.1492 8.1477 8.1462 8.1353 8.1338 8.1322
7.9606 7.9459
7.8756 7.8740 7.8643 7.8625 7.8613 7.8595 7.8497 7.8481
7.4958 7.4946 7.4843 7.4830 7.4819 7.4806 7.4704 7.4692
1.0000
1.0013
1.0069
1.0221
8.2
8.0
7.8
7.6
[ppm]

$^{13}C\{^1H\}$-NMR

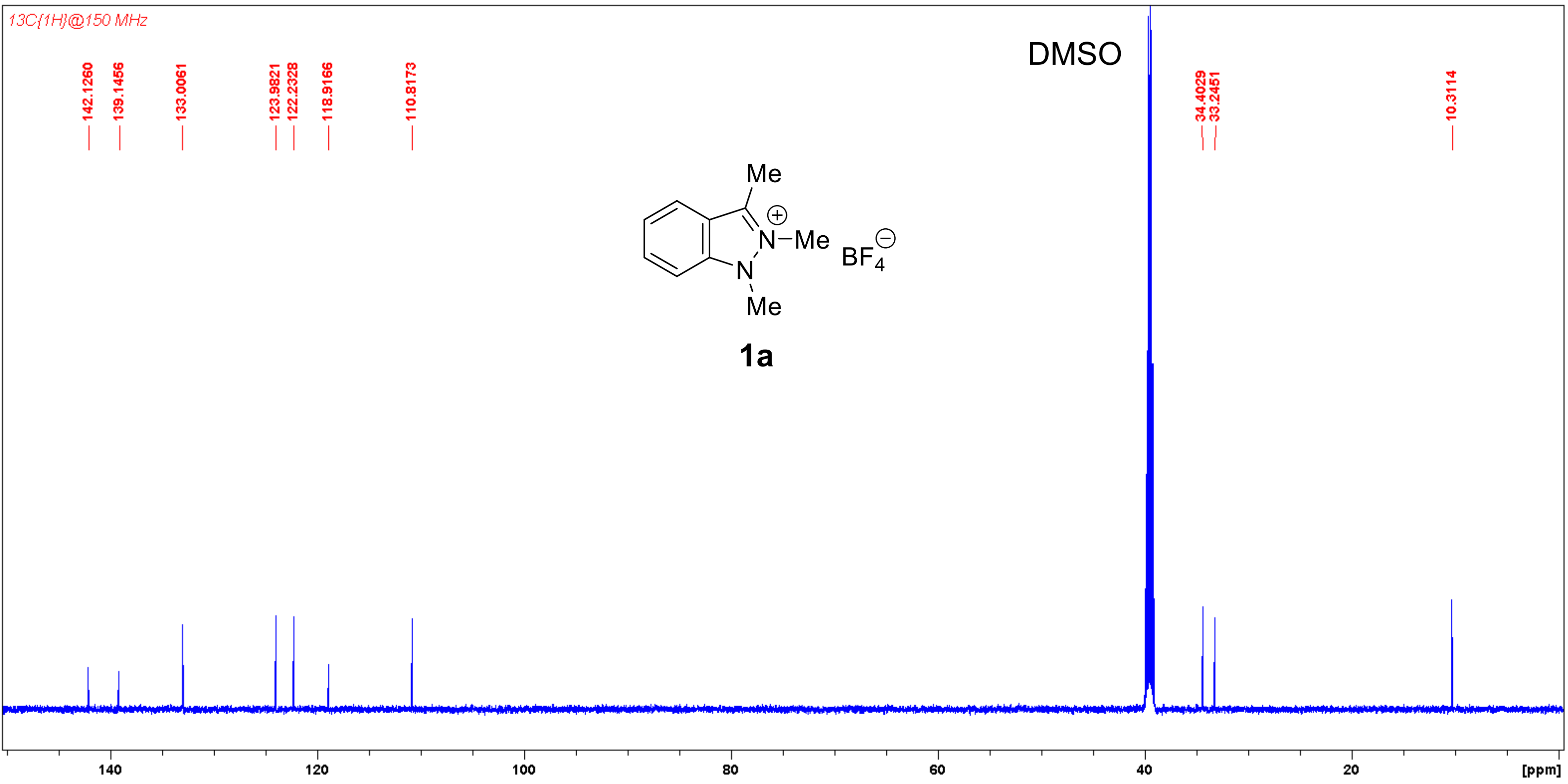

^{13}C-DEPT

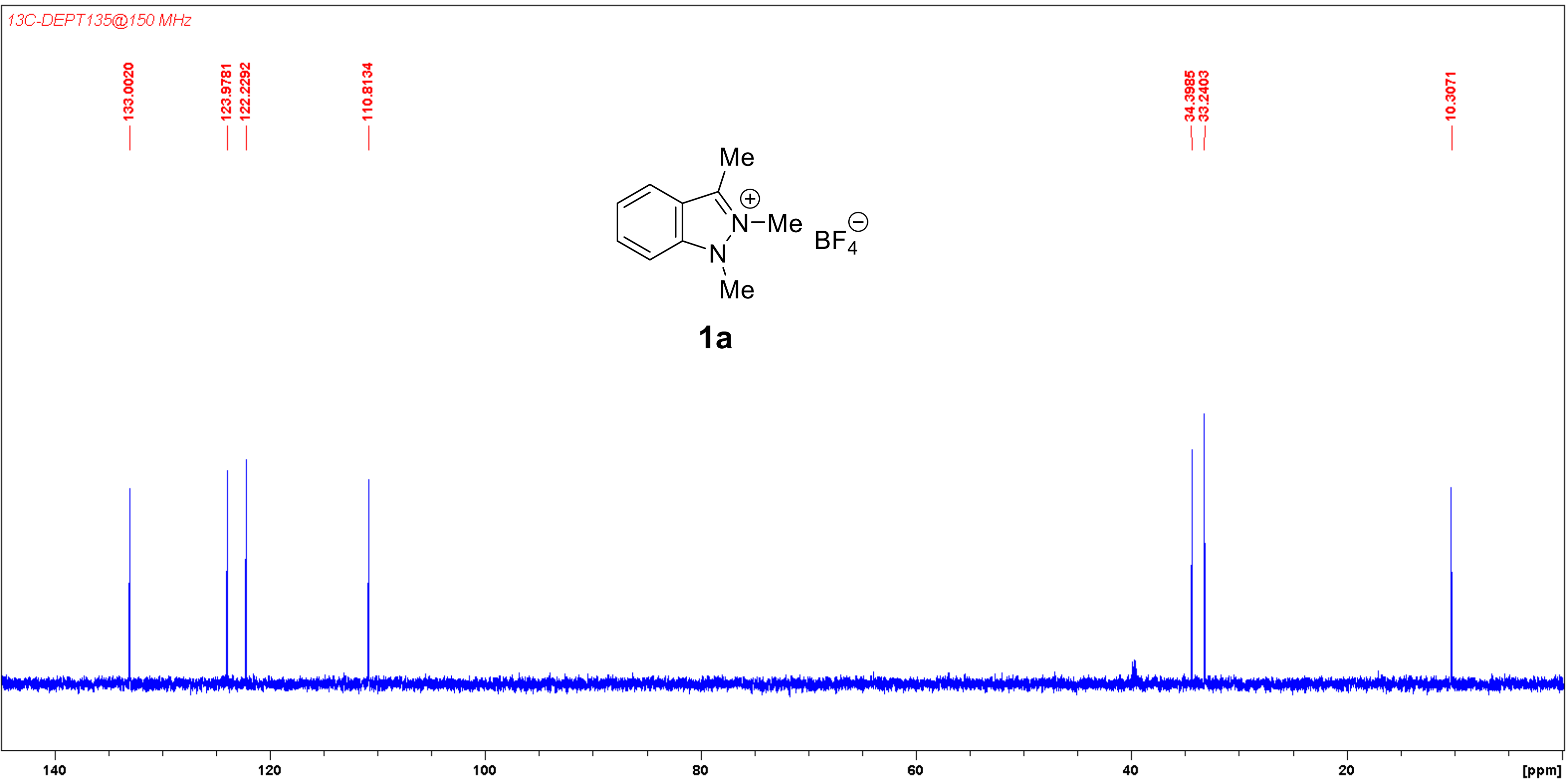

H,H-COSY

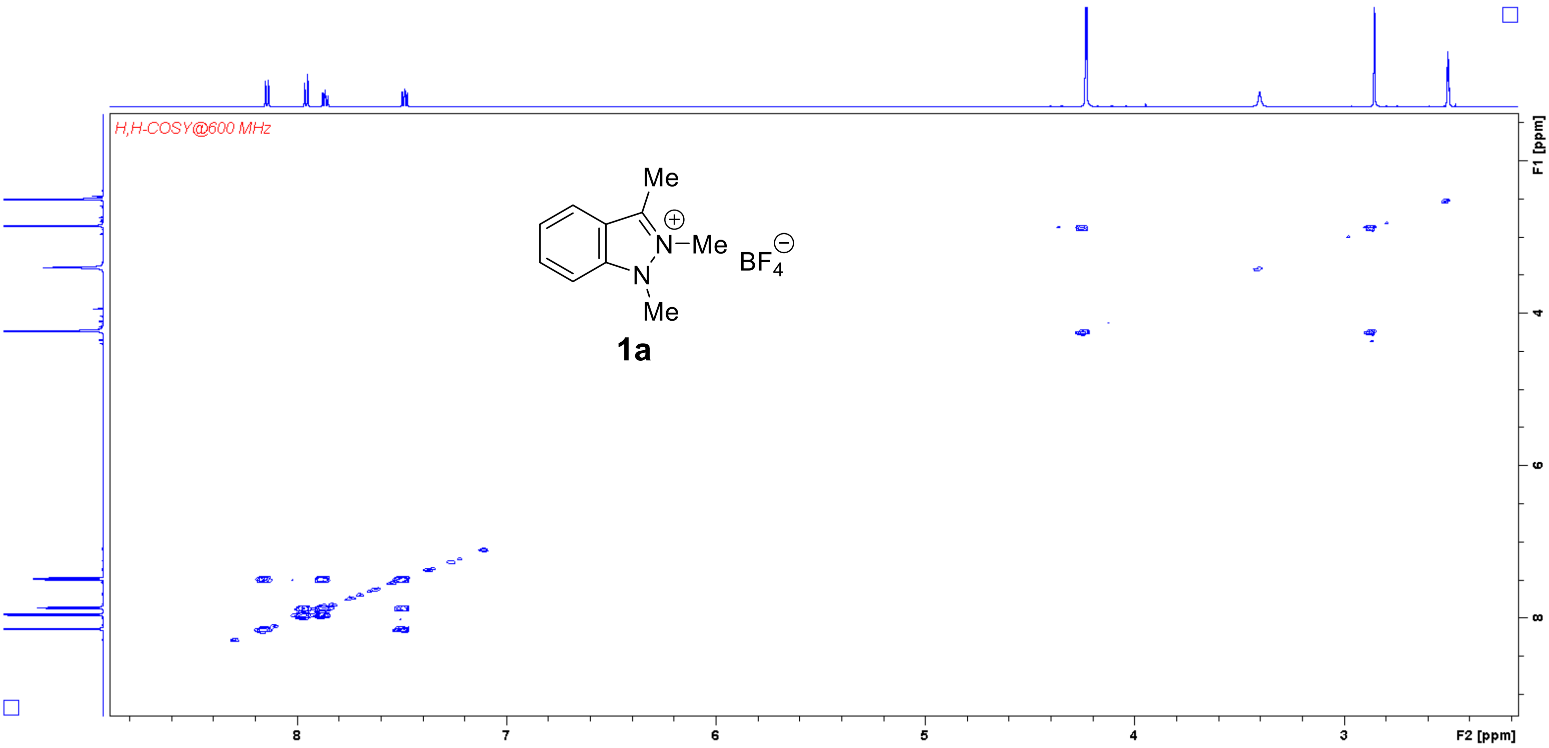
H,H-COSY@600 MHz
Me
N−Me
N
Me
BF4
1a
F1 [ppm]
4
6
8
8
7
6
5
4
3
F2 [ppm]

HMBC

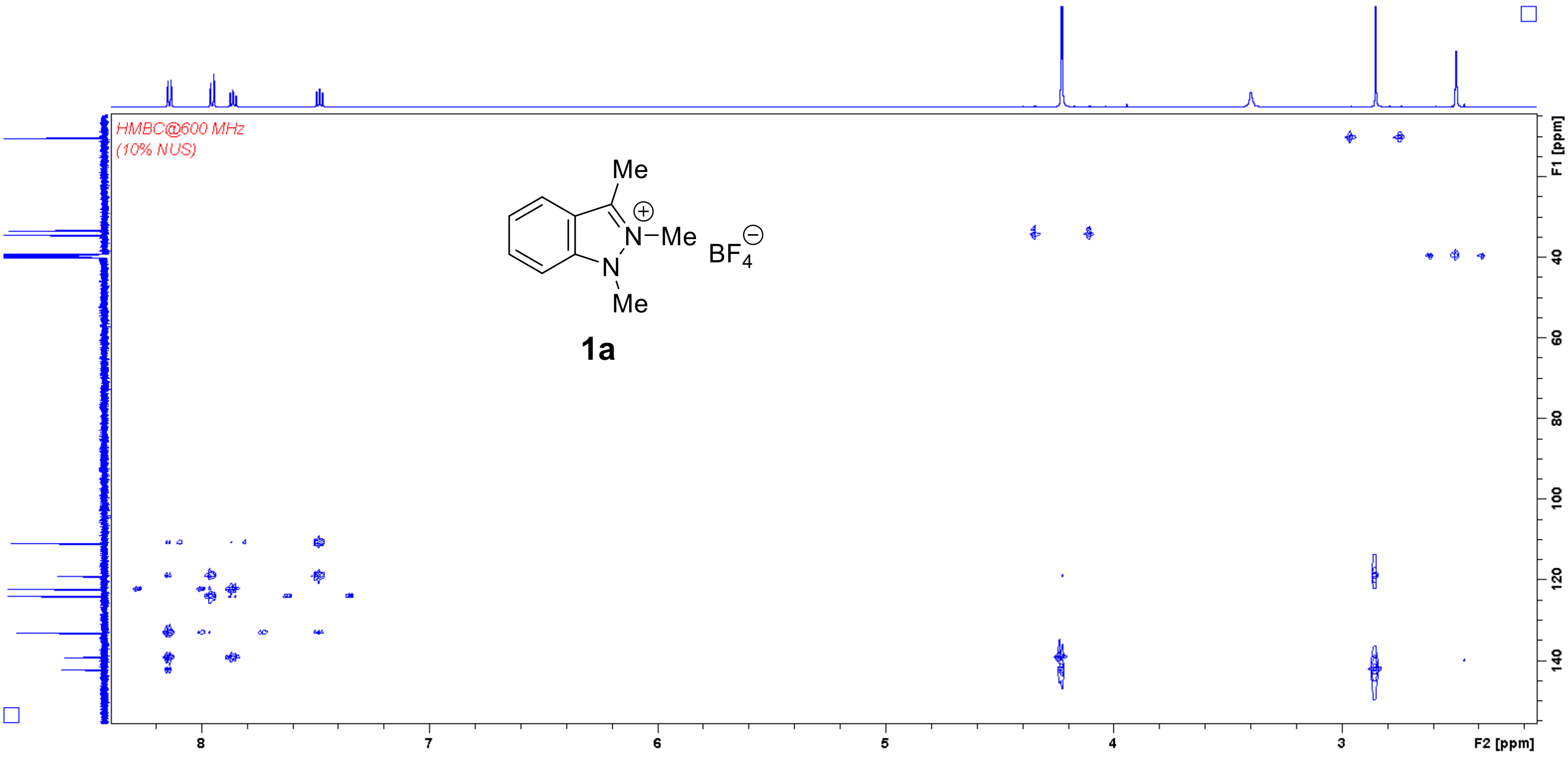
HMBC@600 MHz
(10% NUS)
Me
N−Me
N
Me
BF4
1a
F1 [ppm]
40
60
80
100
120
140
8
7
6
5
4
3
F2 [ppm]

HSQC

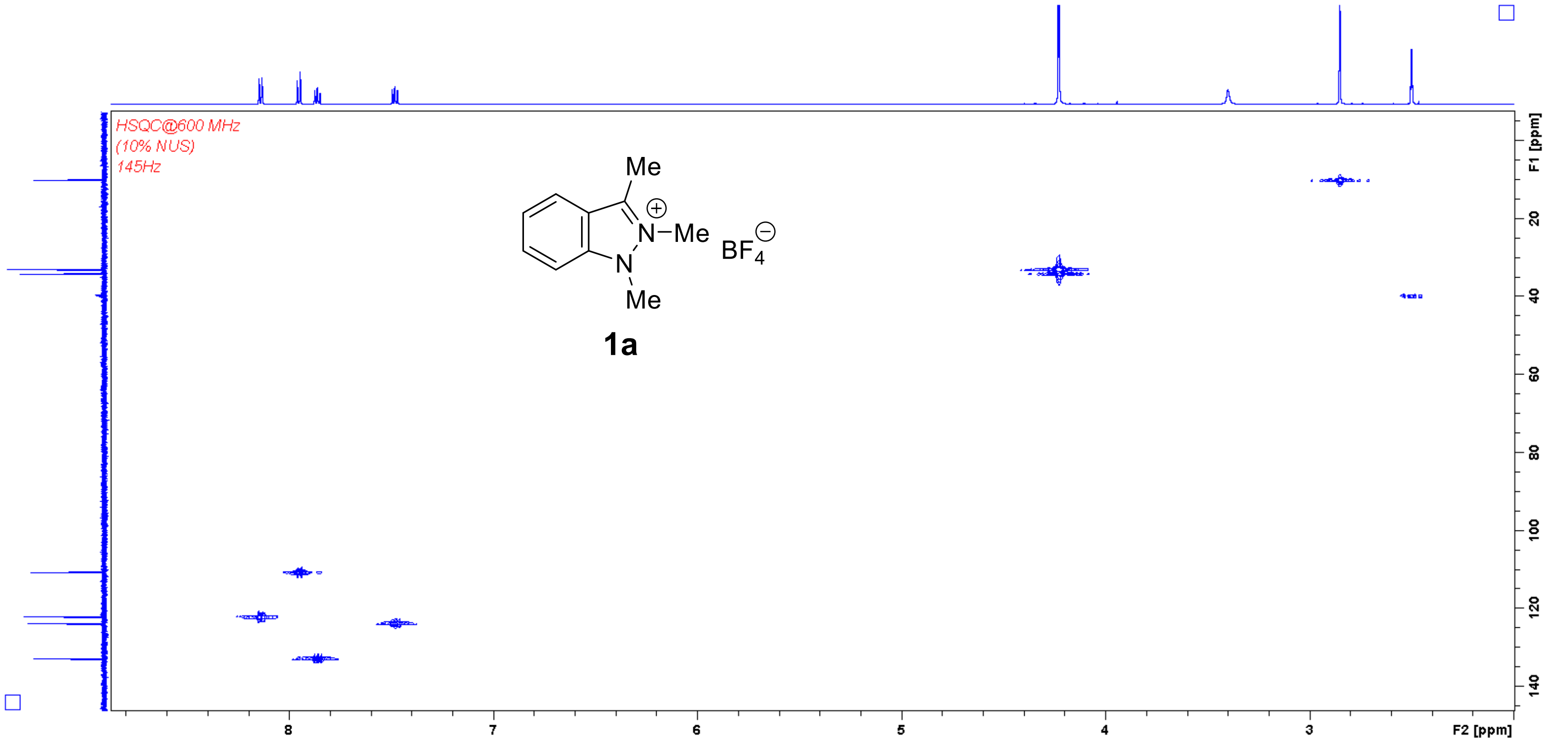
HSQC@600 MHz
(10% NUS)
145Hz
Me
N−Me
BF4
N
Me
1a
F1 [ppm]
20
40
60
80
100
120
140
8
7
6
5
4
3
F2 [ppm]

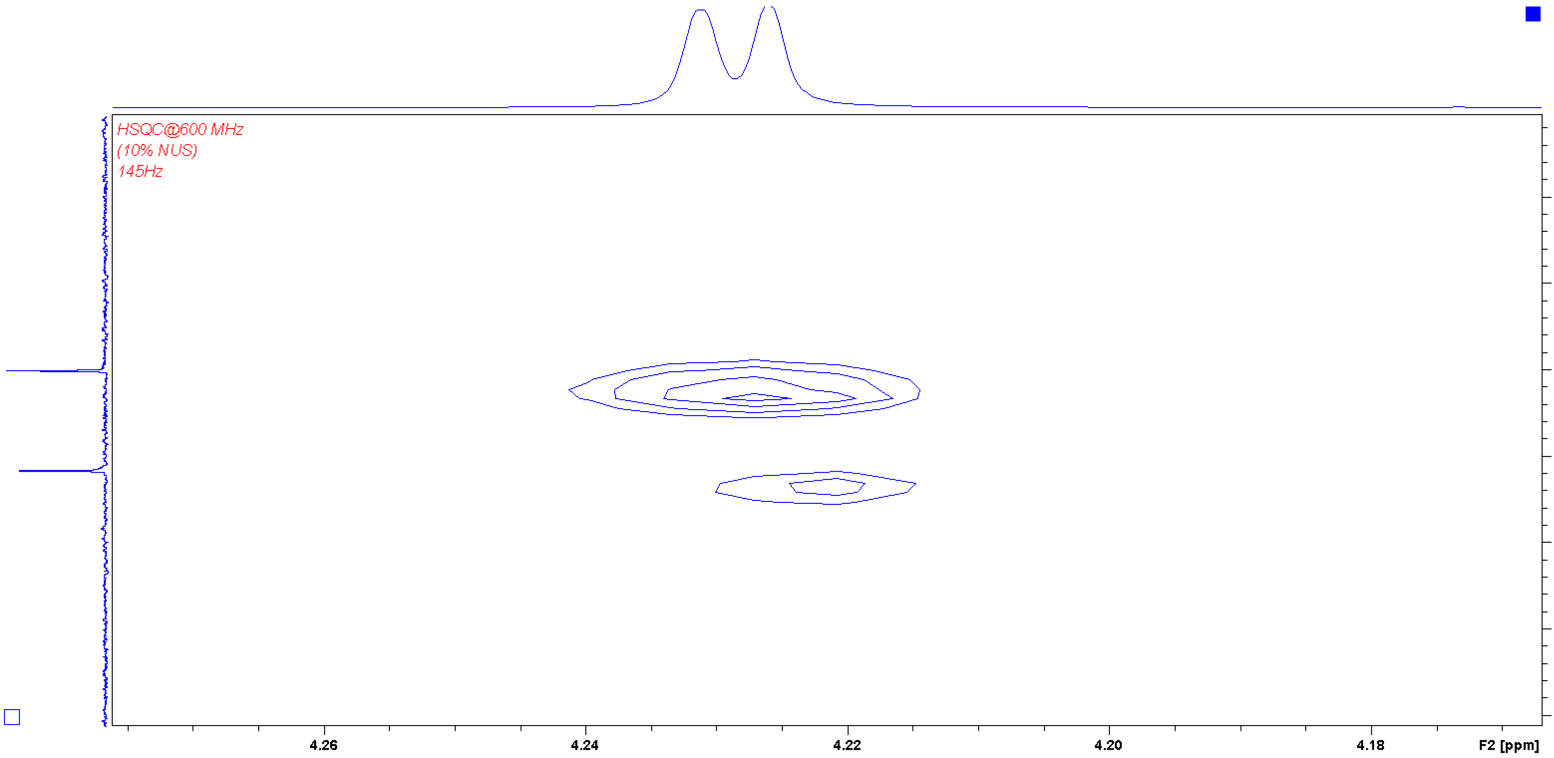
HSQC@600 MHz
(10% NUS)
145Hz
4.26
4.24
4.22
4.20
4.18
F2 [ppm]

Synthesis of 3-methyl-1-phenyl-1H-indazole

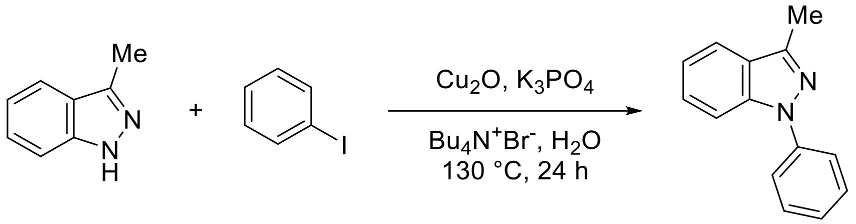

This synthesis is carried out using a ligand free copper (I) catalyzed *N*-arylation according to the literature: Yong, F.-F.; Teo, Y.-C.; Tay, S.-H.; Tan, B. Y.-H.; Lim. K.-H., A ligand-free copper(I) oxide catalyzed strategy for the N-arylation of azoles in water, *Tetrahedron Lett.* **2011**, 52(11), 1161-1164. DOI: 10.1016/j.tetlet.2011.01.005.

In a sealed tube 3-methyl-1*H*-indazole (1.0 Eq, 1.800 g, 13.62 mmol), iodobenzene (1.2 Eq, 3.334 g, 16.34 mmol), cuprous oxide Cu_2O (0.1 Eq, 0.195 g, 1.36 mmol), potassium phosphate K_3PO_4 (2 Eq, 5.781 g, 27.24 mmol), tetrabutylammonium bromide (0.1 Eq, 0.439 g, 1.36 mmol) and water (8 mL) were added. A screw cap was used to seal the reactor. Then the mixture was stirred in a closed system in an oil bath at 130 °C for 24 h. After the reactor was cooled down to room temperature, the mixture was diluted with dichloromethane and then filtered through a pad of Celite. The organic phase was collected and dried with anhydrous $MgSO_4$. After the solvent was dried under vacuum, the crude product was purified by column chromatography (petro ether : ethyl acetate = 4 : 1). The product was obtained as yellow oil (1.764 g, 62%).

^{1}H-NMR ($CDCl_3$, 600 MHz): 7.74-7.70 (m, 4H), 7.53-7.50 (m, 2H), 7.42 (td, J = 7.5, 1.0 Hz, 1H), 7.32 (tt, J = 7.5, 1.0 Hz, 1H), 7.21 (td, J = 7.5, 1.0 Hz, 1H), 2.66 (s, 3H) ppm.

The NMR spectra correspond to the above-mentioned literature.

1H-NMR

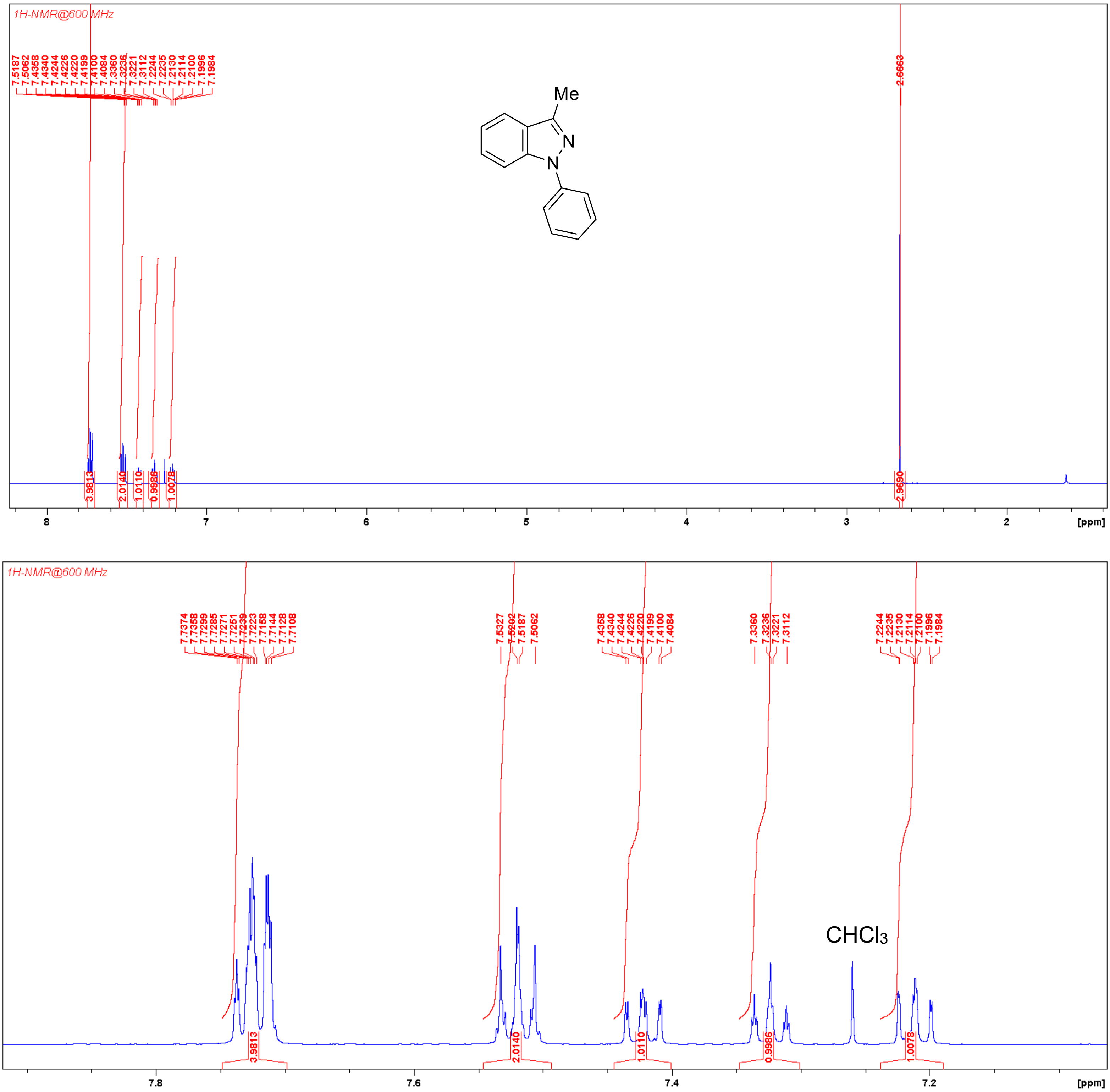

Synthesis of 2,3-dimethyl-1-phenyl-1*H*-indazolium tetrafluoroborate **1b**

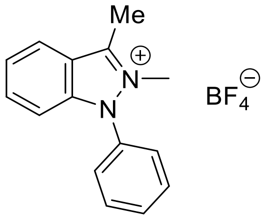

Followed by procedure A, **1b** was synthesized from 1-phenyl-3-dimethyl-1*H*-indazole (1.764 g, 12.71 mmol) in 50 ml anhydrous DCM as white solid (1.444 g, 55%).

^{1}H-NMR (DMSO-d_6, 600 MHz): 8.30 (d, J = 8.1 Hz, 1H, Ar-H), 7.87 -7.78 (m, 4H, Ar-H), 7.77-7.74 (m, 2H, Ar-H), 7.58 (t, J = 8.1 Hz, 1H, Ar-H), 7.34 (d, J = 8.5 Hz, 1H, Ar-H), 3.97 (s, 3H, N-Me), 2.97 (s, 3H, Me) ppm.

^{13}C{^{1}H}-NMR (DMSO-d_6, 150 MHz): 144.7 (o, Ar-C), 139.8 (o, Ar -C), 134.2 (+, Ar-C), 132.2 (+, Ar-C), 131.2 (o, Ar-C), 130.7 (+, Ar-C), 129.0 (+, Ar-C), 124.7 (+, Ar-C), 122.6 (+, Ar-C), 119.4 (o, Ar-C), 110.6 (+, Ar-C), 35.3 (+, N-Me), 10.6 (+, Me) ppm.

ESI-MS (m/z): calculated for $[C_{15}H_{15}N_2]^+$: 223.1230, found 223.1233.

IR (ATR): $\tilde{\nu}$ = 1047, 1035, (indazole ring vibration), 759 (BF_4^-) cm^{-1}.

Melting point: 170-171 °C.

^{1}H-NMR

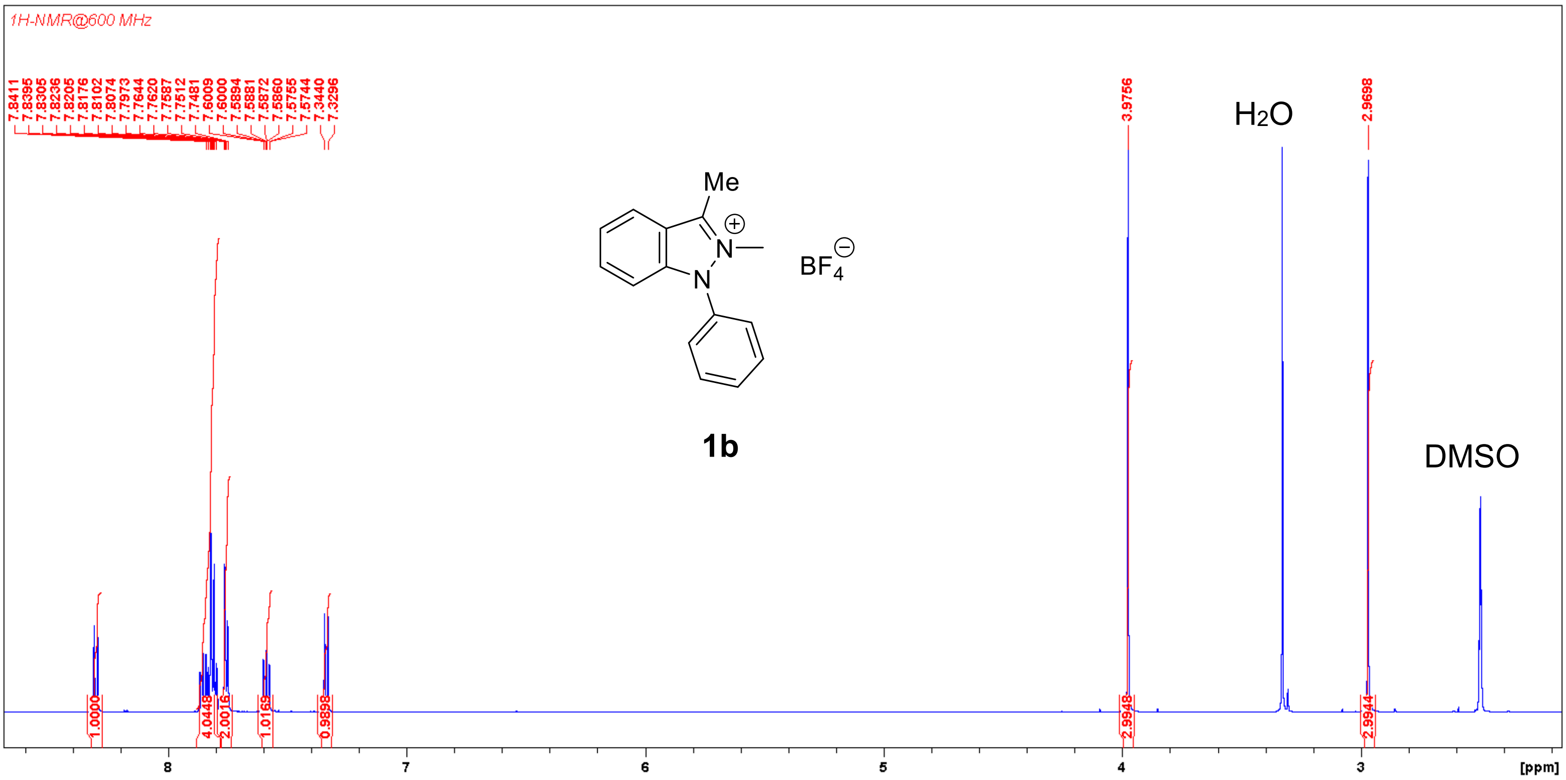

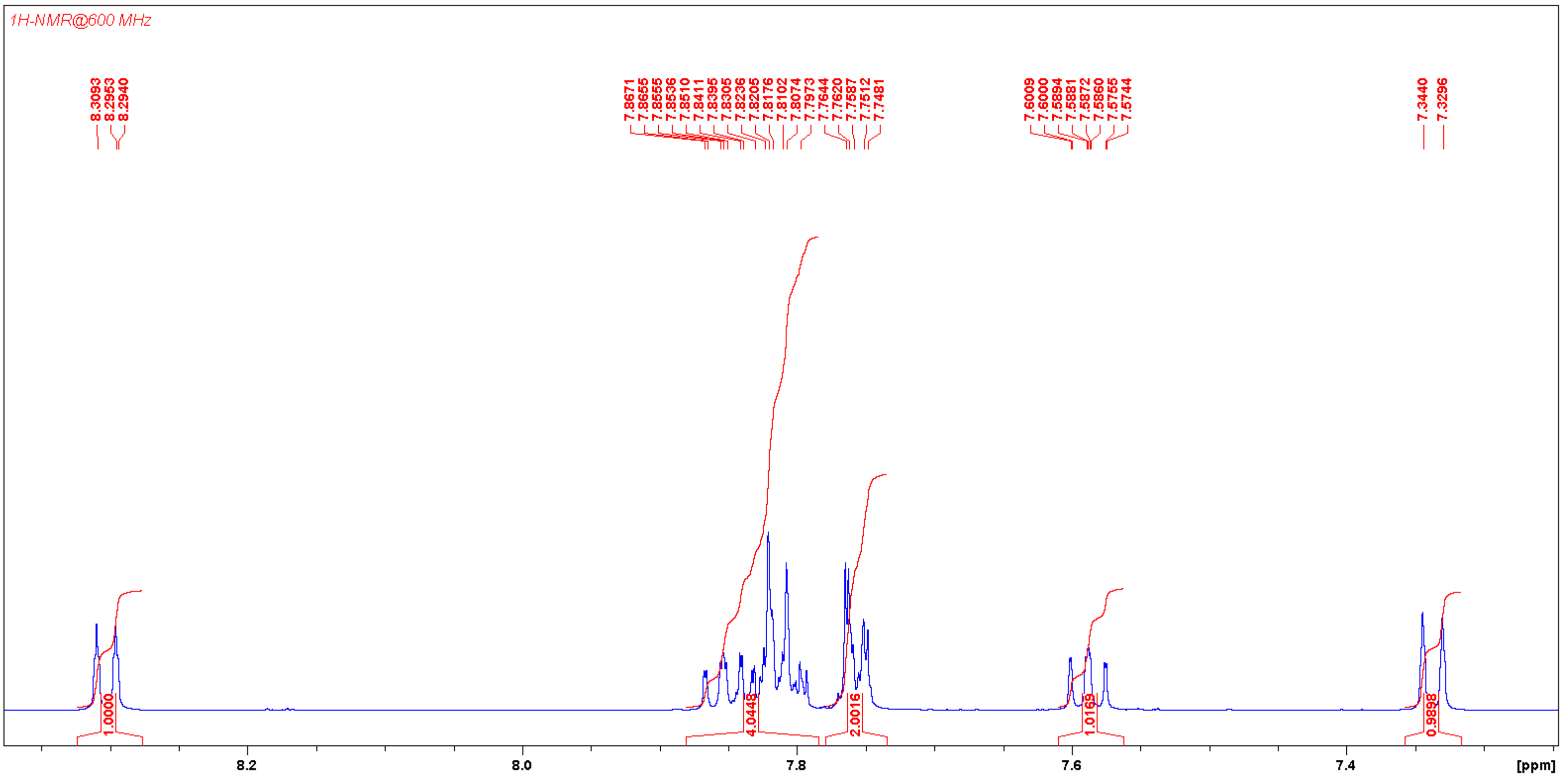

$^{13}C\{^{1}H\}$-NMR

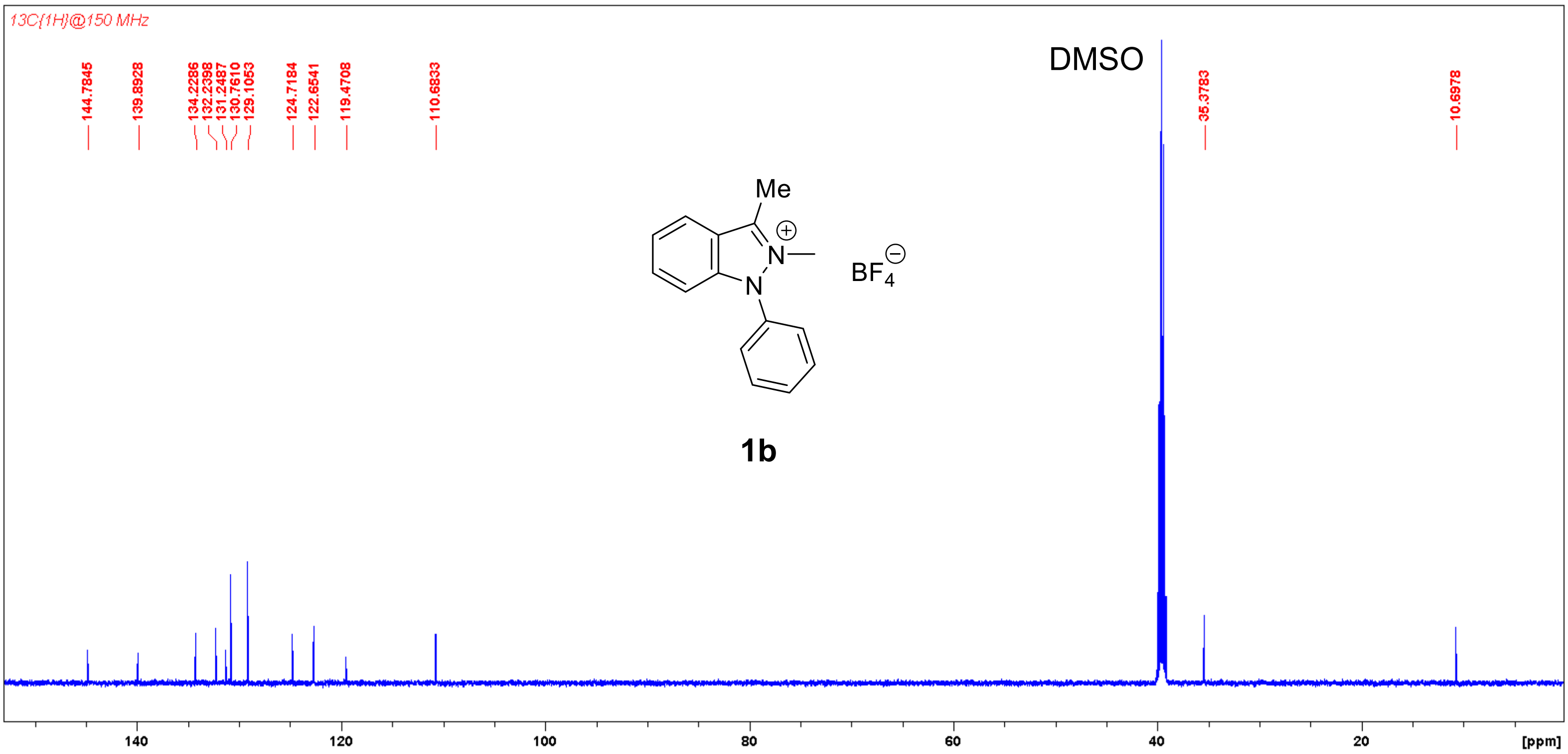

^{13}C-DEPT

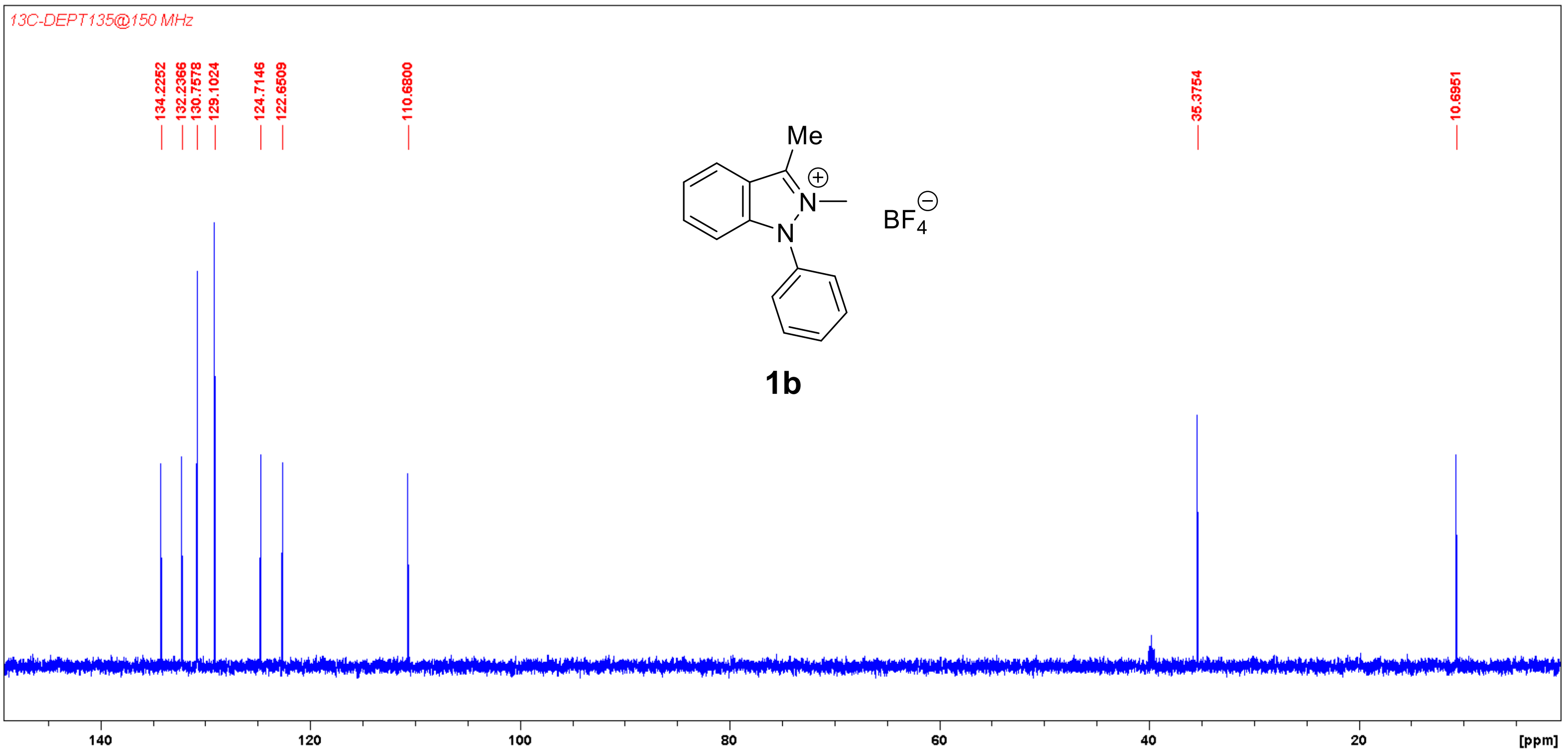

H,H-COSY

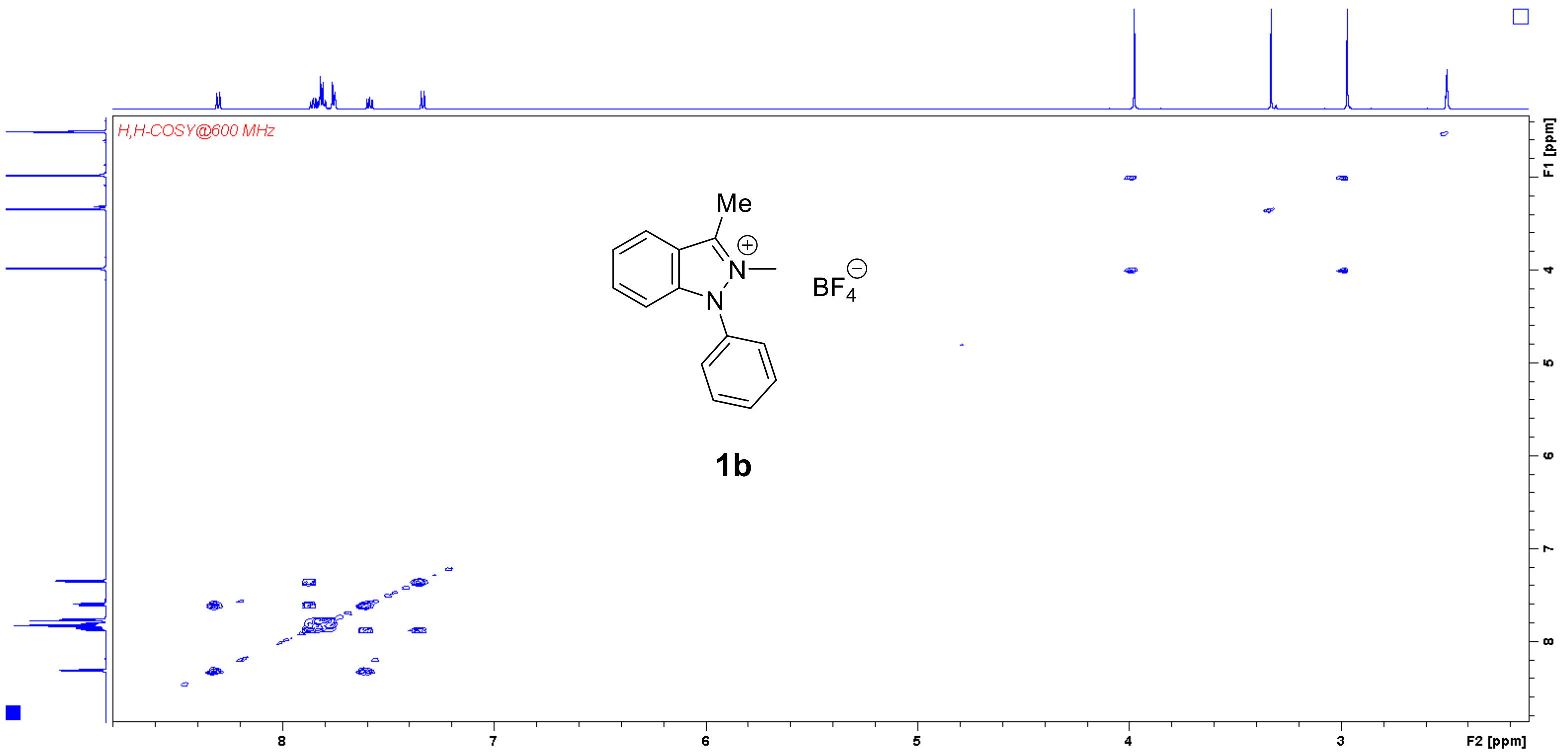
H,H-COSY@600 MHz
Me
N
N
BF4
1b
F1 [ppm]
F2 [ppm]

HMBC

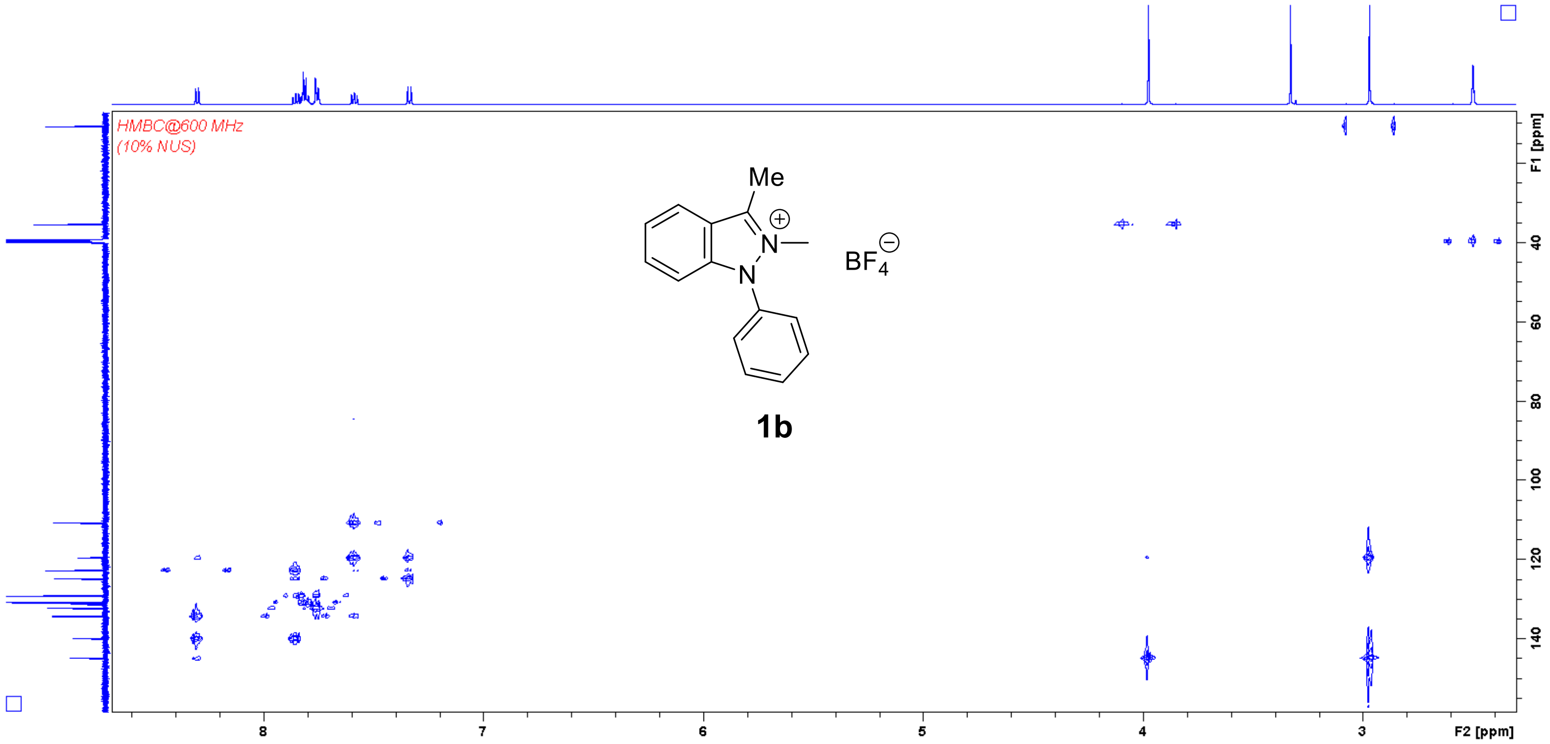
HMBC@600 MHz
(10% NUS)
Me
N
N
BF4
1b
F1 [ppm]
F2 [ppm]

HSQC

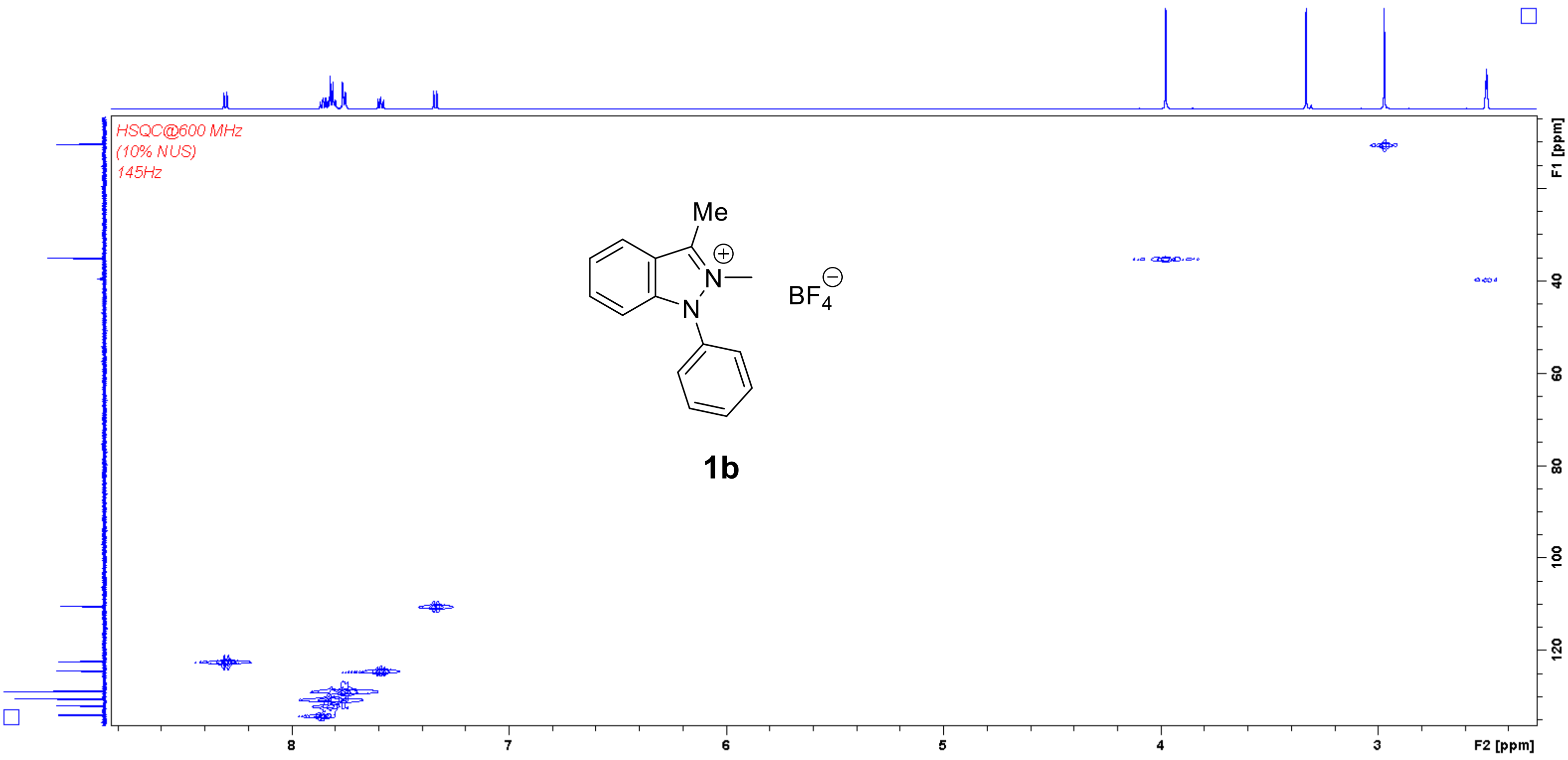

HSQC@600 MHz
(10% NUS)
145Hz
Me
N
N
BF4
1b
F1 [ppm]
F2 [ppm]

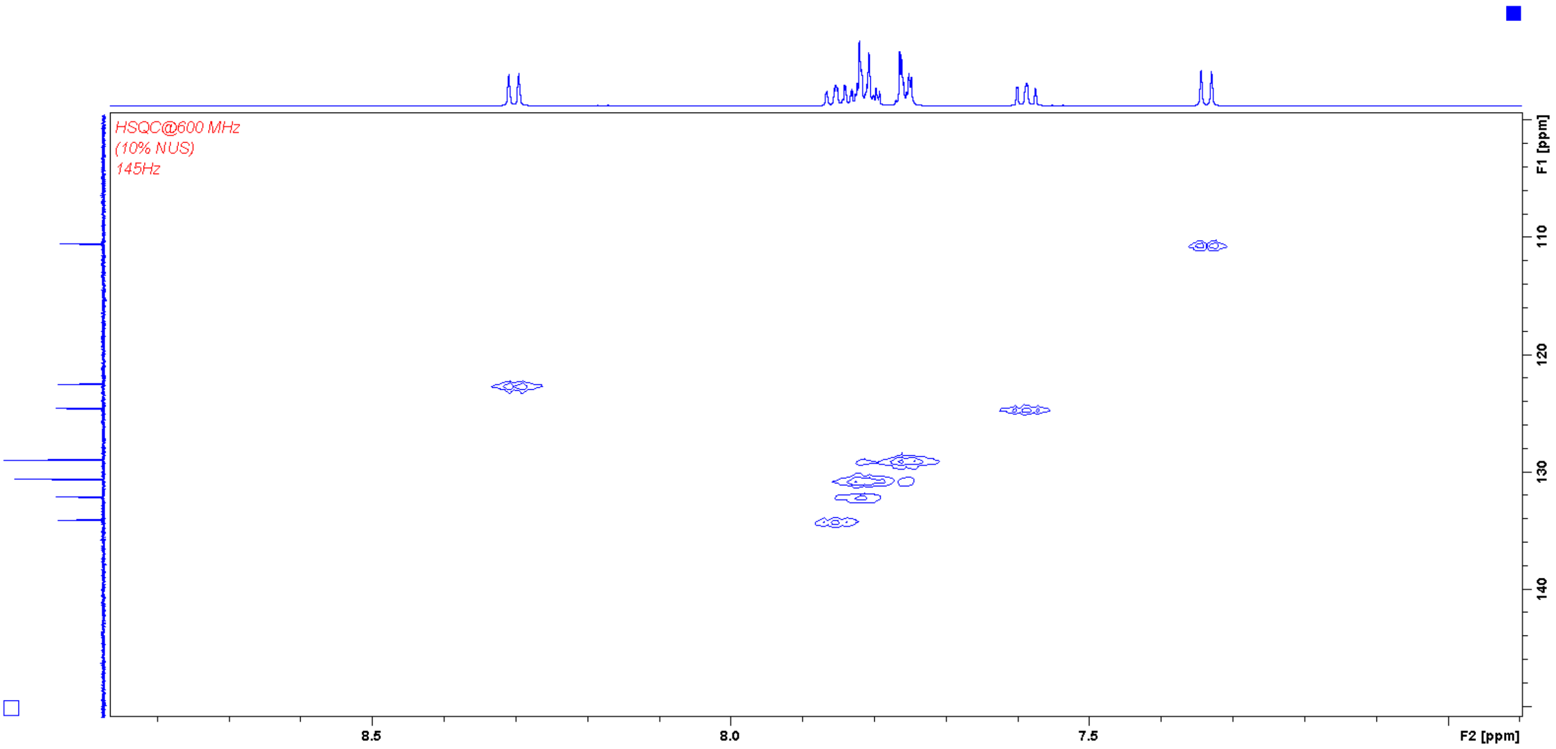

HSQC@600 MHz
(10% NUS)
145Hz
F1 [ppm]
F2 [ppm]

Synthesis of 1-(4-methoxylphenyl)-3-methyl-1*H*-indazole

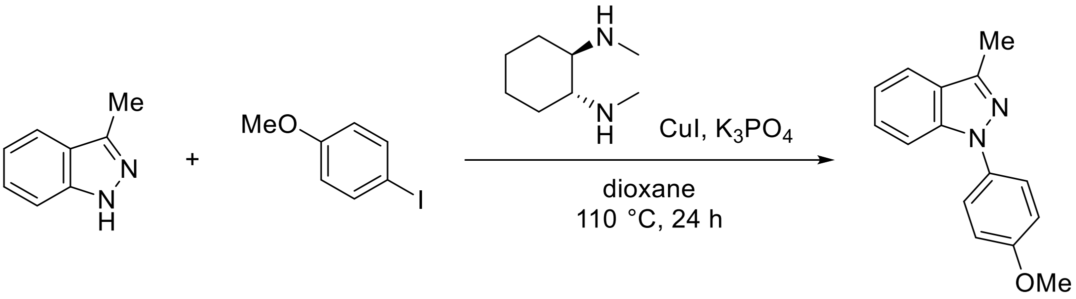

This synthesis is carried out using copper (I) catalyzed *N*-arylation according to the literature: Guan, Z.; Wiechmann, S.; Drafz, M.; Hübner, E., Schmidt, A., Pericyclic rearrangements of N-heterocyclic carbenes of indazole to substituted 9-aminoacridines. *Org. Biomol. Chem.* **2013**, 11, 3558-3567. DOI: 10.1039/C3OB40379C

In a flask 3-methyl-1*H*-indazole (1.0 Eq, 500 mg, 3.78 mmol), 4-iodoanisole (1.2 Eq, 1.062 g, 4.54 mmol), cuprous iodide CuI (0.1 Eq, 72 mg, 0.378 mmol), potassium phosphate K_3PO_4 (2.0 Eq, 1.606 g, 7.56 mmol), trans-*N,N'*-dimethylcyclohexane-1,2-diamine (0.1 Eq, 54 mg, 0.378 mmol) and dioxane (10 mL) were added. The mixture was stirred in an oil bath at reflux temperature for 24 h. After the reactor was cooled down to room temperature, the mixture was dried under vacuum and purified by column chromatography. The product was obtained as yellow oil (891 mg, 99%).

^{1}H-NMR ($CDCl_3$, 600 MHz): 7.72 (dt, J = 7.8, 1.0 Hz, 1H,), 7.61-7.57 (m, 3H,), 7.39 (td, J = 7.8, 1.0 Hz, 1H), 7.18 (td, J = 7.8, 1.0 Hz, 1H), 7.05-7.02 (m, 2H,), 3.87 (s, 3H), 2.65 (s, 3H) ppm.

The NMR spectra correspond to the literature: Arepally, S.; Kim, T.; Kim, G.; Yang, H.; Park, J. K., Exploring Synthetic Strategies for 1*H*-Indazoles and Their *N*-Oxides: Electrochemical Synthesis of 1*H*-Indazole *N*-Oxides and Their Divergent C−H Functionalizations. *Angew. Chem., Int. Ed.*, **2023**, 62, e202303460.

^{1}H-NMR

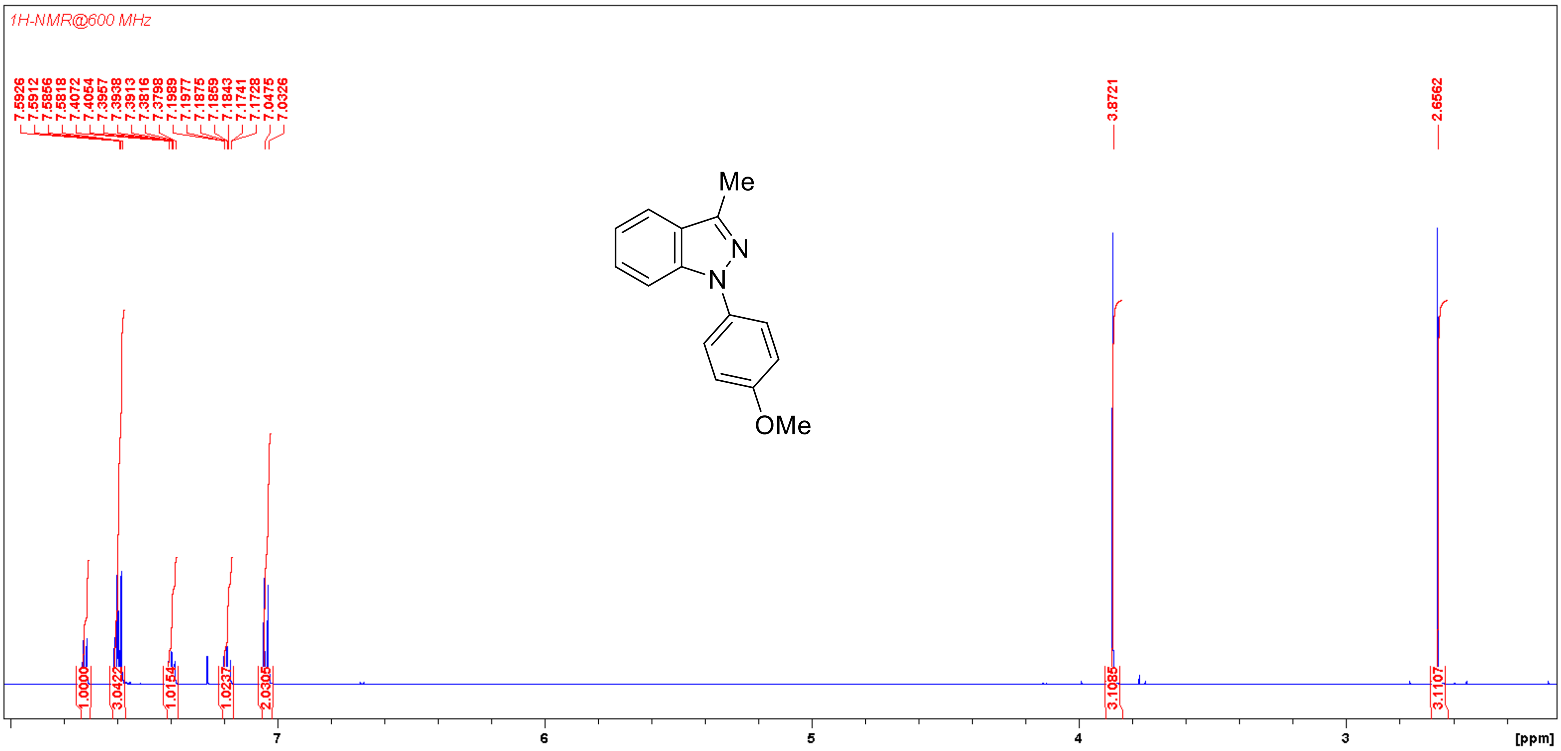
1H-NMR@600 MHz
7.5926
7.5912
7.5856
7.5818
7.4072
7.4054
7.3957
7.3938
7.3913
7.3816
7.3798
7.1989
7.1977
7.1875
7.1859
7.1843
7.1741
7.1728
7.0475
7.0326
3.8721
2.6562
Me
N
N
OMe
1.0000
3.0422
1.0154
1.0237
2.0305
3.1085
3.1107
7
6
5
4
3
[ppm]

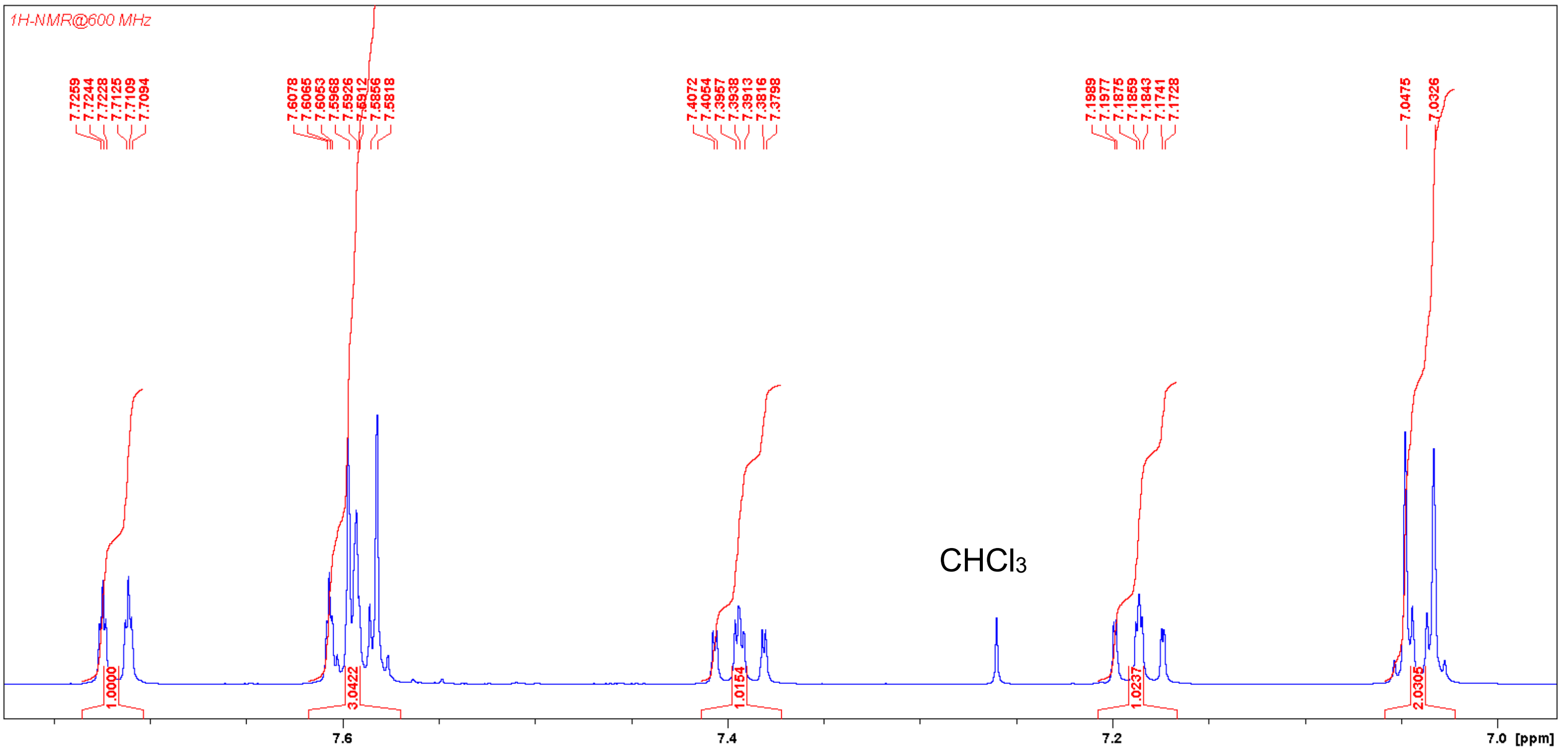
1H-NMR@600 MHz
7.7259
7.7244
7.7228
7.7125
7.7109
7.7094
7.6078
7.6065
7.6053
7.5968
7.5926
7.5912
7.5856
7.5818
7.4072
7.4054
7.3957
7.3938
7.3913
7.3816
7.3798
7.1989
7.1977
7.1875
7.1859
7.1843
7.1741
7.1728
7.0475
7.0326
CHCl3
1.0000
3.0422
1.0154
1.0237
2.0305
7.6
7.4
7.2
7.0
[ppm]

Synthesis of 2,3-dimethyl-1-(4-methoxylphenyl)-1*H*-indazolium iodide **1c**

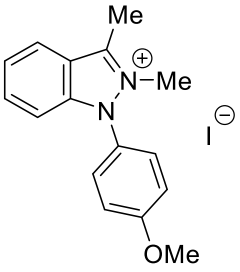

Followed by procedure B, **1c** was synthesized from 1-(4-methoxylphenyl)-3-methyl-1*H*-indazole (893 mg, 3.75 mmol) in 10 mL acetonitrile as pale solid (1.203 g, 84%).

^{1}H-NMR (DMSO-d$_6$, 600 MHz): 8.27 (d, J = 7.8 Hz, 1H, Ar-H), 7.83 (td, J = 7.8, 1.0 Hz, 1H, Ar-H), 7.68-7.65 (m, 2H, Ar-H), 7.56 (t, J = 7.8 Hz, 1H, Ar-H), 7.33-7.29 (m, 3H, Ar-H), 3.94 (s, 3H, OMe), 3.91 (s, 3H, N-Me), 2.95 (s, 3H, Me) ppm.

^{13}C{^{1}H}-NMR (DMSO-d$_6$, 150 MHz): 161.7 (o, Ar-C), 144.1 (o, Ar-C), 140.1 (o, Ar-C), 134.0 (+, Ar-C), 130.8 (+, Ar-C), 124.6 (+, Ar-C), 123.3 (o, Ar-C), 122.6 (+, Ar-C), 119.3 (o, Ar-C), 115.8 (+, Ar-C), 110.7 (+, Ar-C), 55.9 (+, OMe), 35.0 (+, N-Me), 10.7 (+, Me) ppm.

ESI-MS (m/z): calculated for $[C_{16}H_{17}N_2O]^+$: 253.1341, found 253.1345.

IR (ATR): $\tilde{\nu}$ =1252 (indazole ring), 559 (C-O-C deformation) cm^{-1}.

Melting point: 196-197 °C

^{1}H-NMR

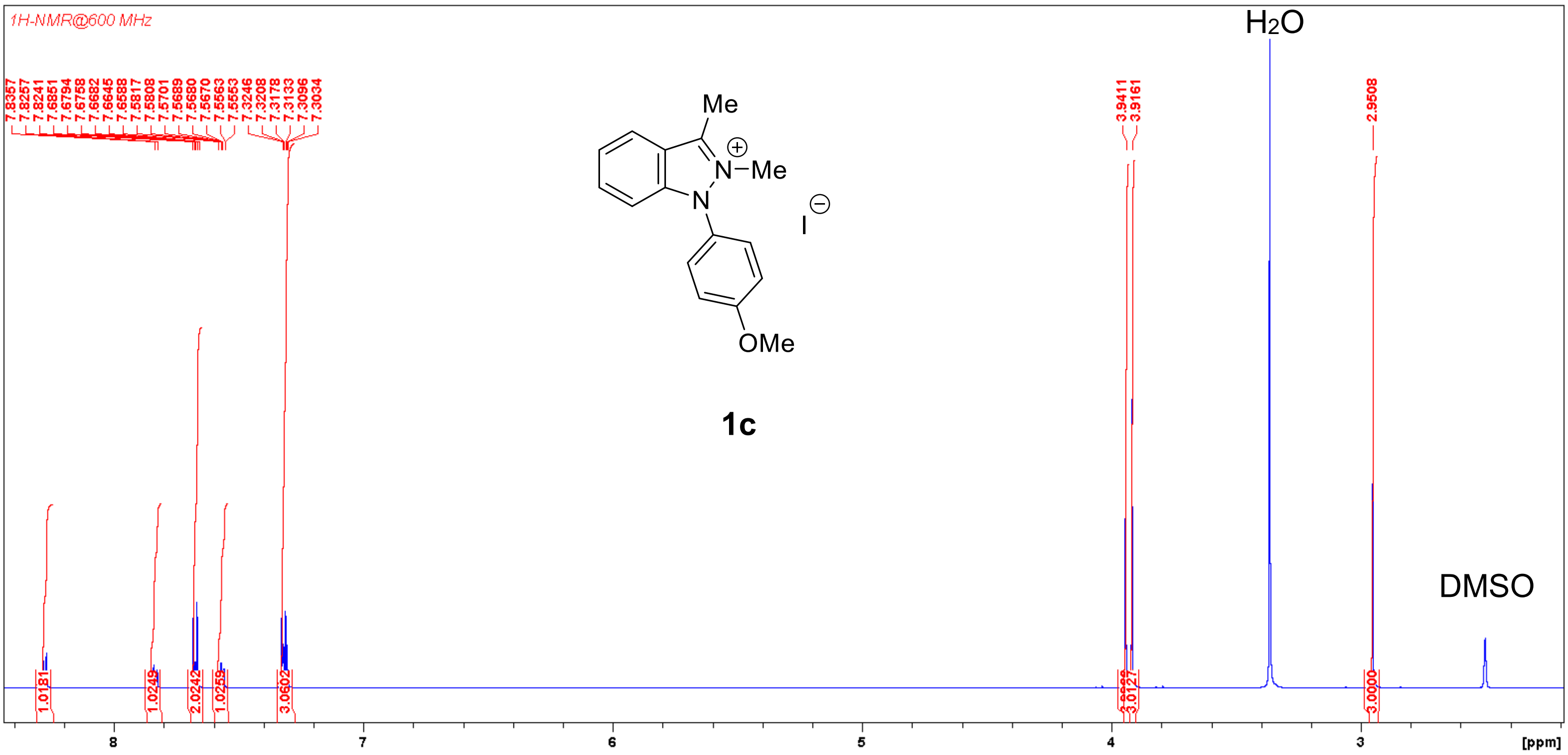

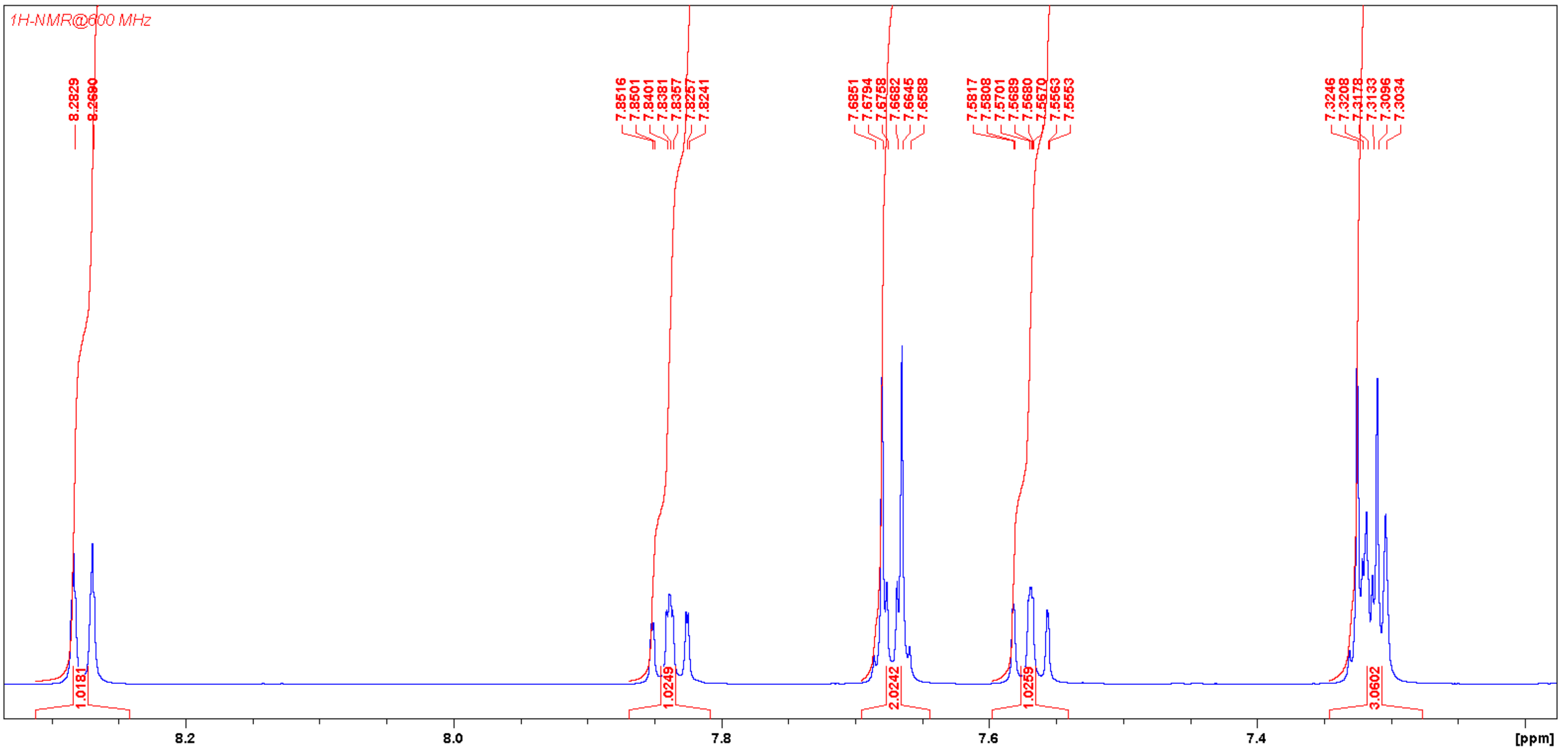

^{13}C-NMR

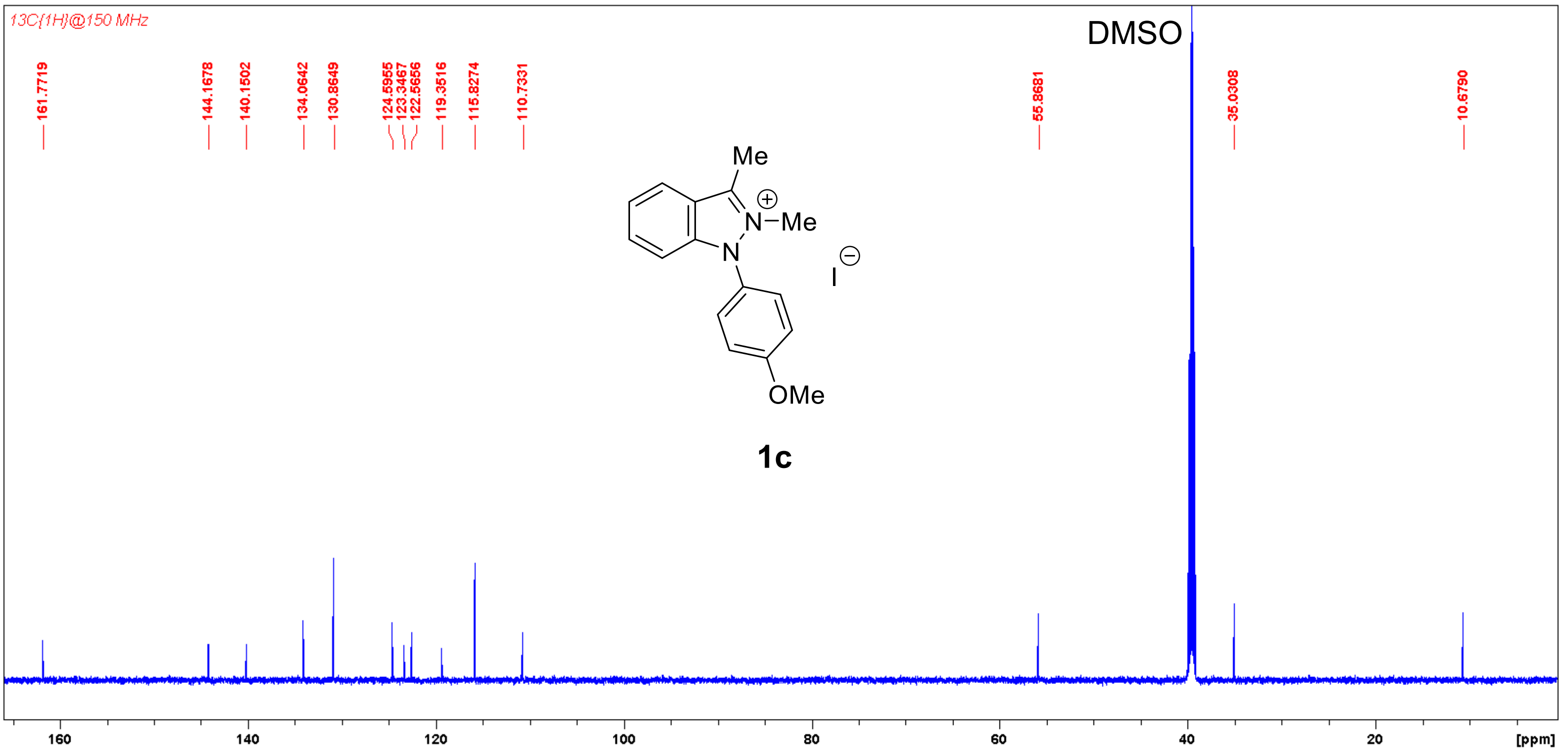

^{13}C{^{1}H}-DEPT

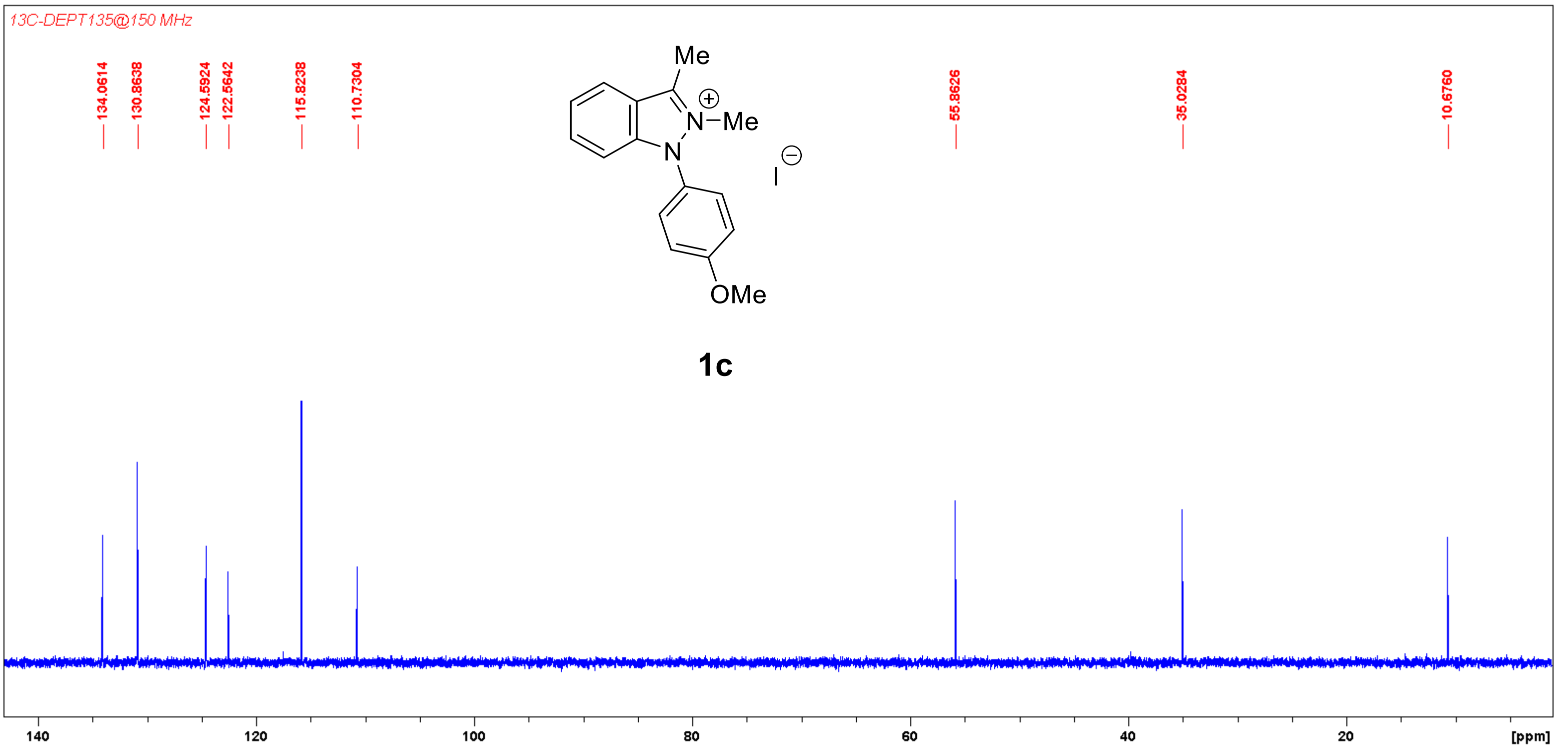

H,H-COSY

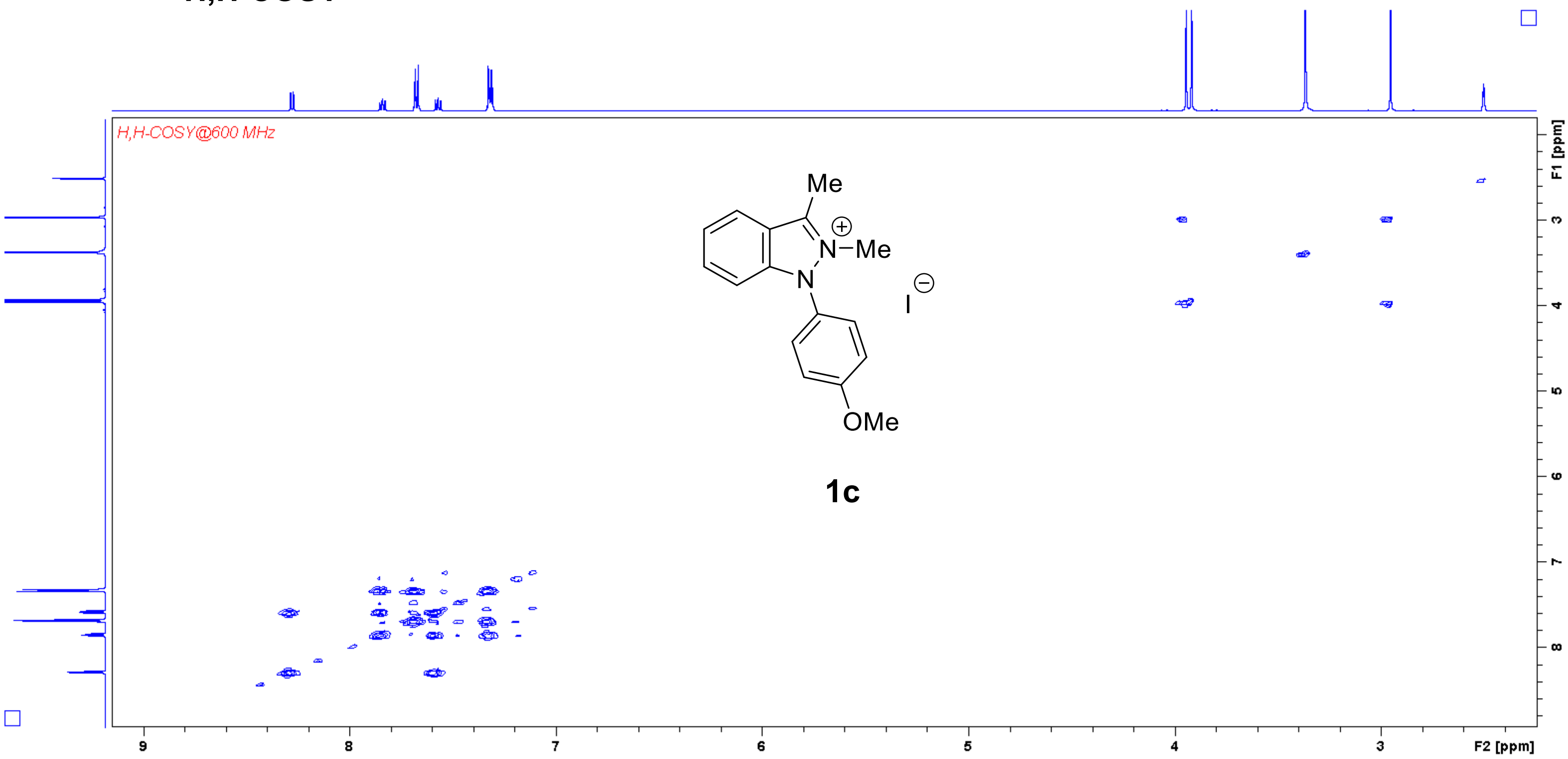

H,H-COSY@600 MHz
Me
N−Me
N
I
OMe
1c
F1 [ppm]
F2 [ppm]

HMBC

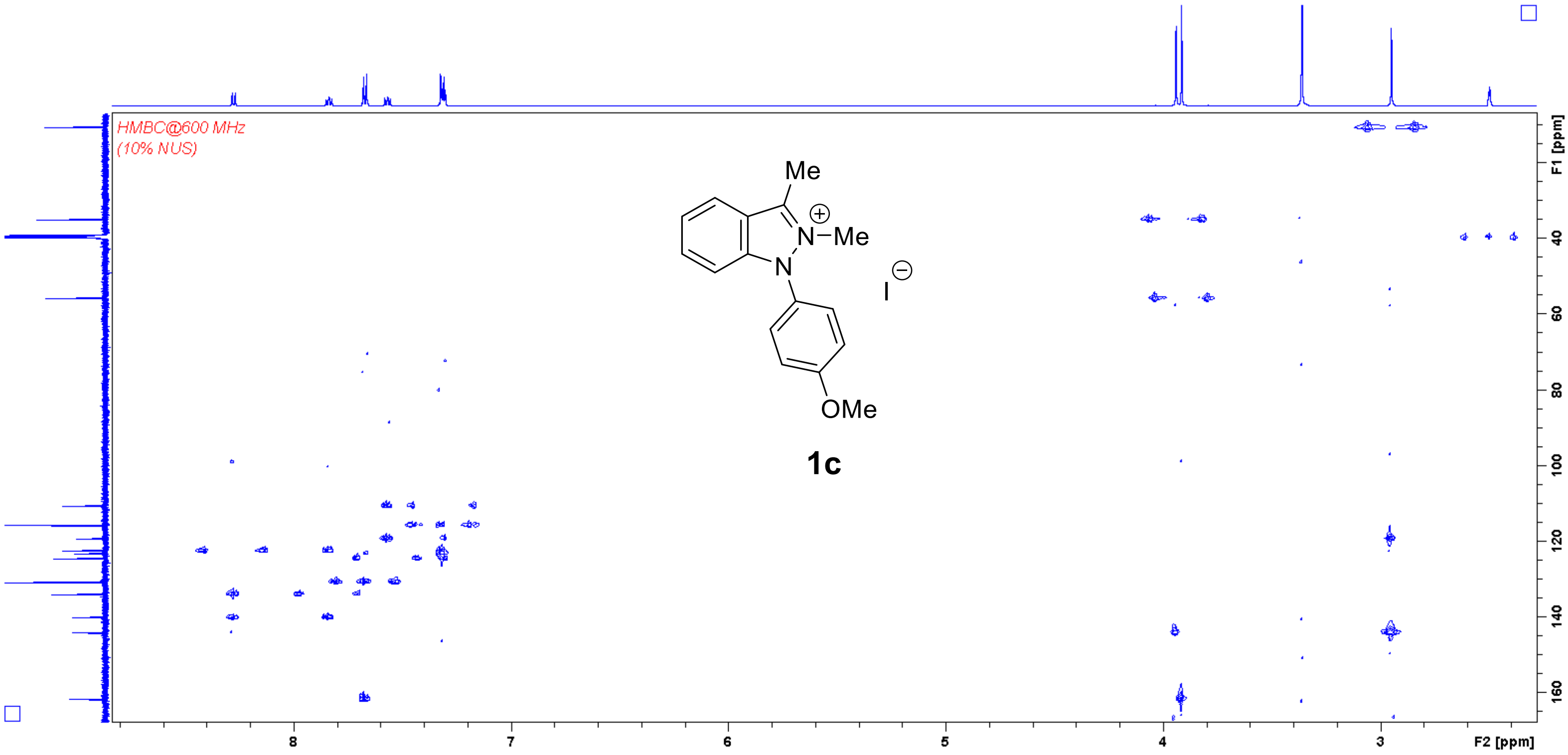

HMBC@600 MHz
(10% NUS)
Me
N−Me
N
I
OMe
1c
F1 [ppm]
F2 [ppm]

HSQC

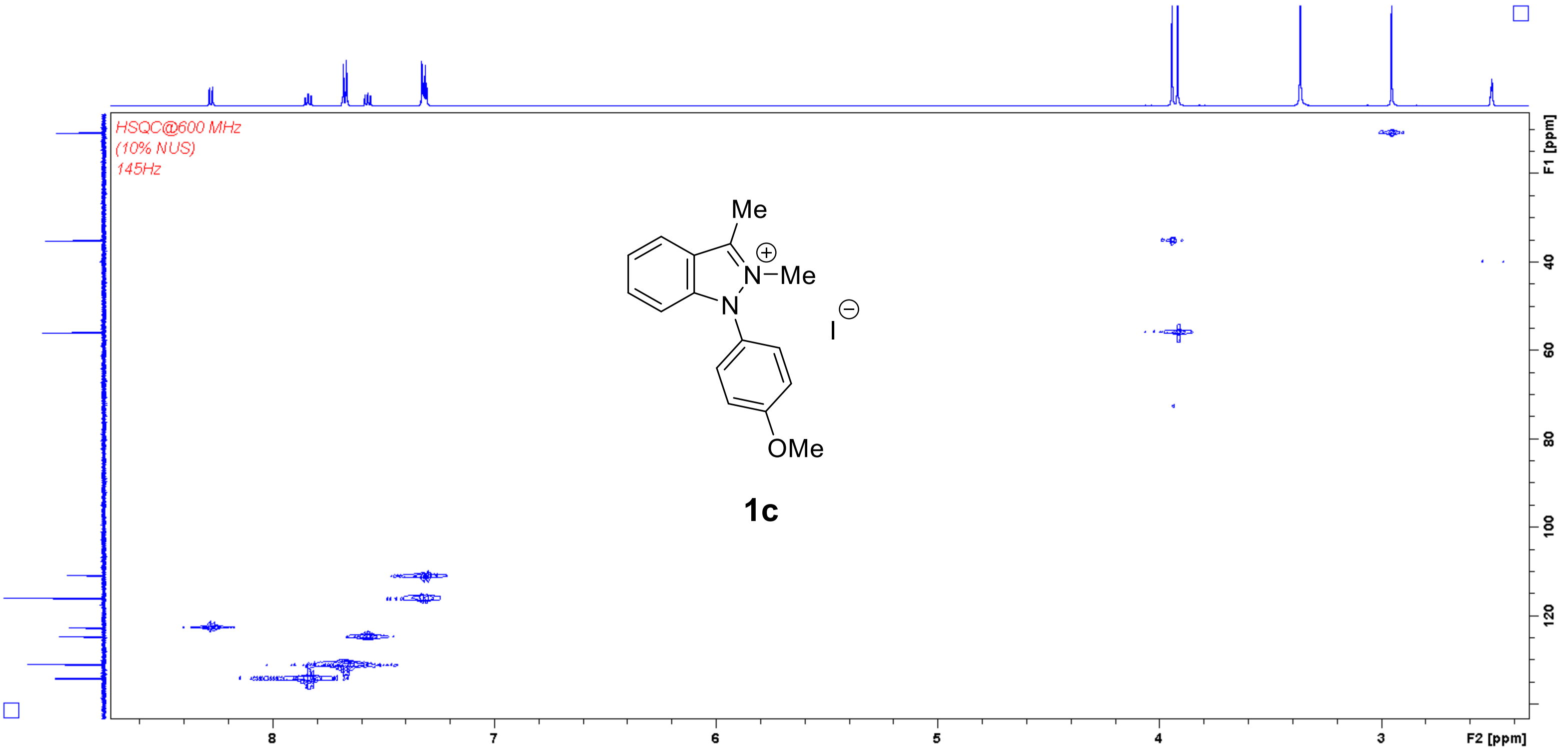

Synthesis of 1,3-dimethyl-4-methoxyl-1*H*-indazole

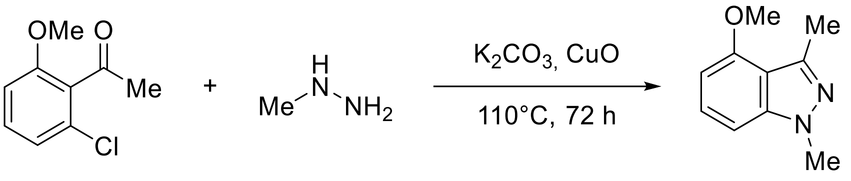

This indazole synthesis is carried out based on the literature: Counceller, C. M.; Eichman, C. C.; Wray, B. C.; Stambuli, J. P., A Practical, Metal-Free Synthesis of 1*H*-Indazoles. *Org. Lett.* **2008**, *10* (5), 1021-1023. DOI: 10.1021/ol800053f.

An oven-dried sealed tube was charged with CuO (2 mol %, 0.216 mmol, 17.2 mg) and K_2CO_3 (1.5 Eq, 16.25 mmol, 2.246 g), evacuated, and refilled with nitrogen. 2-Chloroacetophenone (1 Eq, 10.83 mmol, 2.000 g) and methylhydrazine (6.4 Eq, 69.33 mmol, 3.194 g) were added under nitrogen. The reaction was heated to 110 ºC in an oil bath for 72 h. The reaction mixture was cooled to room temperature and diluted with ethyl acetate. After the suspension was filtrated, the organic phase was washed with water, concentrated and purified by flash chromatography on silica gel. The desired product was afforded by removing the solvents as light-yellow oil (677 mg, 35%).

^{1}H-NMR ($CDCl_3$, 600 MHz): 7.23 (t, J = 7.9 Hz, 1H, Ar-H), 6.86 (d, J = 7.9 Hz, 1H, Ar-H), 6.38 (d, J = 7.9 Hz, 1H, Ar-H), 3.93 (s, 3H, N-Me), 3.92 (s, 3H, OMe), 2.66 (s, 3H, Me) ppm.

^{13}C{^{1}H}-NMR ($CDCl_3$, 150 MHz): 155.4 (o, Ar-C), 143.1 (o, Ar-C), 141.3 (o, Ar-C), 127.7 (+, Ar-C), 114.4 (o, Ar-C), 101.7 (+, Ar-C), 98.7 (+, Ar-C), 55.3 (+, OMe), 35.3 (+, N-Me), 14.2 (+, Me) ppm.

ESI-MS (m/z): calculated for $[C_{10}H_{12}N_2O+Na]^+$: 199.0842, found 199.0834.

IR (ATR): $\tilde{\nu}$ =1257 (indazole ring), 545 (C-O-C deformation) cm^{-1}.

^{1}H-NMR

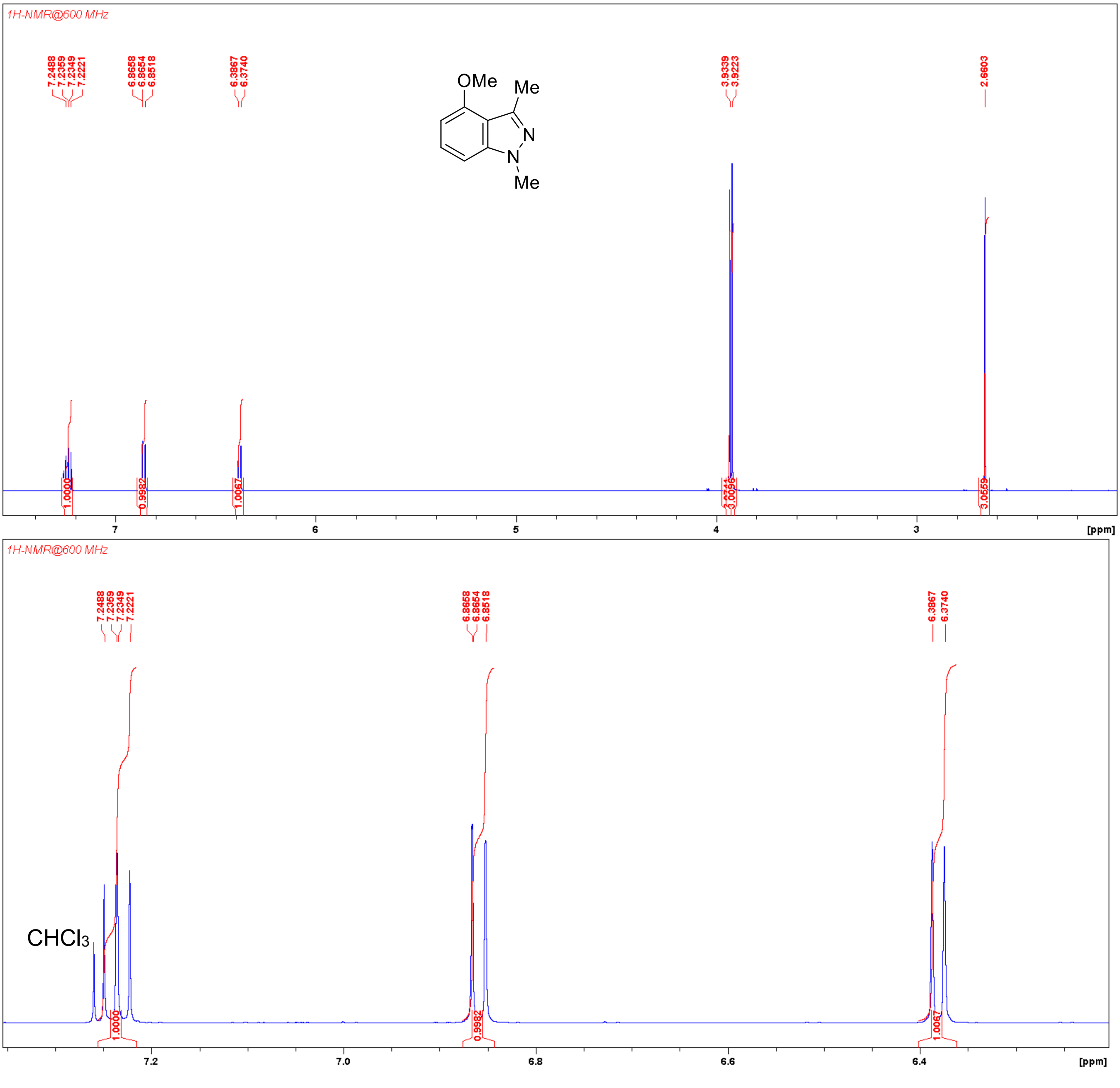
1H-NMR@600 MHz
7.2488
7.2359
7.2349
7.2221
6.8658
6.8654
6.8518
6.3867
6.3740
3.9339
3.9223
2.6603
OMe
Me
N
N
Me
1.0000
0.9982
1.0067
3.0056
3.0559
[ppm]
CHCl3

$^{13}C\{^1H\}$-NMR

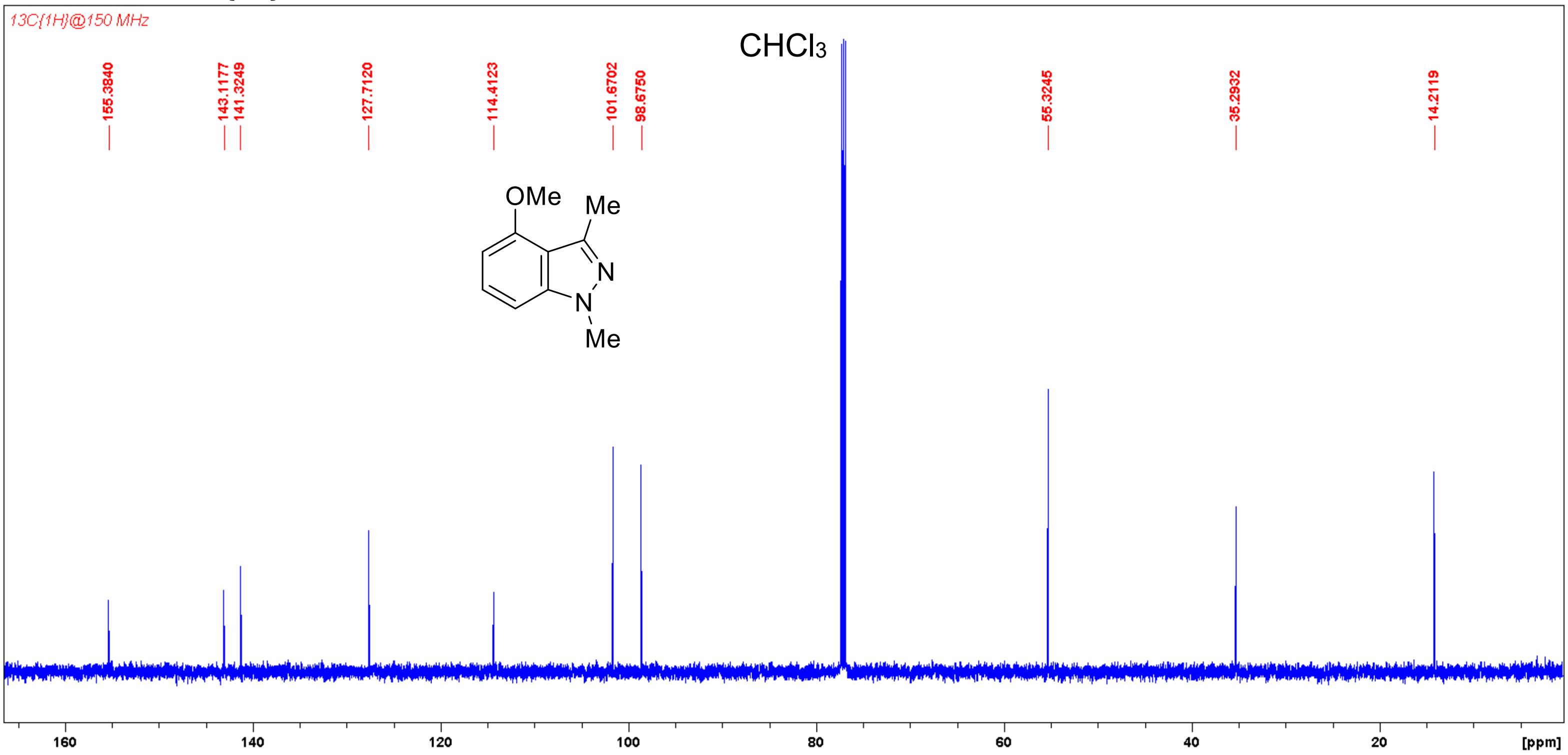

^{13}C-DEPT

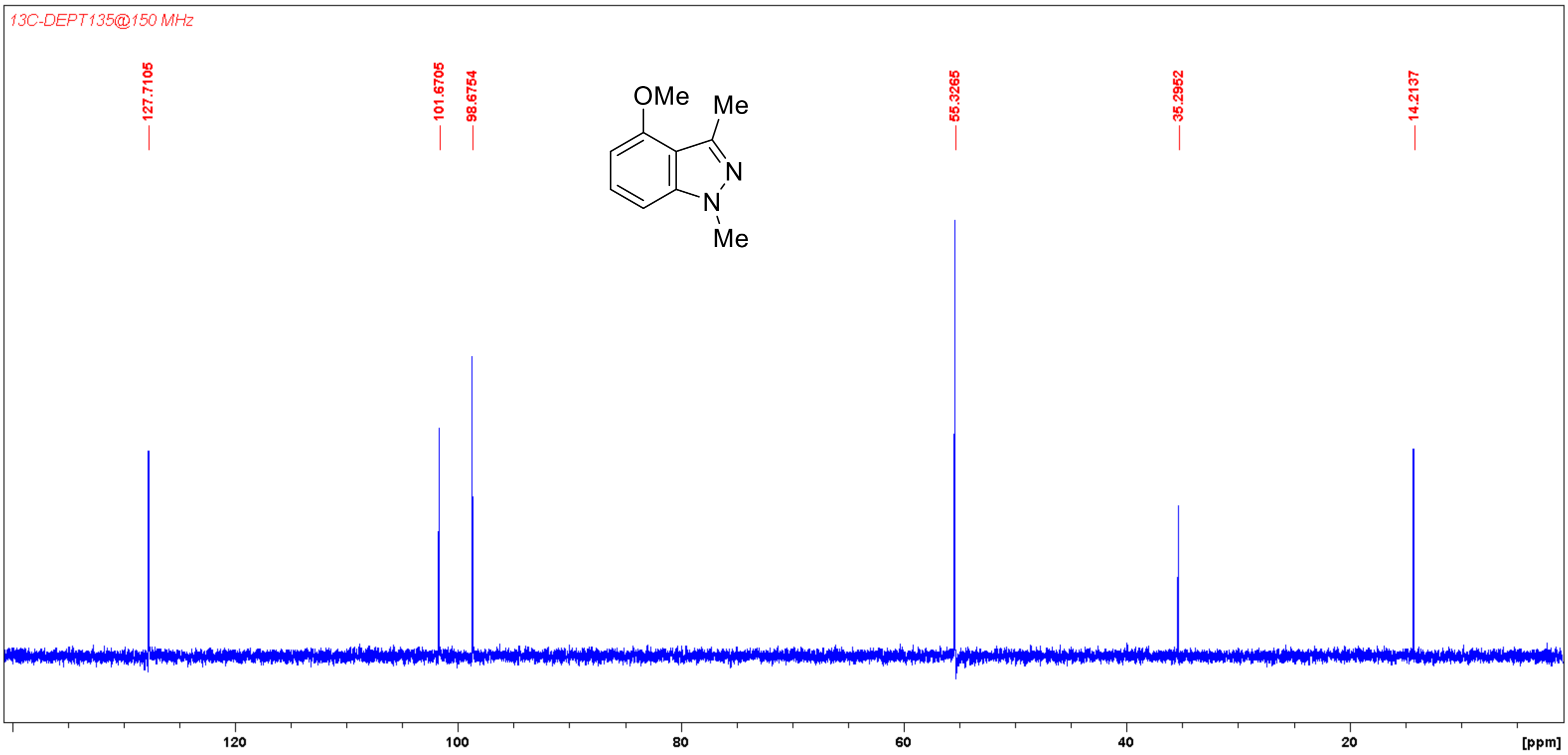

H,H-COSY

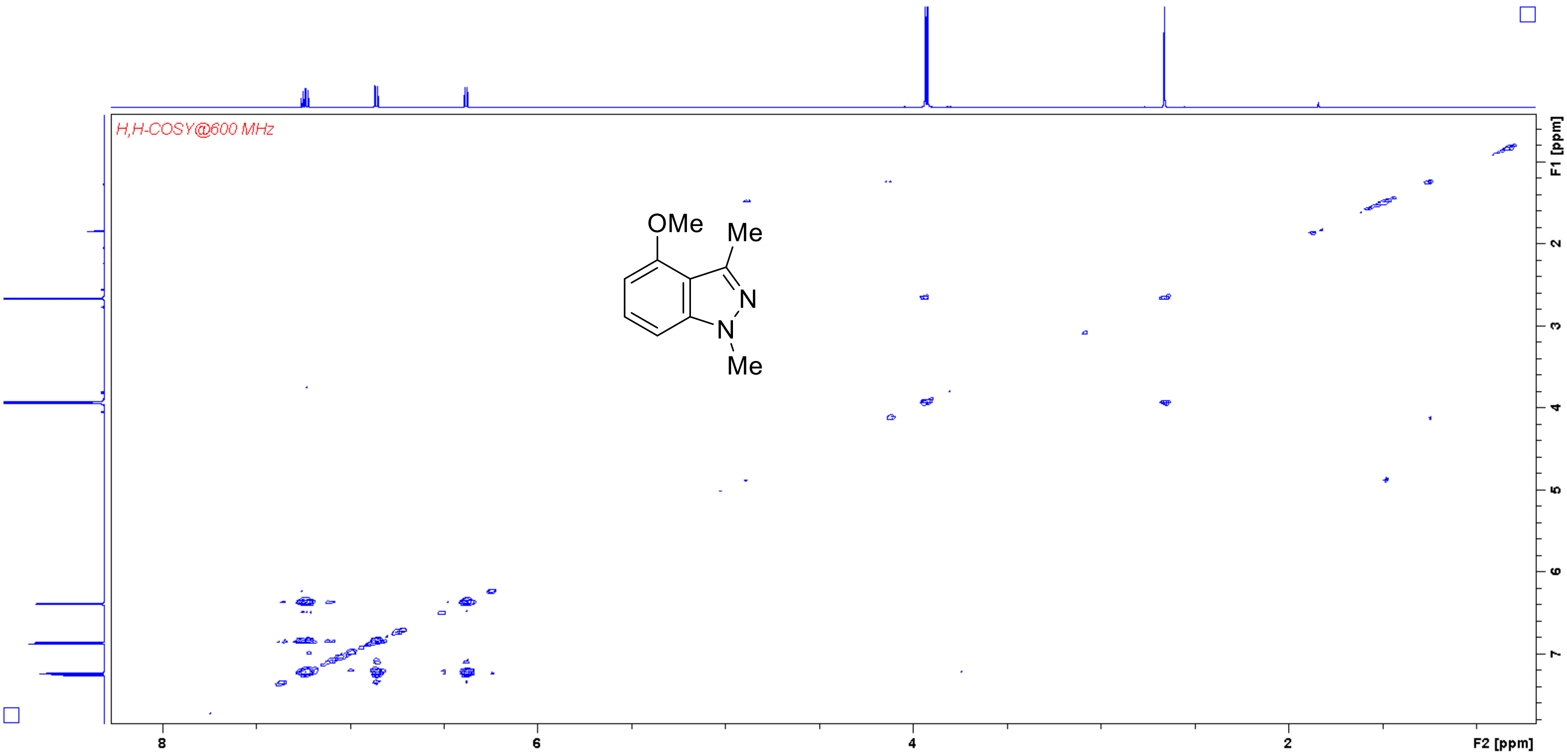

H,H-COSY@600 MHz
OMe
Me
N
N
Me
F1 [ppm]
F2 [ppm]
8
6
4
2
1
2
3
4
5
6
7

HSQC

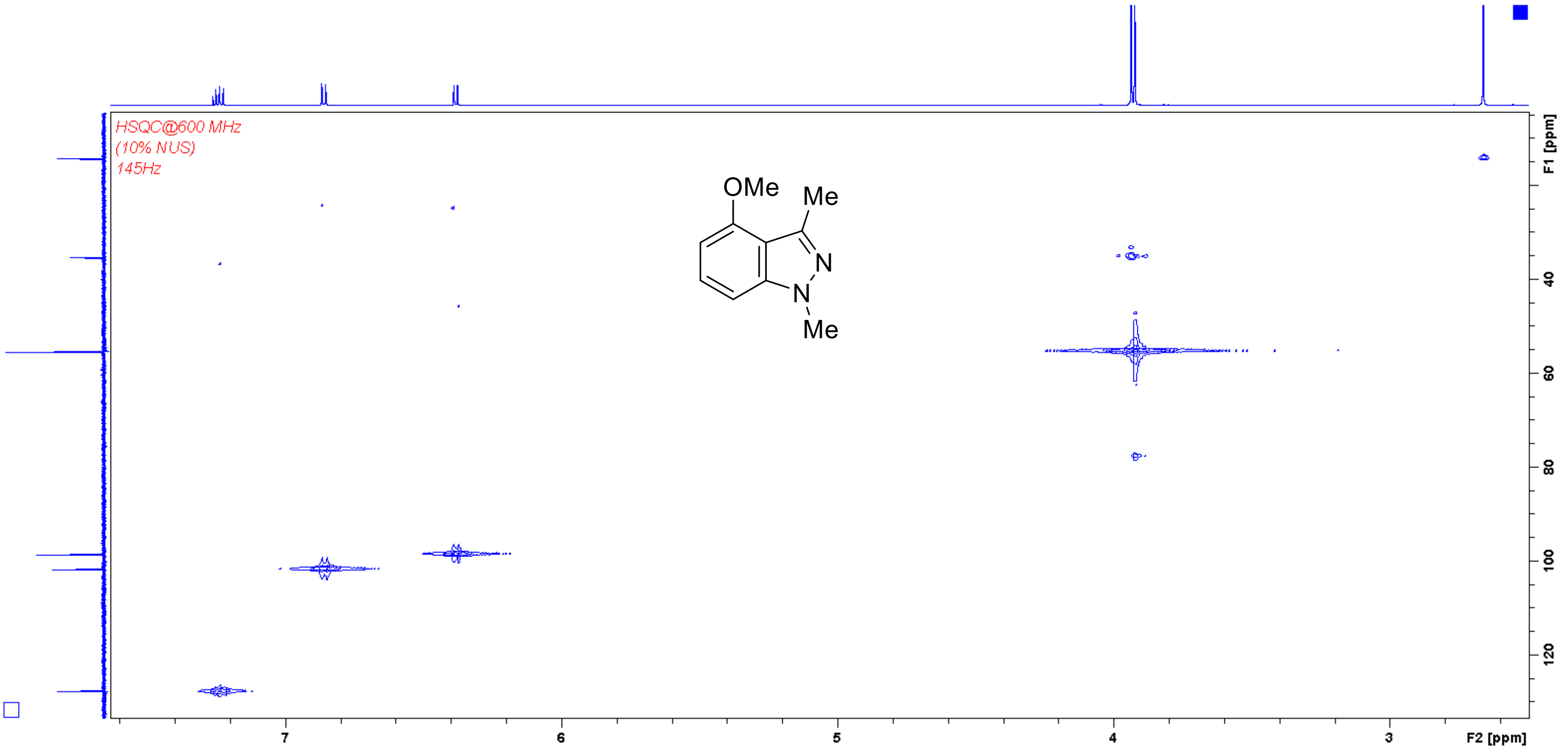

HSQC@600 MHz
(10% NUS)
145Hz
OMe
Me
N
N
Me
F1 [ppm]
F2 [ppm]
7
6
5
4
3
40
60
80
100
120

HMBC

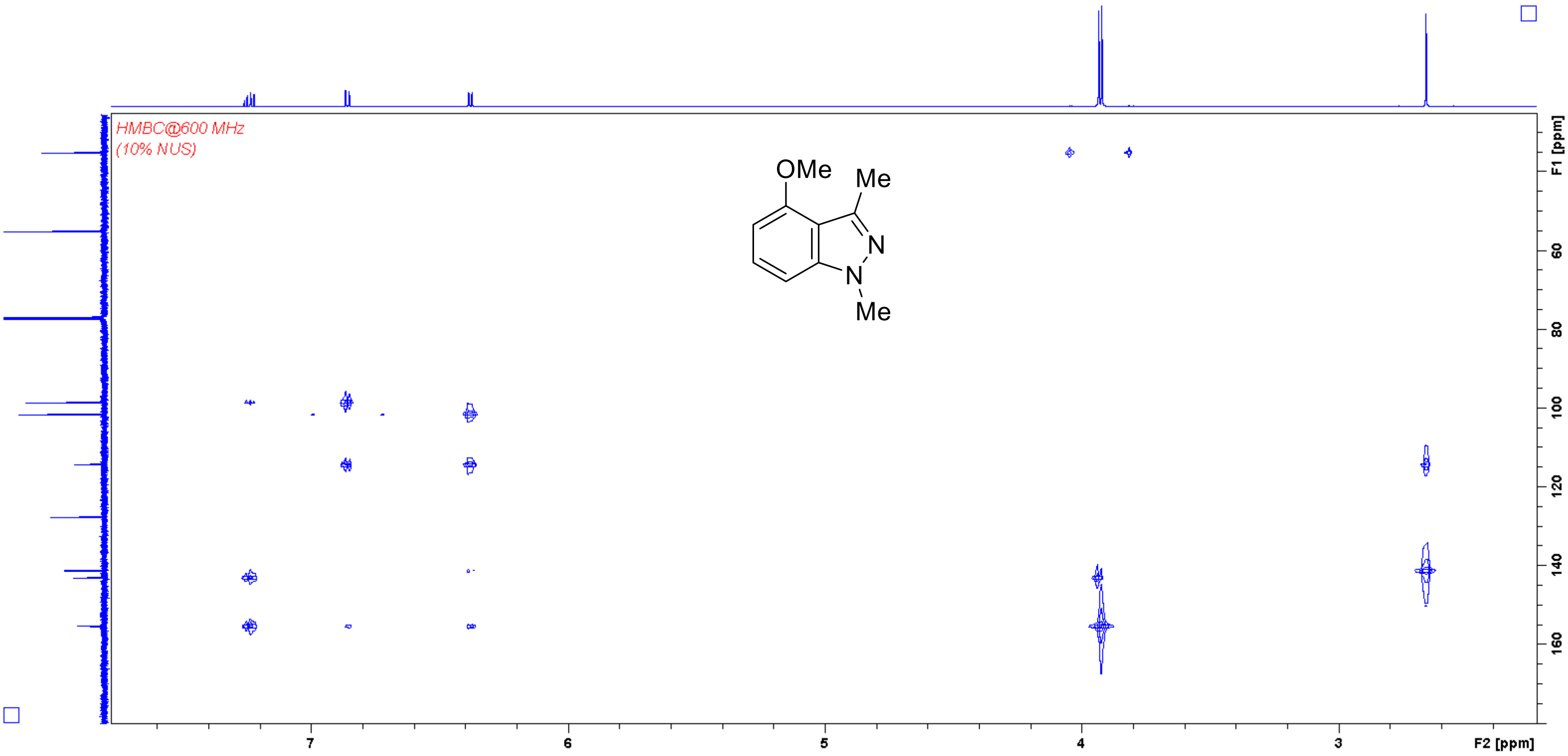
HMBC@600 MHz
(10% NUS)
OMe
Me
N
N
Me
F1 [ppm]
60
80
100
120
140
160
7
6
5
4
3
F2 [ppm]

Synthesis of 1,2,3-trimethyl-4-methoxyl-1*H*-indazolium iodide **1d**

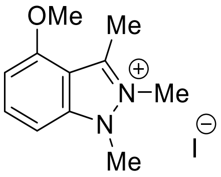

Followed by procedure B, **1d** was synthesized from 1,3-dimethyl-4-methoxyl-1*H*-indazole (407 mg, 2.31 mmol) in 10 mL acetonitrile as white solid (561 mg, 76 %).

^{1}H-NMR (DMSO-d$_6$, 600 MHz): 7.73 (t, J = 8.6 Hz, 1H, Ar-H), 7.44 (d, J = 8.6 Hz, 1H, Ar-H), 6.85 (d, J = 8.6 Hz, 1H, Ar-H), 4.19 (s, 3H, N-Me), 4.17 (s, 3H, N-Me), 3.99 (s, 3H, OMe), 2.89 (s, 3H, Me) ppm.

^{13}C{^{1}H}-NMR (DMSO-d$_6$, 150 MHz): 155.1 (o, Ar-C), 141.4 (o, Ar-C), 140.6 (o, Ar-C), 134.5 (+, Ar-C), 110.7 (o, Ar-C), 102.6 (+, Ar-C), 56.3 (+, OMe), 34.2 (+, N-Me), 33.5 (+, N-Me), 12.1 (+, Me) ppm.

ESI-MS (m/z): calculated for $[C_{11}H_{15}N_2O]^+$: 191.1184, found 191.1181.

IR (ATR): $\tilde{\nu}$ = 1275 (indazole ring), 572 (C-O-C deformation) cm^{-1}.

Melting point: 237 °C.

^{1}H-NMR

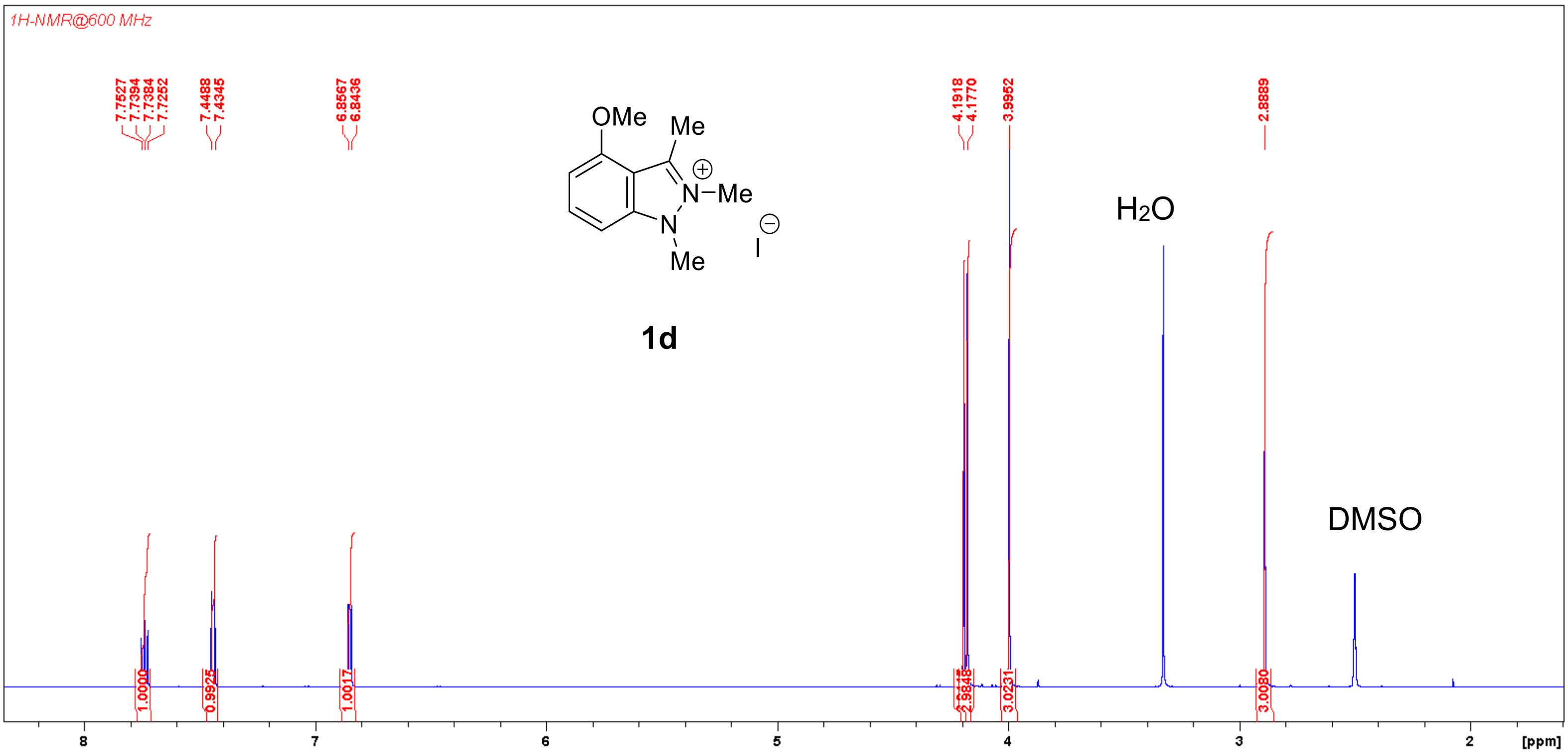
1H-NMR@600 MHz
7.7527
7.7394
7.7384
7.7252
7.4488
7.4345
6.8567
6.8436
4.1918
4.1770
3.9952
2.8889
OMe
Me
N
N–Me
Me
I
1d
H2O
DMSO
1.0000
0.9925
1.0017
3.0231
3.0080
2.9848
8
7
6
5
4
3
2
[ppm]

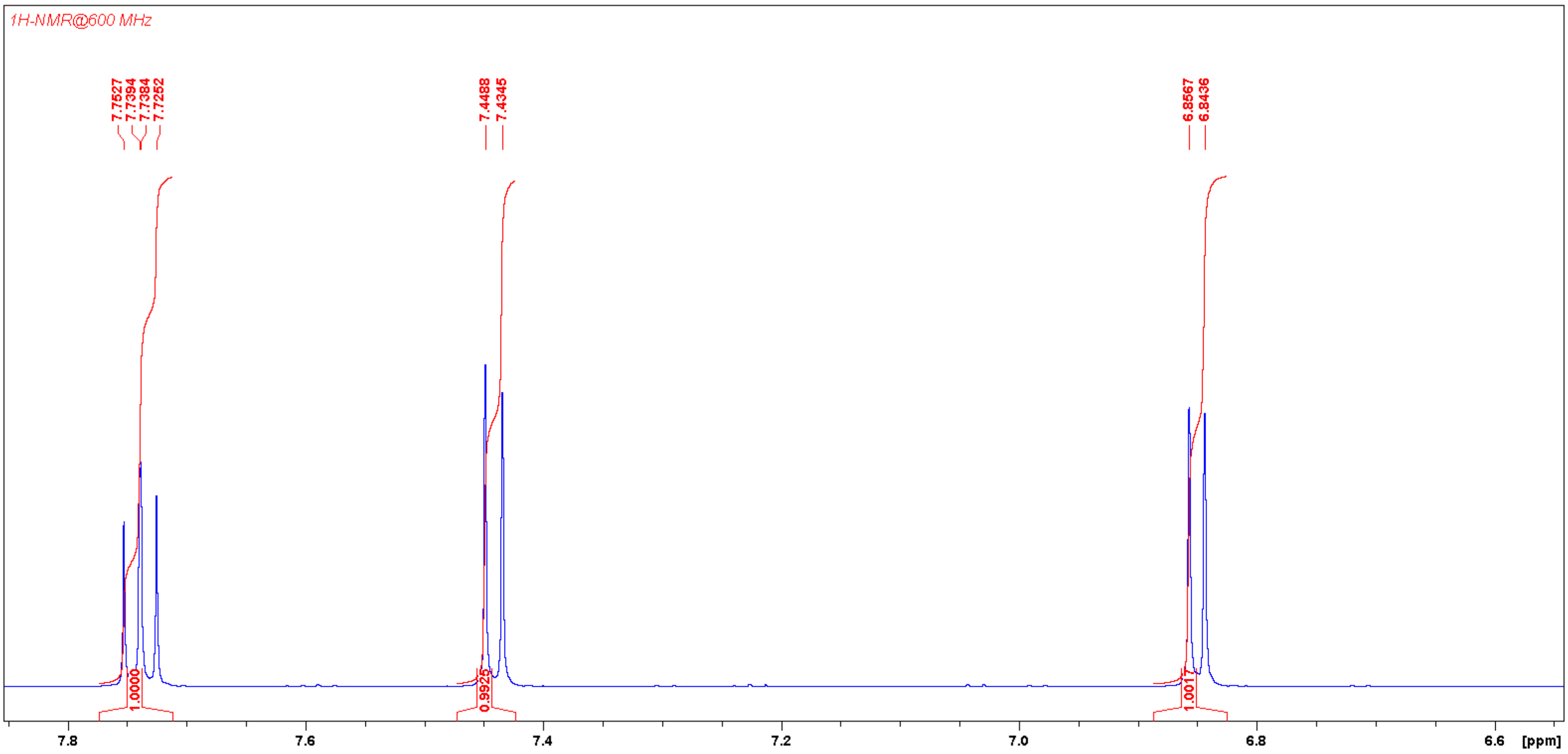
1H-NMR@600 MHz
7.7527
7.7394
7.7384
7.7252
7.4488
7.4345
6.8567
6.8436
1.0000
0.9925
1.0017
7.8
7.6
7.4
7.2
7.0
6.8
6.6
[ppm]

$^{13}C\{^1H\}$-NMR

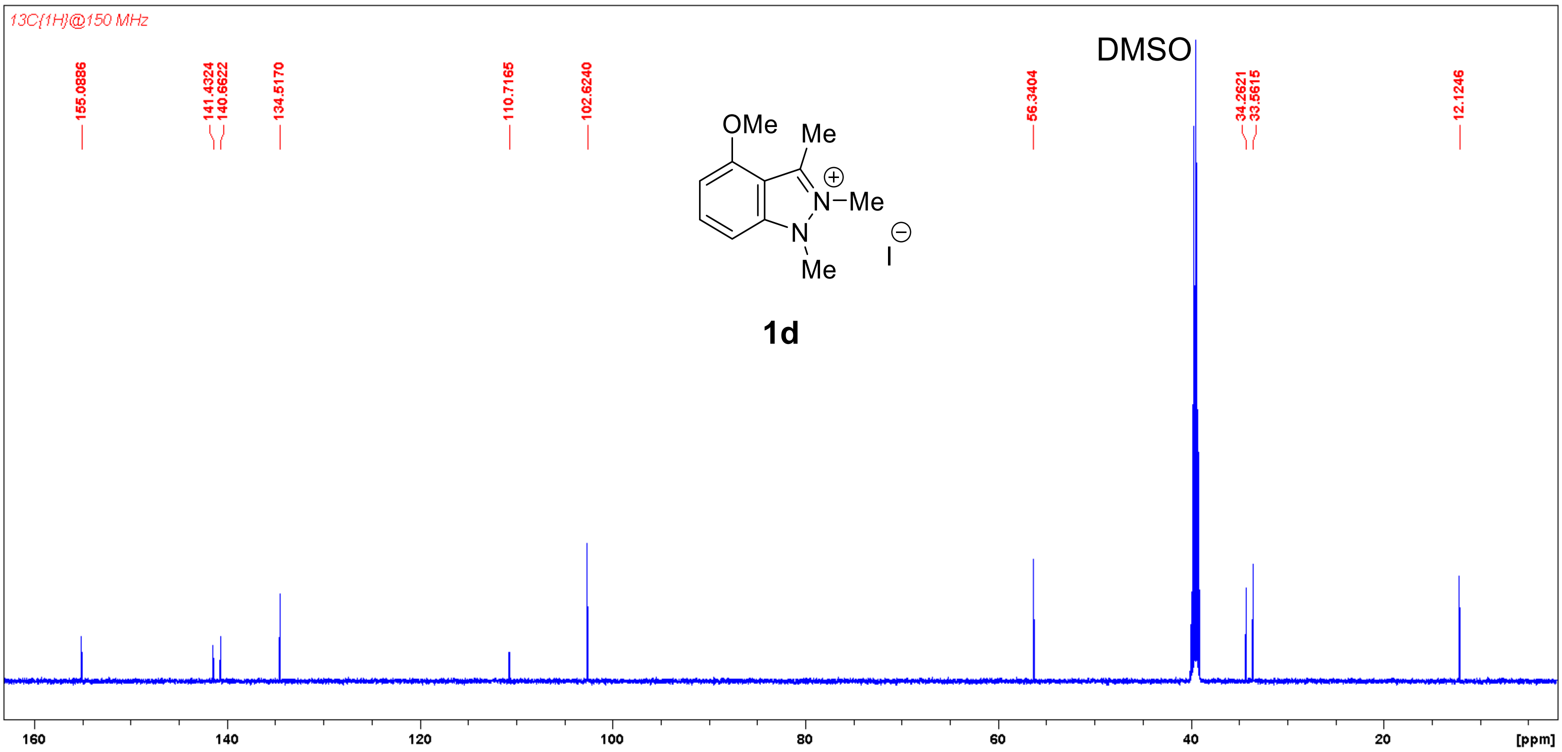

^{13}C-DEPT

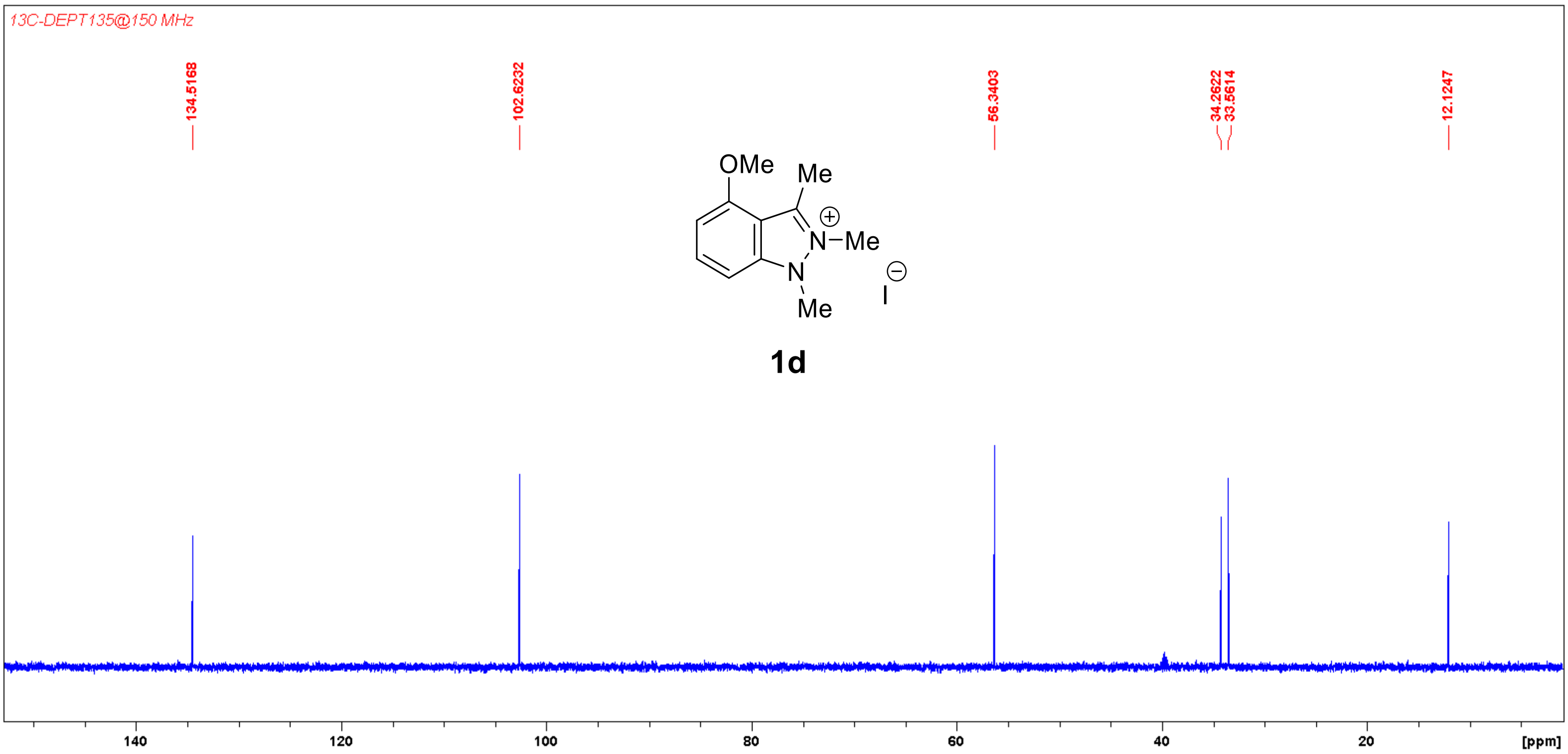

H,H-COSY

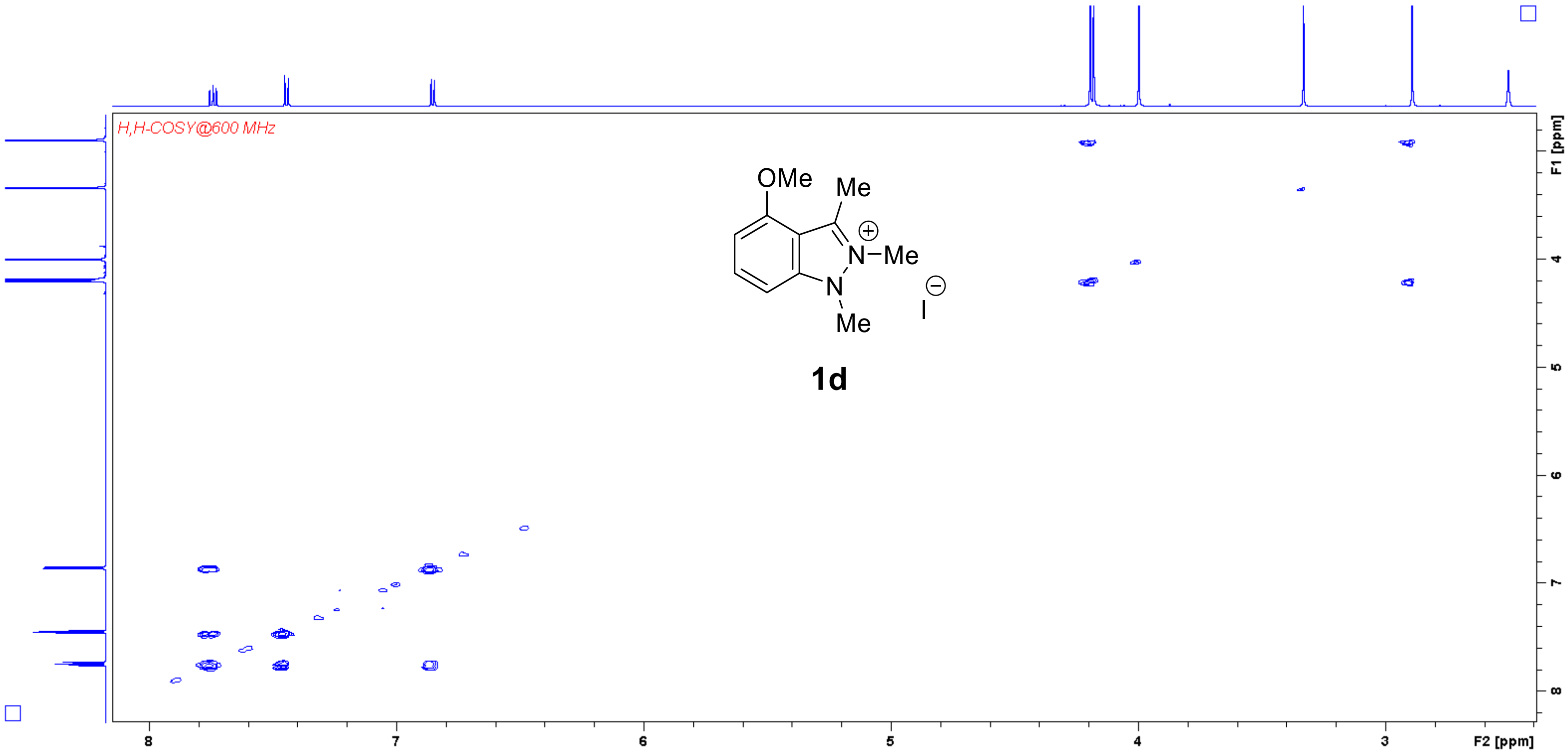
H,H-COSY@600 MHz
OMe
Me
N−Me
N
Me
I
1d
F1 [ppm]
F2 [ppm]
8
7
6
5
4
3

HSQC

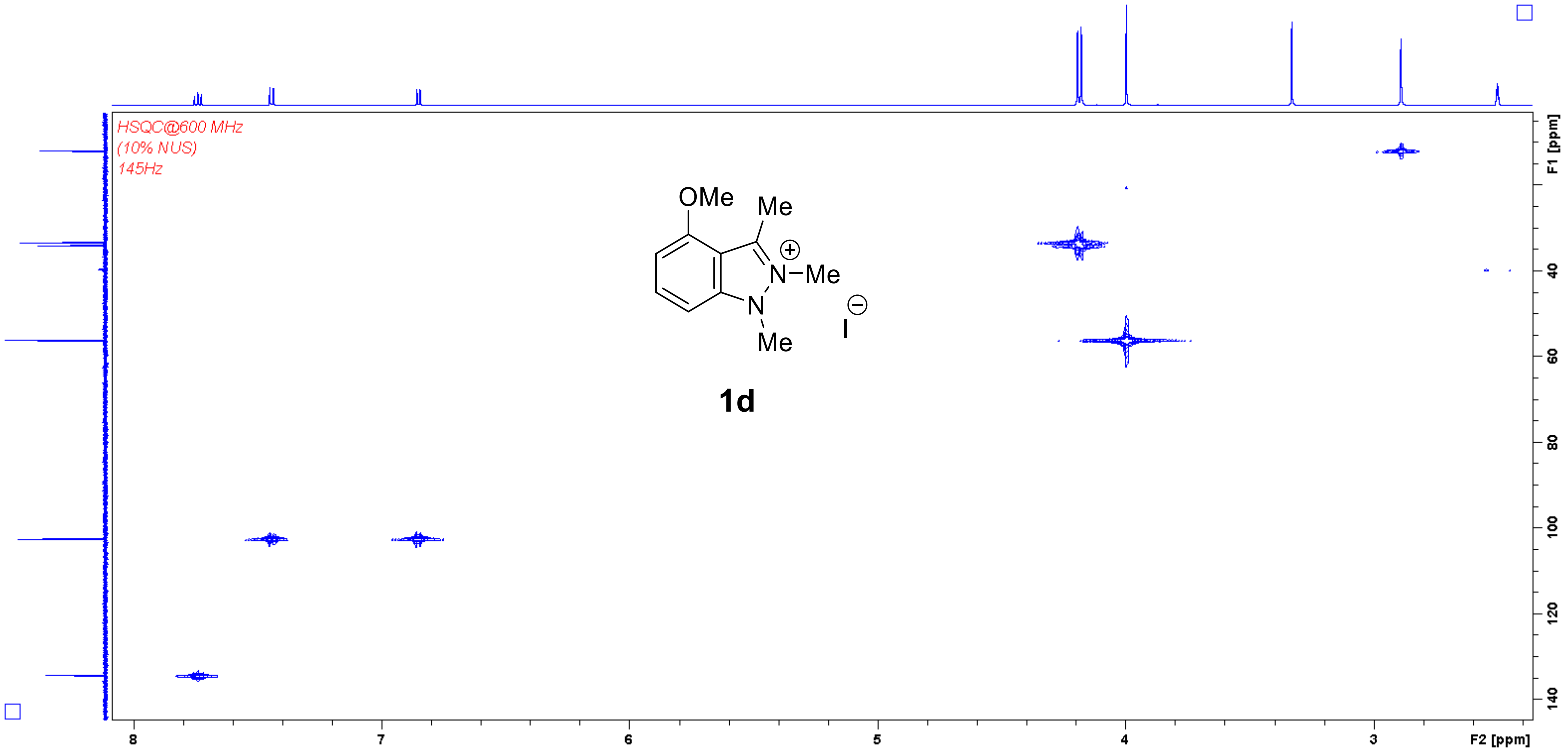
HSQC@600 MHz
(10% NUS)
145Hz
OMe
Me
N−Me
N
Me
I
1d
F1 [ppm]
F2 [ppm]
40
60
80
100
120
140
8
7
6
5
4
3

HMBC

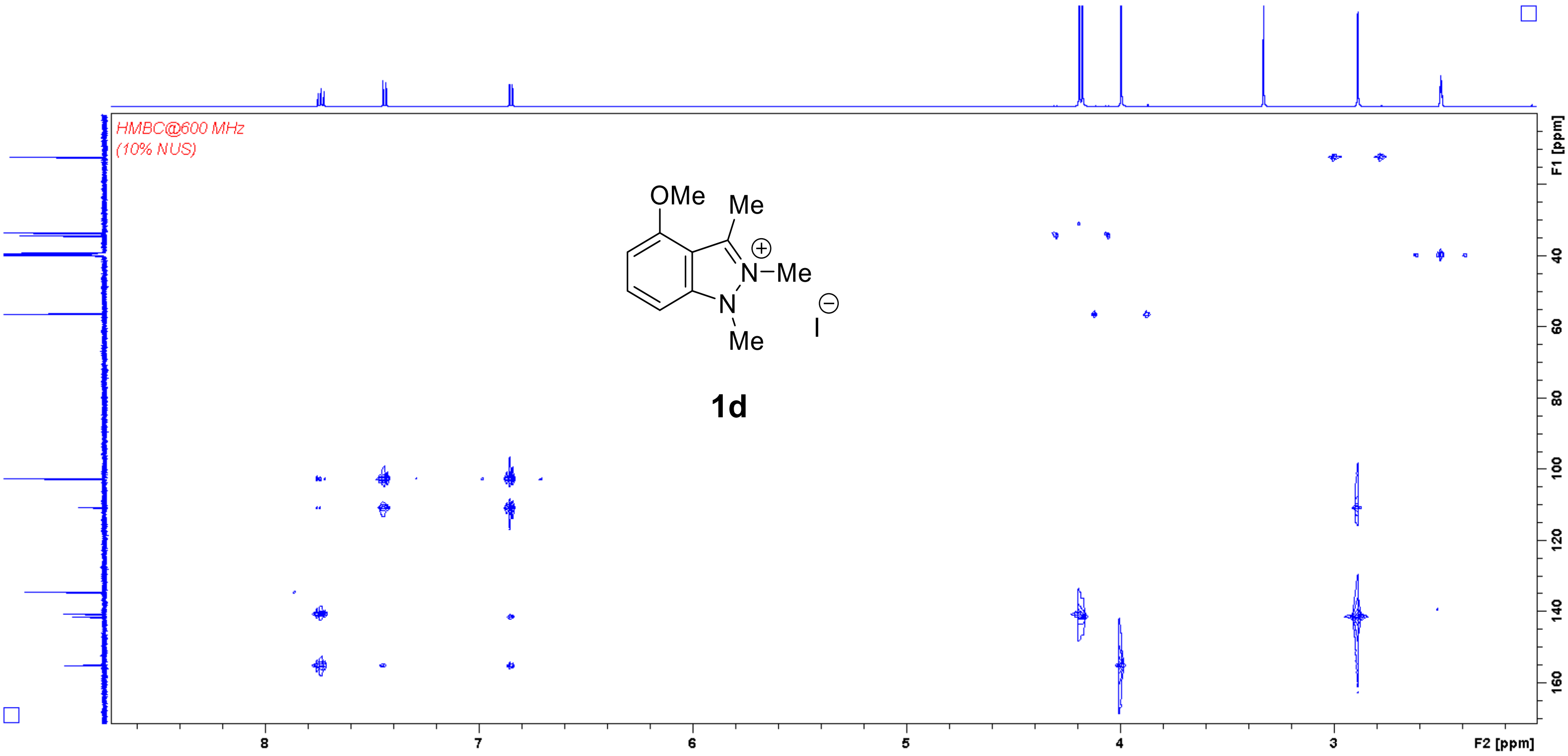

HMBC@600 MHz
(10% NUS)
OMe
Me
N−Me
N
Me
I
1d
F1 [ppm]
F2 [ppm]
8
7
6
5
4
3
40
60
80
100
120
140
160

Synthesis of 1,3,4,5-tetramethyl-1*H*-pyrazole

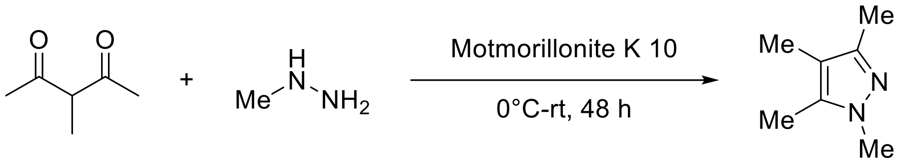

This synthesis is made according to a modified literature procedure: Texier-Boullet, F.; Klein, B.; Hamelin, J., Pyrrole and Pyrazole Ring Closure in Heterogeneous Media. *Synthesis*, **1986**, 5, 409-411. DOI: 10.1055/s-1986-31655.

Under ice bath cooling, 3-methylpentane-2,4-dione (1.180 g, 1 Eq. 10.34 mmol) was added to Montmorillonite K 10 (3 g) by thorough mixing. Methylhydrazine (476 mg, 1 Eq, 10.34 mmol)) was added dropwise at 0°C. After the mixture is well mixed, the mixture was allowed to stand at room temperature for 48 h. DCM was then added and Montmorillonite was filtered off. Evaporation of the solvent gives the desired product as light yellow solid (1.123 g, 90%).

^{1}H-NMR (400 MHz, $CDCl_3$): δ = 3.66 (s, 3H), 2.12 (s, 3H), 2.10 (s, 3H), 1.86 (s, 3H) ppm.

The spectroscopic data is in accordance with the literature: Thompson, C. M.; Poole, J. L.; Cross, J. L.; Akritopoulou-Zanze, I.; Djuric, S. W., Small Molecule Library Synthesis Using Segmented Flow, *Molecules* **2011**, 16(11), 9161–9177. DOI: 10.3390/molecules16119161.

^{1}H-NMR

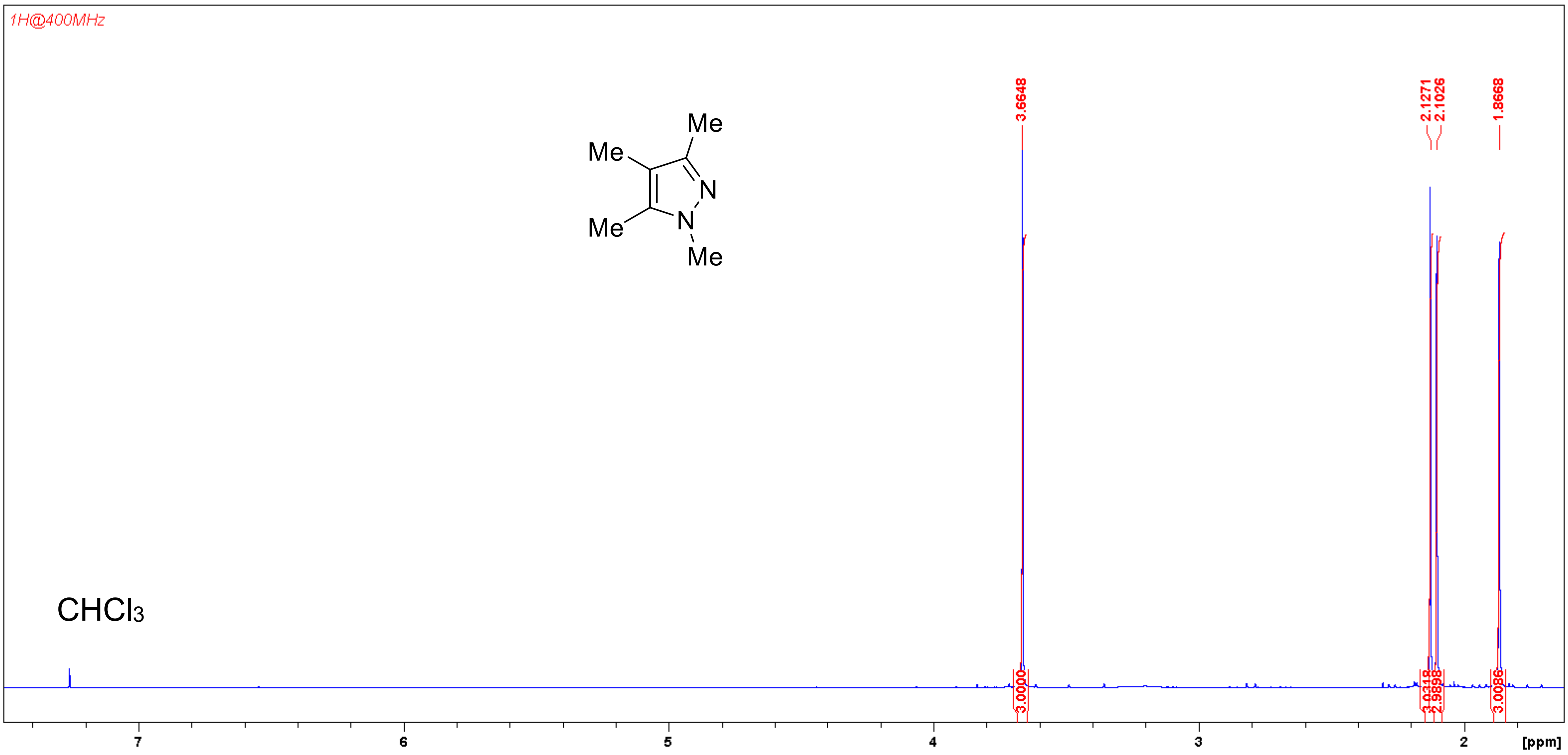
1H@400MHz
3.6648
2.1271
2.1026
1.8668
CHCl3
Me
Me
N
N
Me
Me
3.0000
3.0318
2.9898
3.0086
7
6
5
4
3
2
[ppm]

Synthesis of 1,2,3,4,5-pentamethyl-1*H*-pyrazolium iodide **3a**

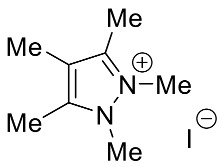

Followed by procedure B, **3a** is afforded from 1,3,4,5-tetramethyl-1H-pyrazole (1.123 g, 9.04 mmol) in 10 mL acetonitrile as light-yellow product (2.181 g, 90%).

^{1}H-NMR (400 MHz, DMSO-d$_6$): δ = 3.91 (s, 6H, 1,2-H), 2.35 (s, 6H, 3,5-H), 2.00 (s, 3H, 4-H).

The spectroscopic data is in accordance with the literature. Leigh, W. J.; Arnold, D. R., Photochemical and thermal rearrangements of some 3*H*-pyrazoles, *Can. J. Chem.* **1979**, *57*, 1186–1200. DOI: 10.1139/v79-194.

^{1}H-NMR

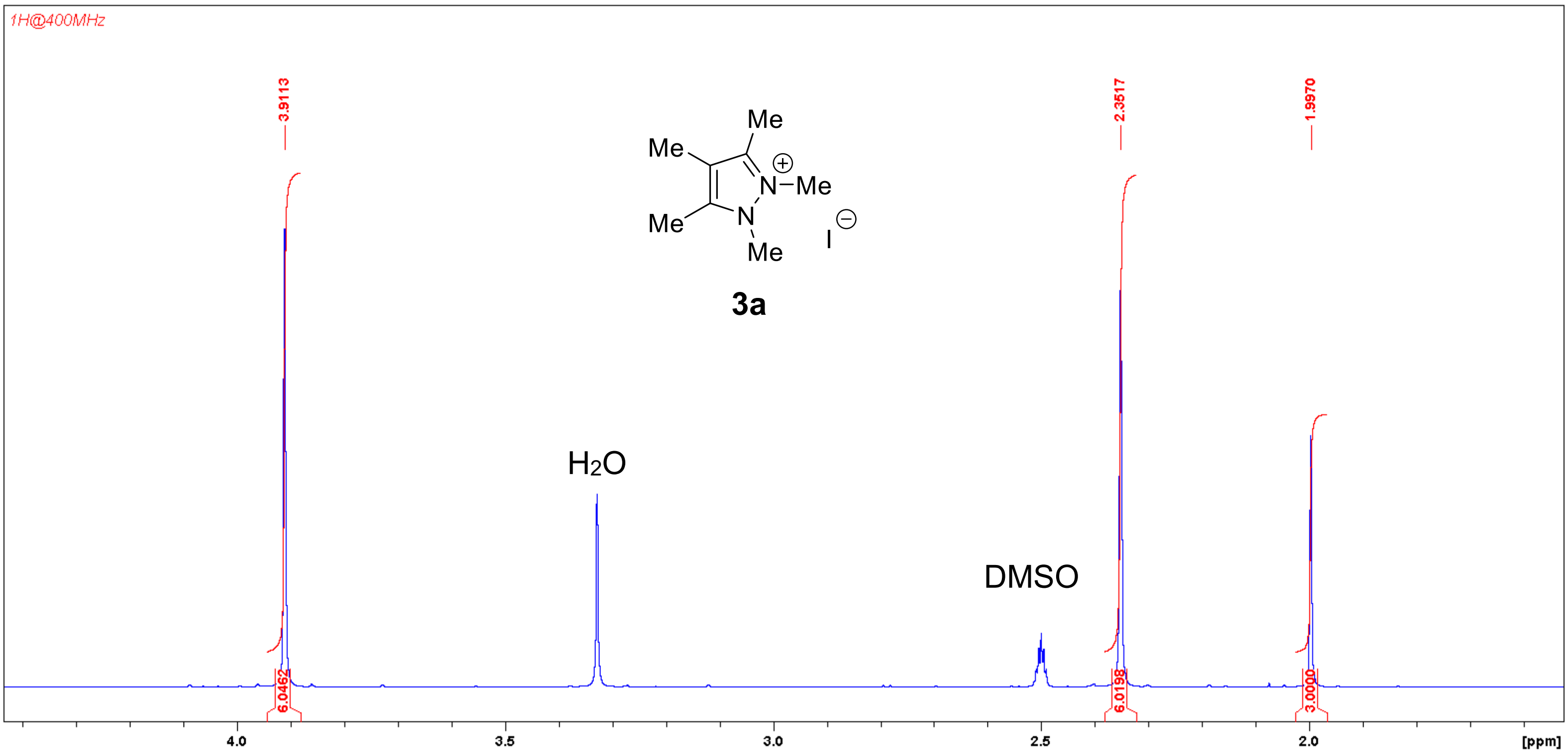

Synthesis of 3,4-dimethyl-1,5-diphenyl-1*H*-pyrazole

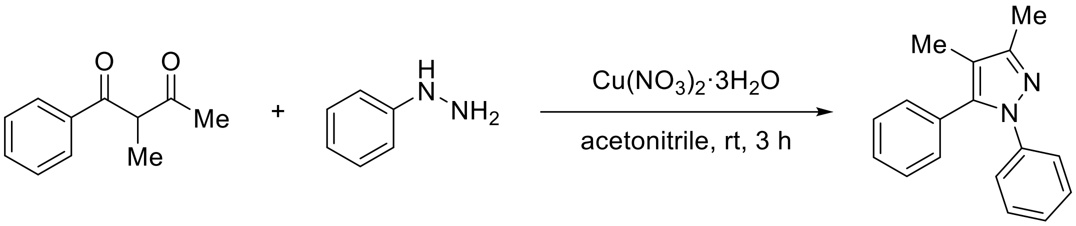

Synthesis according to modified literature procedures: Wang. H.; Sun, X.; Zhang, S.; Liu, G.; Wang, C.; Zhu, L.; Zhang, H., Efficient Copper-Catalyzed Synthesis of Substituted Pyrazoles at Room Temperature. *Synlett.* **2018**, 29(20), 2689-2692. DOI: 10.1055/s-0037-1610330

2-Methyl-1-phenylbutane-1,3-dione (1.2 Eq, 773 mg, 4.39 mmol) and phenyl hydrazine (1.0 Eq, 395 mg, 3.66 mmol) were added and dissolved in acetonitrile (10 mL). After the mixture was cooled to 0 °C, copper(II)nitrate trihydrate (0.1 Eq, 88 mg, 0.366 mmol) was added. The solution was then stirred at room temperature for 3 h. The solvent was evaporated *in vacuo* and the residue dissolved in DCM. The organic layer was washed with water three times and dried with anhydrous Na_2SO_4. The solvent was evaporated *in vacuo* and the crude product was purified by column chromatography on silica gel and afforded as orange solid (728 mg, 80%).

^{1}H-NMR (400 MHz, $CDCl_3$): δ = 7.36-7.30 (m, 3H), 7.27-7.21 (m, 2H), 7.21-7.14 (m, 5H), 2.34 (s, 3H), 2.04 (s, 3H) ppm.

The spectra are in accordance to the literature: Genin, M. J.; Biles, C.; Keiser, B. J.; Poppe, S. M.; Swaney, S. M.; Tarpley, W. G.; Yagi, Y.; Romero, D. L., Novel 1,5-Diphenylpyrazole Nonnucleoside HIV-1 Reverse Transcriptase Inhibitors with Enhanced Activity versus the Delavirdine-Resistant P236L Mutant: Lead Identification and SAR of 3- and 4-Substituted Derivatives. *J. Med. Chem.* **2000**, *43*, 1034–1040. DOI: 10.1021/jm990383f.

^{1}H-NMR

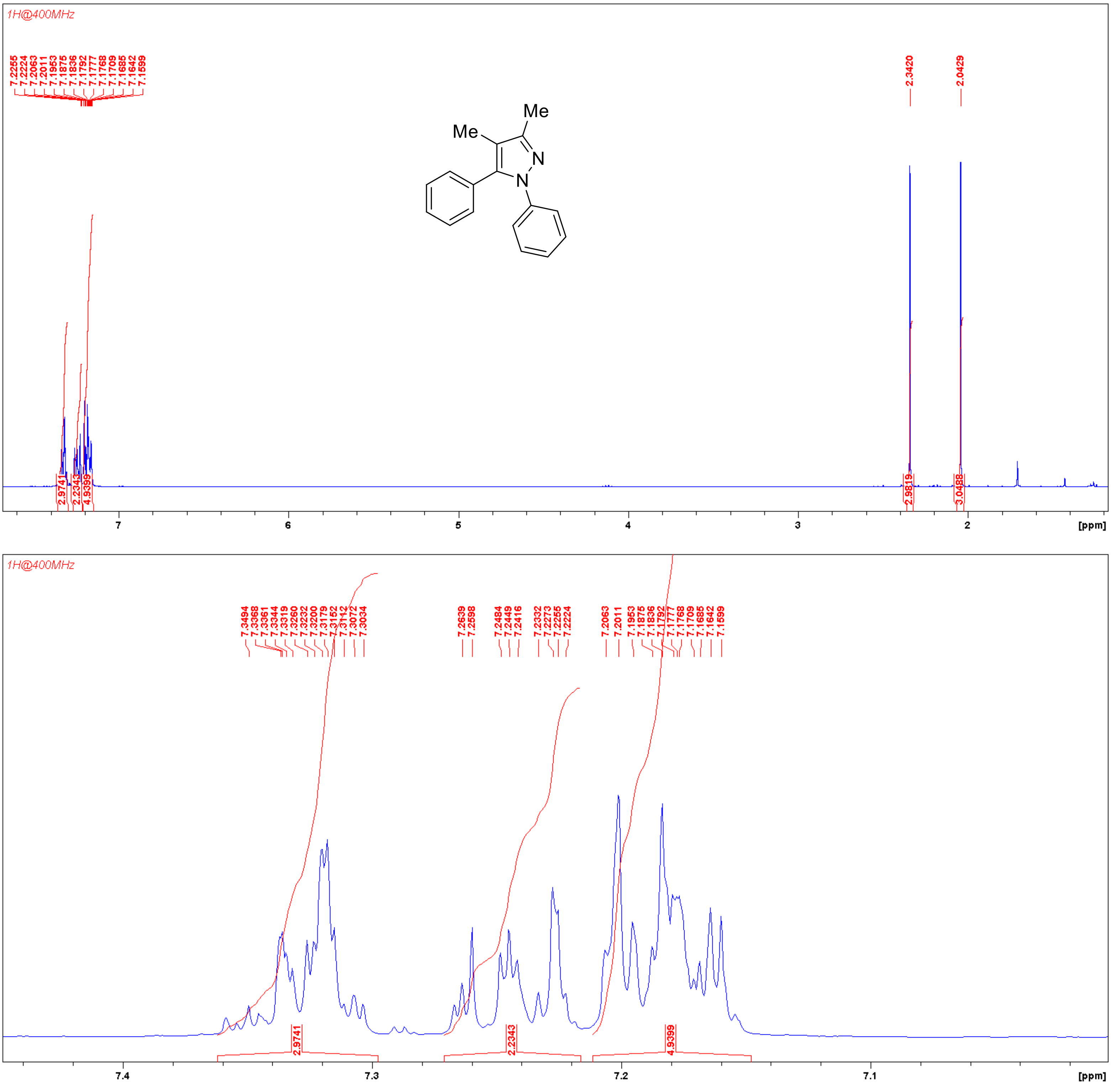
1H@400MHz
7.2255
7.2224
7.2063
7.2011
7.1953
7.1875
7.1836
7.1792
7.1777
7.1768
7.1709
7.1685
7.1642
7.1599
2.3420
2.0429
Me
Me
N
N
2.9741
2.2343
4.9399
2.9819
3.0488
[ppm]
1H@400MHz
7.3494
7.3368
7.3361
7.3344
7.3319
7.3260
7.3232
7.3200
7.3179
7.3152
7.3112
7.3072
7.3034
7.2639
7.2598
7.2484
7.2449
7.2416
7.2332
7.2273
7.2255
7.2224
7.2063
7.2011
7.1953
7.1875
7.1836
7.1792
7.1777
7.1768
7.1709
7.1685
7.1642
7.1599
2.9741
2.2343
4.9399
[ppm]

Synthesis of 1,5-Diphenyl-2,3,4-trimethyl-1*H*-pyrazolium iodide **3b**

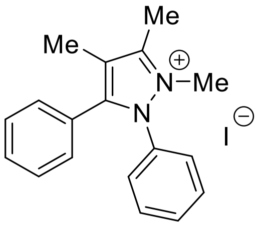

Followed by procedure B, **3b** was afforded from 3,4-dimethyl-1,5-diphenyl-1*H*-pyrazole (728 mg, 2.93 mmol) in 10 mL acetonitrileas yellow solid (766 mg, 67%).

^{1}H-NMR (600 MHz, DMSO-d$_6$): δ = 7.64-7.60 (m, 3H, Ar-H), 7.59-7.56 (m, 2H, Ar-H), 7.45-7.39 (m, 3H, Ar-H), 7.34-7.31 (m, 2H, Ar-H), 3.73 (s, 3H, N-Me), 2.59 (s, 3H, Me), 2.11 (s, 3H, Me) ppm.

^{13}C{^{1}H}-NMR (150 MHz, DMSO-d$_6$): δ = 146.0 (o, Ar-C), 145.9 (o, Ar-C), 132.0 (+, Ar-C), 131.7 (o, Ar-C), 130.3 (+, Ar-C), 130.1 (+, Ar-C), 129.7 (+, Ar-C), 129.6 (+, Ar-C), 128.8 (+, Ar-C), 125.6 (o, Ar-C), 115.0 (o, Ar-C), 34.9 (+, N-Me), 10.4 (+, Me), 8.0 (+, Me) ppm.

HRMS (ESI): *m/z* calculated for $[C_{18}H_{19}N_2]^+$: 263.1543, found 263.1542.

IR (ATR): $\tilde{\nu}$ = 1612 (aromatic ring) cm^{-1}.

Melting point: 202-203 °C

^{1}H-NMR

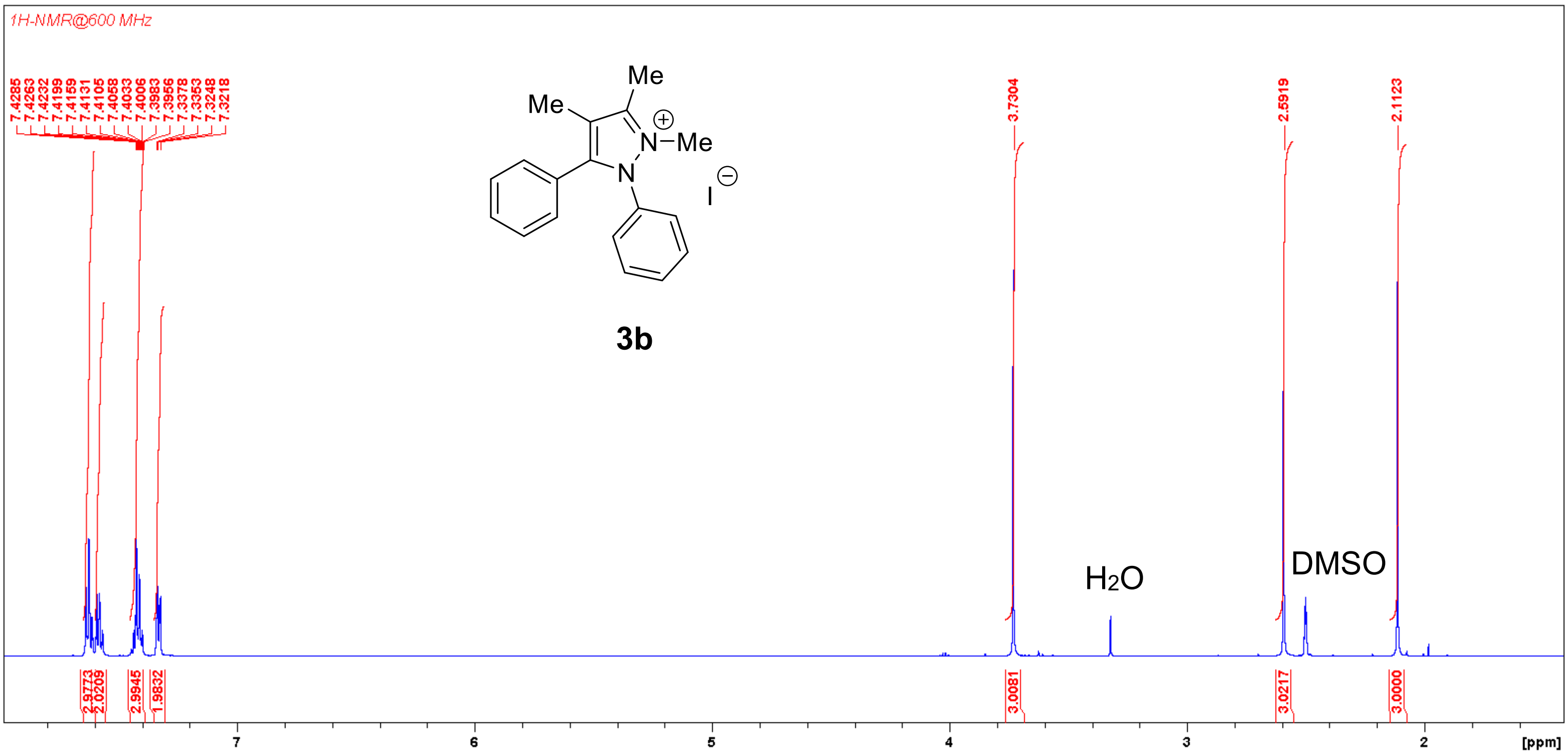
1H-NMR@600 MHz
Me
Me
N−Me
N
I
3b
H2O
DMSO
3.7304
2.5919
2.1123
2.9773
2.0209
2.9945
1.9832
3.0081
3.0217
3.0000
7
6
5
4
3
2
[ppm]

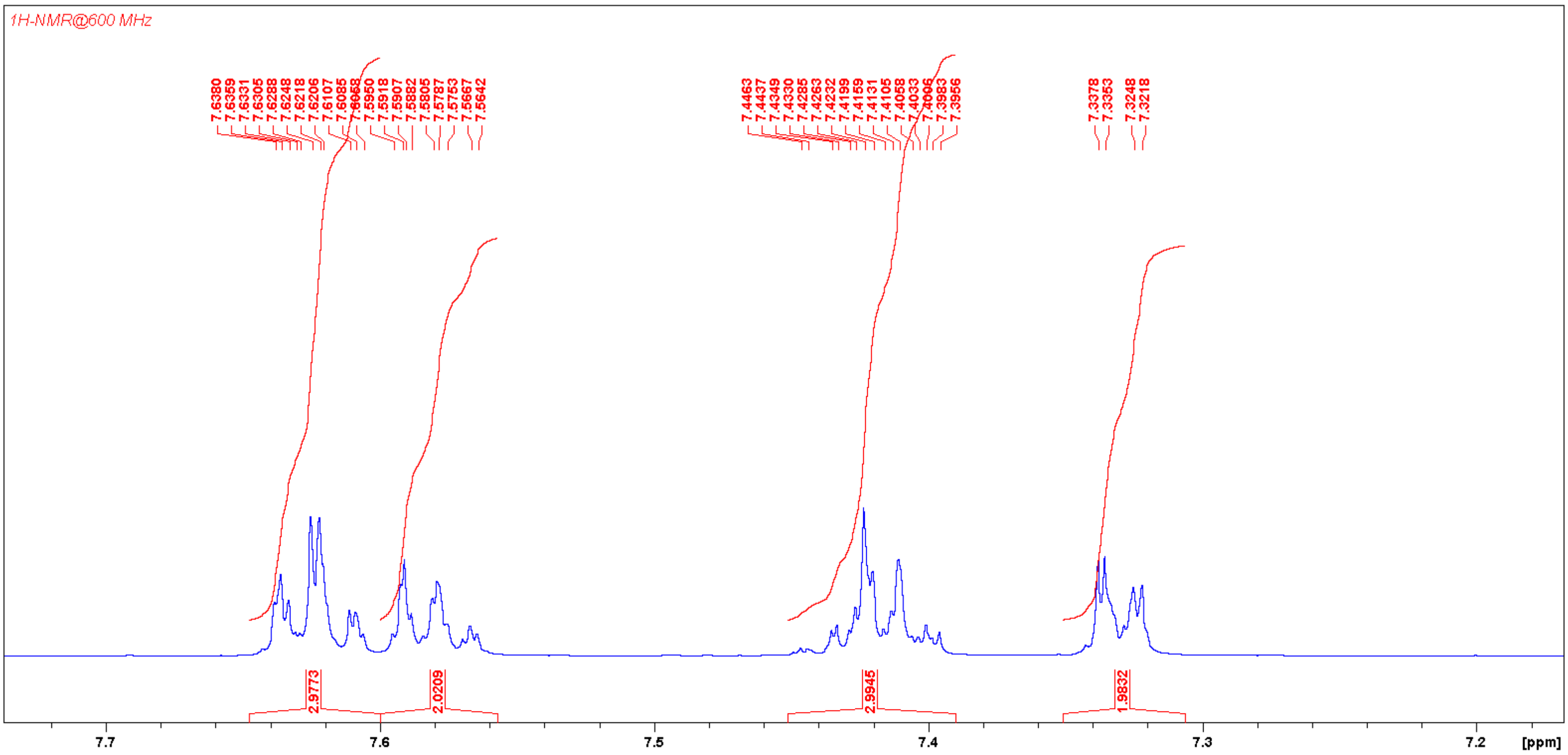
1H-NMR@600 MHz
2.9773
2.0209
2.9945
1.9832
7.7
7.6
7.5
7.4
7.3
7.2
[ppm]

$^{13}C\{^{1}H\}$-NMR

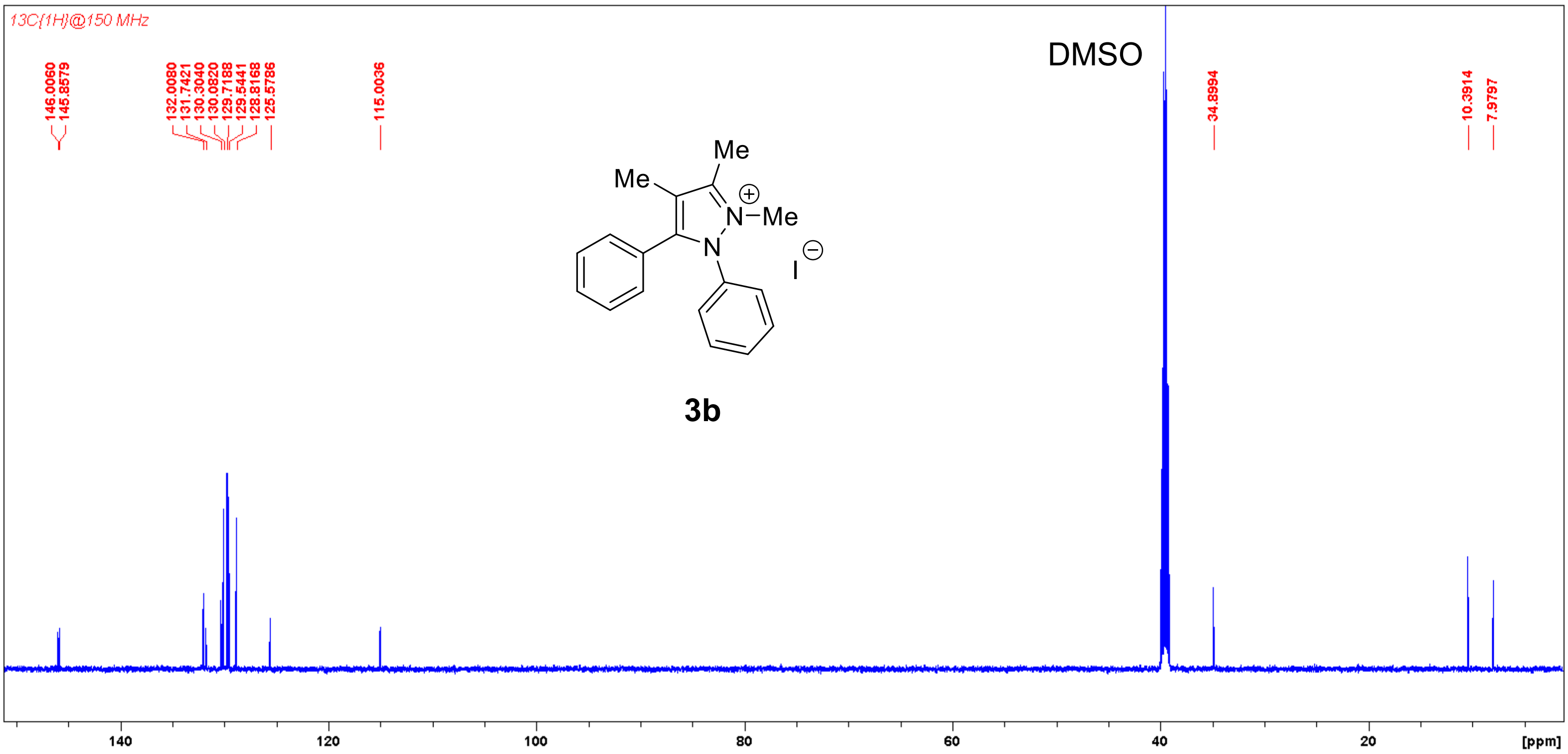
13C{1H}@150 MHz
146.0060
145.8579
132.0080
131.7421
130.3040
130.0820
129.7188
129.5441
128.8168
125.5786
115.0036
DMSO
34.8994
10.3914
7.9797
Me
Me
N−Me
N
I
3b
140
120
100
80
60
40
20
[ppm]

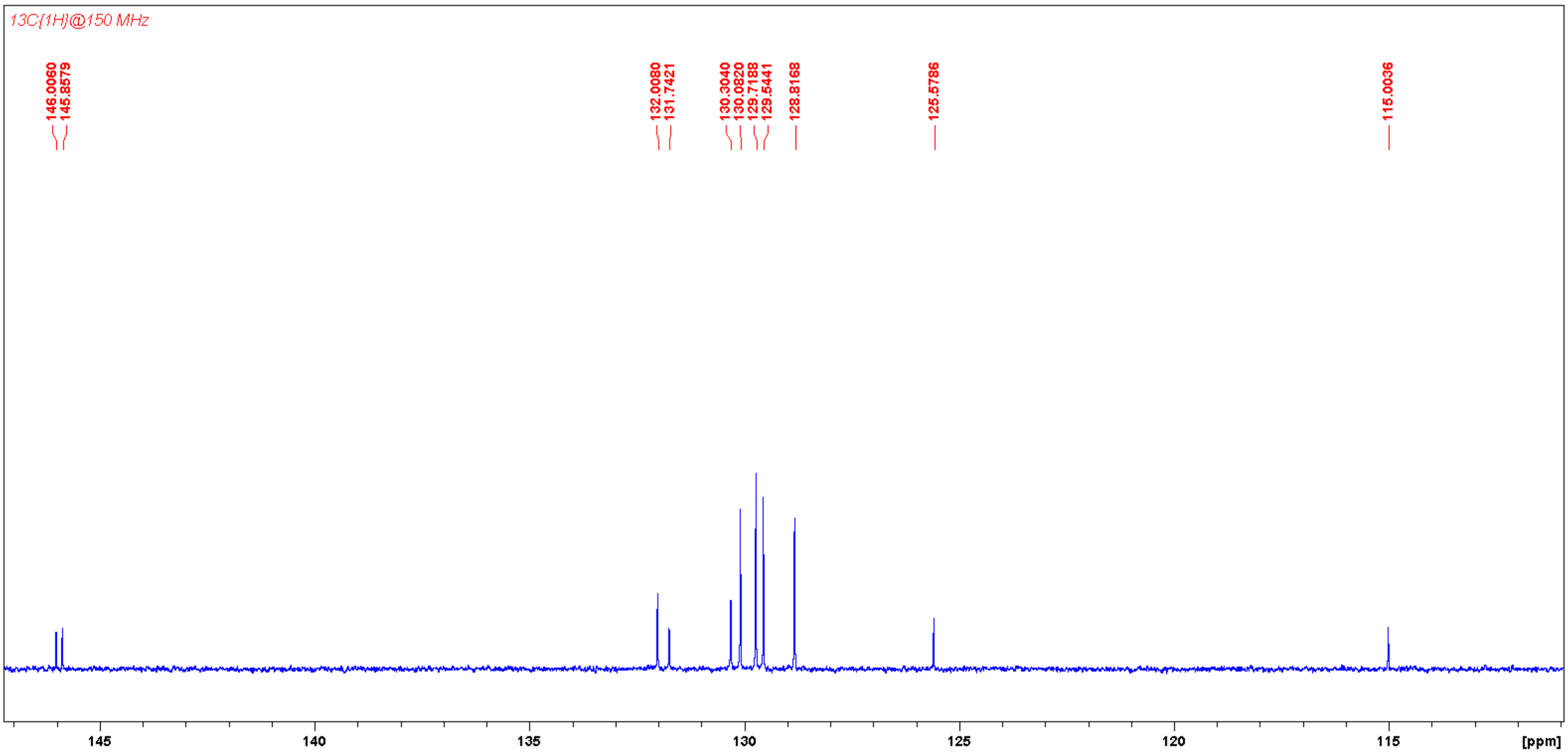
13C{1H}@150 MHz
146.0060
145.8579
132.0080
131.7421
130.3040
130.0820
129.7188
129.5441
128.8168
125.5786
115.0036
145
140
135
130
125
120
115
[ppm]

^{13}C-DEPT

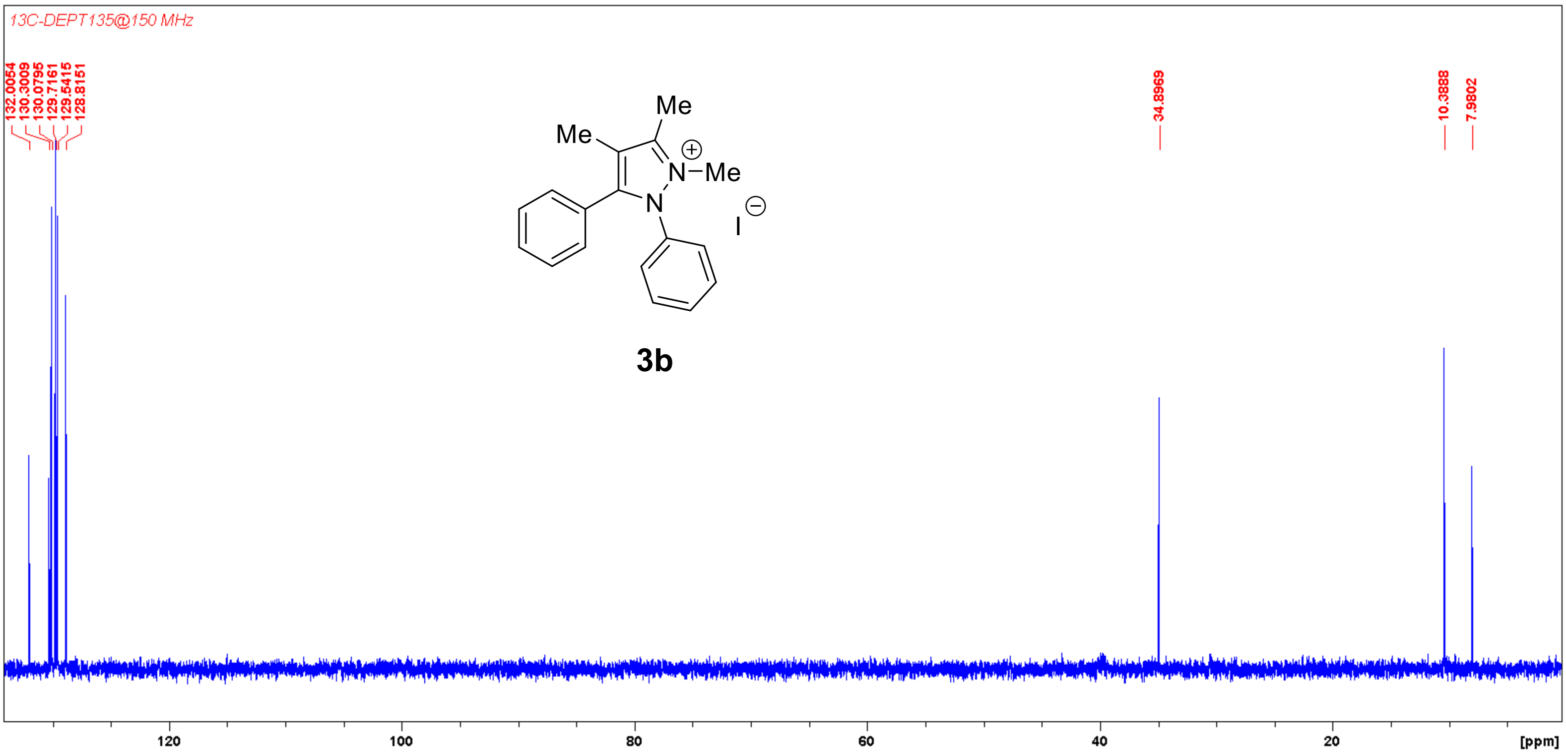

13C-DEPT135@150 MHz
132.0054
130.3009
130.0795
129.7161
129.5415
128.8151
34.8969
10.3888
7.9802
Me
Me
N−Me
N
I
3b
120
100
80
60
40
20
[ppm]

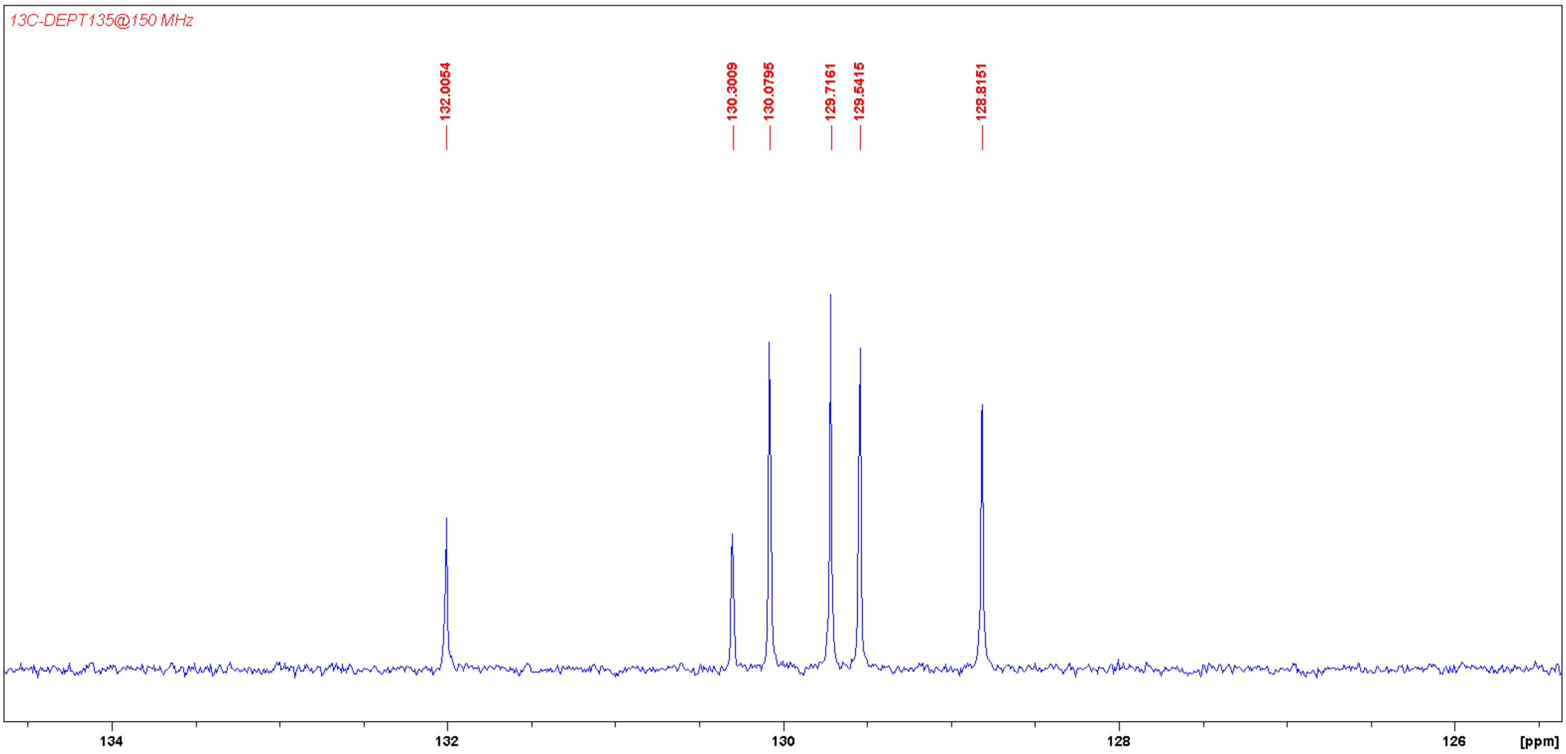

13C-DEPT135@150 MHz
132.0054
130.3009
130.0795
129.7161
129.5415
128.8151
134
132
130
128
126
[ppm]

H,H-COSY

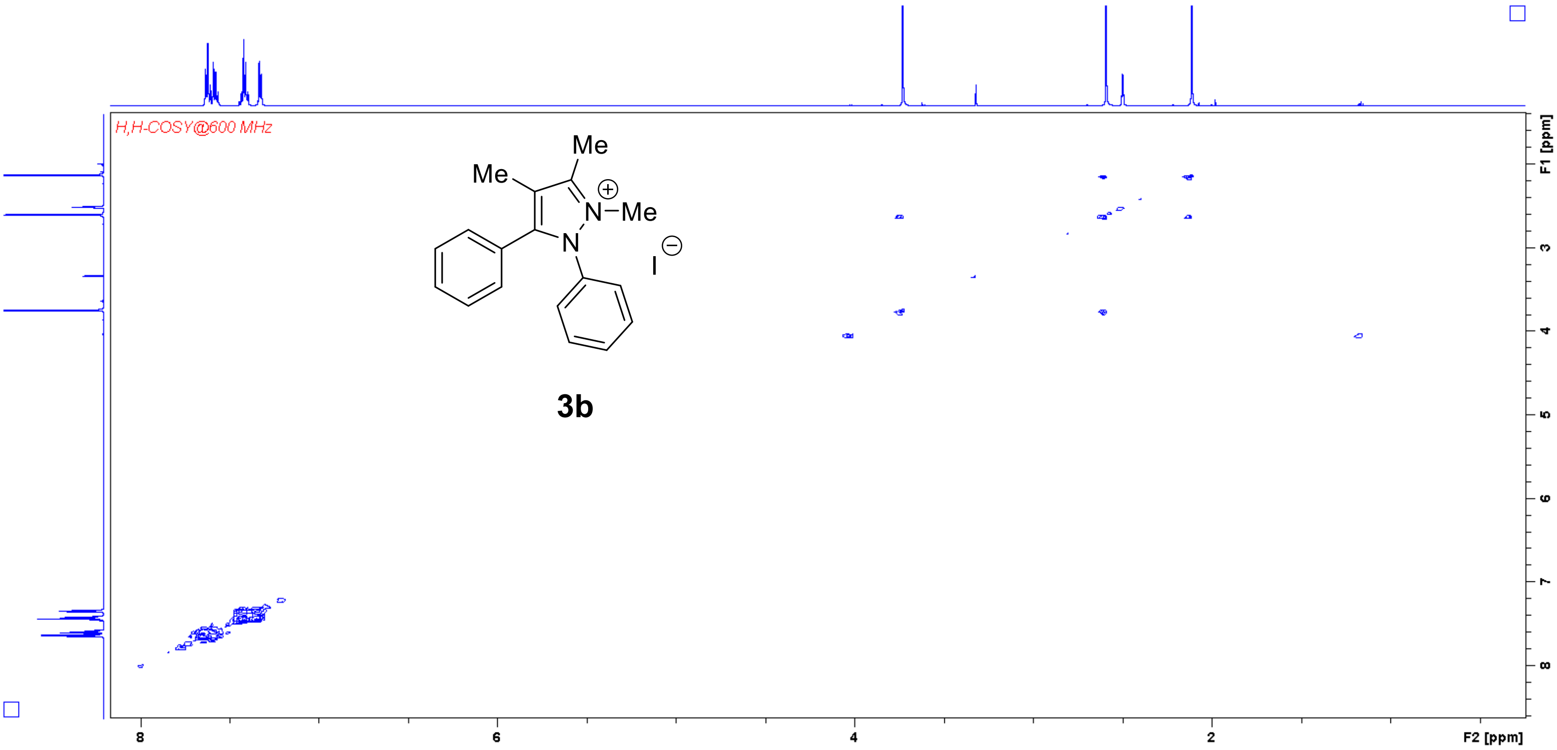

HSQC

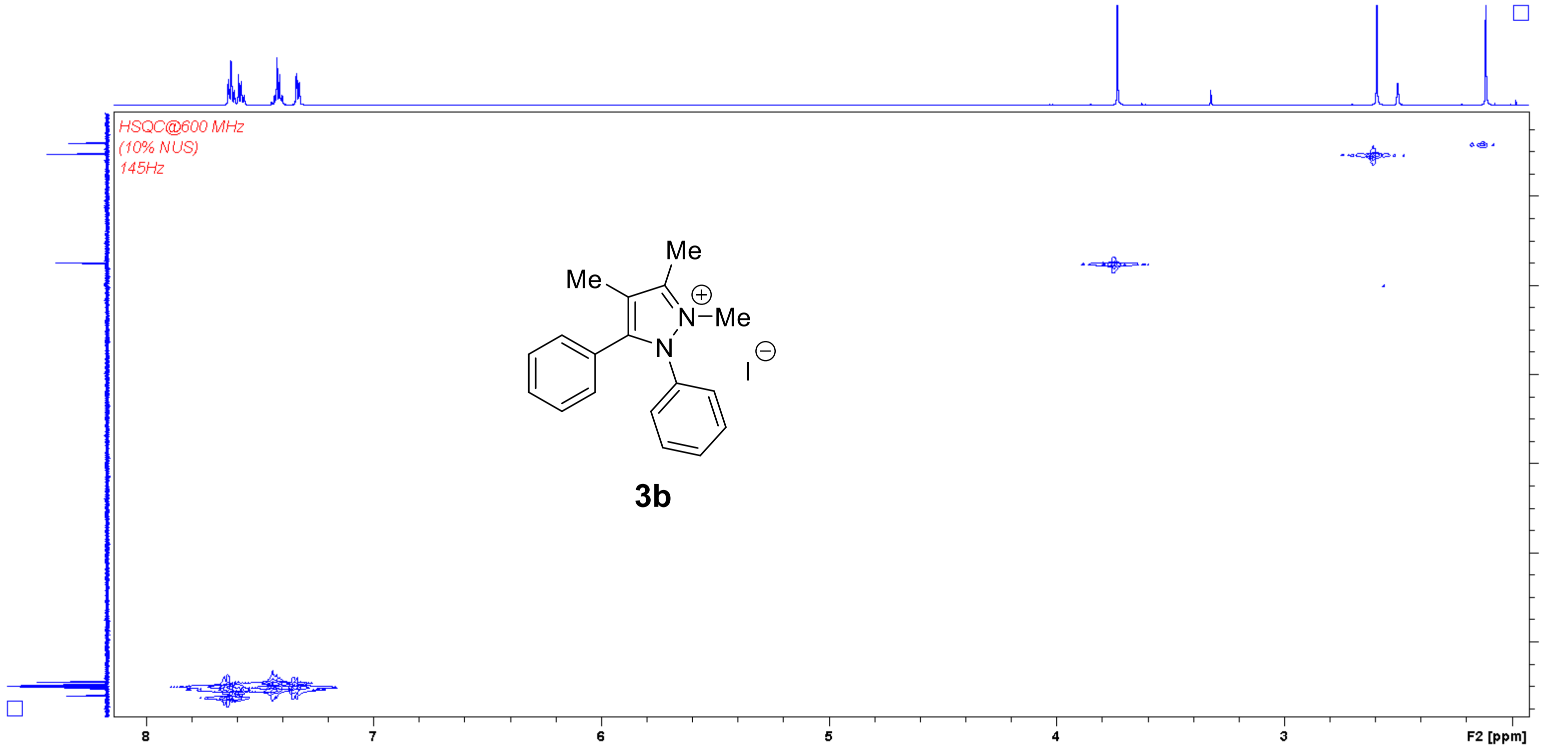
HSQC@600 MHz
(10% NUS)
145Hz
Me
Me
N−Me
N
I
3b
8
7
6
5
4
3
F2 [ppm]
F1 [ppm]
20
40
60
80
100
120

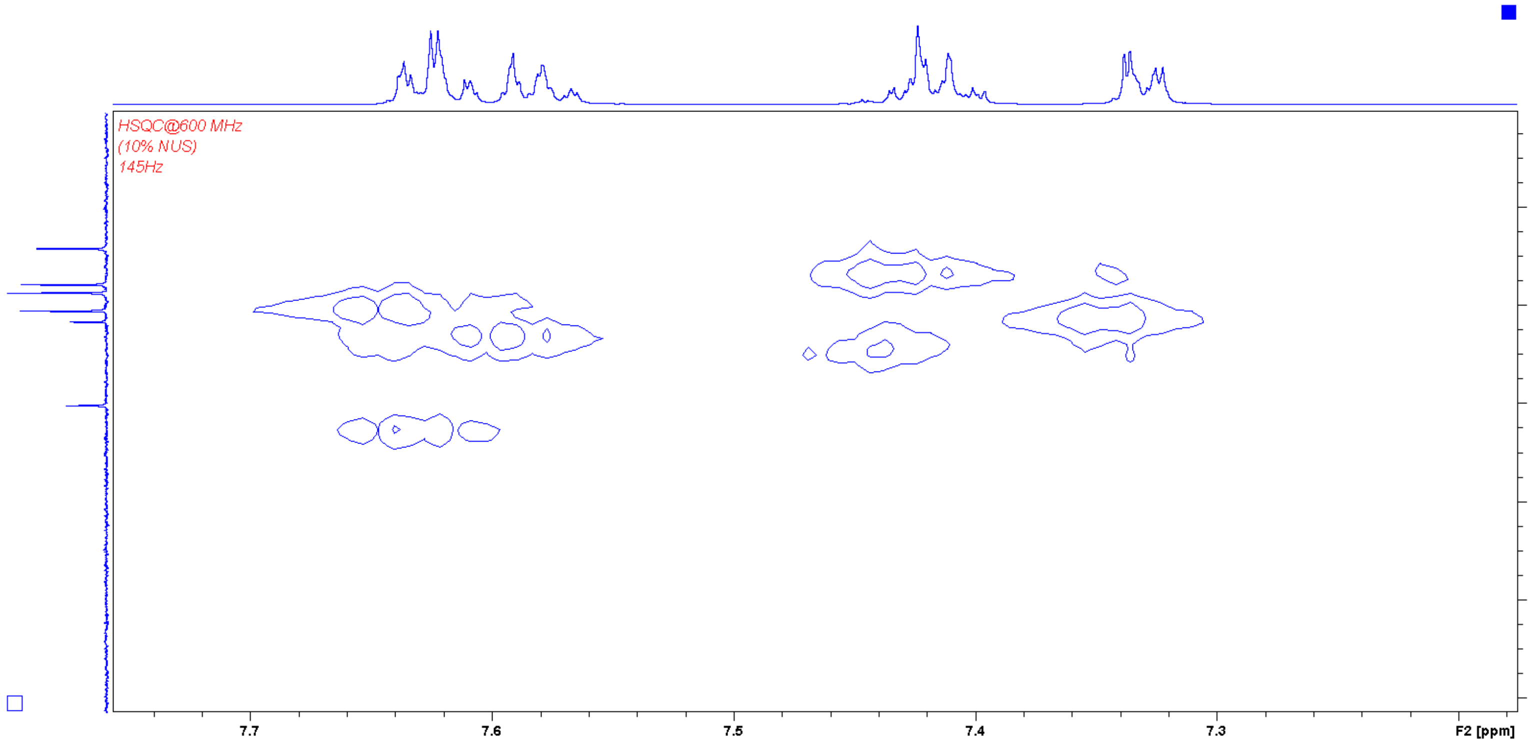
HSQC@600 MHz
(10% NUS)
145Hz
7.7
7.6
7.5
7.4
7.3
F2 [ppm]
F1 [ppm]
128
130
132
134
136

HMBC

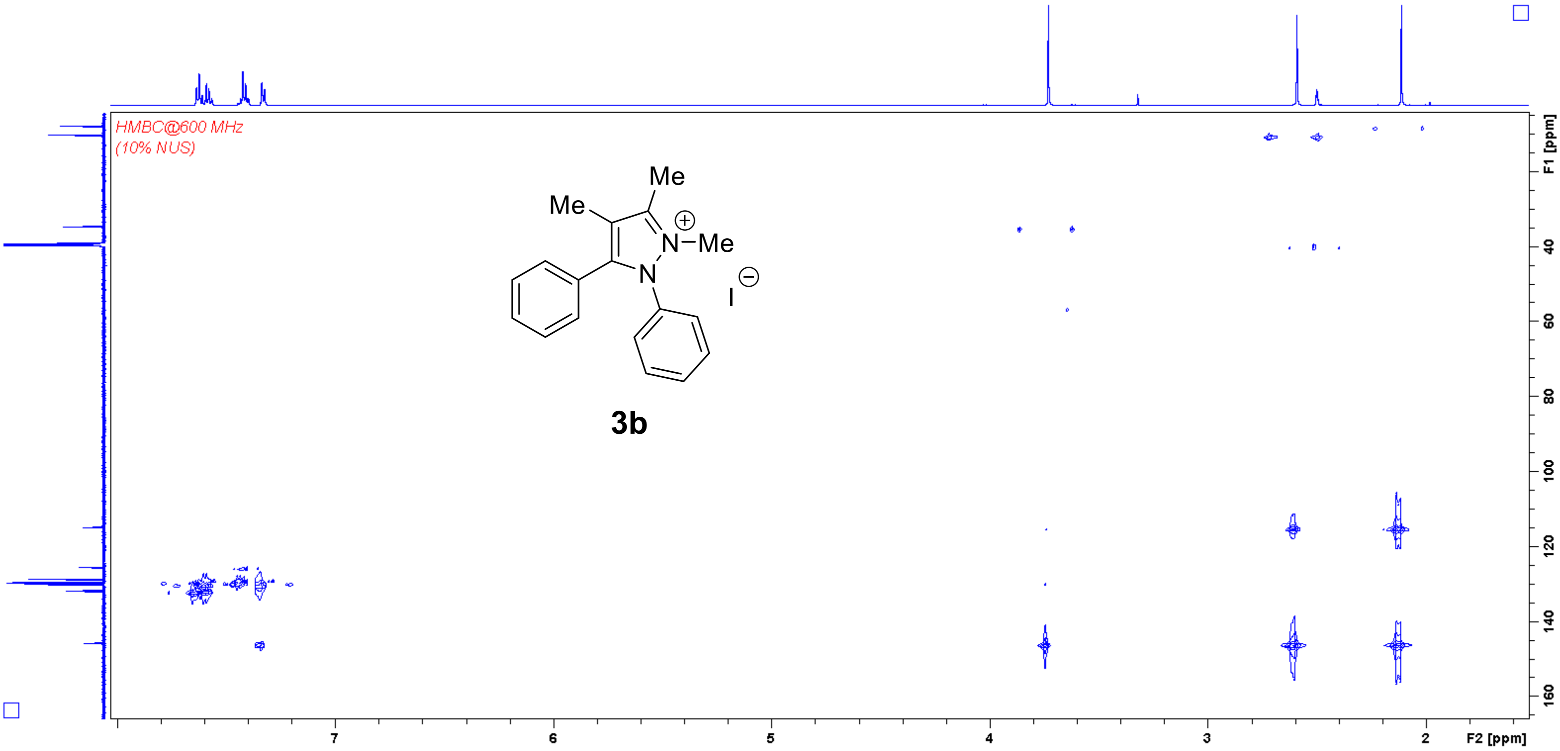

HMBC@600 MHz
(10% NUS)
Me
Me
N−Me
N
I
3b
F1 [ppm]
F2 [ppm]

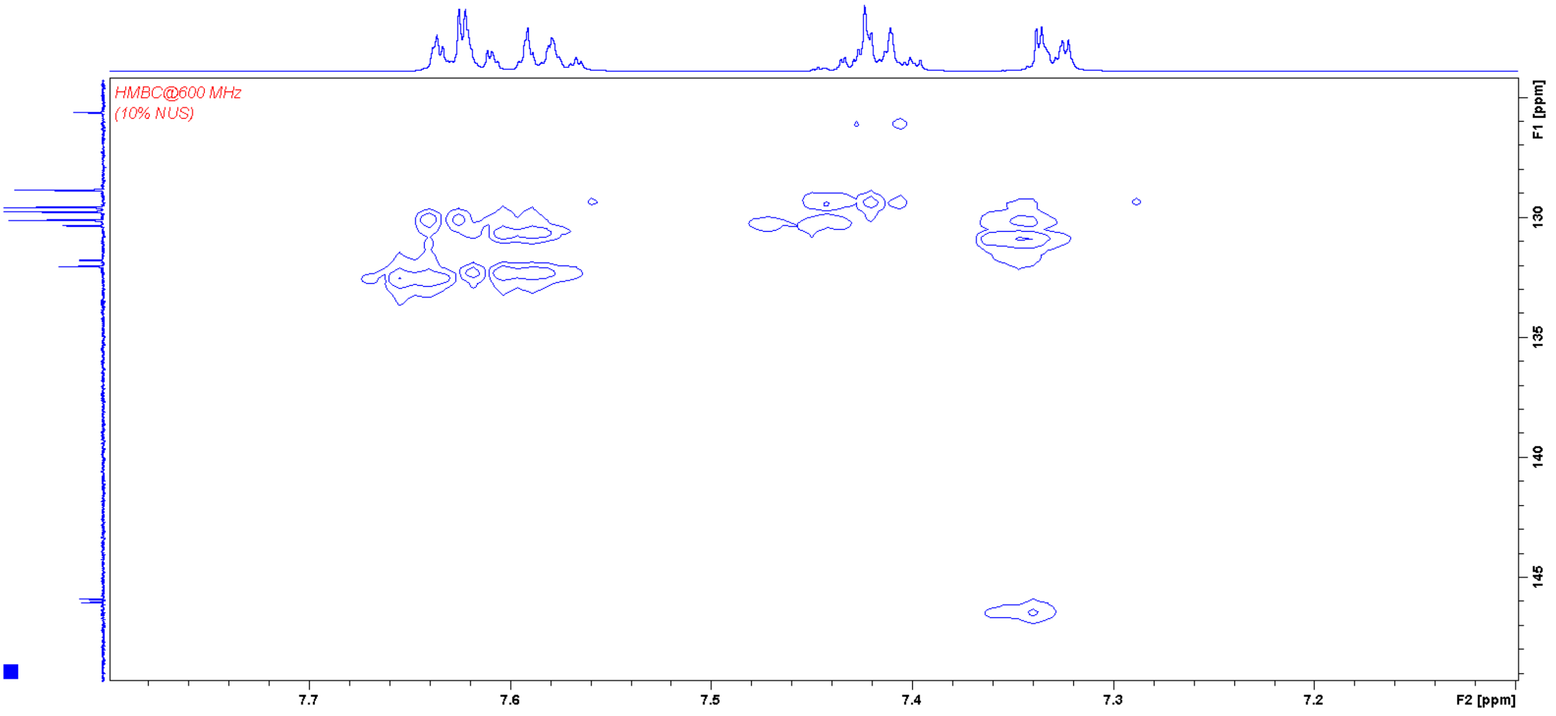

HMBC@600 MHz
(10% NUS)
F1 [ppm]
F2 [ppm]

General procedure of NHO synthesis

In an oven-dried Schlenk flask was added indazolium/pyrazolium salt (1.0 Eq), anhydrous THF (10 mL) and potassium hydride (2.0 Eq) under nitrogen atmosphere. The suspension was stirred at room temperature for 24 h and filtrated through a glass filter under nitrogen protection. The desired NHO solution is subsequently utilized as a reactant, or alternatively, isolated through the removal of the solvent in vacuum.

Synthesis of 2,3-dihydro-1,2-dimethyl-3-methylene-1*H*-indazole **2a**

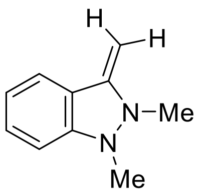

Followed by general procedure of NHO synthesis, **2a** was obtained as yellow-brown oil (54 mg, 84%).

^{1}H-NMR (DMSO-d$_6$, 600 MHz): 7.43 (d, J = 7.7 Hz, 1H, Ar-H), 7.27 (t, J = 7.7 Hz, 1H, Ar-H), 6.99 (d, J = 7.7 Hz, 1H, Ar-H), 6.95 (t, J = 7.7 Hz, 1H, Ar-H), 4.23 (d, J = 1.5 Hz, 1H, CH$_2$), 3.78 (d, J = 1.5 Hz, 1H, CH$_2$), 2.93 (s, 3H, N-Me), 2.86 (s, 3H, N-Me) ppm.

^{13}C{^{1}H}-NMR (DMSO-d$_6$, 150 MHz): 151.5 (o, Ar-C), 150.3 (o, Ar-C), 129.6 (+, Ar-C), 124.6 (o, Ar-C), 121.5 (+, Ar-C), 120.4 (+, Ar-C), 111.2 (+, Ar-C), 71.8 (-, CH$_2$), 40.0 (+, N-Me), 37.4 (+, N-Me) ppm.

IR (ATR): $\tilde{\nu}$ = 1625 (conjugated C=C), 1152 (indazole ring) cm^{-1}.

ESI-MS (m/z): calculated for [$C_{10}H_{12}N_2$+H]$^+$: 161.1073, found 161.1073.

^{1}H-NMR

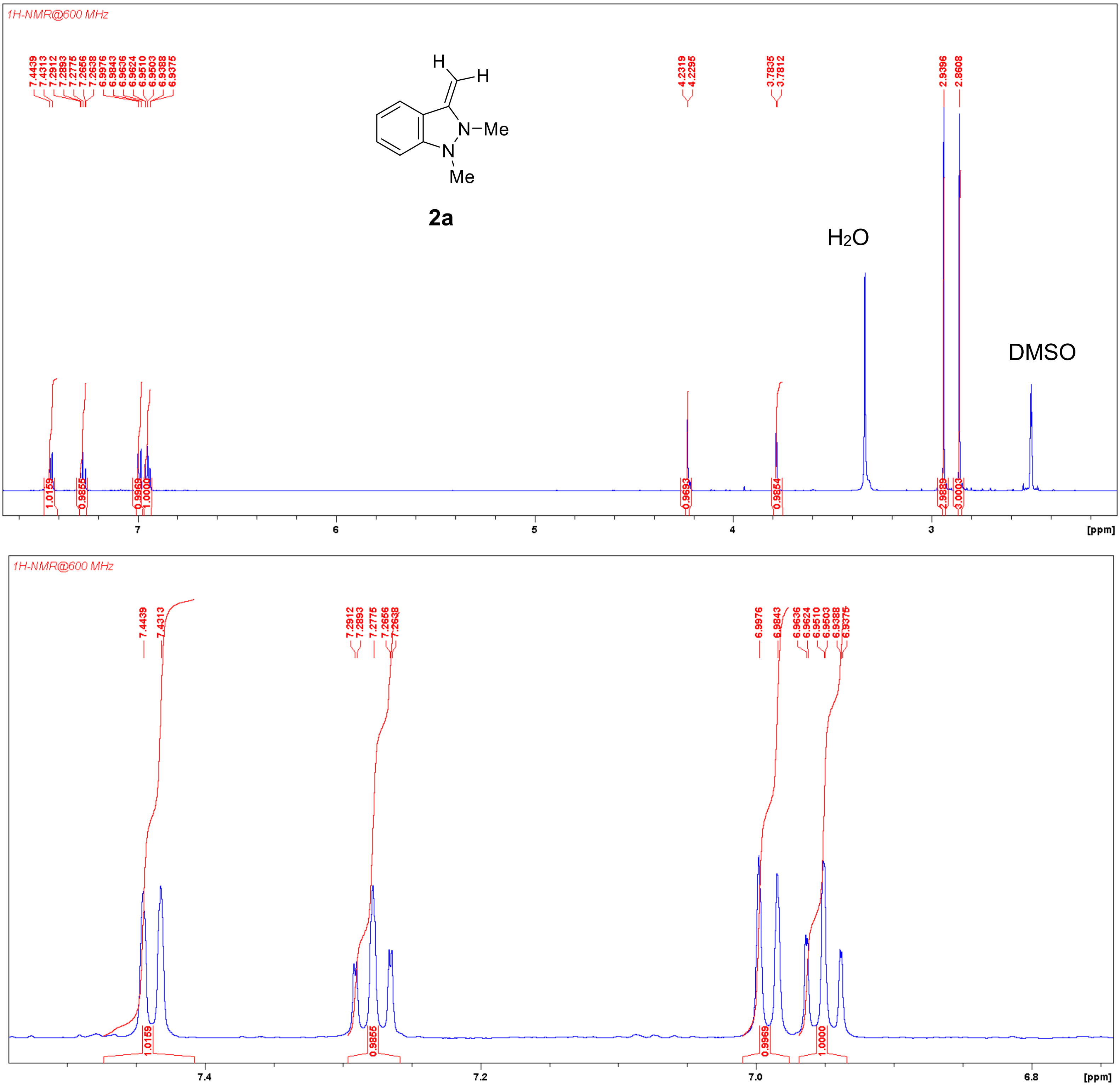

1H-NMR@600 MHz
7.4439
7.4313
7.2912
7.2893
7.2775
7.2656
7.2638
6.9976
6.9843
6.9636
6.9624
6.9510
6.9503
6.9388
6.9375
4.2319
4.2295
3.7835
3.7812
2.9396
2.8608
H
H
N−Me
N
Me
2a
H2O
DMSO
1.0159
0.9855
0.9969
1.0000
0.9693
0.9854
2.9889
3.0003
7
6
5
4
3
[ppm]
1H-NMR@600 MHz
7.4439
7.4313
7.2912
7.2893
7.2775
7.2656
7.2638
6.9976
6.9843
6.9636
6.9624
6.9510
6.9503
6.9388
6.9375
1.0159
0.9855
0.9969
1.0000
7.4
7.2
7.0
6.8
[ppm]

$^{13}C\{^{1}H\}$-NMR

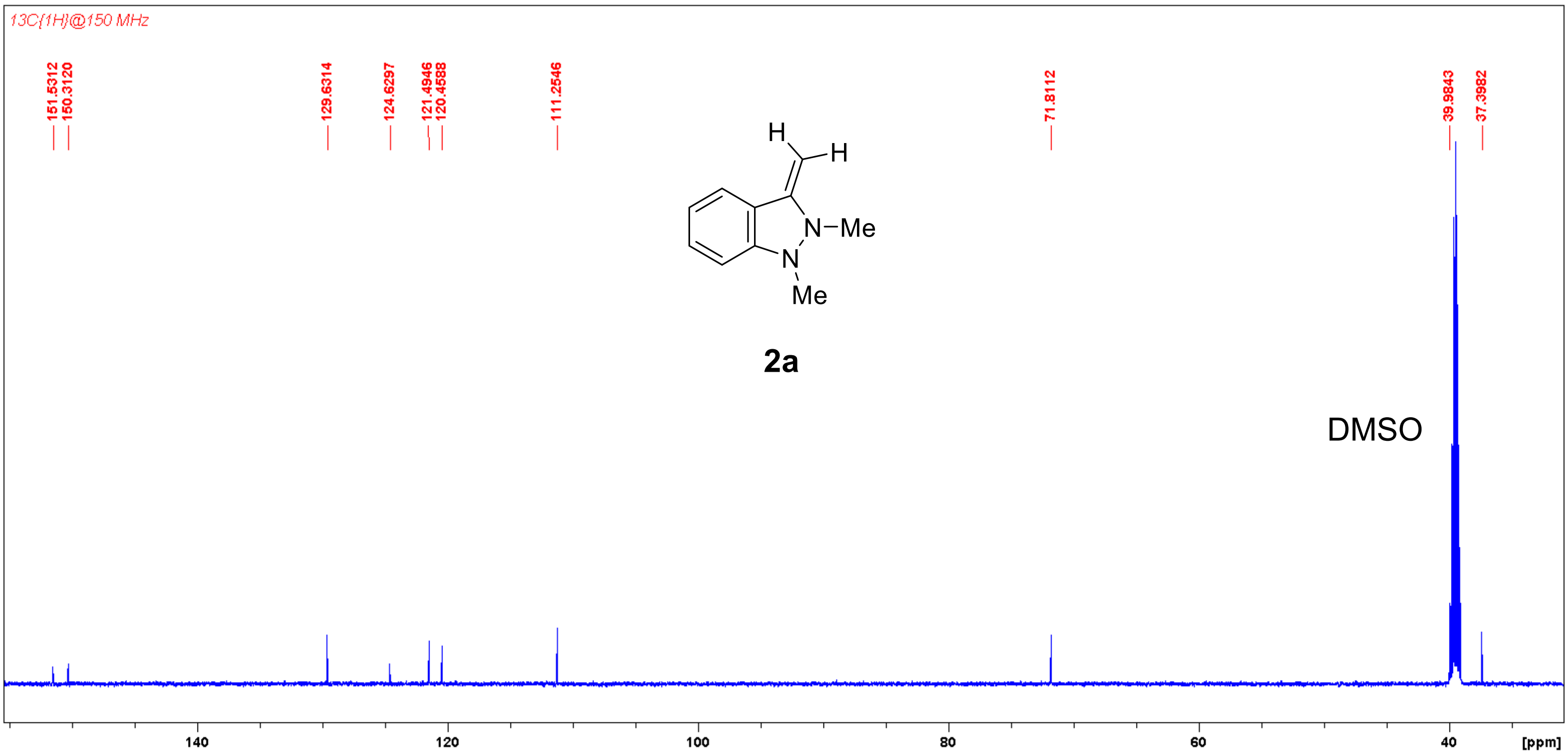

^{13}C-DEPT

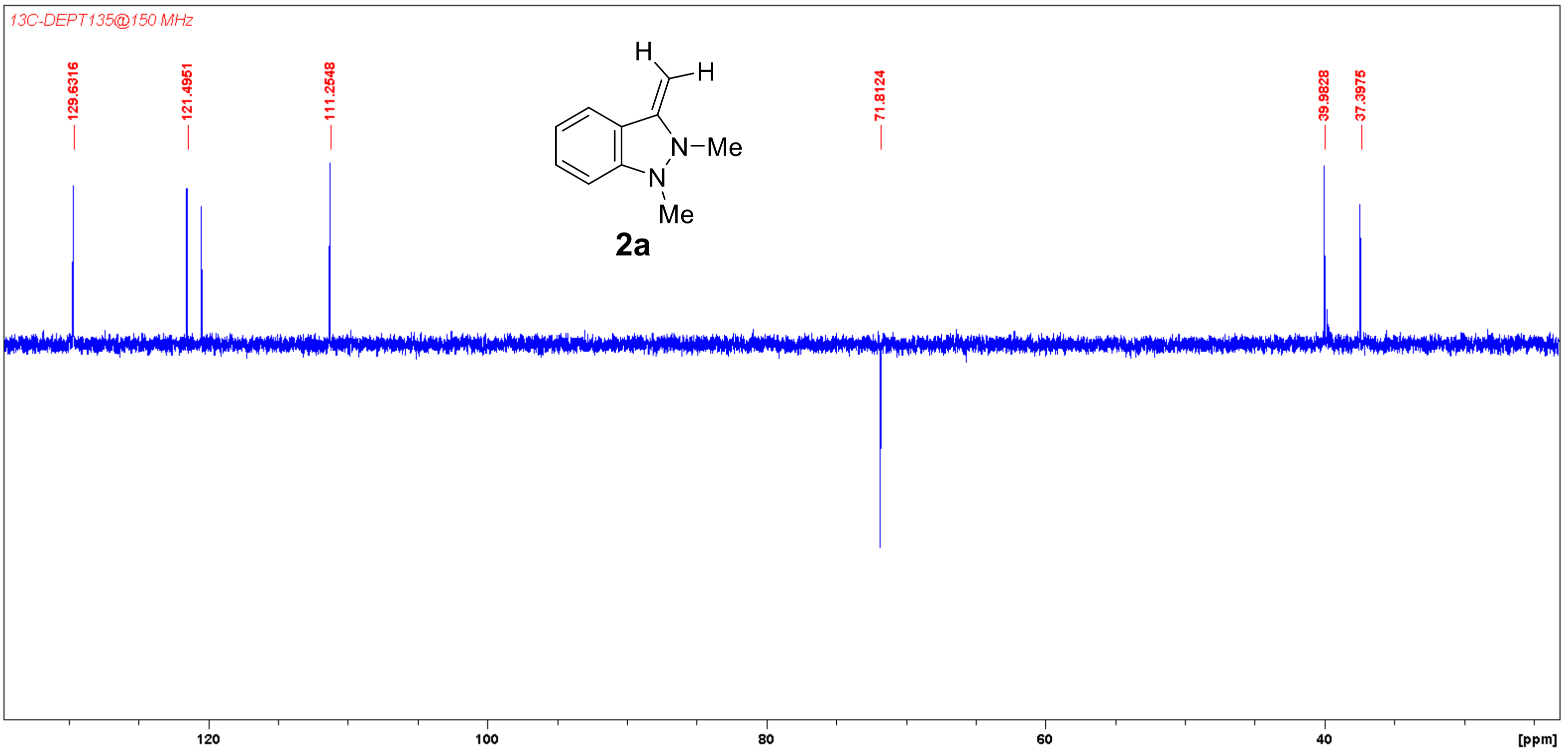

H,H-COSY

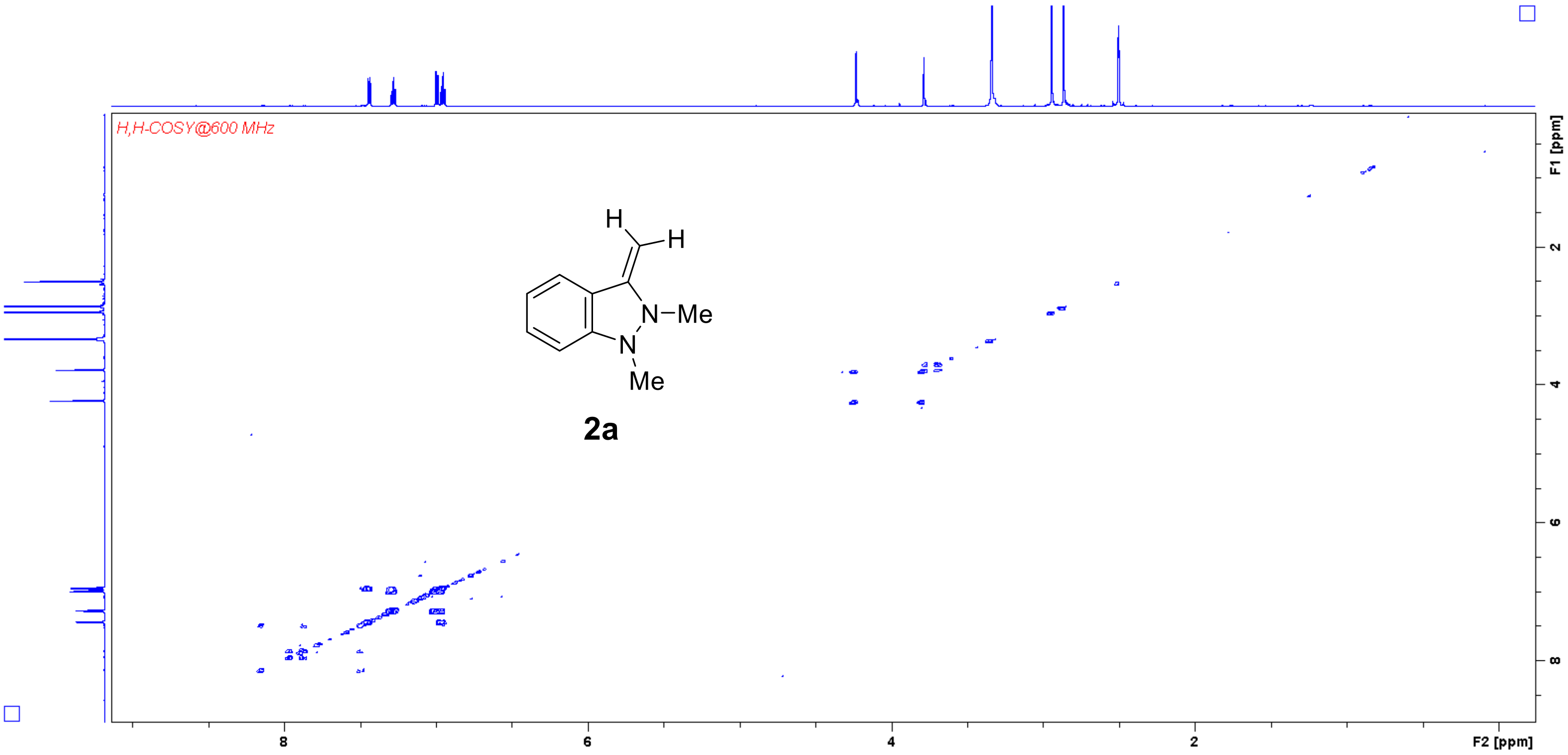
H,H-COSY@600 MHz
H
H
N−Me
N
Me
2a
F1 [ppm]
F2 [ppm]
8
6
4
2

HSQC

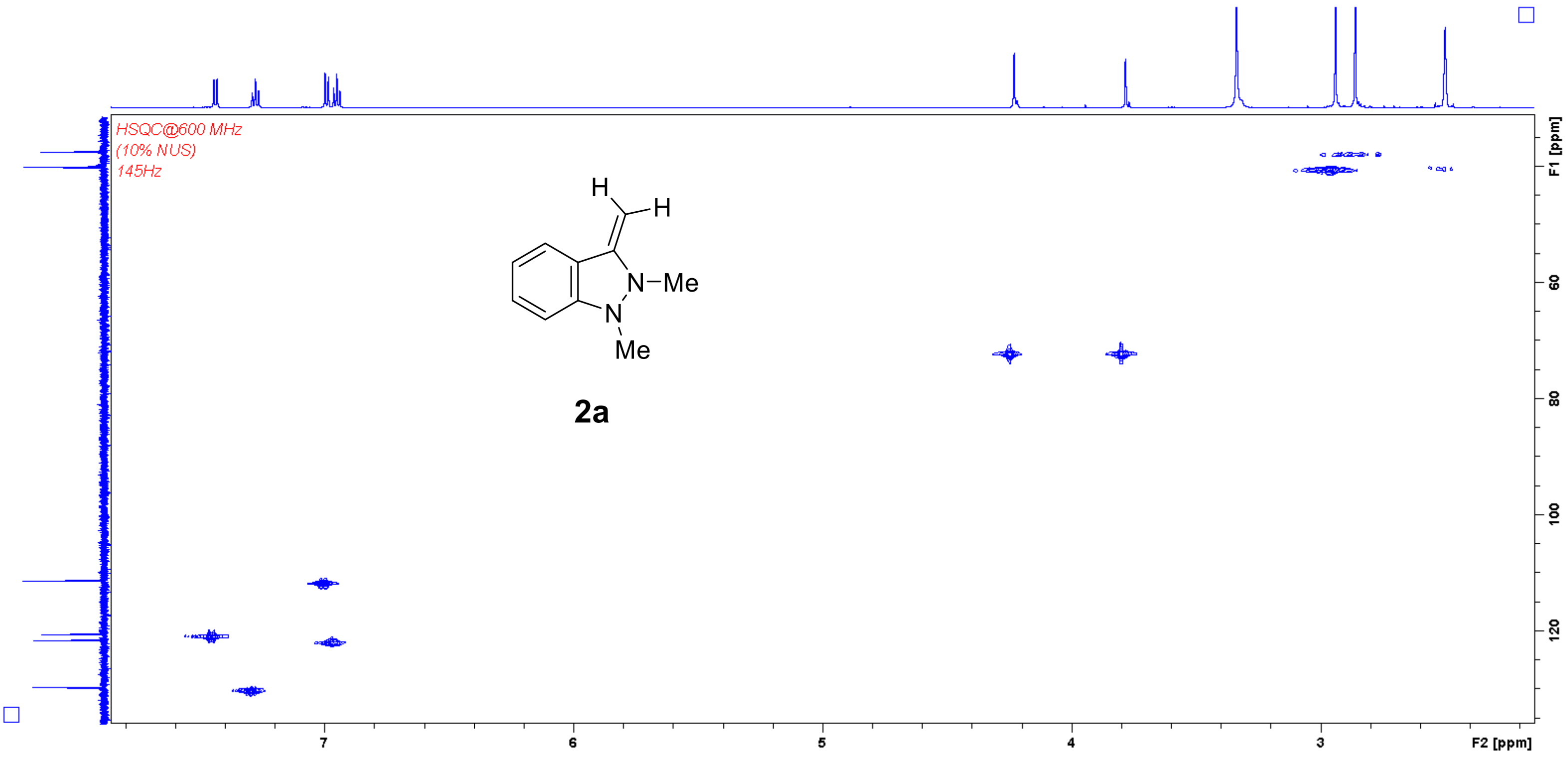
HSQC@600 MHz
(10% NUS)
145Hz
H
H
N−Me
N
Me
2a
F1 [ppm]
60
80
100
120
F2 [ppm]
7
6
5
4
3

HMBC

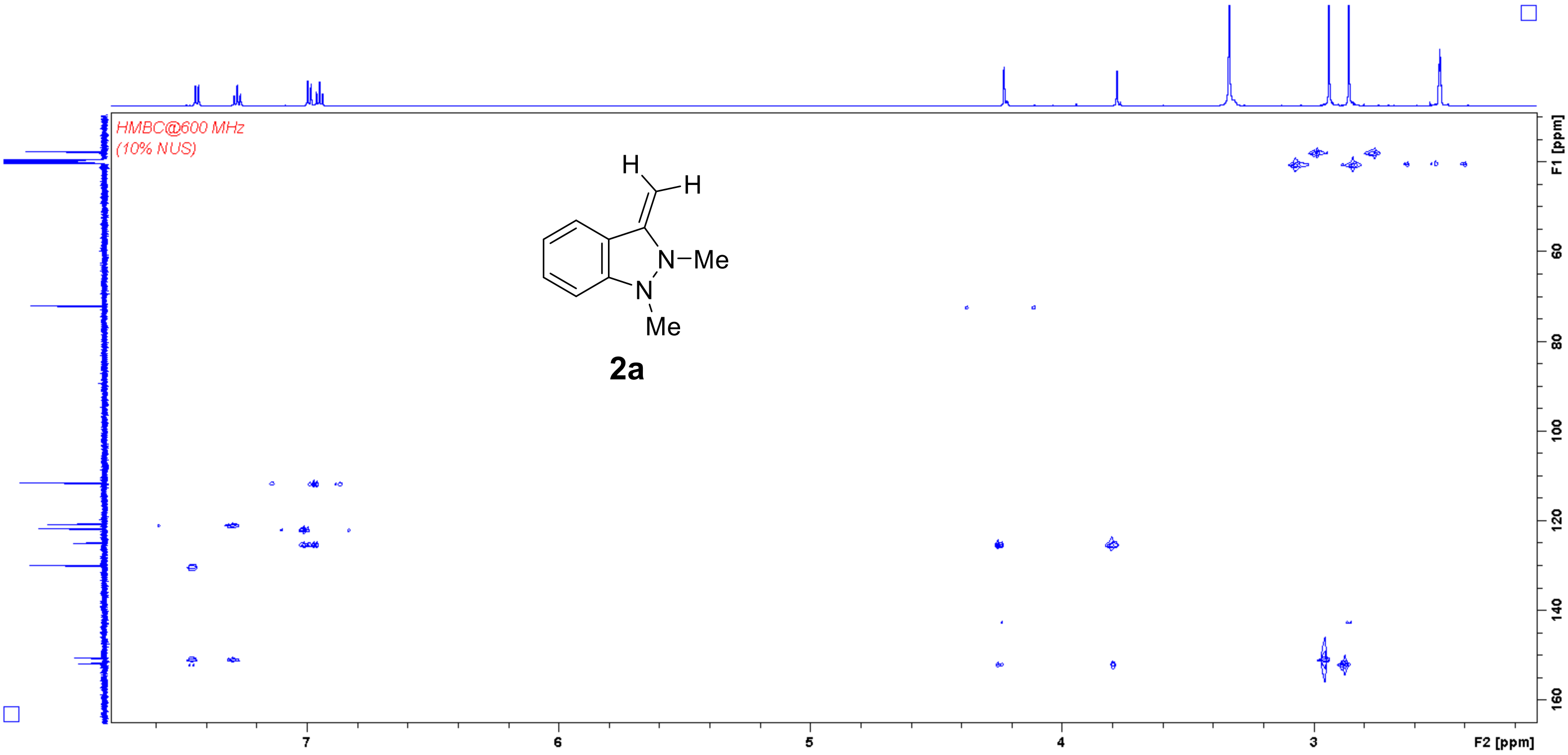

Synthesis of 2,3-dihydro-2-methyl-3-methylene-1-phenyl-1*H*-indazole **2b**

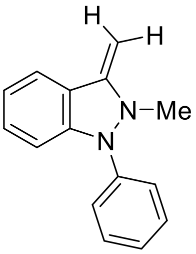

A Schlenk flask was dried with a heat gun in vacuum. After the Schlenk flask was cooled to room temperature, 2,3-dimethyl-1-phenyl-1*H*-indazolium tetrafluoroborate (1 Eq, 105 mg, 0.339 mmol) was added and further dried in vacuum. Abs. THF (10 mL) was added under nitrogen atmosphere. After the salt was partially dissolved, LHMDS solution in THF (1.2 Eq, 1.0 M, 0.406 mL, 0.406 mmol) was added dropwise at room temperature. The mixture was stirred at room temperature for 2 h. The solvent was removed in vacuum. The crude product was washed with 5 mL diethyl ether and obtained as yellow oil with $LiBF_4$ as impurity (70 mg, 93%).

^{1}H-NMR (DMSO-d_6, 600 MHz): 7.56 (d, J = 7.7 Hz, 1H, Ar-H), 7.47 (t, J = 8.3 Hz, 2H, Ar-H), 7.39 (d, J= 8.3 Hz, 2H, Ar-H), 7.26 (t, J = 8.3 Hz, 1H, Ar-H), 7.22 (t, J = 7.7 Hz, 1H, Ar-H), 7.01 (t, J = 7.7 Hz, 1H, Ar-H), 6.90 (d, J = 7.7 Hz, 1H, Ar-H), 4.42 (d, J = 1.5 Hz, 1H, CH_2), 3.99 (d, J = 1.5 Hz, 1H, CH_2), 2.84 (s, 3H, Me) ppm.

^{13}C{^{1}H}-NMR (DMSO-d_6, 150 MHz): 150.9 (o, Ar-C), 147.1 (o, Ar-C), 144.6 (o, Ar-C), 129.8 (+, Ar-C), 129.5 (+, Ar-C), 125.6 (+, Ar-C), 124.2 (o, Ar-C), 122.8 (+, Ar-C), 122.1 (+, Ar-C), 120.8 (+, Ar-C), 110.0 (+, Ar-C), 73.2 (-, CH_2), 38.6 (+, Me) ppm.

ESI-MS (m/z): calculated for $[C_{15}H_{14}N_2+H]^+$: 223.1230, found 223.1236.

IR (ATR): $\tilde{\nu}$ = 1630 (conjugated C=C) cm^{-1}.

^{1}H-NMR

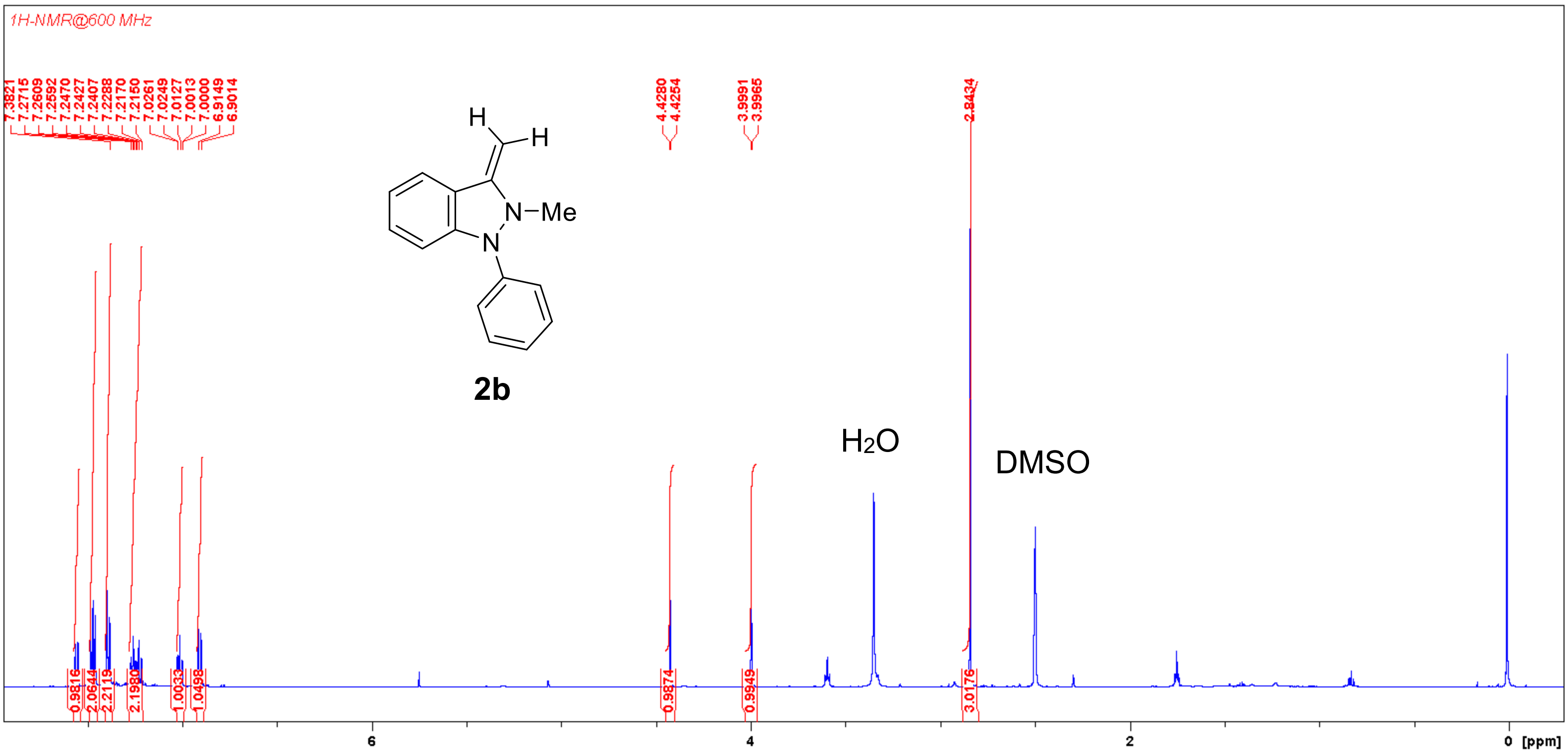

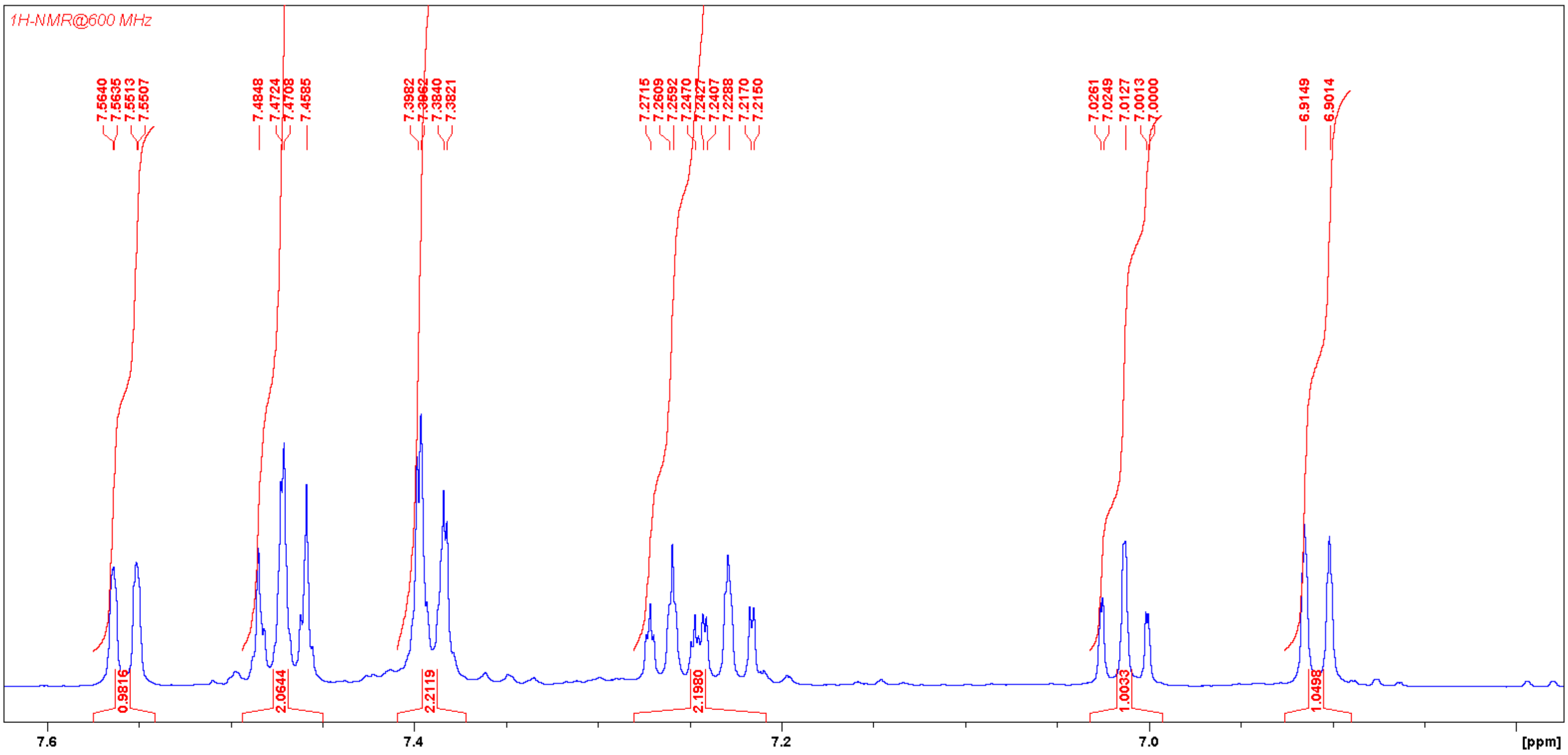

$^{13}C\{^{1}H\}$-NMR

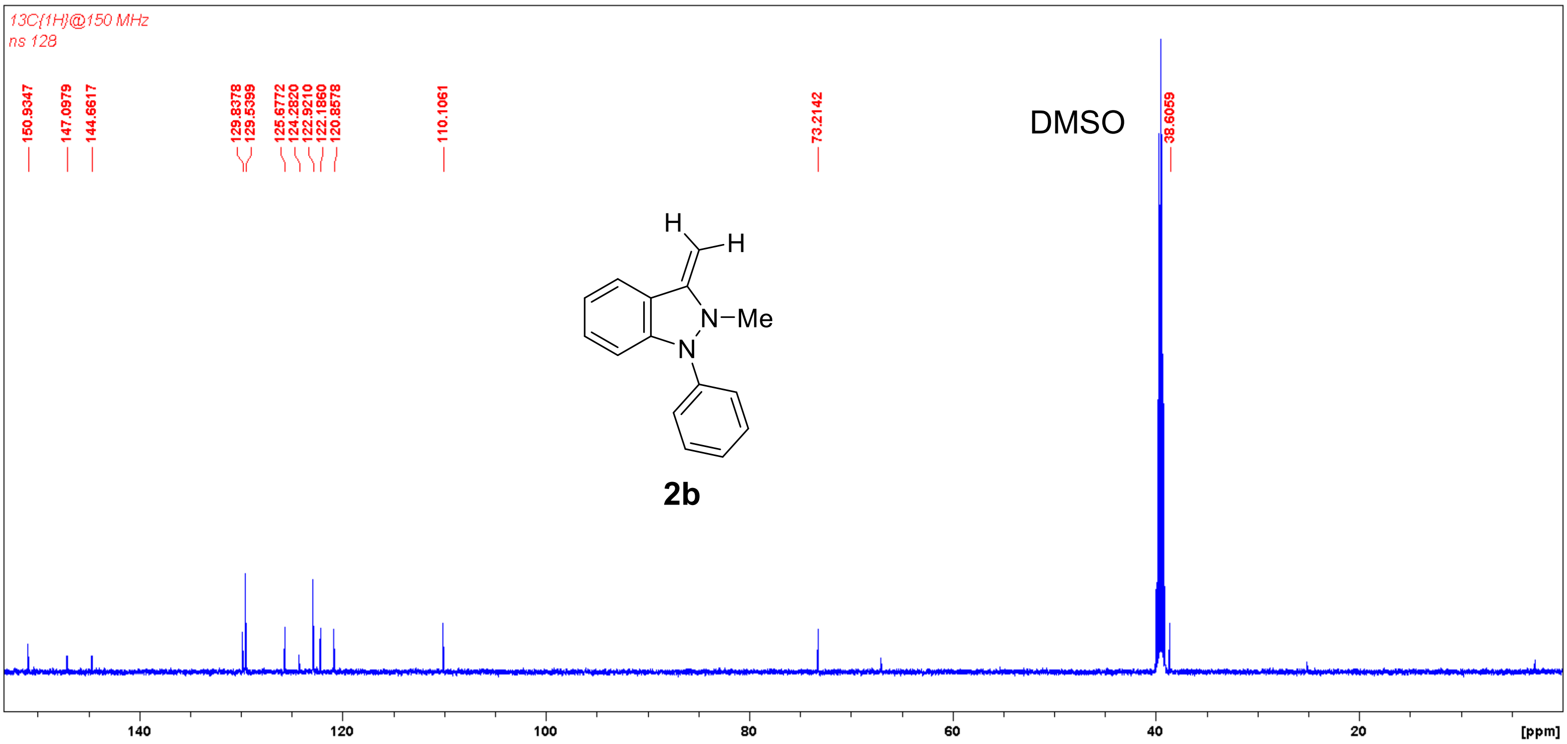

^{13}C-DEPT

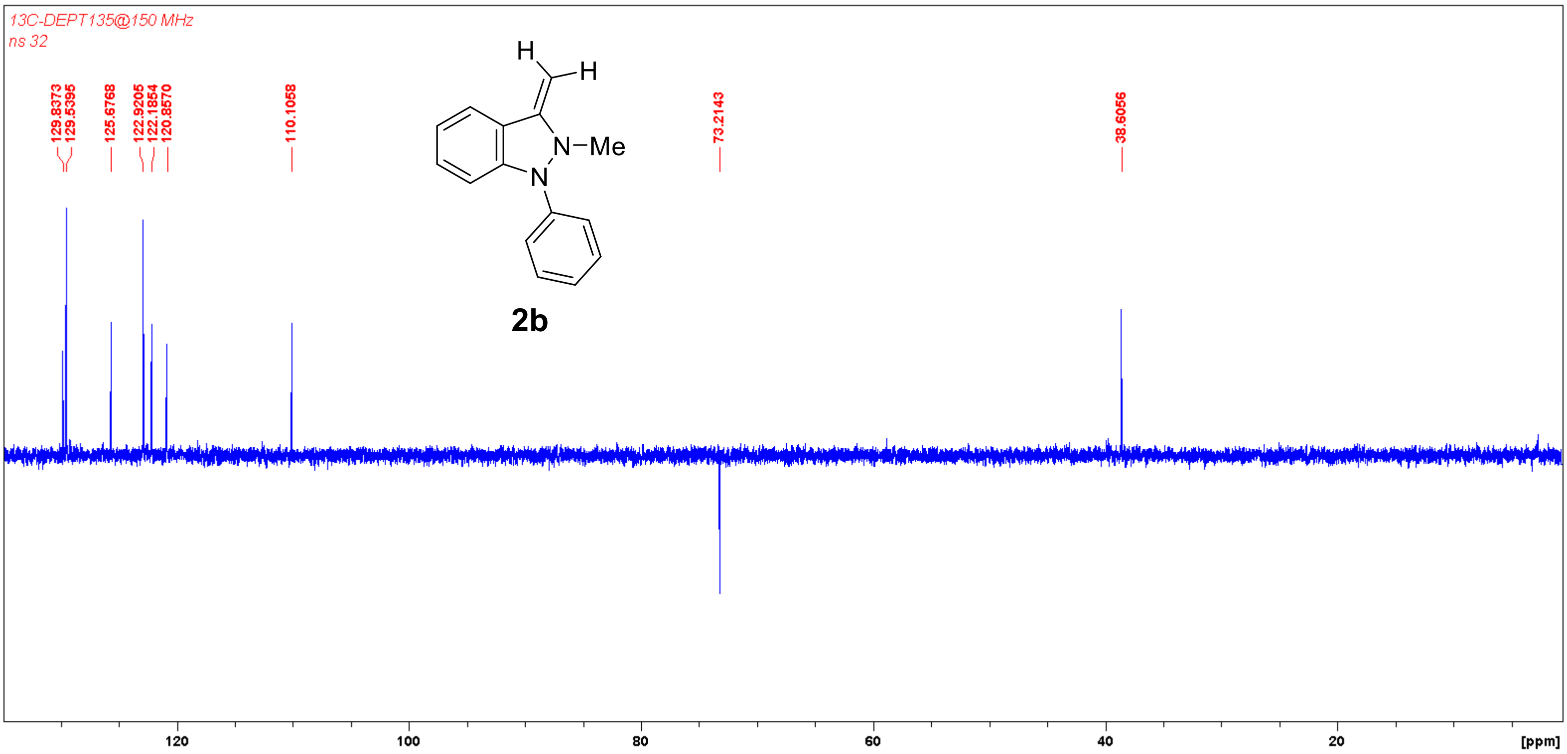

H,H-COSY

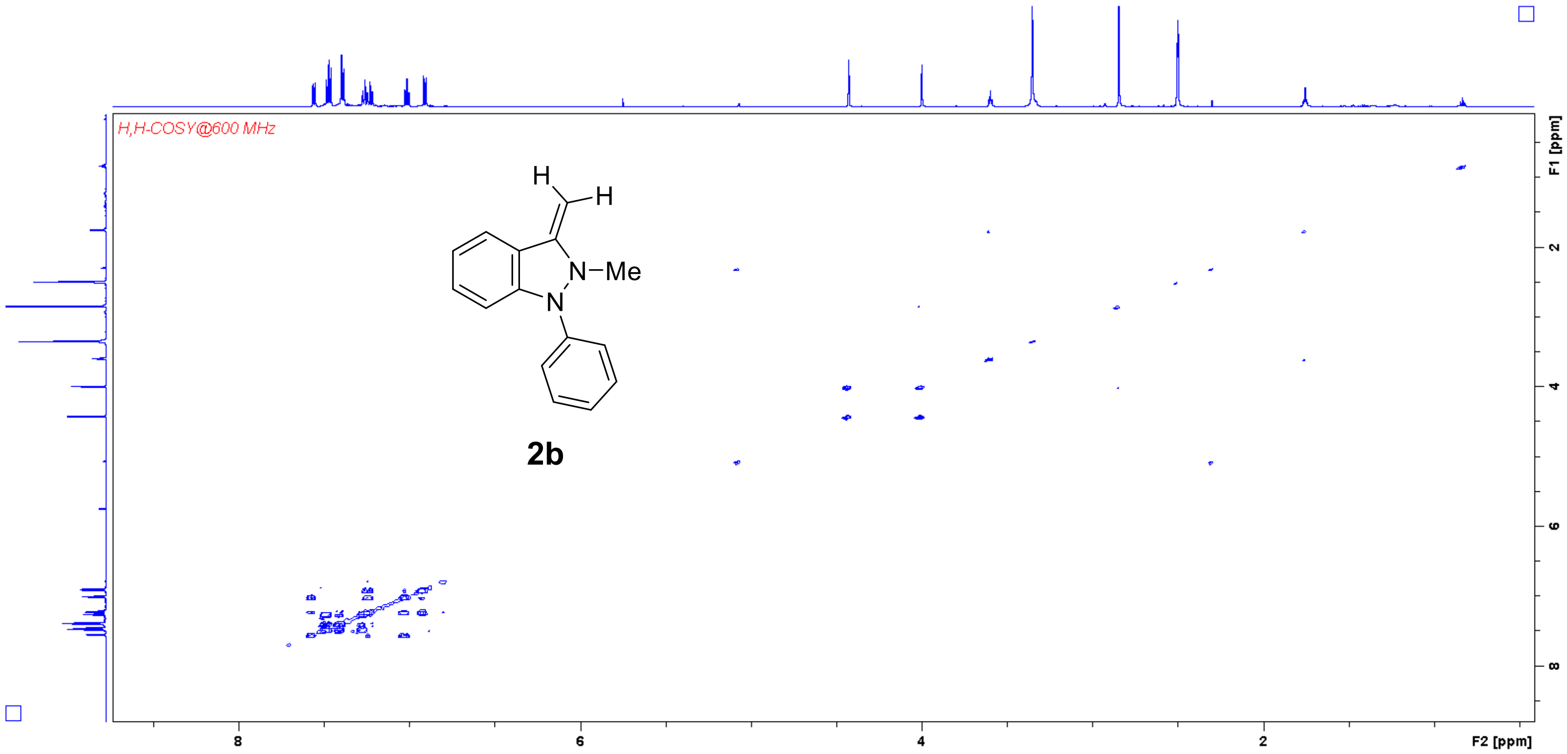

H,H-COSY@600 MHz
H
H
N–Me
N
2b
F1 [ppm]
F2 [ppm]

HSQC

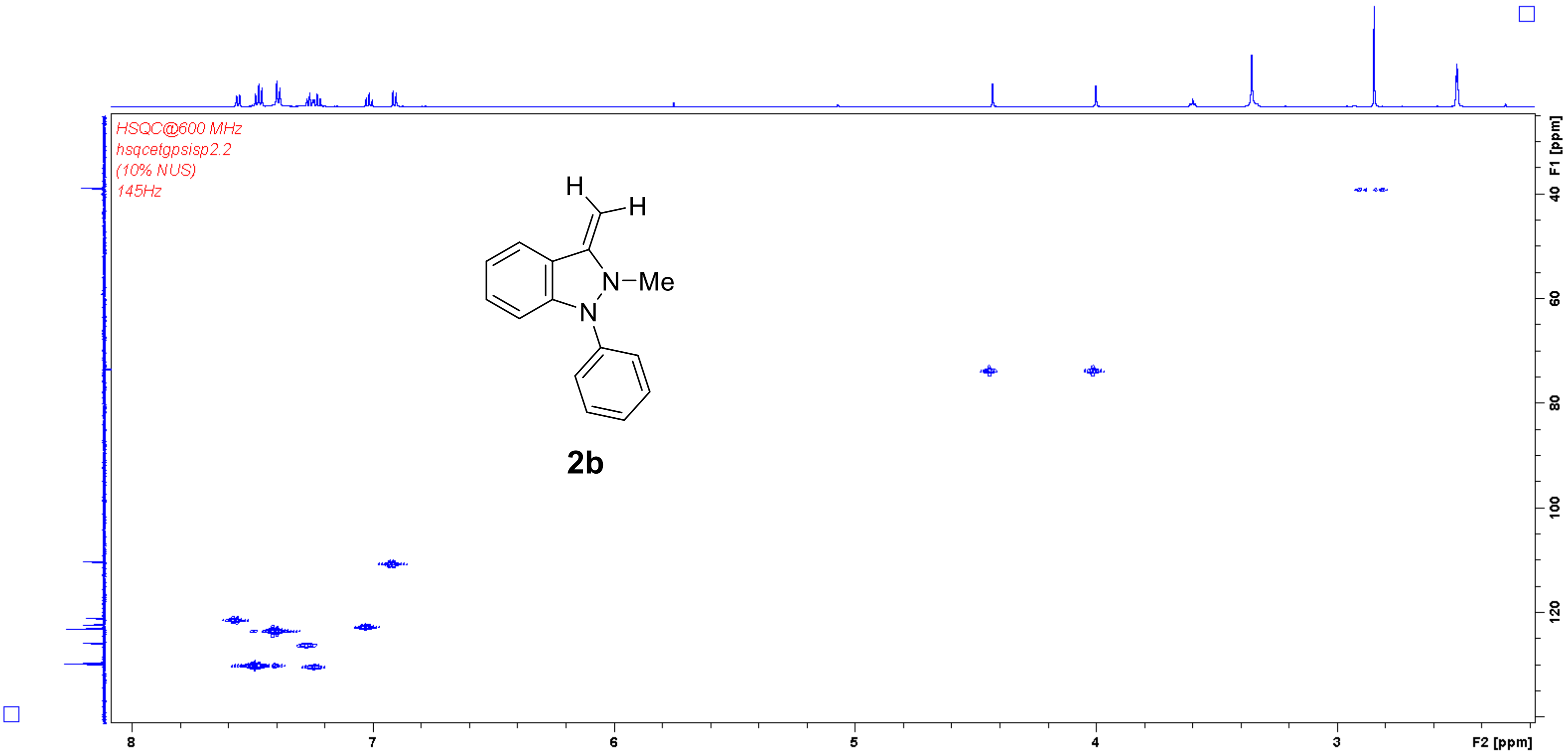

HSQC@600 MHz
hsqcetgpsisp2.2
(10% NUS)
145Hz
H
H
N–Me
N
2b
F1 [ppm]
F2 [ppm]

HMBC

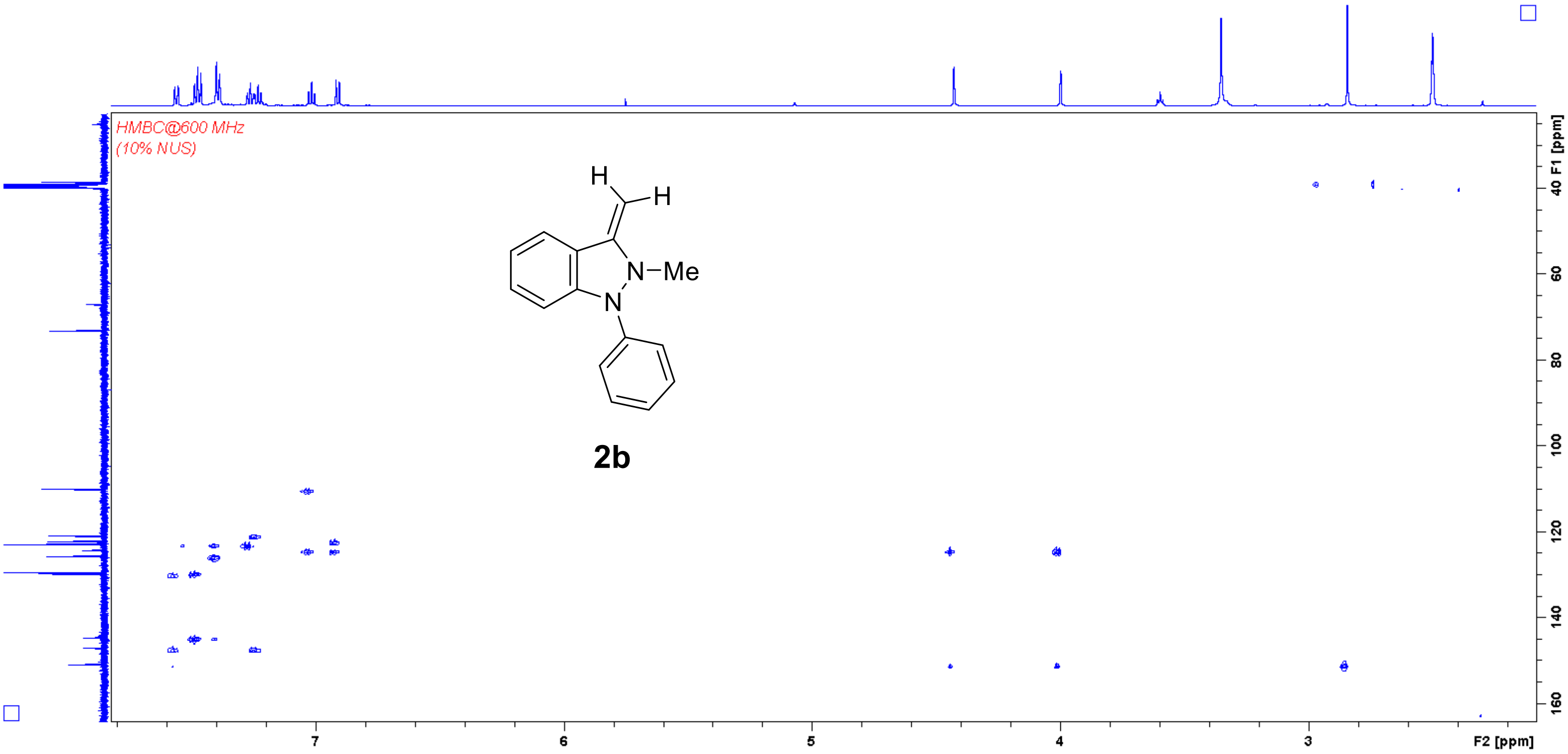
HMBC@600 MHz
(10% NUS)
H
H
N–Me
N
2b
40 F1 [ppm]
60
80
100
120
140
160
7
6
5
4
3
F2 [ppm]

Synthesis of 1-(4-methoxyphenyl)-2-methyl-3-methylene-2,3-dihydro-1*H*-indazole **2c**

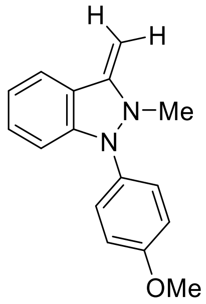

Followed by general procedure of NHO synthesis, **2c** was obtained as yellow-brown oil (56 mg, 84%).

^{1}H-NMR (DMSO-d$_6$, 600 MHz): 7.53 (d, J = 7.6 Hz, 1H, Ar-H), 7.28 (d, J = 8.7 Hz, 2H, Ar-H), 7.20 (t, J = 7.6 Hz, 1H, Ar-H), 7.02 (d, J = 8.7 Hz, 2H, Ar-H), 6.98 (t, J = 7.6 Hz, 1H, Ar-H), 6.67 (d, J = 7.6 Hz, 1H, Ar-H), 4.36 (d, J = 1.3 Hz, 1H, CH_2), 3.92 (d, J = 1.3 Hz, 1H, CH_2), 3.77 (s, 3H, OMe), 2.79 (s, 3H, N-Me) ppm.

^{13}C{^{1}H}-NMR (DMSO-d$_6$, 150 MHz): 157.7 (o, Ar-C), 150.9 (o, Ar-C), 148.3 (o, Ar-C), 137.7 (o, Ar-C), 129.7 (+, Ar-C), 125.7 (+, Ar-C), 124.3 (o, Ar-C), 121.9 (+, Ar-C), 120.7 (+, Ar-C), 114.7 (+, Ar-C), 110.5 (+, Ar-C), 72.5 (-, CH_2), 55.3 (+, OMe), 37.9 (+, N-Me) ppm.

ESI-MS (m/z): calculated for $[C_{16}H_{16}N_2O+H]^+$: 263.1548, found 263.1557.

IR (ATR): $\tilde{\nu}$ = 1627 (conjugated C=C), 1240 (indazole ring), 555 (C-O-C deformation) cm^{-1}.

^{1}H-NMR

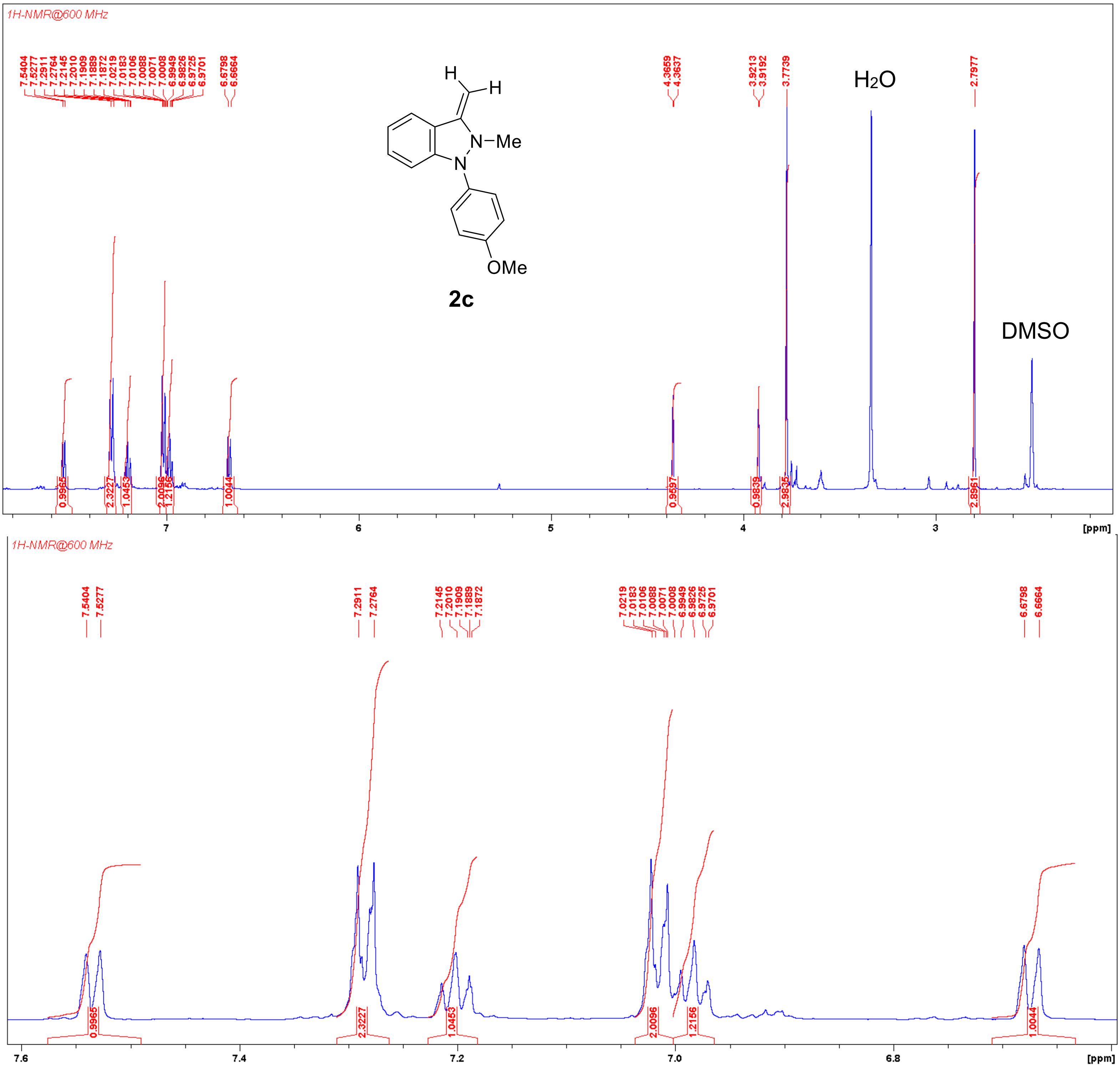

1H-NMR@600 MHz
7.5404
7.5277
7.2911
7.2764
7.2145
7.2010
7.1909
7.1889
7.1872
7.0219
7.0183
7.0106
7.0088
7.0071
7.0008
6.9949
6.9826
6.9725
6.9701
6.6798
6.6664
4.3659
4.3637
3.9213
3.9192
3.7739
2.7977
H2O
DMSO
N−Me
N
OMe
2c
0.9965
2.3227
1.0453
2.0096
1.2156
1.0044
0.9597
0.9839
2.9835
2.8961
[ppm]

$^{13}C\{^1H\}$-NMR

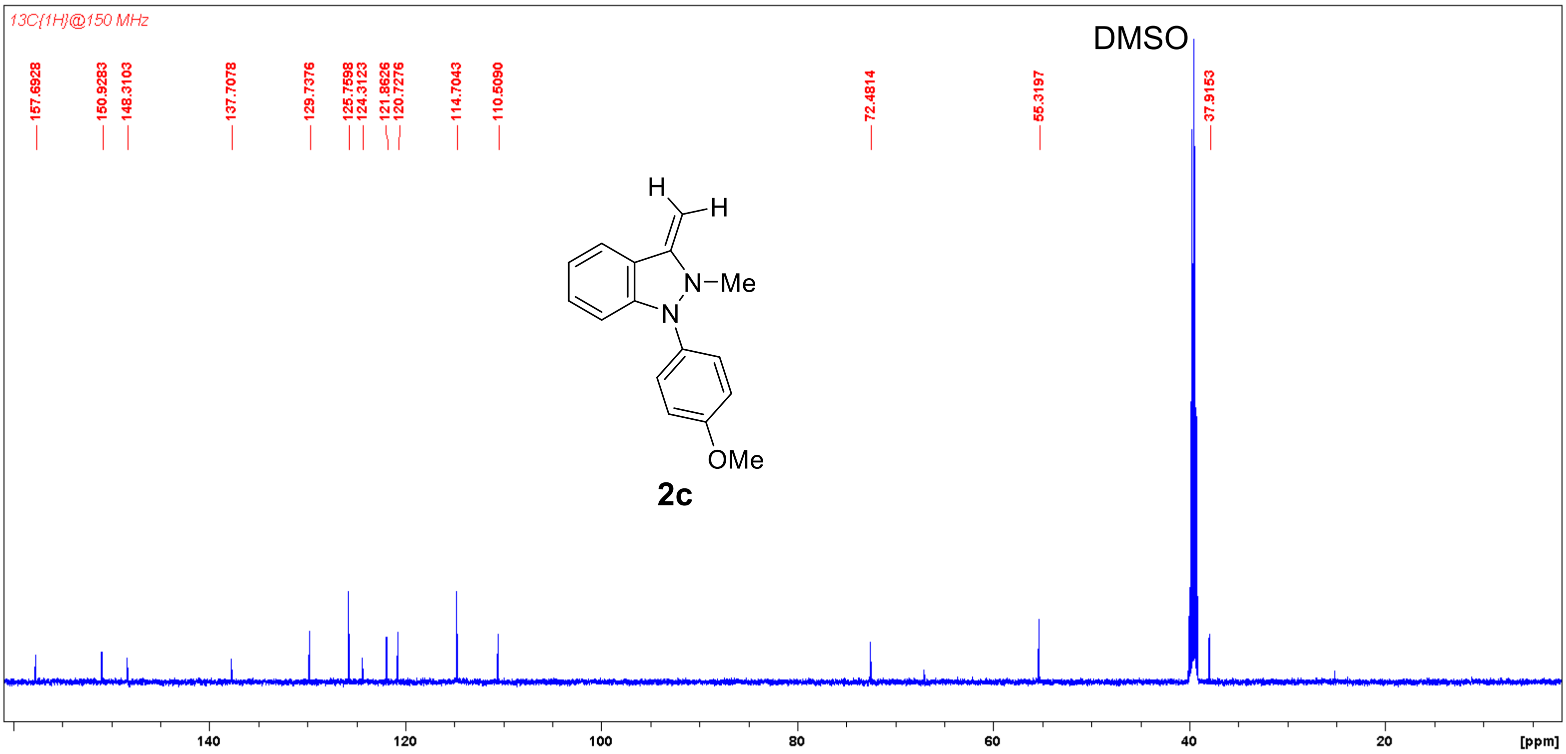

^{13}C-DEPT

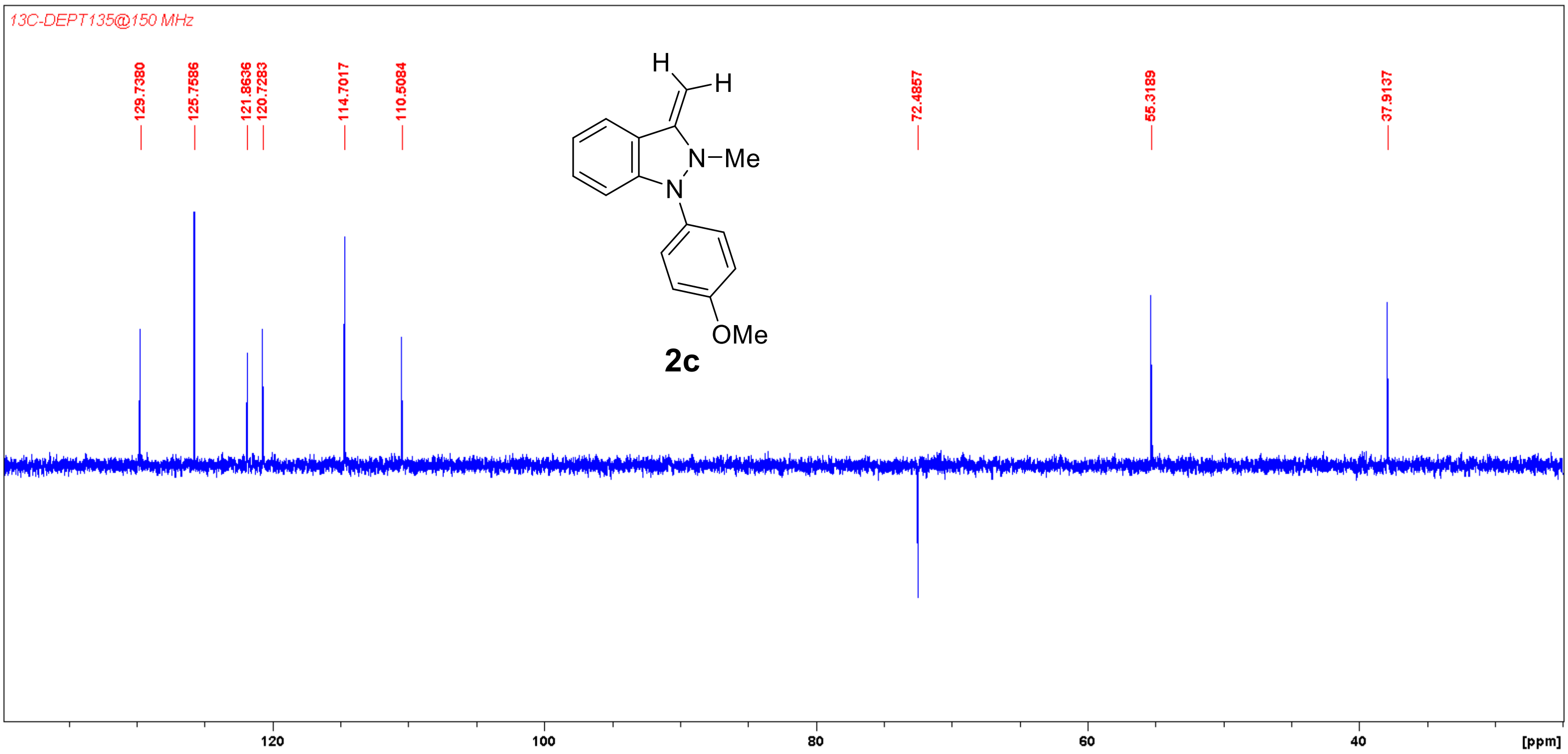

H,H-COSY

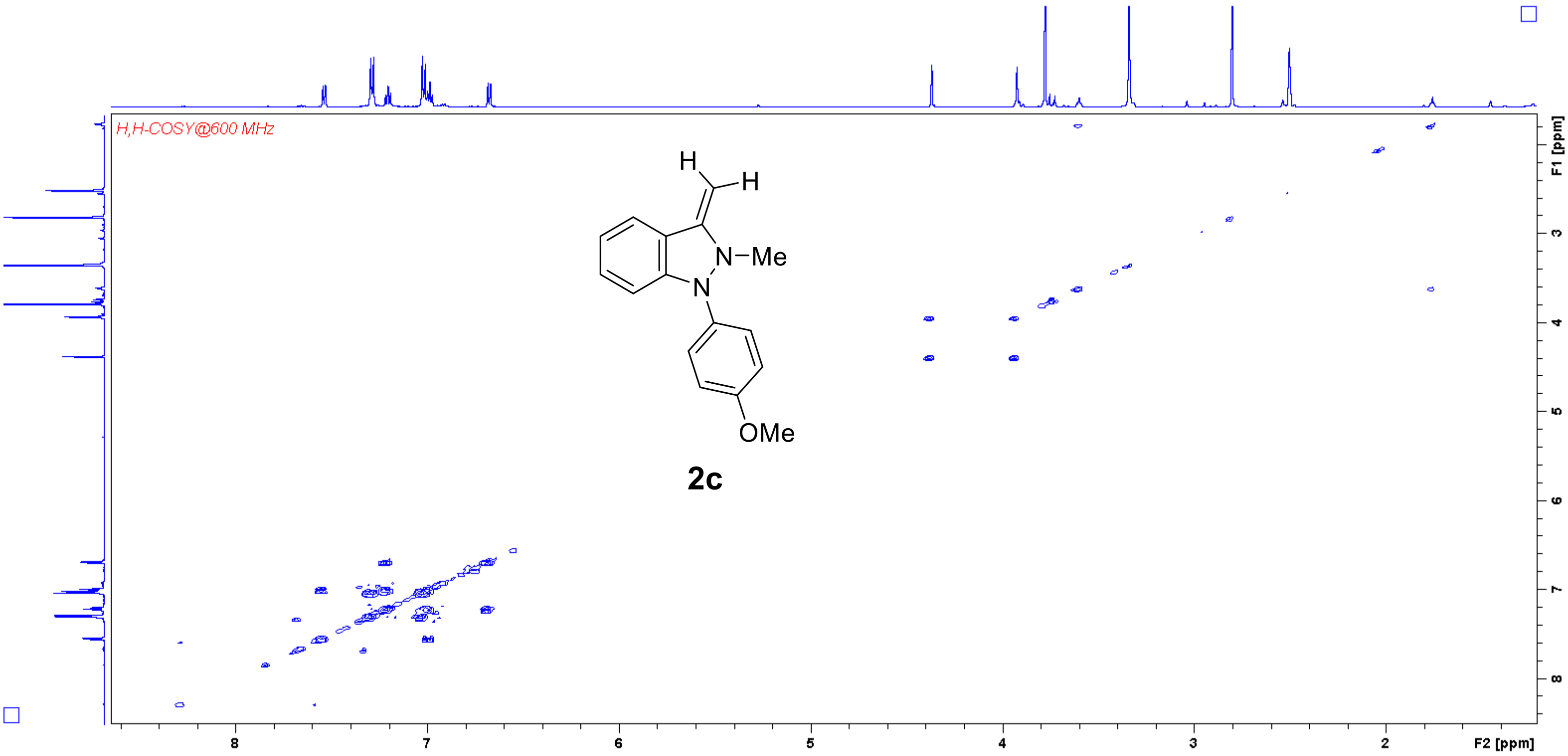
H,H-COSY@600 MHz
H
H
N−Me
N
OMe
2c
F1 [ppm]
F2 [ppm]

HSQC

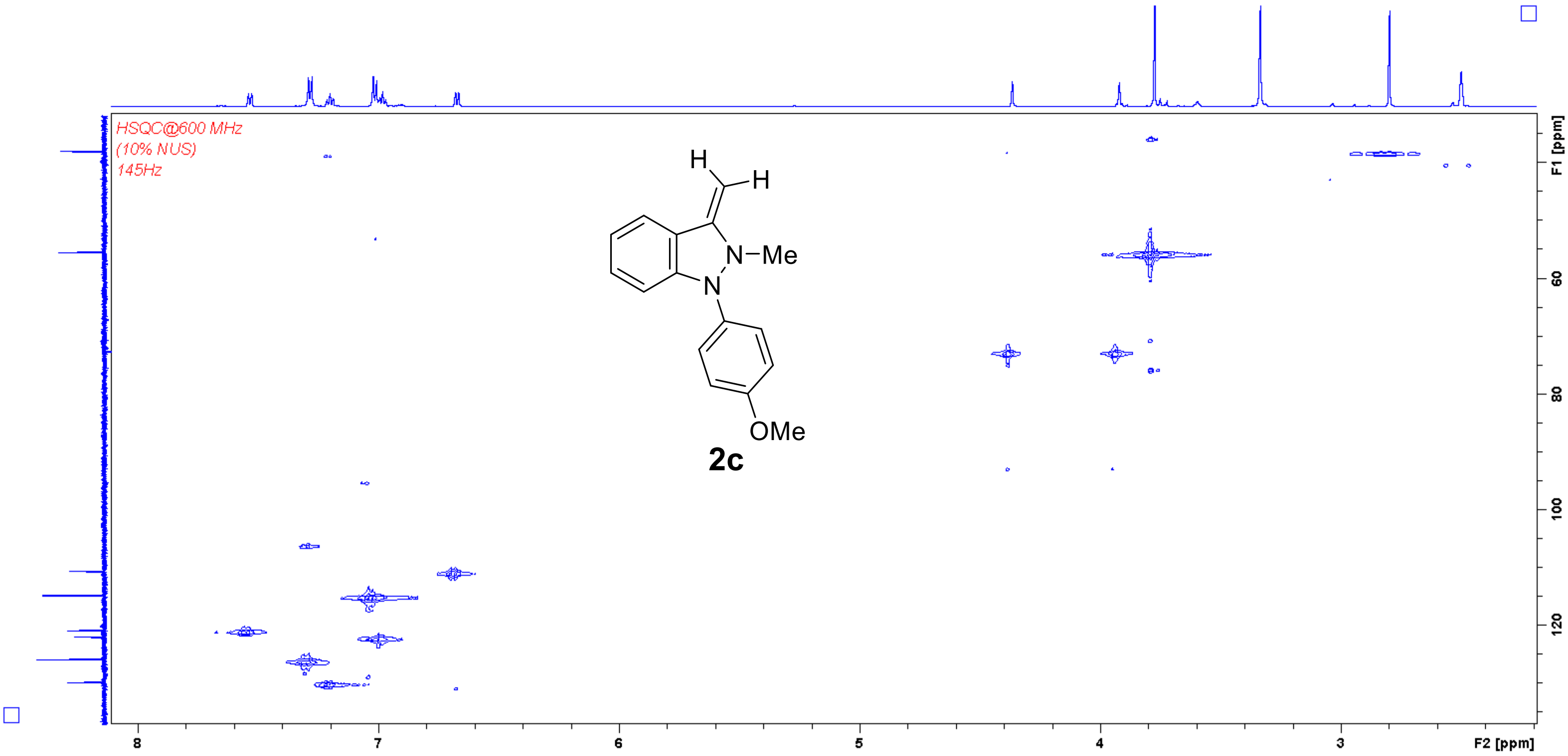
HSQC@600 MHz
(10% NUS)
145Hz
H
H
N−Me
N
OMe
2c
F1 [ppm]
F2 [ppm]

HMBC

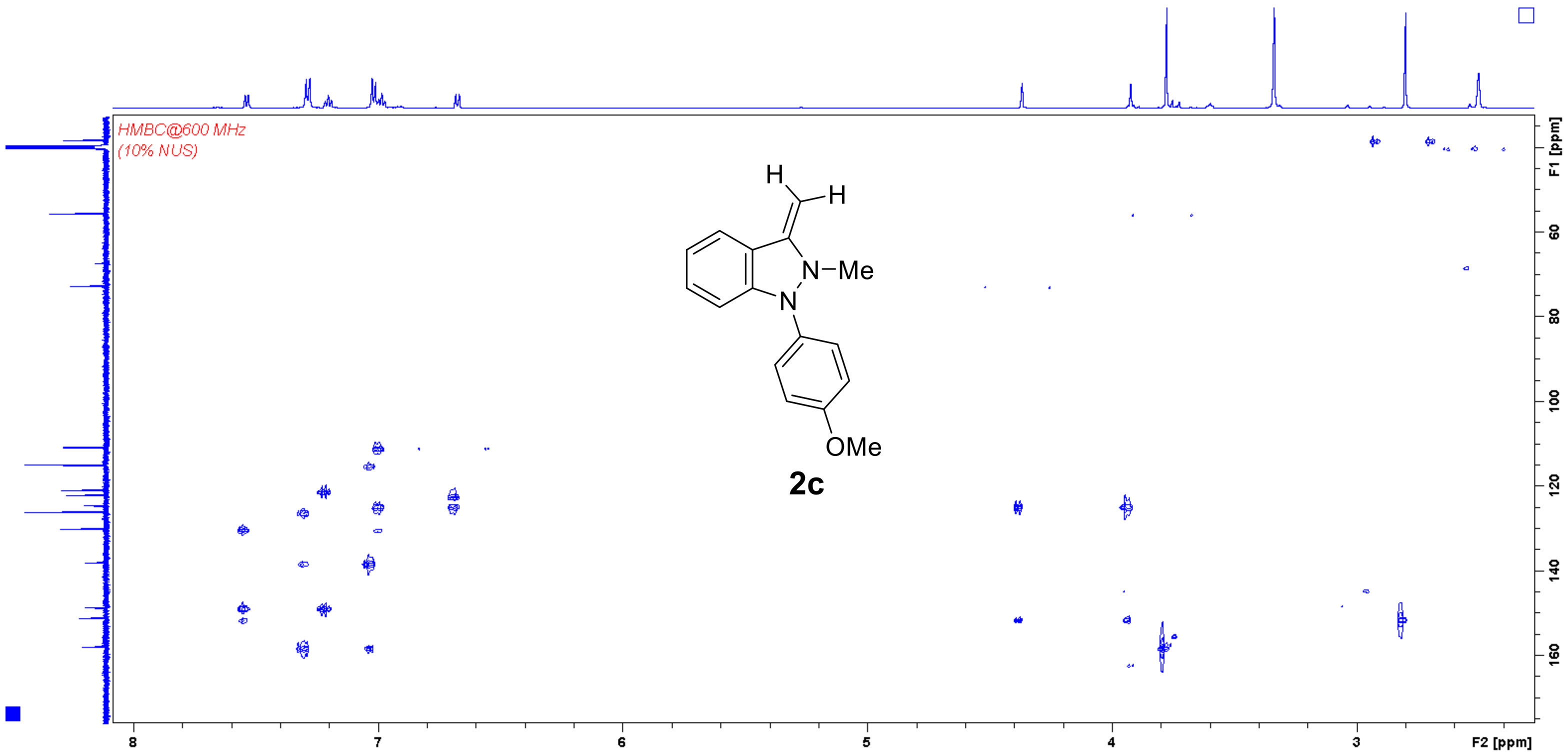

2c

Synthesis of 2,3-dihydro-1,2-dimethyl-3-methylene-4-methoxyl-1*H*-indazole **2d**

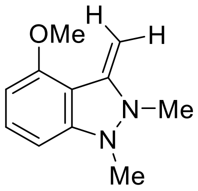

Followed by general procedure of NHO synthesis, the reaction suspension was stirred at room temperature for 72 h. **2d** was obtained as yellow-orange oil (29 mg, 97%).

^{1}H-NMR (DMSO-d$_6$, 600 MHz): 7.19 (t, J = 8.1 Hz, 1H, Ar-H), 6.55 (d, J = 8.1 Hz, 1H, Ar-H), 6.53 (d, J = 8.1 Hz, 1H, Ar-H), 4.51 (s, 1H, CH$_2$), 3.86 (s, 1H, CH$_2$), 3.81 (s, 3H, OMe), 2.90 (s, 3H, N-Me), 2.80 (s, 3H, N-Me) ppm.

^{13}C{^{1}H}-NMR (DMSO-d$_6$, 150 MHz): 155.6 (o, Ar-C), 152.2 (o, Ar-C), 150.8 (o, Ar-C), 130.6 (+, Ar-C), 112.0 (o, Ar-C), 103.5 (+, Ar-C), 76.2 (-, CH$_2$), 55.3 (+, OMe), 40.1 (+, N-Me) 37.7 (+, N-Me) ppm.

IR (ATR): $\tilde{\nu}$ = 1626 (conjugated C=C), 1262 (indazole ring), 504 (C-O-C deformation) cm^{-1}.

^{1}H-NMR

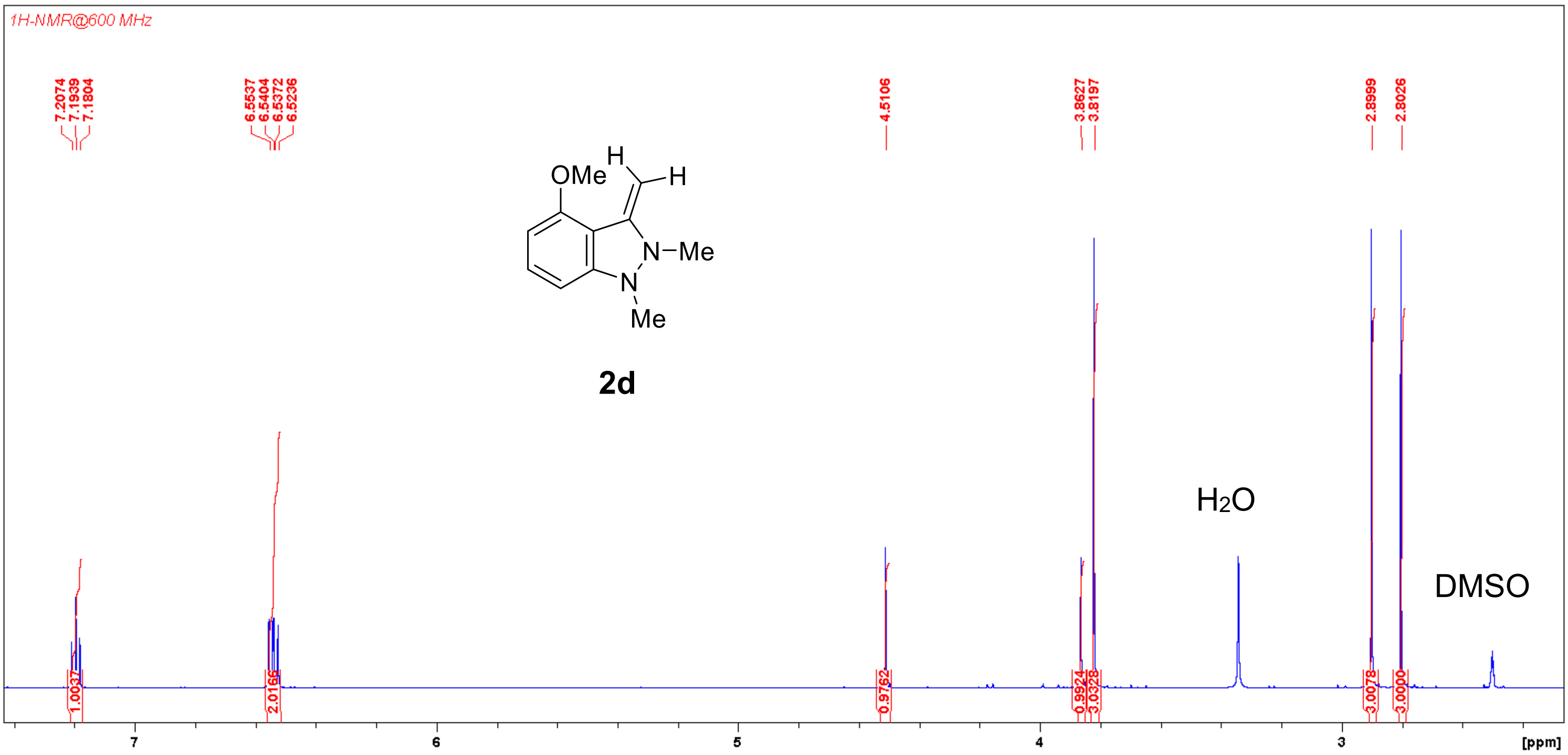

1H-NMR@600 MHz
7.2074
7.1939
7.1804
6.5537
6.5404
6.5372
6.5236
4.5106
3.8627
3.8197
2.8999
2.8026
OMe
H
H
N−Me
N
Me
2d
H2O
DMSO
1.0037
2.0166
0.9762
0.9924
3.0328
3.0078
3.0000
7
6
5
4
3
[ppm]

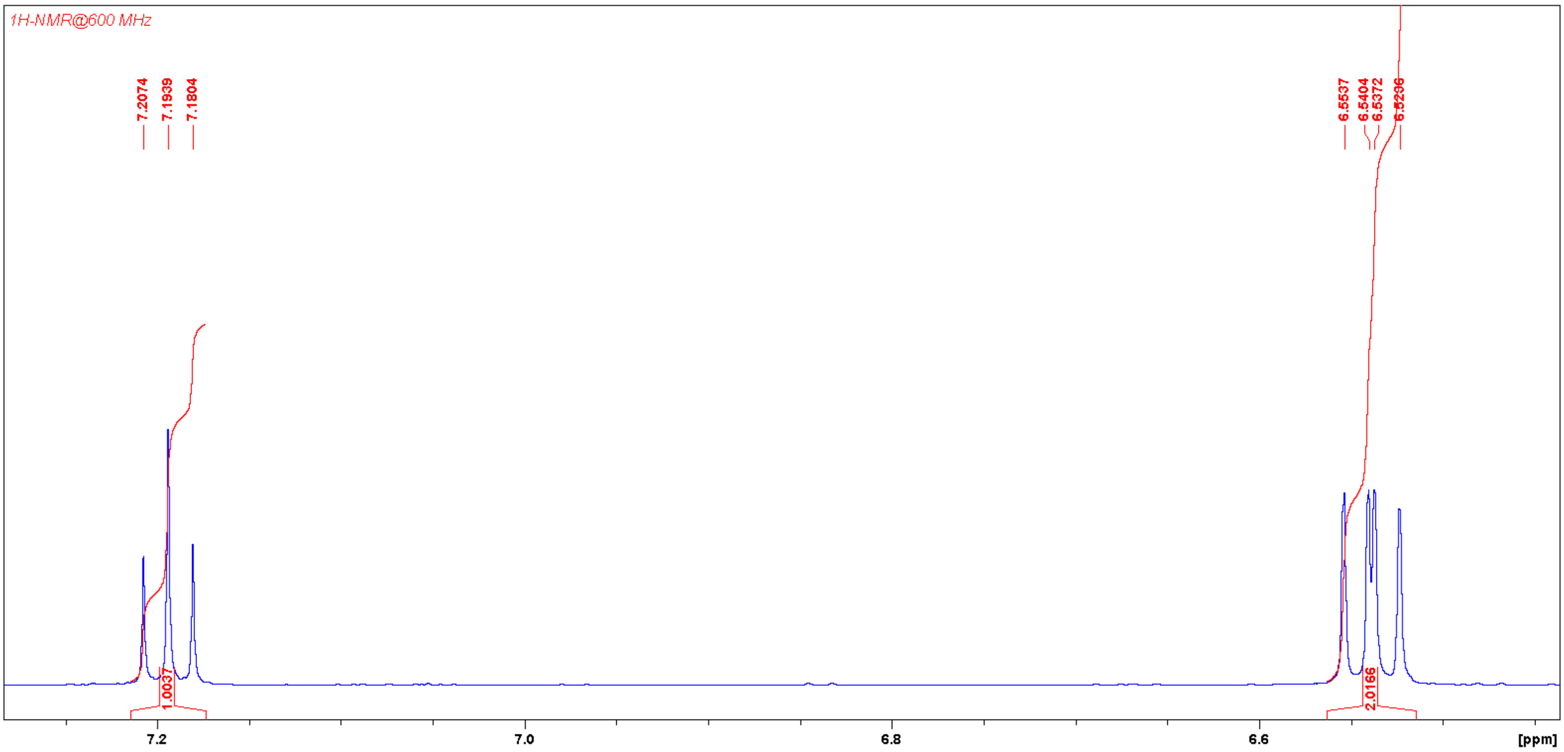

1H-NMR@600 MHz
7.2074
7.1939
7.1804
6.5537
6.5404
6.5372
6.5236
1.0037
2.0166
7.2
7.0
6.8
6.6
[ppm]

$^{13}C\{^1H\}$-NMR

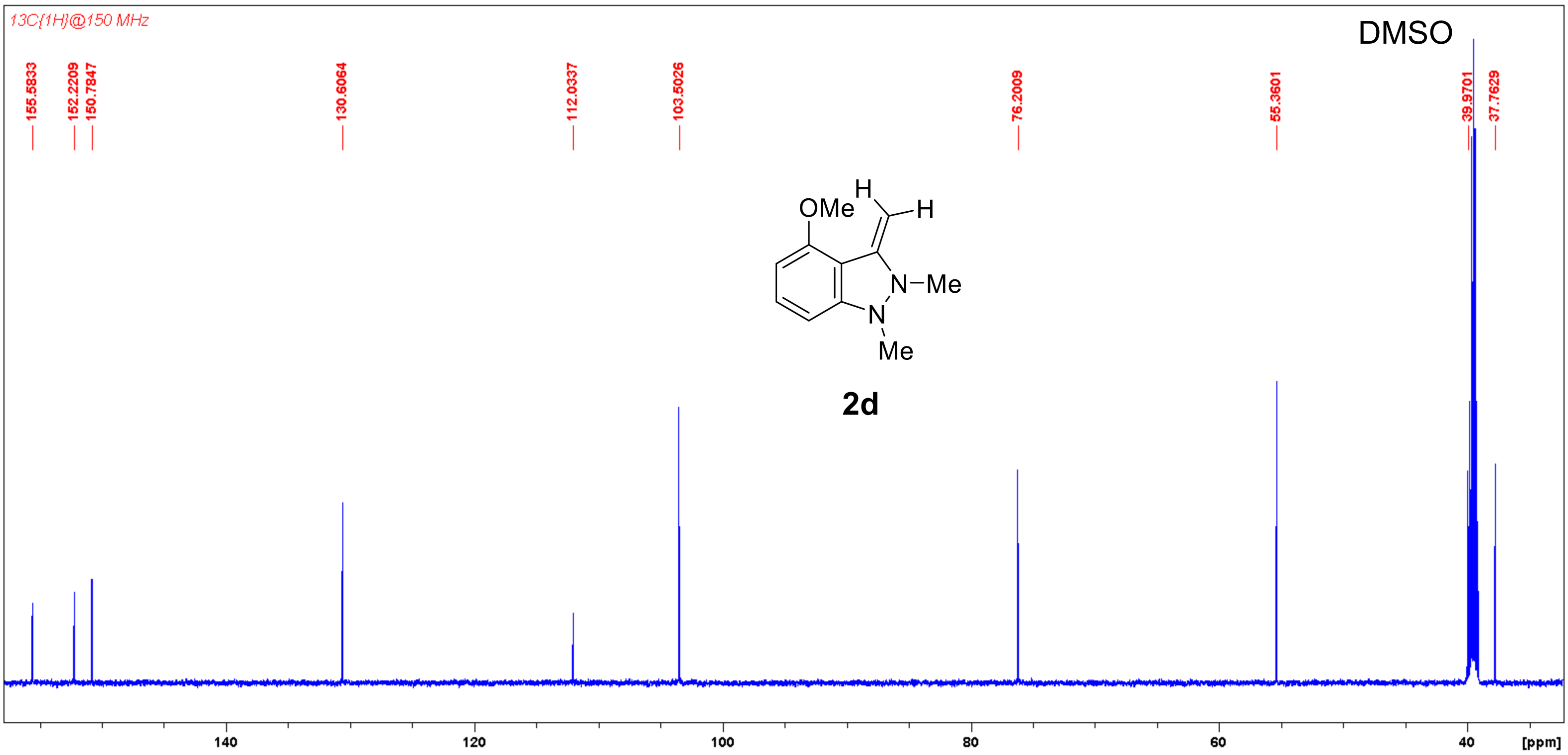
13C{1H}@150 MHz
DMSO
155.5833
152.2209
150.7847
130.6064
112.0337
103.5026
76.2009
55.3601
39.9701
37.7629
OMe
H
H
N−Me
N
Me
2d
140
120
100
80
60
40
[ppm]

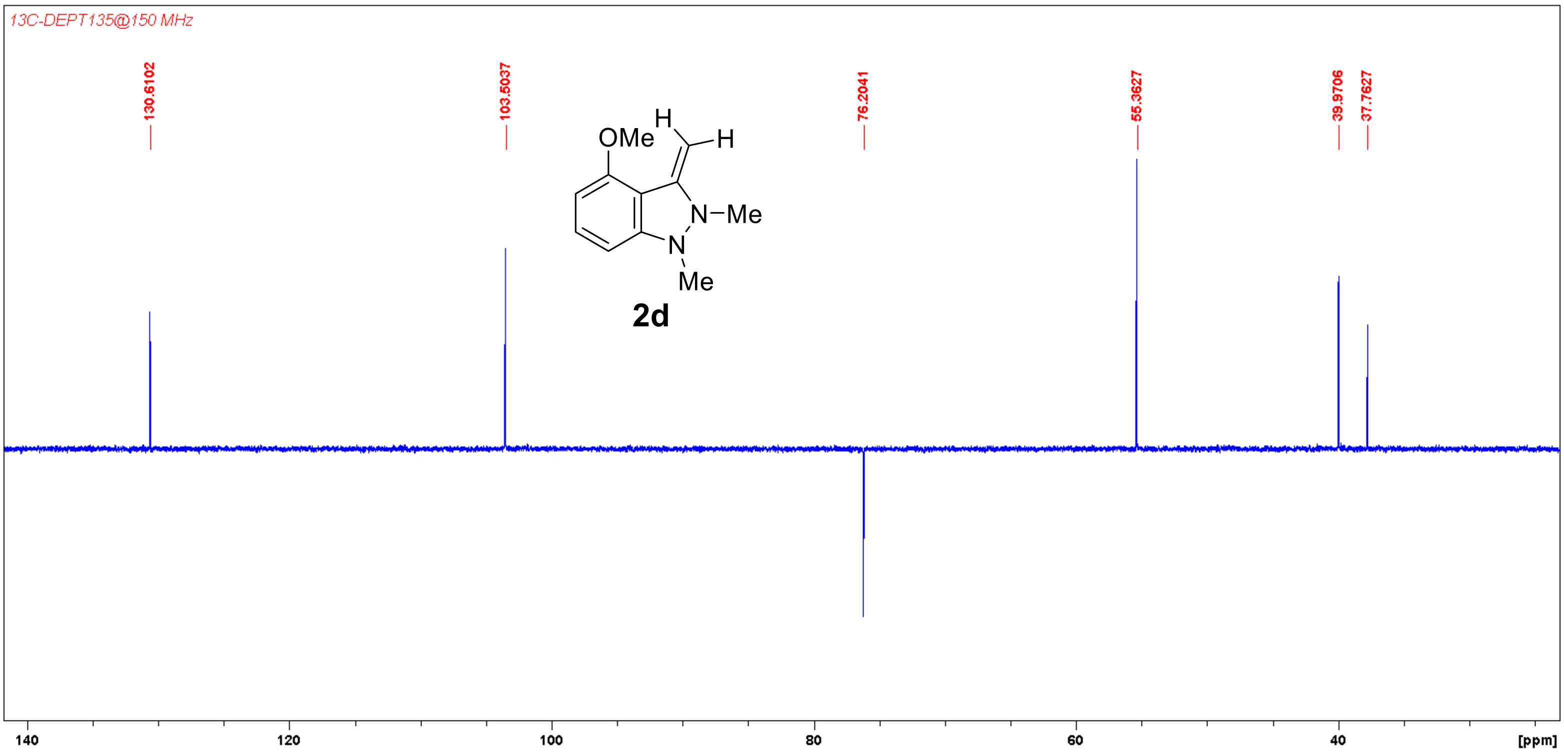
13C-DEPT135@150 MHz
130.6102
103.5037
76.2041
55.3627
39.9706
37.7627
OMe
H
H
N−Me
N
Me
2d
140
120
100
80
60
40
[ppm]

H,H-COSY

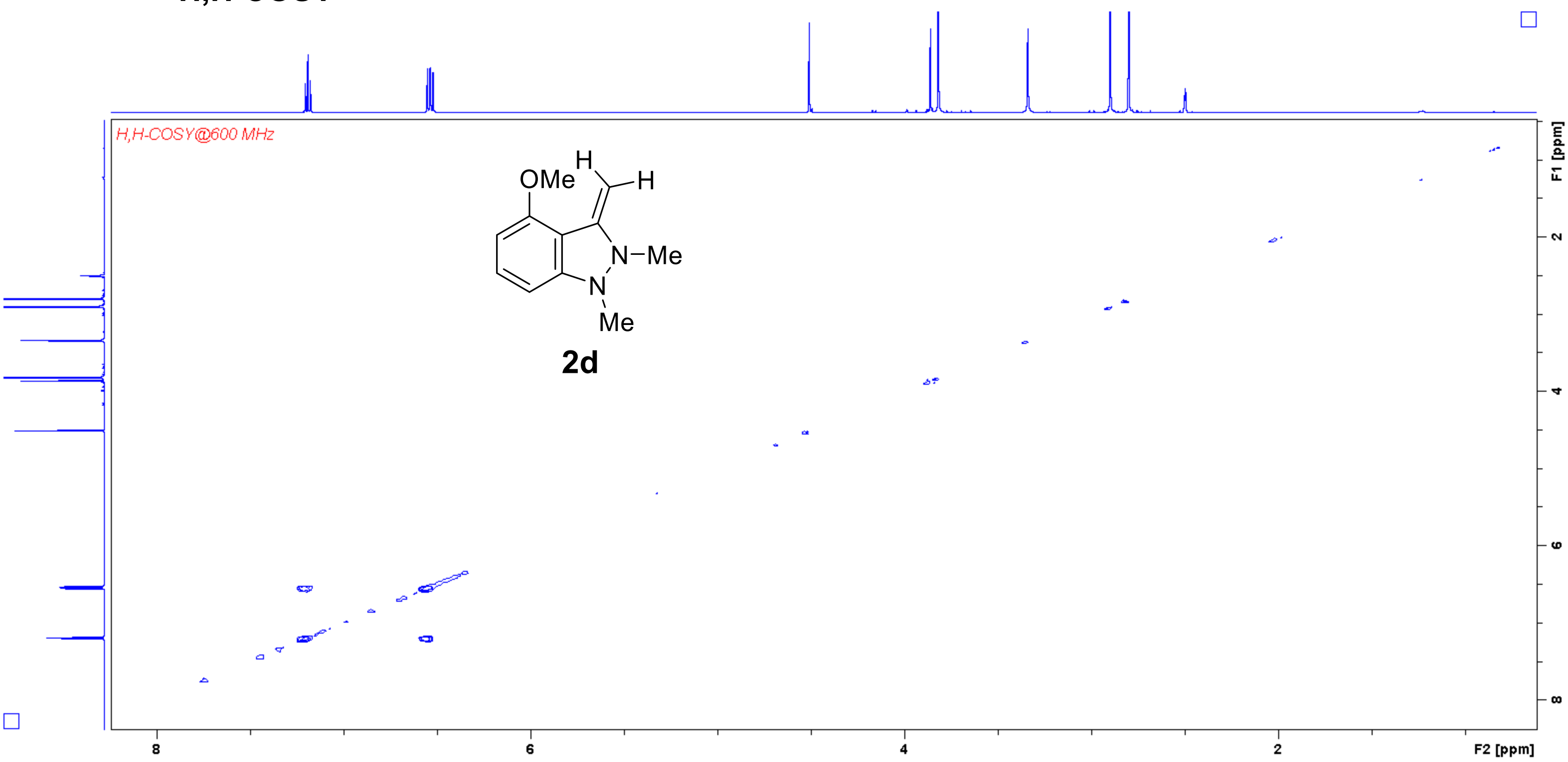
H,H-COSY@600 MHz
OMe
H
H
N−Me
N
Me
2d
F1 [ppm]
F2 [ppm]
8
6
4
2

HSQC

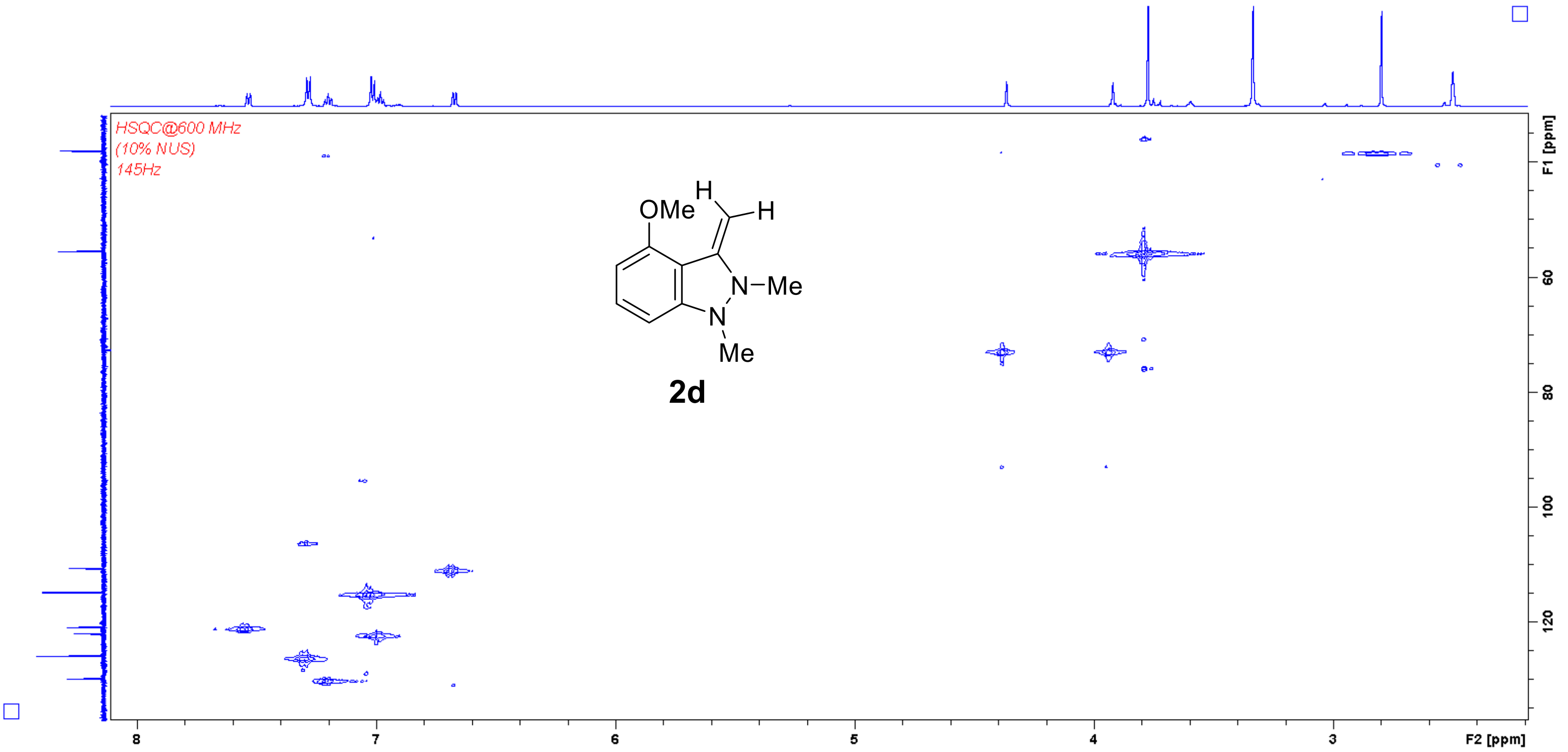
HSQC@600 MHz
(10% NUS)
145Hz
OMe
H
H
N−Me
N
Me
2d
F1 [ppm]
F2 [ppm]
60
80
100
120
8
7
6
5
4
3

HMBC

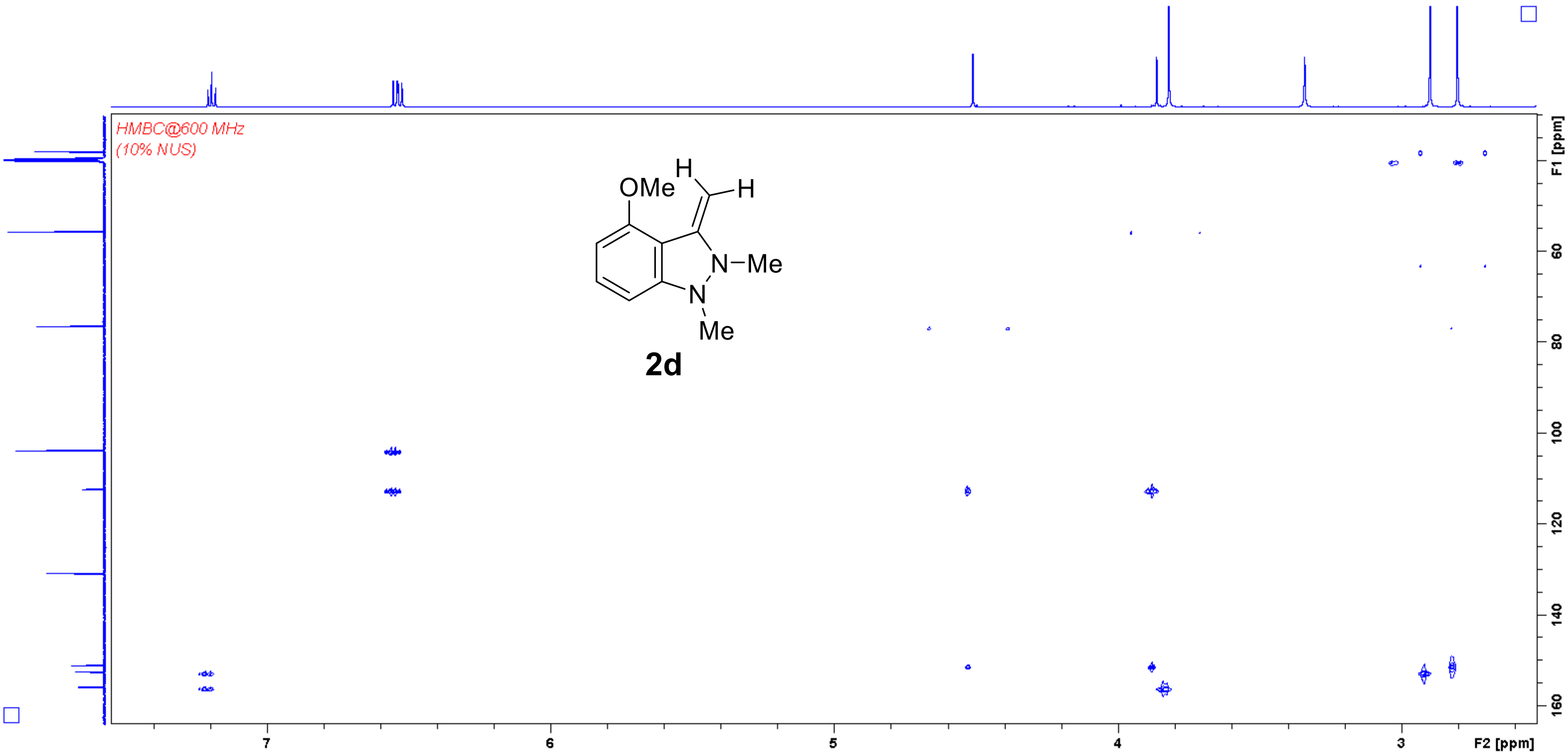
HMBC@600 MHz
(10% NUS)
OMe
H
H
N−Me
N
Me
2d
F1 [ppm]
60
80
100
120
140
160
7
6
5
4
3
F2 [ppm]

Synthesis of 2,4-dimethyl-3-methylene-1,5-diphenyl-2,3-dihydro-1*H*-pyrazole **4a**

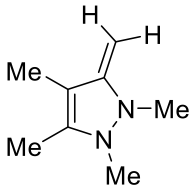

Followed by general procedure of NHO synthesis, **4a** was obtained as yellow-orange solution in THF. Removal of the solvents leads to decompotion.

NMR sample preparation: KHMDS solution in THF (1 Eq, 1.0 M, 0.376 mmol, 0.376 mL) was added in an oven-dried Schlenk-flask under nitrogen atmosphere. The solvent was evaporated in vacuum. 1,2,3,4,5-Pentamethyl-1*H*-pyrazolium iodide (1 Eq, 100 mg, 0.376 mmol) and 1.5 mL THF-d_8 was added and the mixture was stirred magnetically for 2 h at room temperature. NMR sample was obtained via filtration using a syringe and syringe filter.

^{1}H-NMR (THF-d_8, 600 MHz): 3.19 (d, J = 4.6 Hz, 2H, CH_2), 2.68 (s, 3H, N-Me), 2.60 (s, 3H, N-Me), 1.74 (s, 3H, Me), 1.55 (s, 3H, Me) ppm.

^{13}C{^{1}H}-NMR (THF-d_8, 150 MHz): 158.8 (o, Ar-C), 144.5 (o, Ar-C), 108.7 (o, Ar-C), 64.1 (-, CH_2), 38.2 (+, N-Me), 37.3 (+, N-Me), 9.4 (+, Me), 7.7 (+, Me) ppm.

^{1}H-NMR

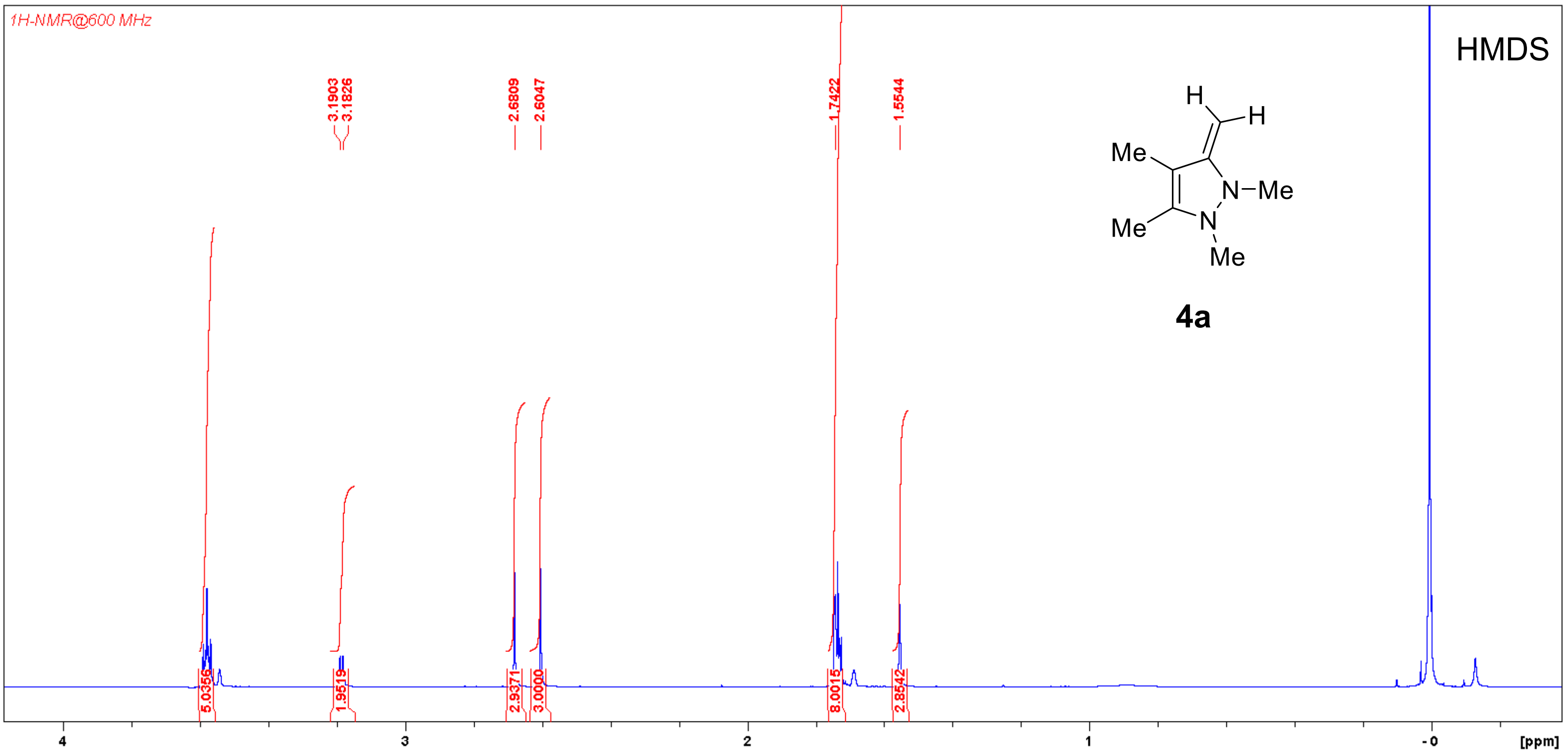

^{13}C{^{1}H}-NMR

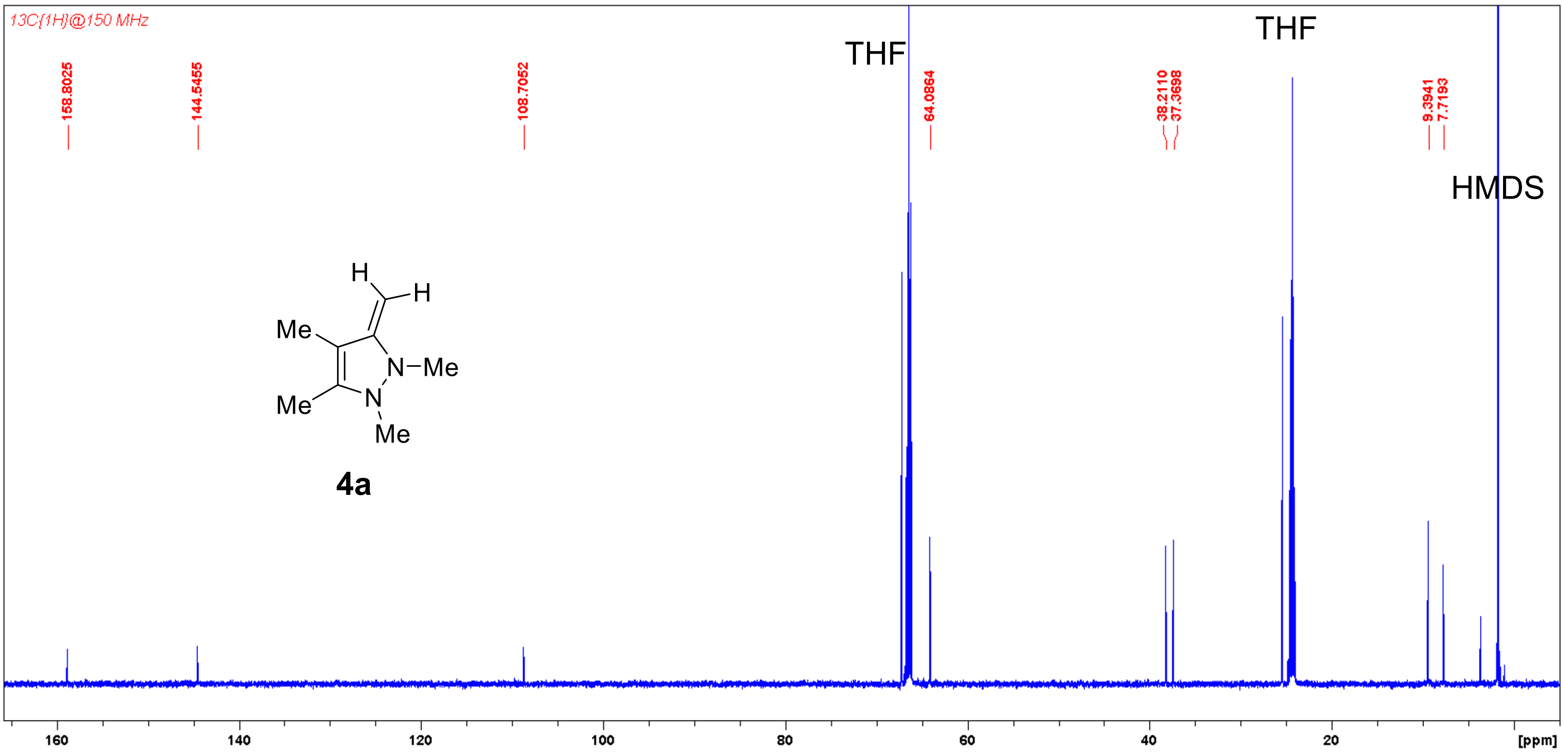

^{13}C-DEPT

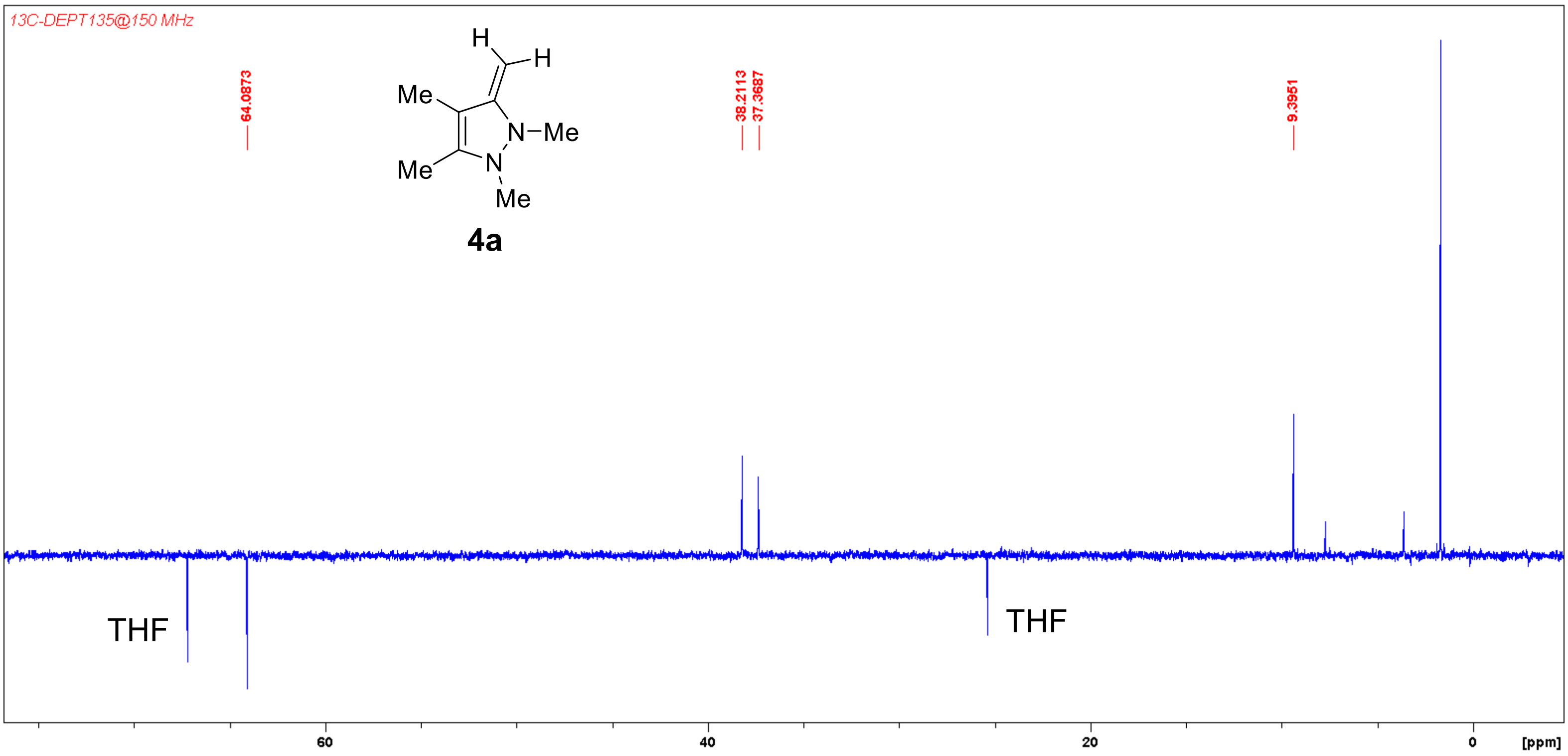

H,H-COSY

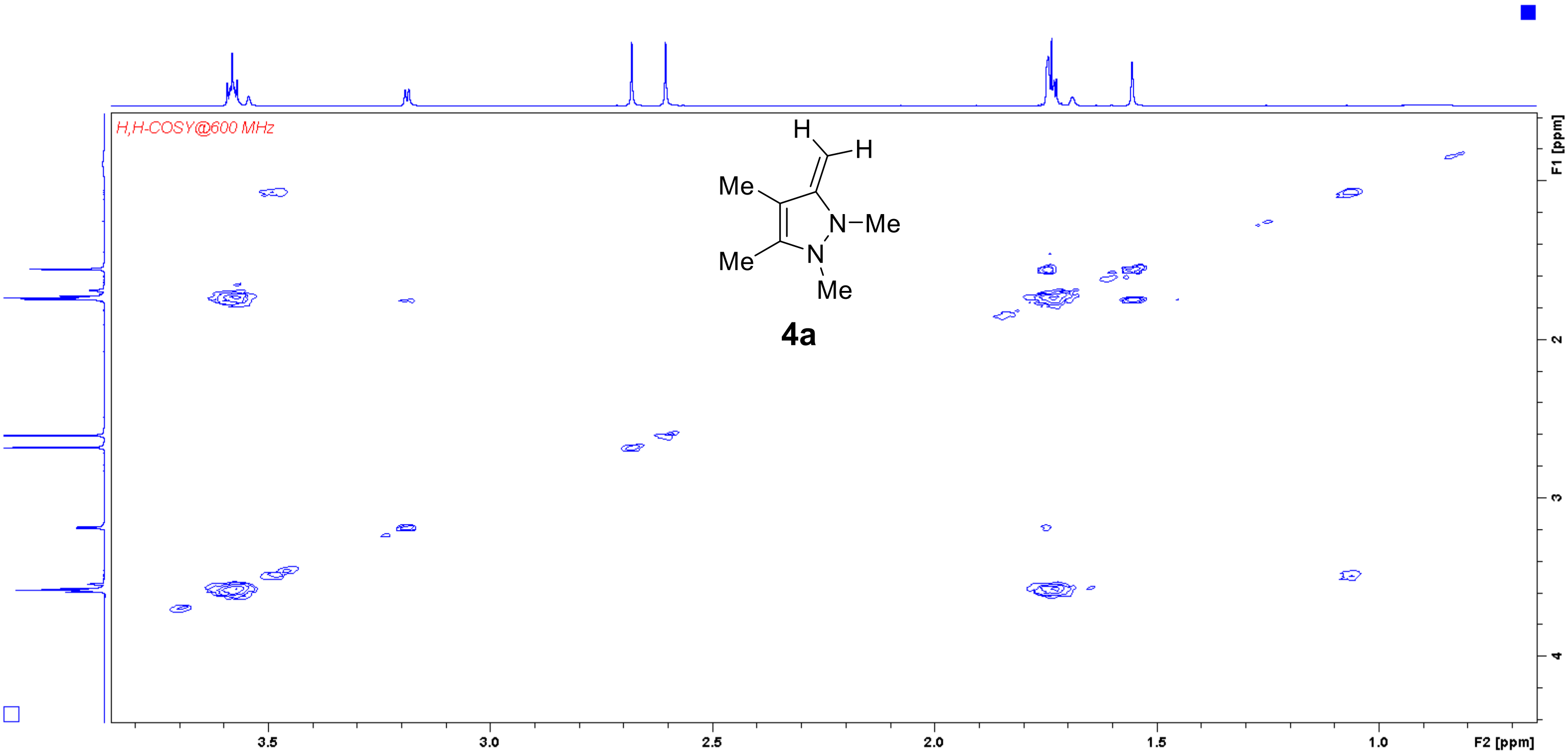

HSQC

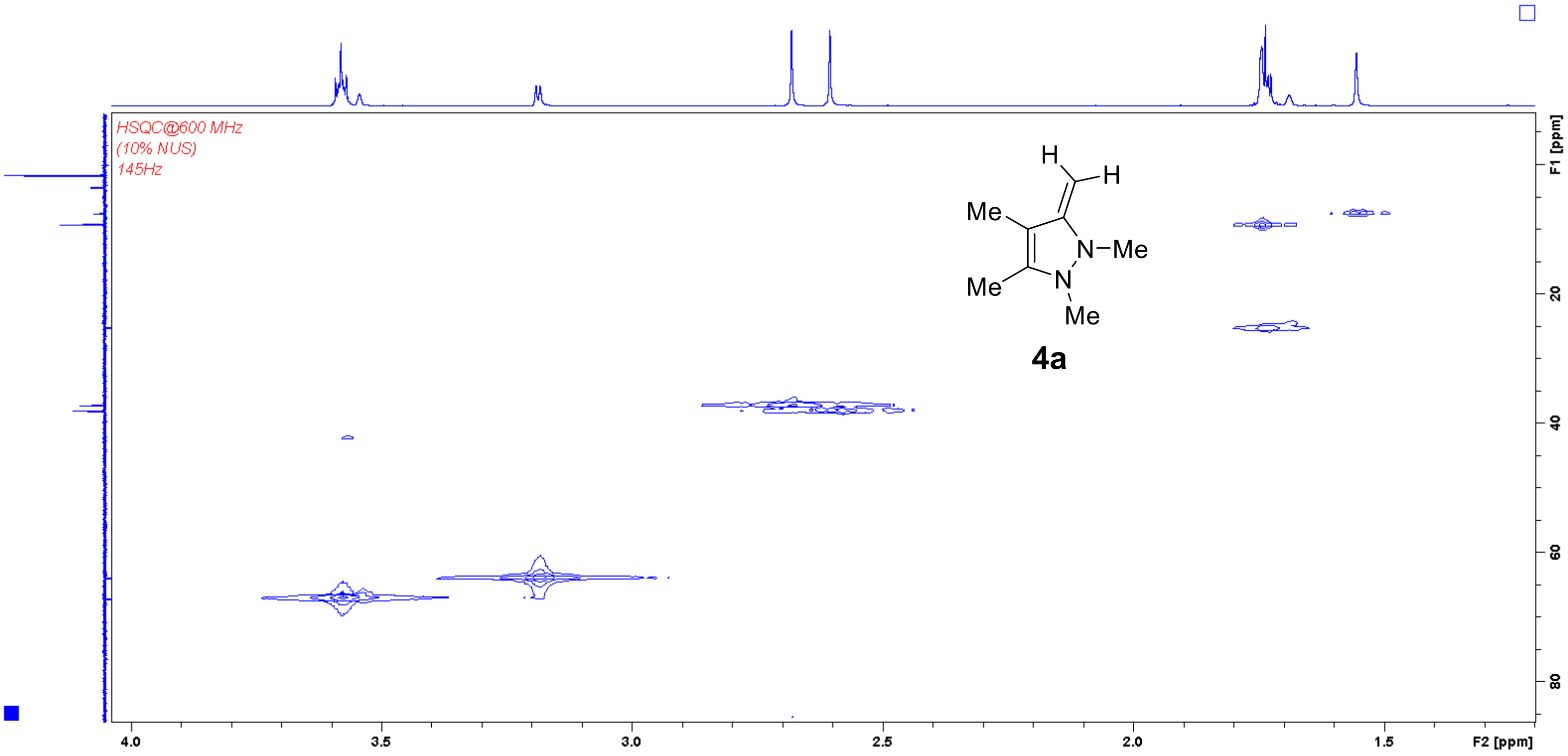
HSQC@600 MHz
(10% NUS)
145Hz
H
H
Me
N−Me
Me
N
Me
4a
F1 [ppm]
20
40
60
80
4.0
3.5
3.0
2.5
2.0
1.5
F2 [ppm]

HMBC

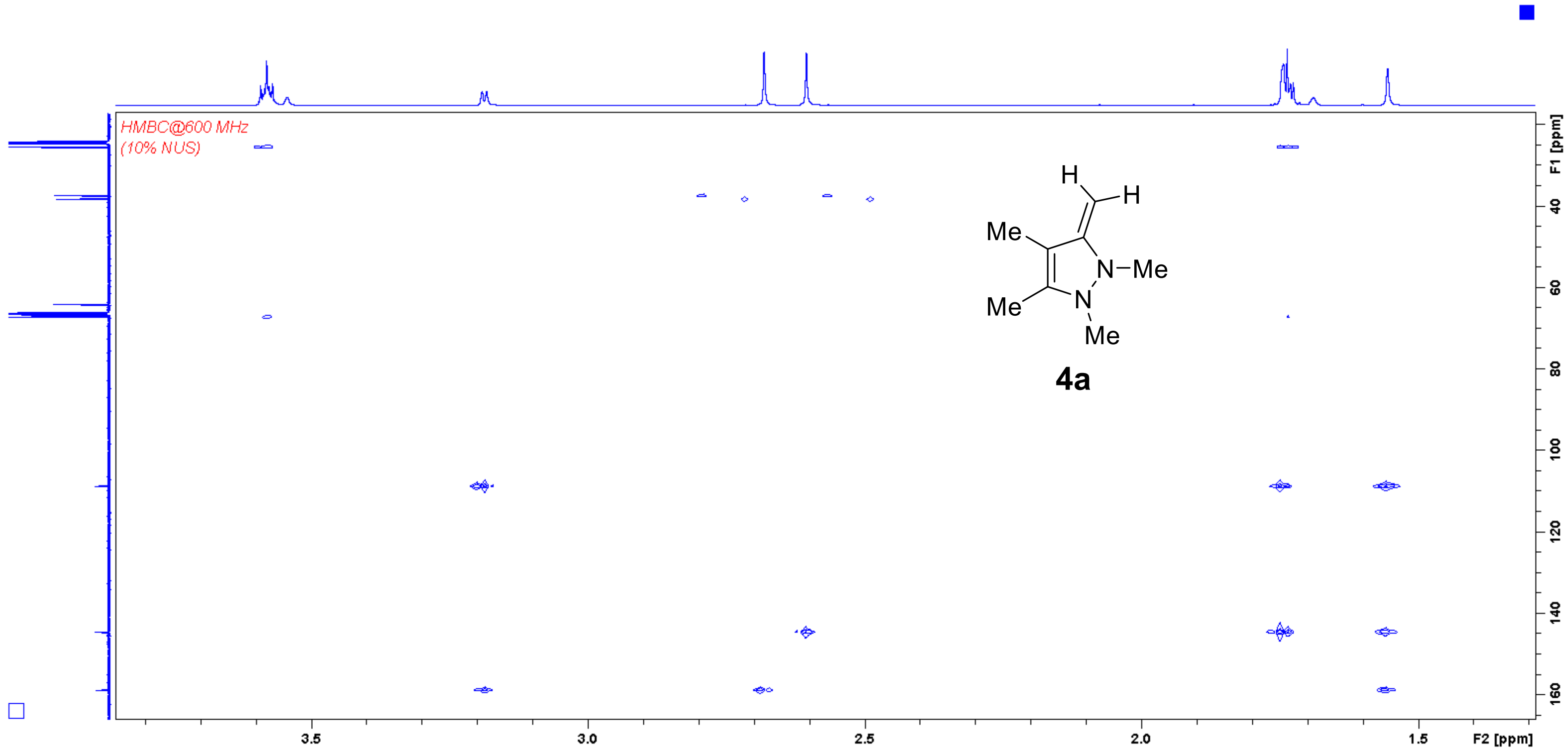
HMBC@600 MHz
(10% NUS)
H
H
Me
N−Me
Me
N
Me
4a
F1 [ppm]
40
60
80
100
120
140
160
3.5
3.0
2.5
2.0
1.5
F2 [ppm]

Synthesis of 2,4-dimethyl-3-methylene-1,5-diphenyl-2,3-dihydro-1*H*-pyrazole **4b**

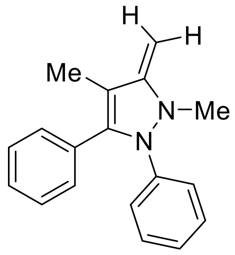

Followed by general procedure of NHO synthesis, **4b** was obtained as yellow solid (320 mg, 95%).

^{1}H-NMR (DMSO-d$_6$, 600 MHz): 7.33-7.26 (m, 4H, Ar-H), 7.25-7.21 (m, 3H, Ar-H), 7.18-7.12 (m, 3H, Ar-H), 3.51 (d, J = 1.1 Hz, 1H, CH$_2$), 3.49 (d, J = 1.1 Hz, 1H, CH$_2$), 2.79 (s, 3H, N-Me), 1.76 (s, 3H, Me) ppm.

^{13}C{^{1}H}-NMR (DMSO-d$_6$, 150 MHz): 157.5 (+, Ar-C), 147.4 (o, Ar-C), 145.3 (o, Ar-C), 130.7 (o, Ar-C), 129.0 (+, Ar-C), 128.8 (+, Ar-C), 128.3 (+, Ar-C), 128.2 (+, Ar-C), 127.1 (+, Ar-C), 126.9 (+, Ar-C), 111.8 (o, Ar-C), 67.0 (-, CH$_2$), 37.9 (+, N-Me), 9.1 (+, Me) ppm.

IR (ATR): $\tilde{\nu}$ = 1612 (conjugated C=C) cm^{-1}.

Melting point: 104-106 °C.

ESI-MS (m/z): calculated for [$C_{18}H_{18}N_2$+H]$^+$: 263.1543, found 263.1543.

^{1}H-NMR (C_6D_6, 600 MHz): 7.20-7.15 (m, 4H, Ar-H), 6.95-6.85 (m, 5H, Ar-H), 6.79-6.75 (m, 1H, Ar-H), 3.94 (d, J = 1.2 Hz, 1H, CH$_2$), 3.85 (d, J = 1.2 Hz, 1H, CH$_2$), 2.79 (s, 3H, N-Me), 1.79 (s, 3H, Me) ppm.

^{13}C{^{1}H}-NMR (C_6D_6, 150 MHz): 158.6 (o, Ar-C), 148.3 (o, Ar-C), 146.4 (o, Ar-C), 132.1 (o, Ar-C), 129.9 (+, Ar-C), 128.8 (+, Ar-C), 128.4 (+, Ar-C), 128.3 (+, Ar-C), 127.0 (+, Ar-C), 126.8 (+, Ar-C), 113.0 (o, 4-C), 67.5 (-, CH$_2$), 38.4 (+, N-Me), 9.5 (+, Me) ppm.

^{1}H-NMR (THF-d$_8$, 600 MHz): 7.27-7.24 (m, 2H, Ar-H), 7.23-7.20 (m, 2H, Ar-H), 7.18-7.12 (m, 1H, Ar-H), 7.05-7.02 (m, 1H, Ar-H), 3.52 (d, J = 1.2 Hz, 1H, CH$_2$), 3.48 (d, J = 1.2 Hz, 1H, CH$_2$), 2.81 (s, 3H, N-Me), 1.77 (s, 3H, Me) ppm.

$^{13}C\{^1H\}$-NMR (THF-d_8, 150 MHz): 158.8 (o, Ar-C), 148.6 (o, Ar-C), 146.8 (o, Ar-C), 132.3 (o, Ar-C), 130.2 (+, Ar-C), 129.1 (+, Ar-C), 128.7 (+, Ar-C), 128.6 (+, Ar-C), 127.4 (+, Ar-C), 127.2 (+, Ar-C), 112.8 (o, 4-C), 66.7 (-, CH_2), 38.3 (+, N-Me), 9.3 (+, Me) ppm.

^1H-NMR (toluene-d_8, 600 MHz): 7.16-7.13 (m, 2H, Ar-H), 7.12-7.09 (m, 2H, Ar-H), 6.94-6.84 (m, 5H, Ar-H), 6.78-6.74 (m, 1H, Ar-H), 3.85 (d, J = 1.2 Hz, 1H, CH_2), 3.76 (d, J = 1.2 Hz, 1H, CH_2), 2.76 (s, 3H, N-Me), 1.77 (s, 3H, Me) ppm.

$^{13}C\{^1H\}$-NMR (toluene-d_8, 150 MHz): 158.5 (o, Ar-C), 148.3 (o, Ar-C), 146.4 (o, Ar-C), 132.1 (o, Ar-C), 129.9 (+, Ar-C), 128.8 (+, Ar-C), 128.3 (+, Ar-C), 128.2 (+, Ar-C), 127.0 (+, Ar-C), 126.8 (+, Ar-C), 112.9 (o, 4-C), 67.5 (-, CH_2), 38.3 (+, N-Me), 9.5 (+, Me) ppm.

^{1}H-NMR

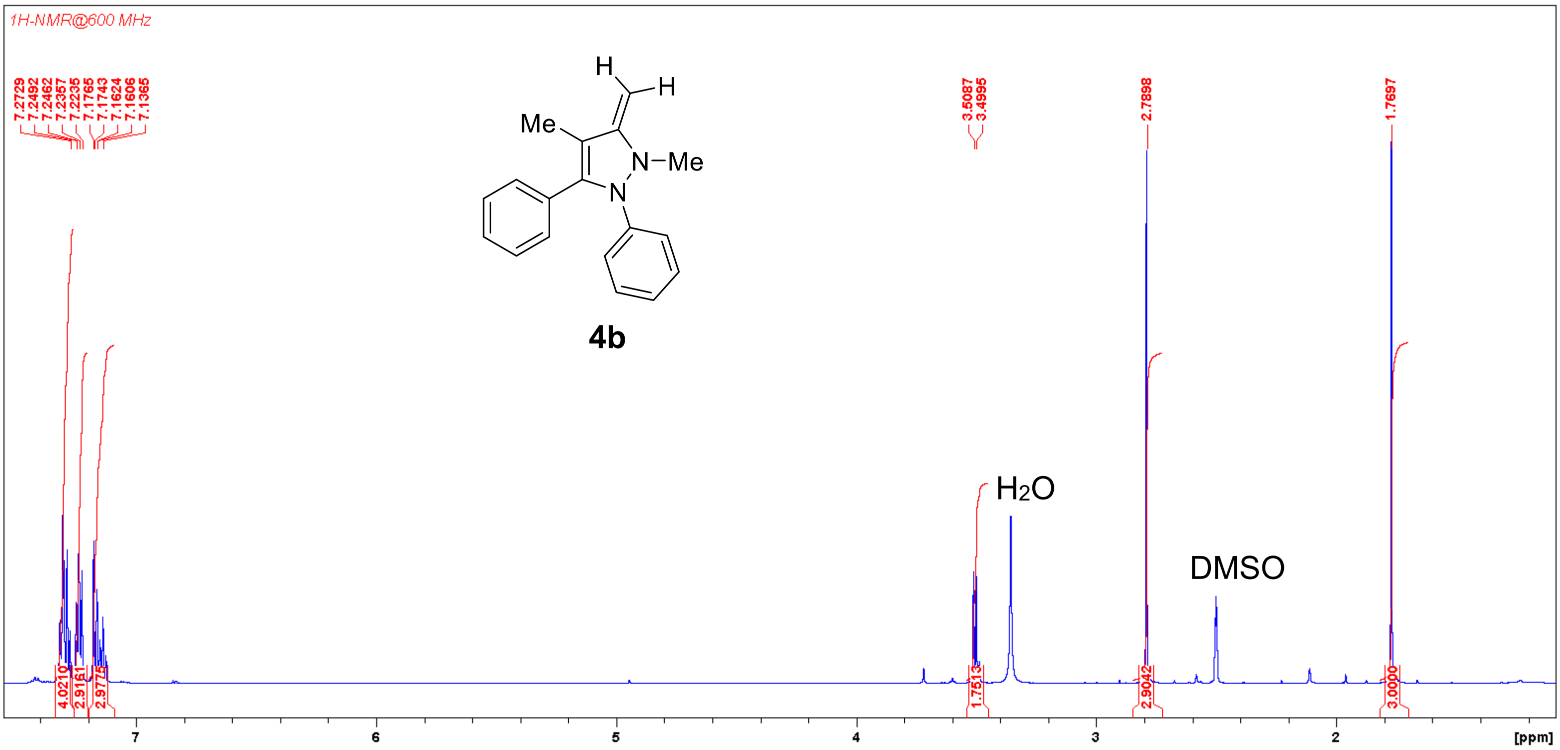
1H-NMR@600 MHz
7.2729
7.2492
7.2462
7.2357
7.2235
7.1765
7.1743
7.1624
7.1606
7.1365
3.5087
3.4995
2.7898
1.7697
H
H
Me
N−Me
N
4b
H2O
DMSO
4.0210
2.9161
2.9775
1.7513
2.9042
3.0000
7
6
5
4
3
2
[ppm]

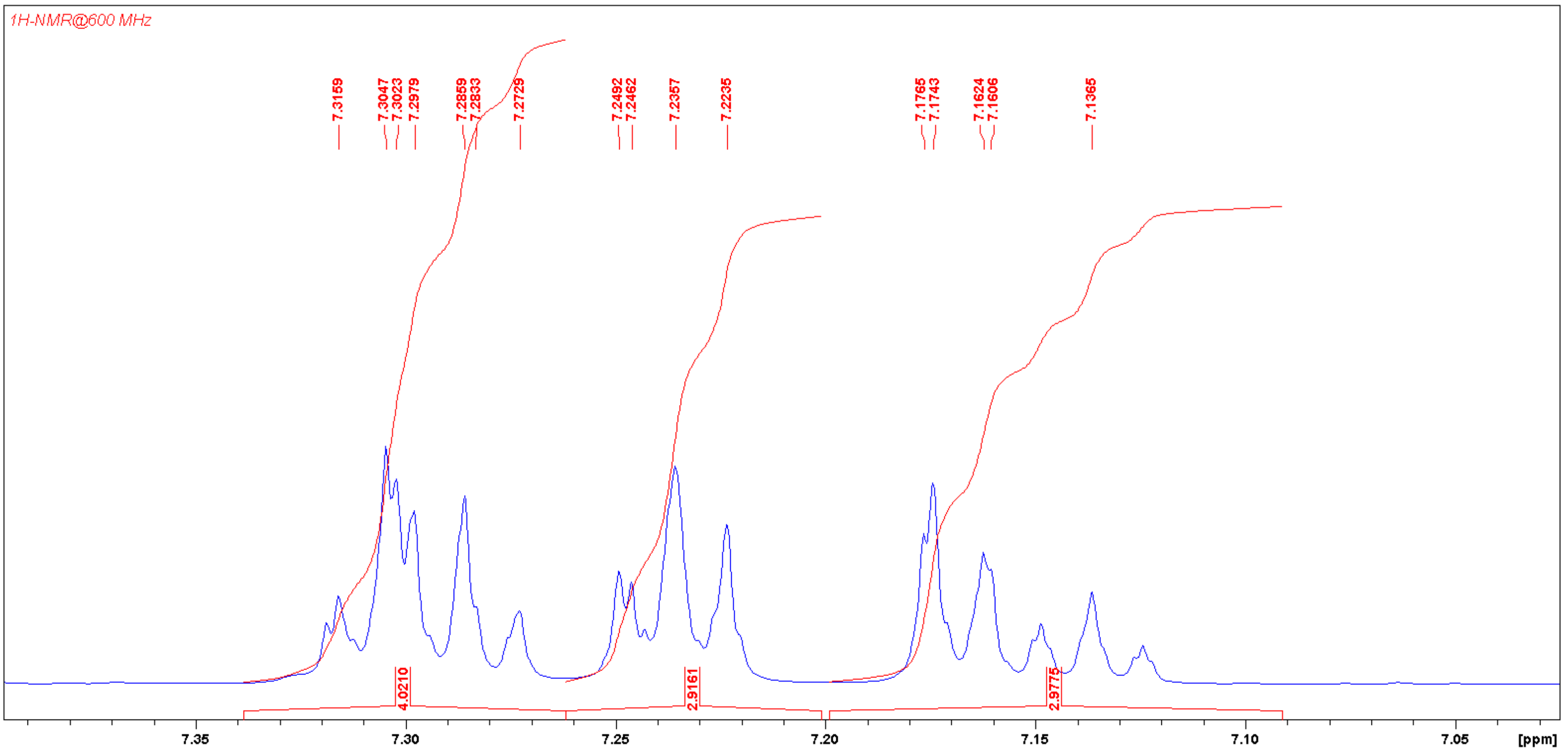
1H-NMR@600 MHz
7.3159
7.3047
7.3023
7.2979
7.2859
7.2833
7.2729
7.2492
7.2462
7.2357
7.2235
7.1765
7.1743
7.1624
7.1606
7.1365
4.0210
2.9161
2.9775
7.35
7.30
7.25
7.20
7.15
7.10
7.05
[ppm]

$^{13}C\{^1H\}$-NMR

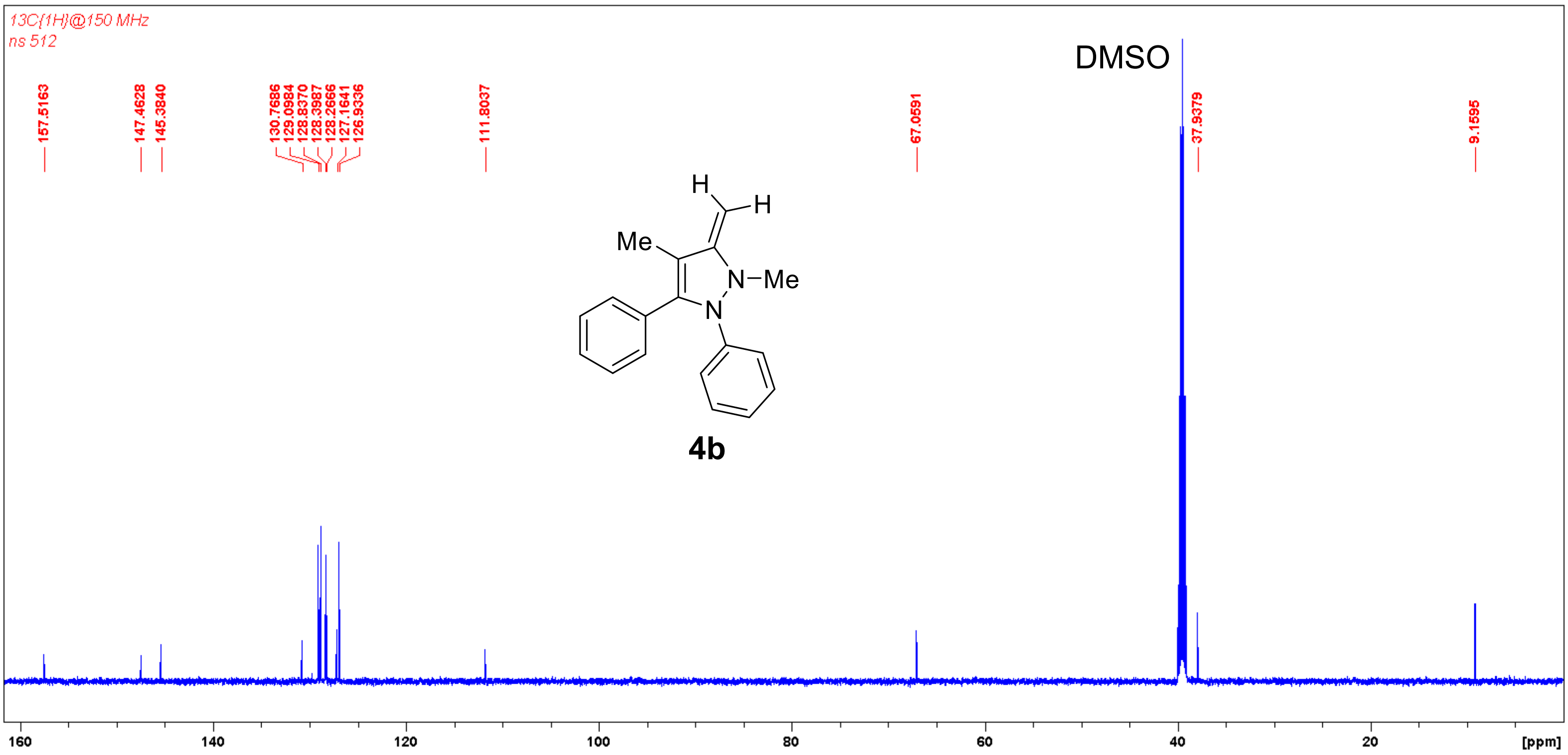

^{13}C-DEPT

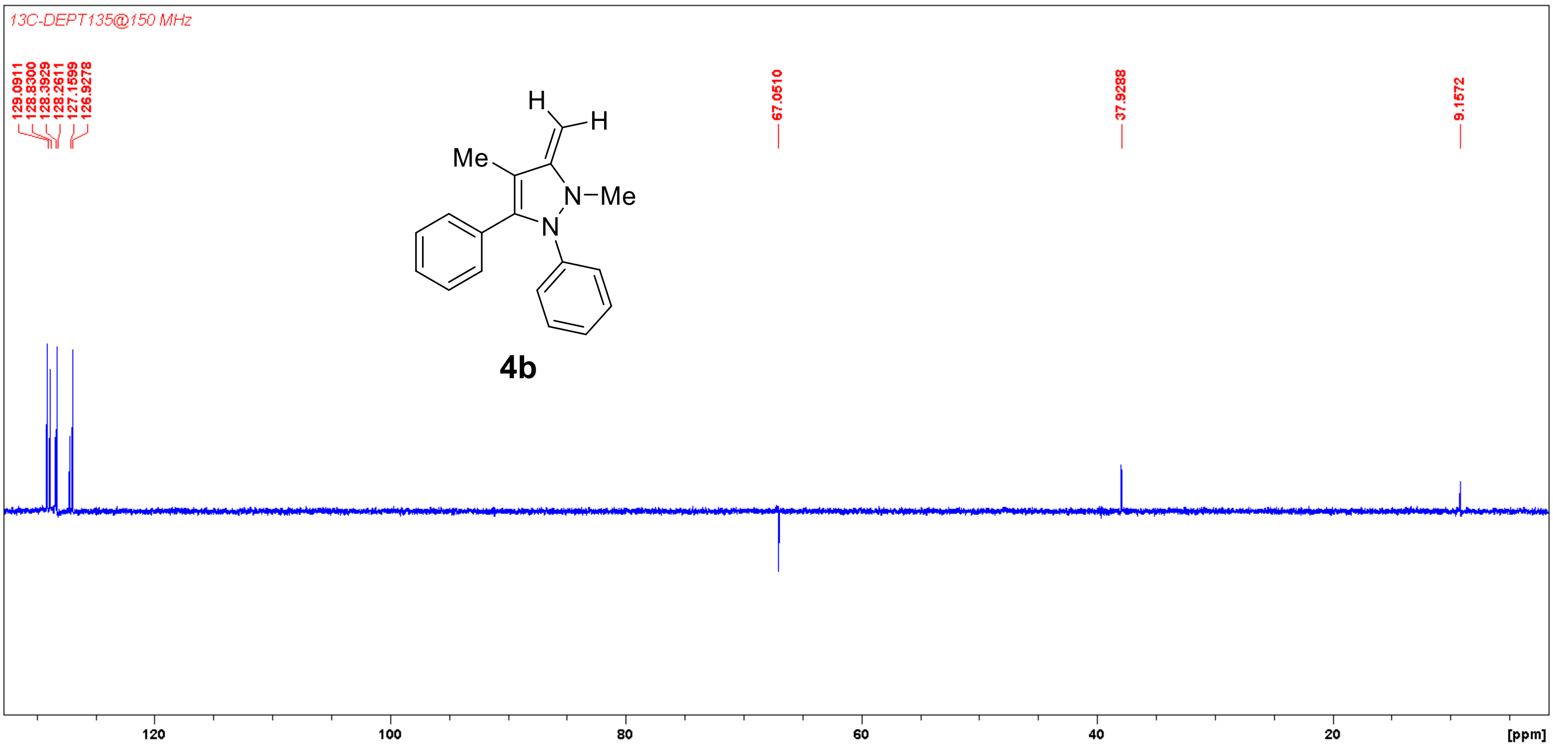

H,H-COSY

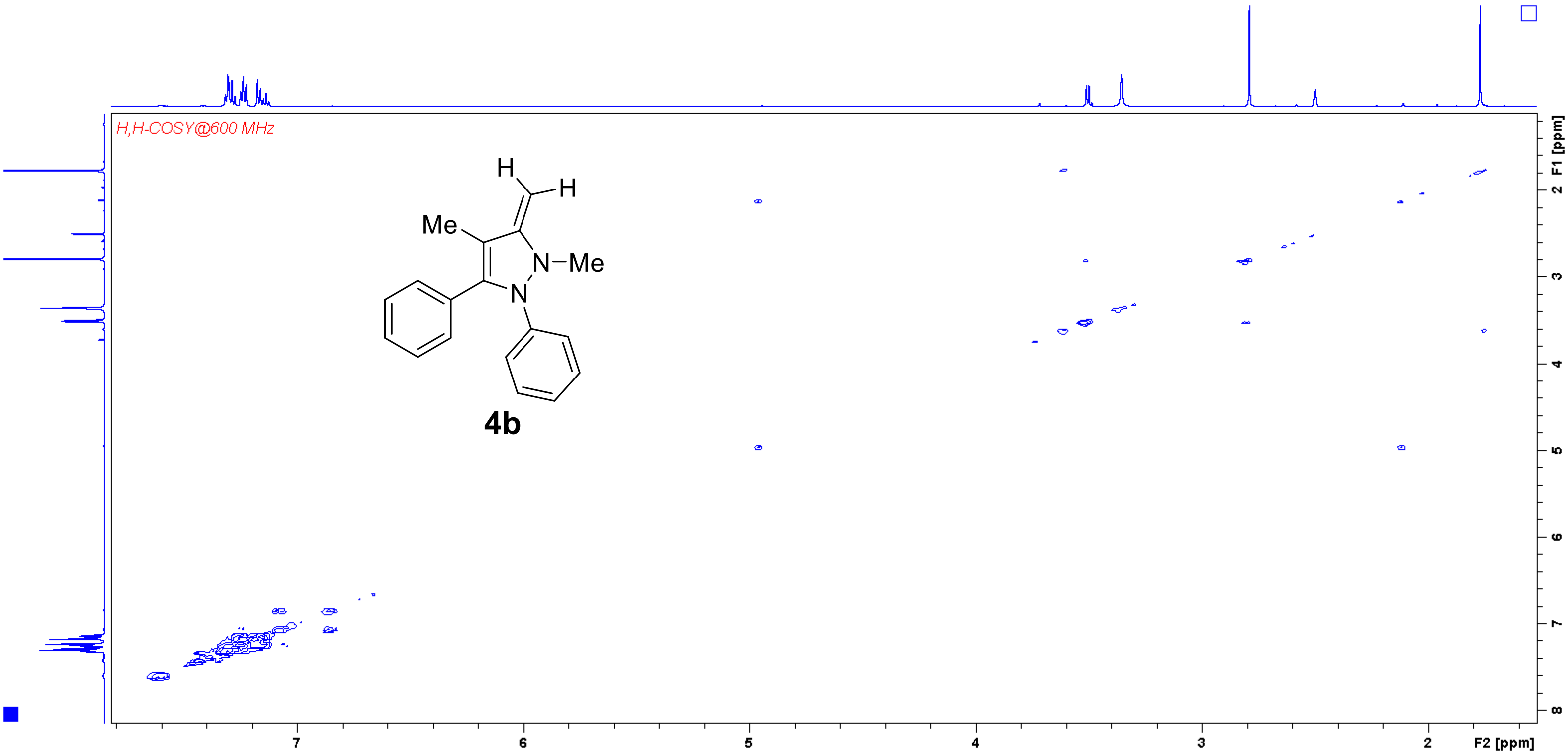
H,H-COSY@600 MHz
H
H
Me
N−Me
N
4b
F1 [ppm]
F2 [ppm]

HMBC

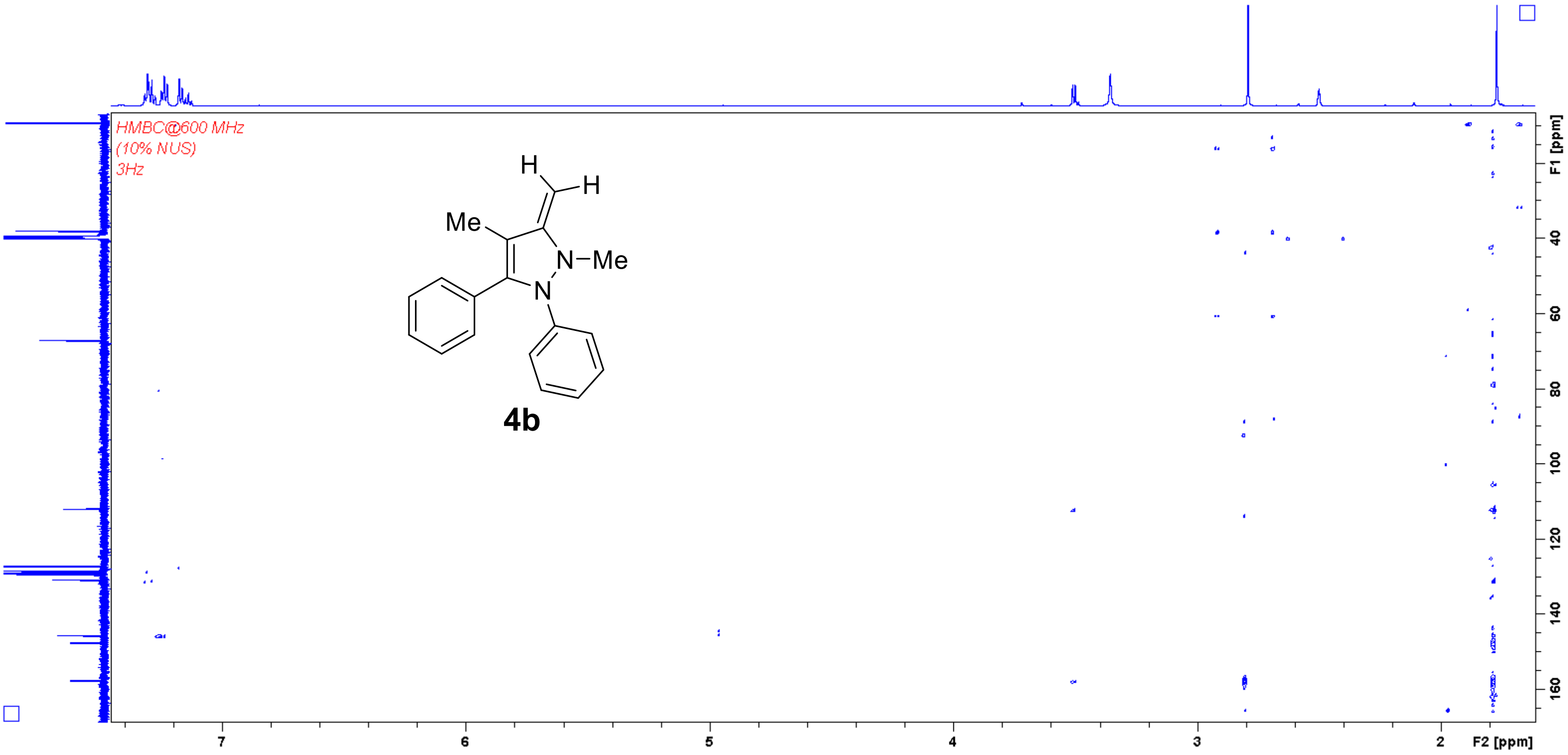
HMBC@600 MHz
(10% NUS)
3Hz
H
H
Me
N−Me
N
4b
F1 [ppm]
F2 [ppm]

HSQC

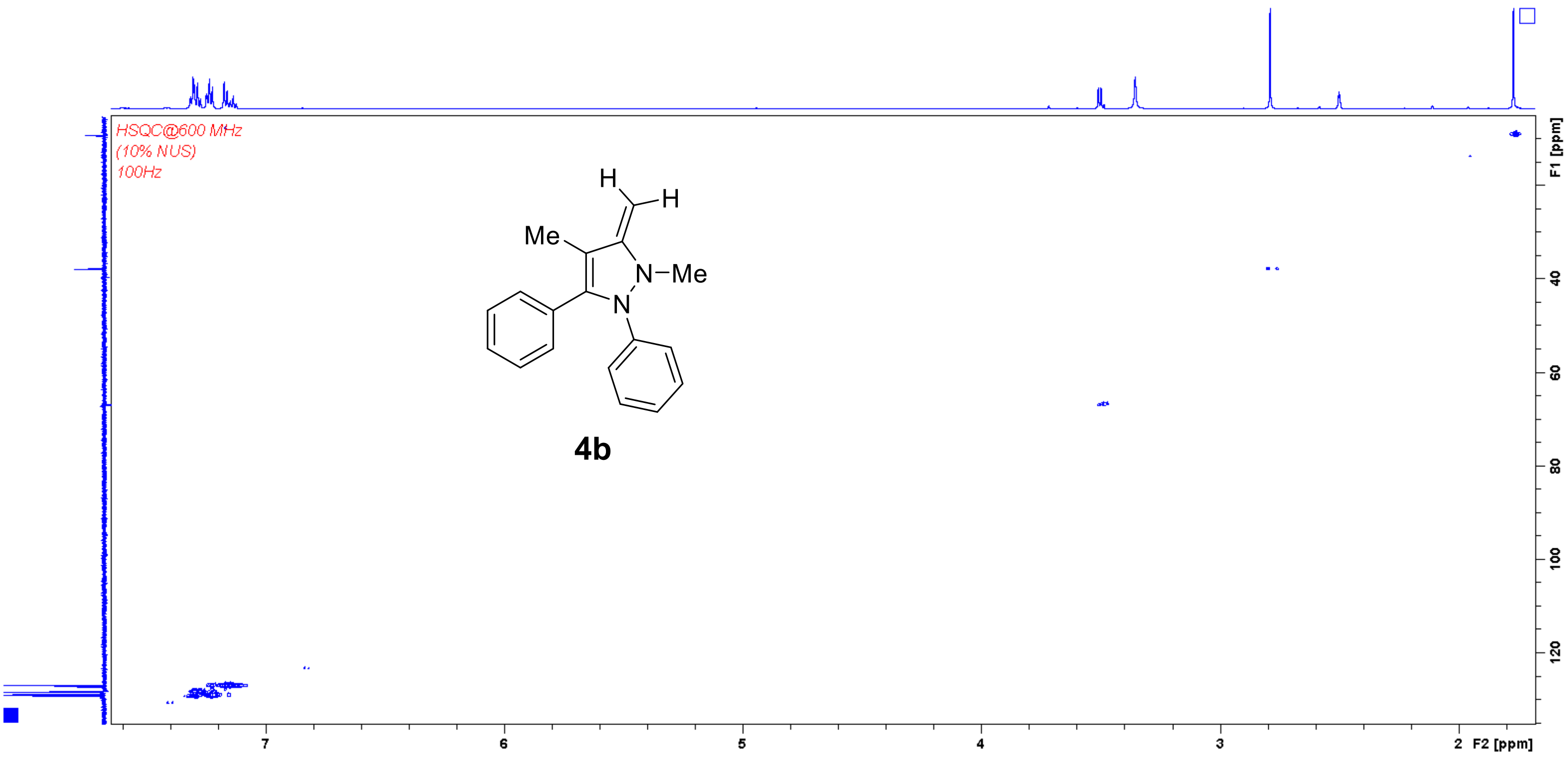

HSQC@600 MHz
(10% NUS)
100Hz
Me
N−Me
N
H
H
4b
F1 [ppm]
F2 [ppm]

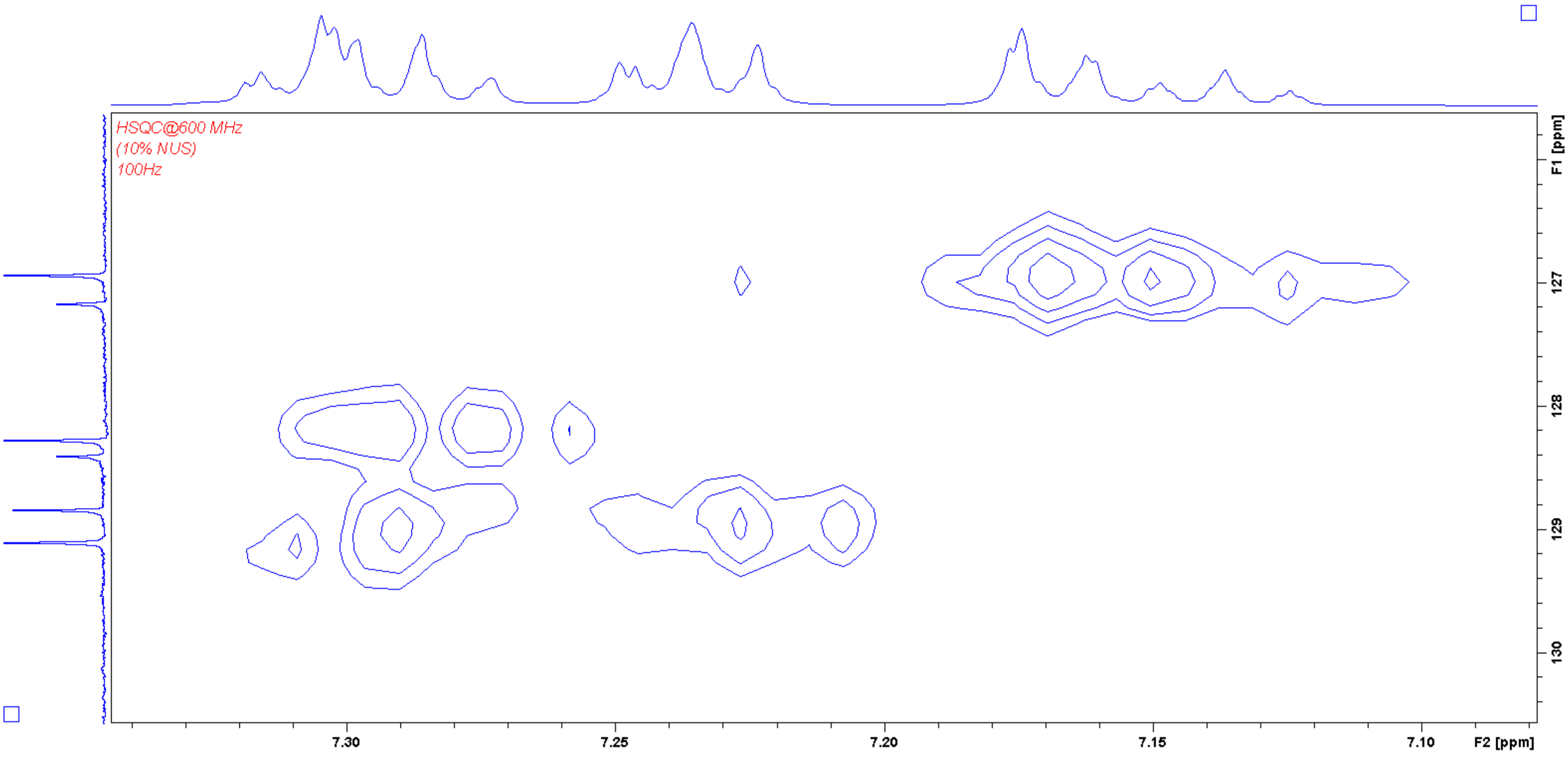

HSQC@600 MHz
(10% NUS)
100Hz
F1 [ppm]
F2 [ppm]

General procedure of NHO adduct synthesis

The NHO solutions in THF is prepared from the corresponding precursor salts (1.0 Eq) followed by the general procedure of NHO synthesis. The NHO solution is cooled to 0 °C under nitrogen atmosphere. Electrophile (1.0 Eq iodine, bromine, carbondisulfide or 2.0 Eq isocyanate) is then added to the solution, which is subsequently stirred at room temperature for 2 h. The reaction mixture is then poured into pentane to form a precipitate, or the precipitate is formed spontaneously, which is then filtered off, washed with ethyl acetate, and dried in a vacuum to give the corresponding adducts.

Synthesis of 3-iodomethyl-1,2-dimethyl-1*H*-indazol-2-ium iodide **9a**

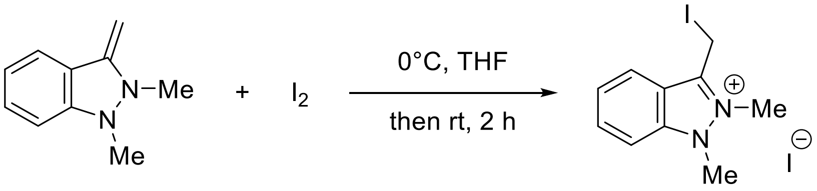

Followed by the general procedure of NHO adduct synthesis, **9a** is prepared from 1,2,3-trimethyl-1H-indazol-2-ium tetrafluoroborate **1a** (1.0 Eq, 100 mg, 0.403 mmol) and iodine (1.0 Eq, 102 mg, 0.403 mmol) as yellow solid (66 mg, 40%).

^{1}H-NMR (DMSO-d$_6$, 600 MHz): 8.20 (d, J = 8.4 Hz, 1H, Ar-H), 8.01 (d, J = 8.4 Hz, 1H, Ar-H), 7.89 (td, J = 8.4, 0.8 Hz, 1H, Ar-H), 7.57 (t, J = 8.4, 0.8 Hz, 1H, Ar-H), 5.23 (s, 2H, CH$_2$), 4.27 (s, 3H, N-Me), 4.17 (s, 3H, N-Me) ppm.

^{13}C{^{1}H}-NMR (DMSO-d$_6$, 150 MHz): 141.7 (o, Ar-C), 139.0 (o, Ar-C), 133.3 (+, Ar-C), 124.7 (+, Ar-C), 121.7 (+, Ar-C), 117.3 (o, Ar-C), 111.1 (+, Ar-C), 34.9 (+, N-Me), 33.7 (+, N-Me), -12.2 (-, CH$_2$) ppm.

ESI-MS (m/z): calculated for $[C_{10}H_{12}N_2I]^+$: 287.0040, found 287.0040.

IR (ATR): $\tilde{\nu}$ = 487 (C-I stretching) cm^{-1}.

Melting point: 158-160 °C, decomposed.

^{1}H-NMR

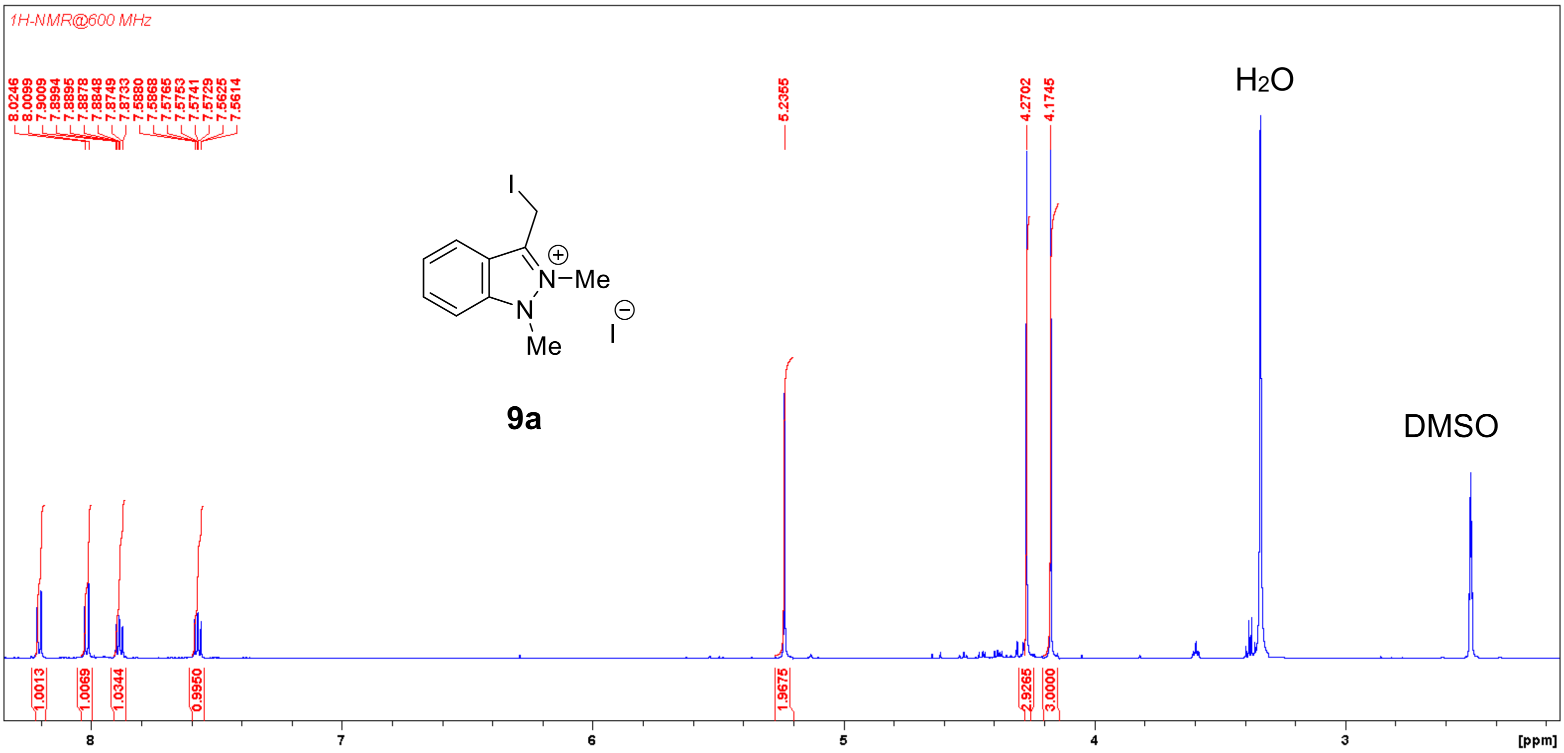
1H-NMR@600 MHz
8.0246
8.0099
7.9009
7.8994
7.8895
7.8878
7.8848
7.8749
7.8733
7.5880
7.5868
7.5765
7.5753
7.5741
7.5729
7.5625
7.5614
5.2355
4.2702
4.1745
H2O
DMSO
I
N−Me
N
Me
I
9a
1.0013
1.0069
1.0344
0.9950
1.9675
2.9265
3.0000
8
7
6
5
4
3
[ppm]

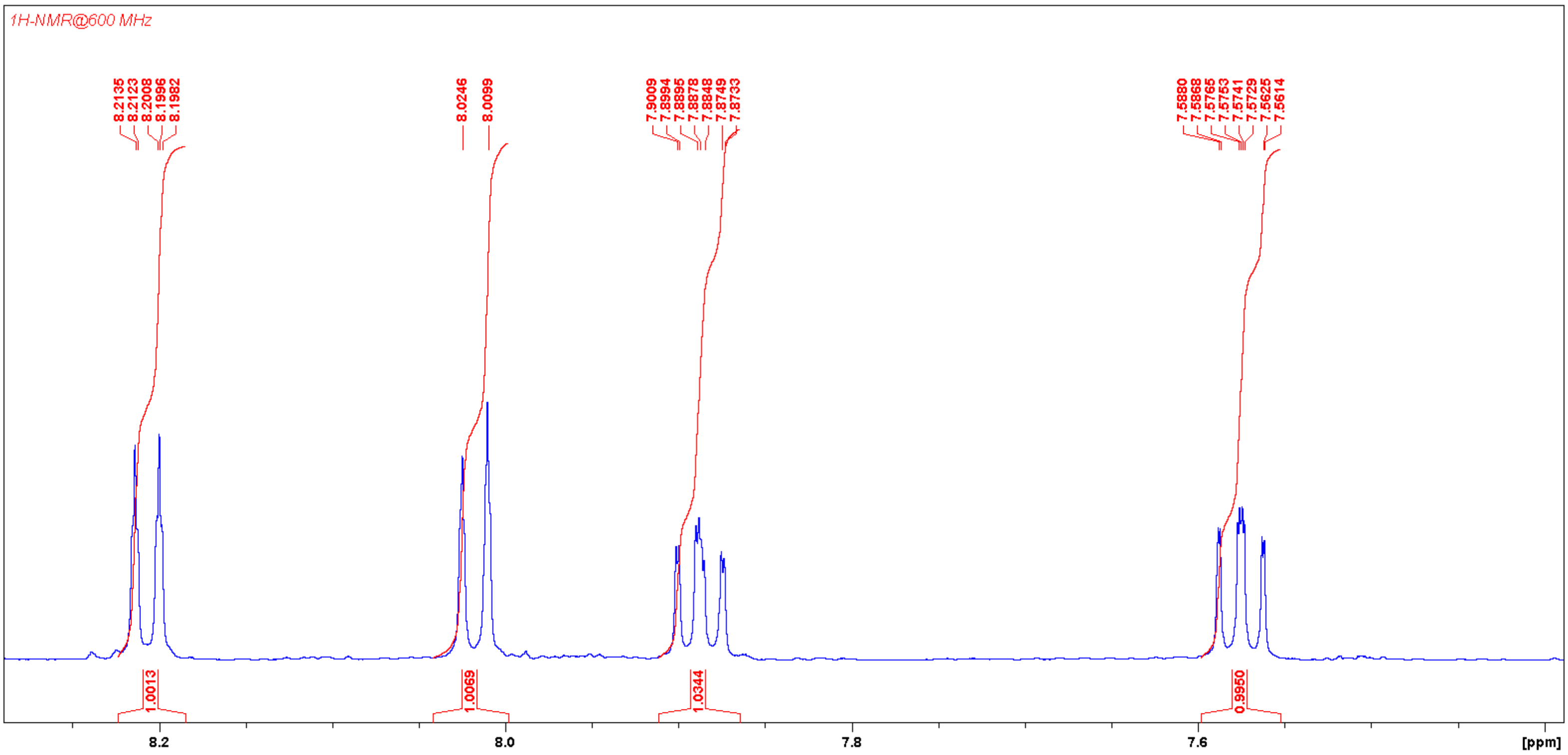
1H-NMR@600 MHz
8.2135
8.2123
8.2008
8.1996
8.1982
8.0246
8.0099
7.9009
7.8994
7.8895
7.8878
7.8848
7.8749
7.8733
7.5880
7.5868
7.5765
7.5753
7.5741
7.5729
7.5625
7.5614
1.0013
1.0069
1.0344
0.9950
8.2
8.0
7.8
7.6
[ppm]

$^{13}C\{^{1}H\}$-NMR

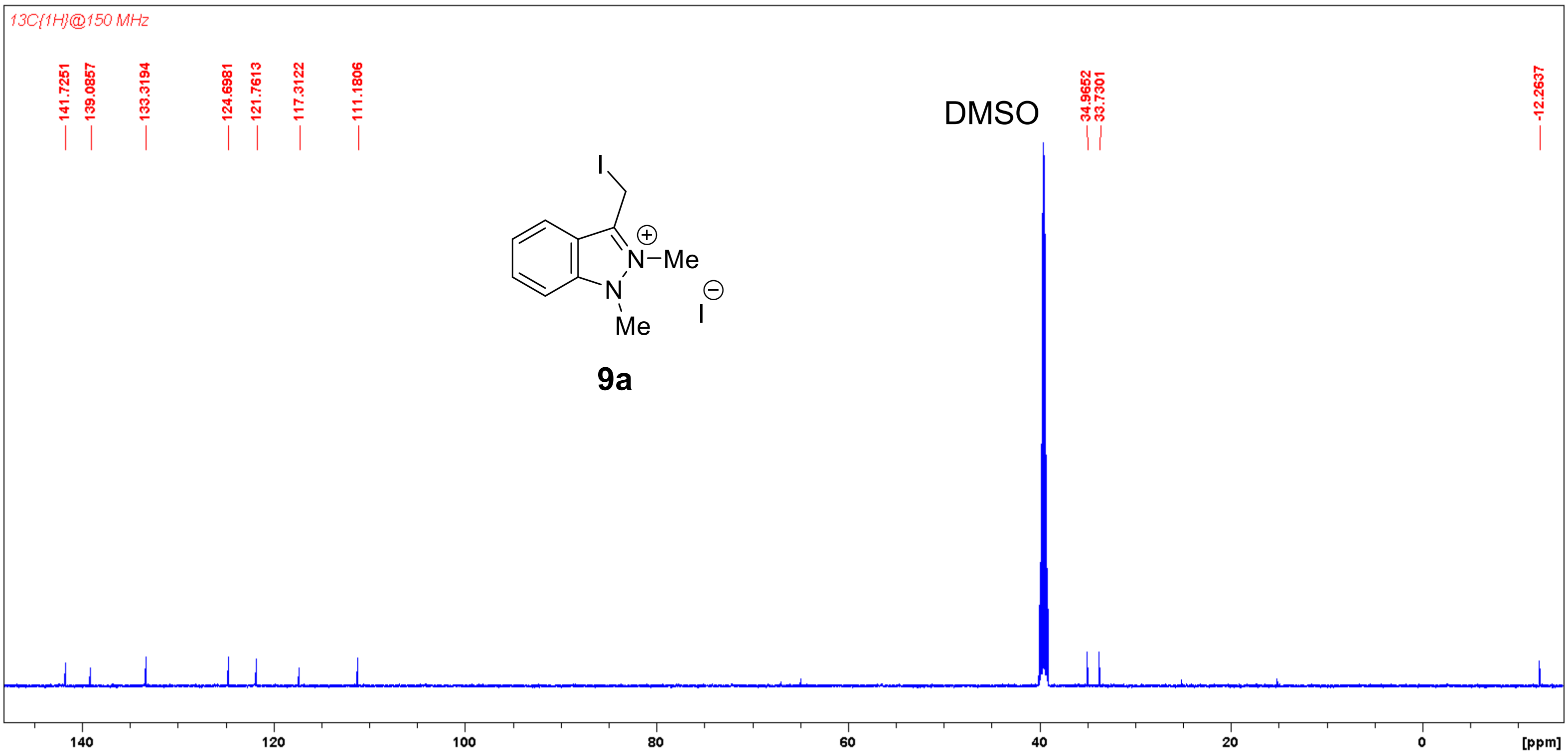

^{13}C-DEPT

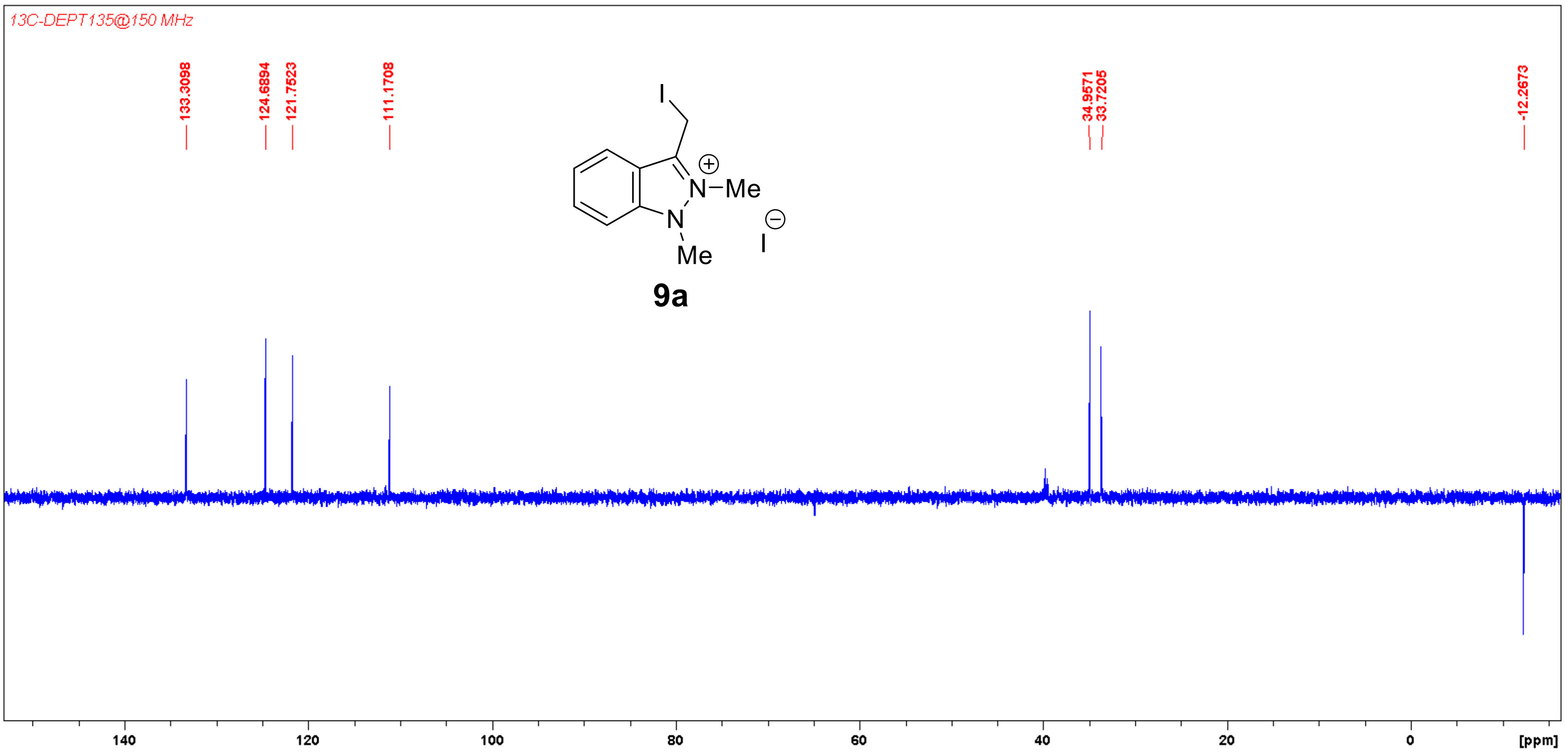

H,H-COSY

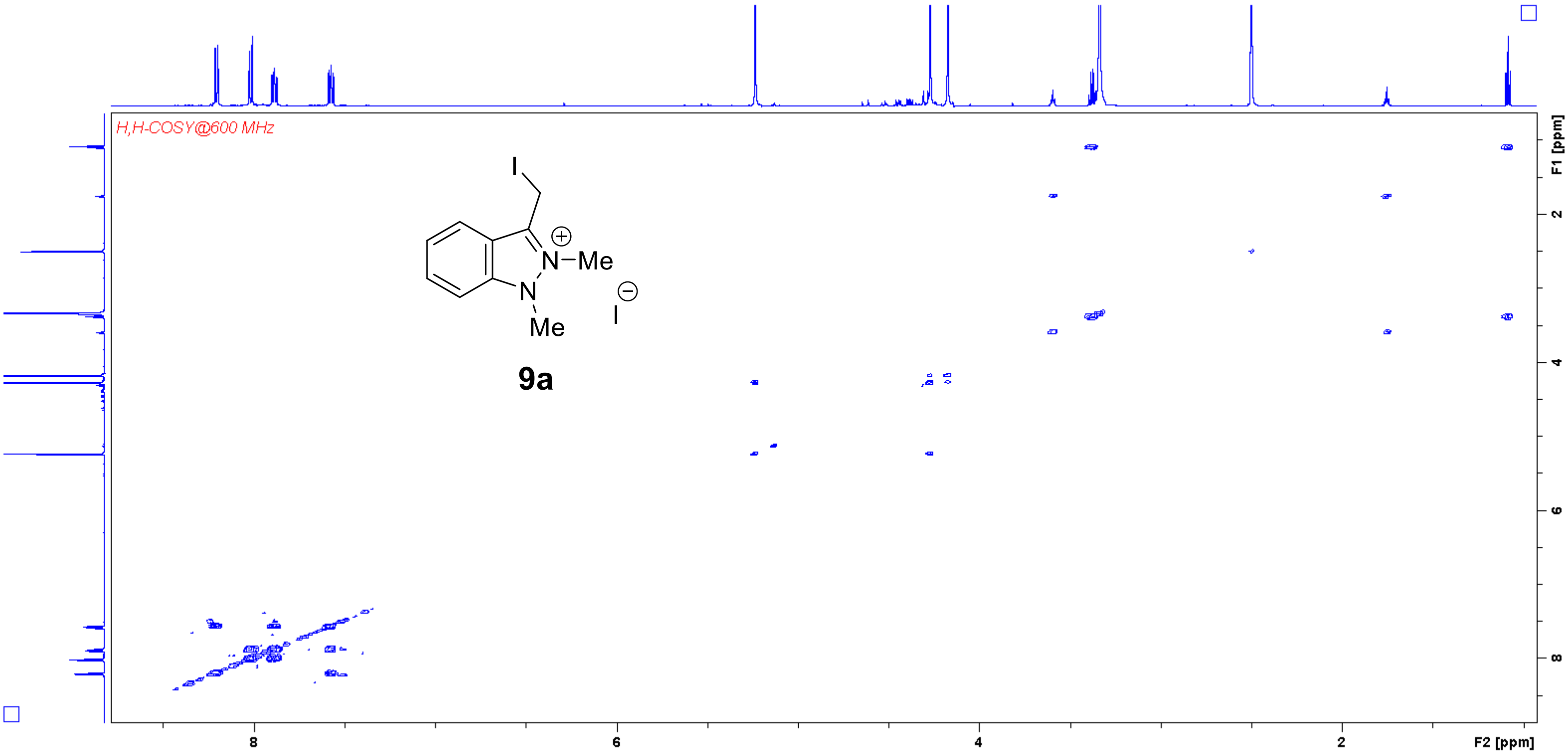
H,H-COSY@600 MHz
I
N−Me
N
Me
I
9a
F1 [ppm]
F2 [ppm]
8
6
4
2

HMBC

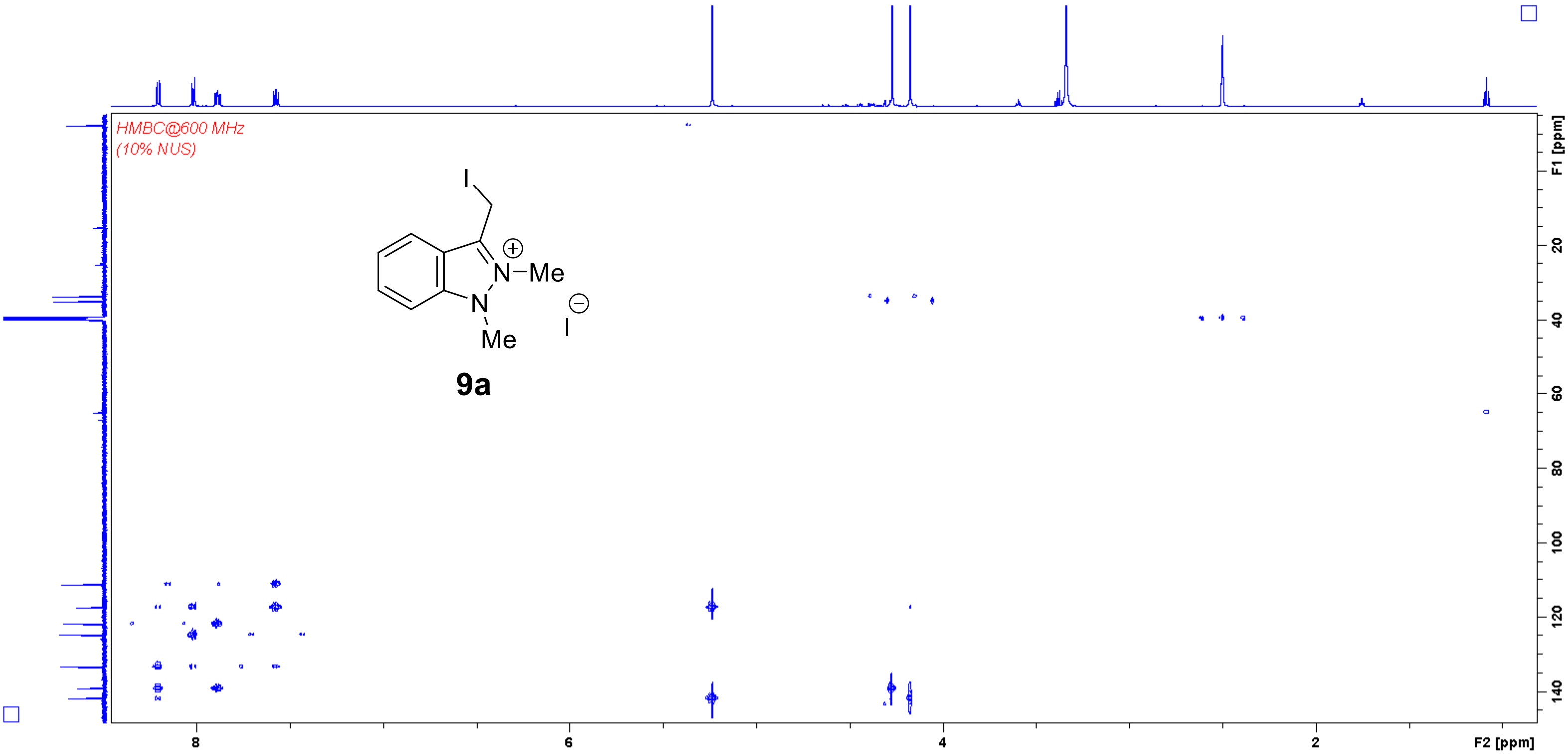
HMBC@600 MHz
(10% NUS)
I
N−Me
N
Me
I
9a
F1 [ppm]
F2 [ppm]
20
40
60
80
100
120
140
8
6
4
2

HSQC

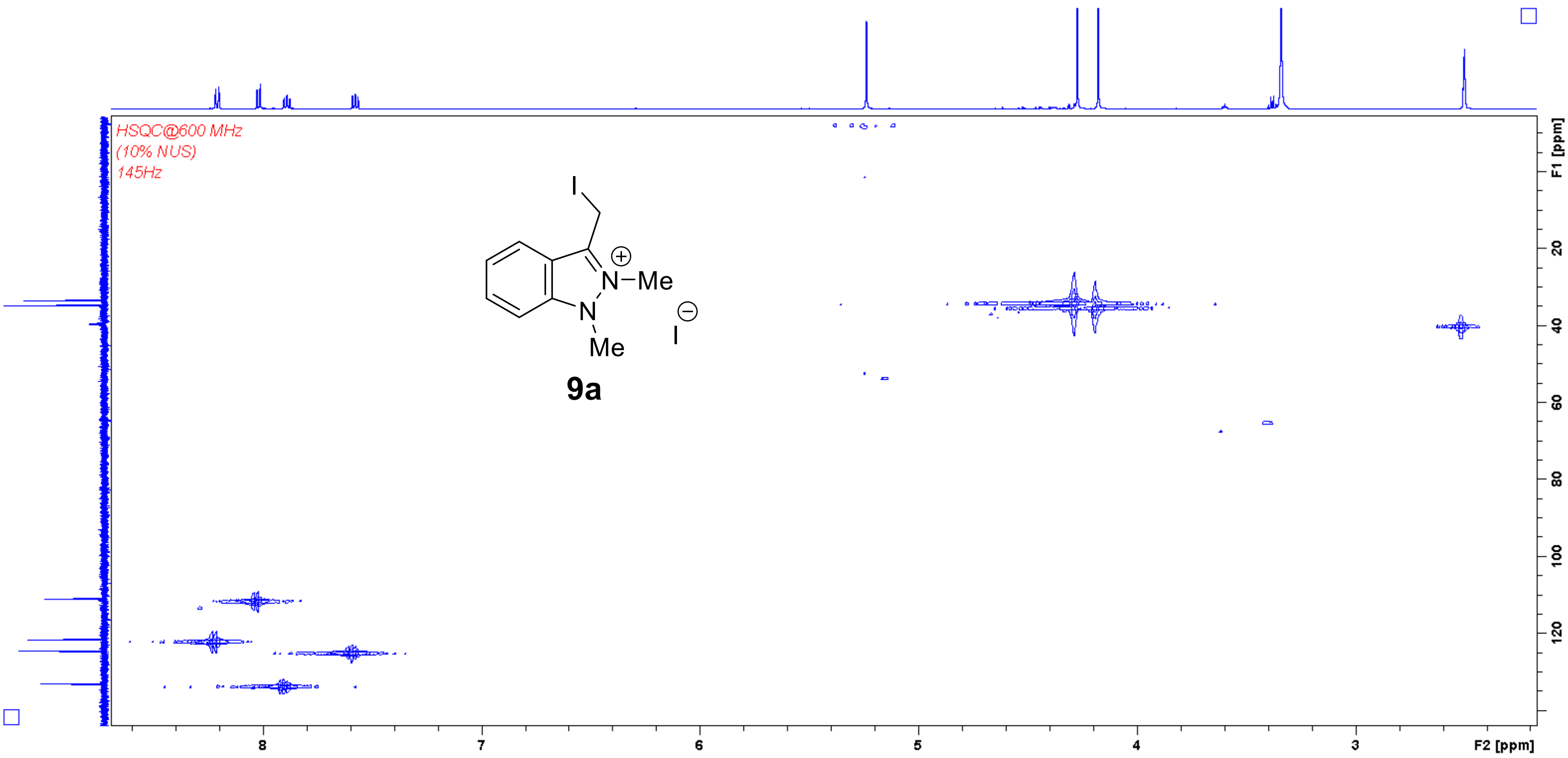

Synthesis of 3-bromomethyl-1,2-dimethyl-1*H*-indazol-2-ium bromide **9b**

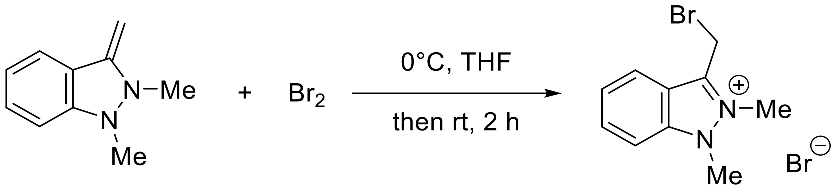

Followed by the general procedure of NHO adduct synthesis, **9b** is prepared from 1,2,3-trimethyl-1*H*-indazolium tetrafluoroborate **1a** (1.0 Eq, 100 mg, 0.403 mmol) and bromine (1 Eq, 65 mg, 0.403 mmol) as yellow solid (90 mg, 70%).

^{1}H-NMR (DMSO-d_6, 600 MHz): 8.27 (d, J = 8.3 Hz, 1H, Ar-H), 8.06 (d, J = 8.3 Hz, 1H, Ar-H), 7.91 (t, J = 8.3 Hz, 1H, Ar-H), 7.59 (t, J = 8.3 Hz, 1H, Ar-H), 5.51 (s, 2H, CH_2), 4.34 (s, 3H, N-Me), 4.33 (s, 3H, N-Me) ppm.

^{13}C{^{1}H}-NMR (DMSO-d_6, 150 MHz): 139.3 (o, Ar-C), 139.0 (o, Ar-C), 133.3 (+, Ar-C), 125.2 (+, Ar-C), 121.5 (+, Ar-C), 117.9 (o, Ar-C), 111.3 (+, Ar-C), 35.2 (+, N-Me), 34.0 (+, N-Me), 17.8 (-, CH_2) ppm.

ESI-MS (m/z): calculated for $[C_{10}H_{12}N_2Br]^+$: 239.0178, found 239.0174.

IR (ATR): $\tilde{\nu}$ = 600 (C-Br stretching) cm^{-1}.

Melting point: 197-199 °C, decomposed.

^{1}H-NMR

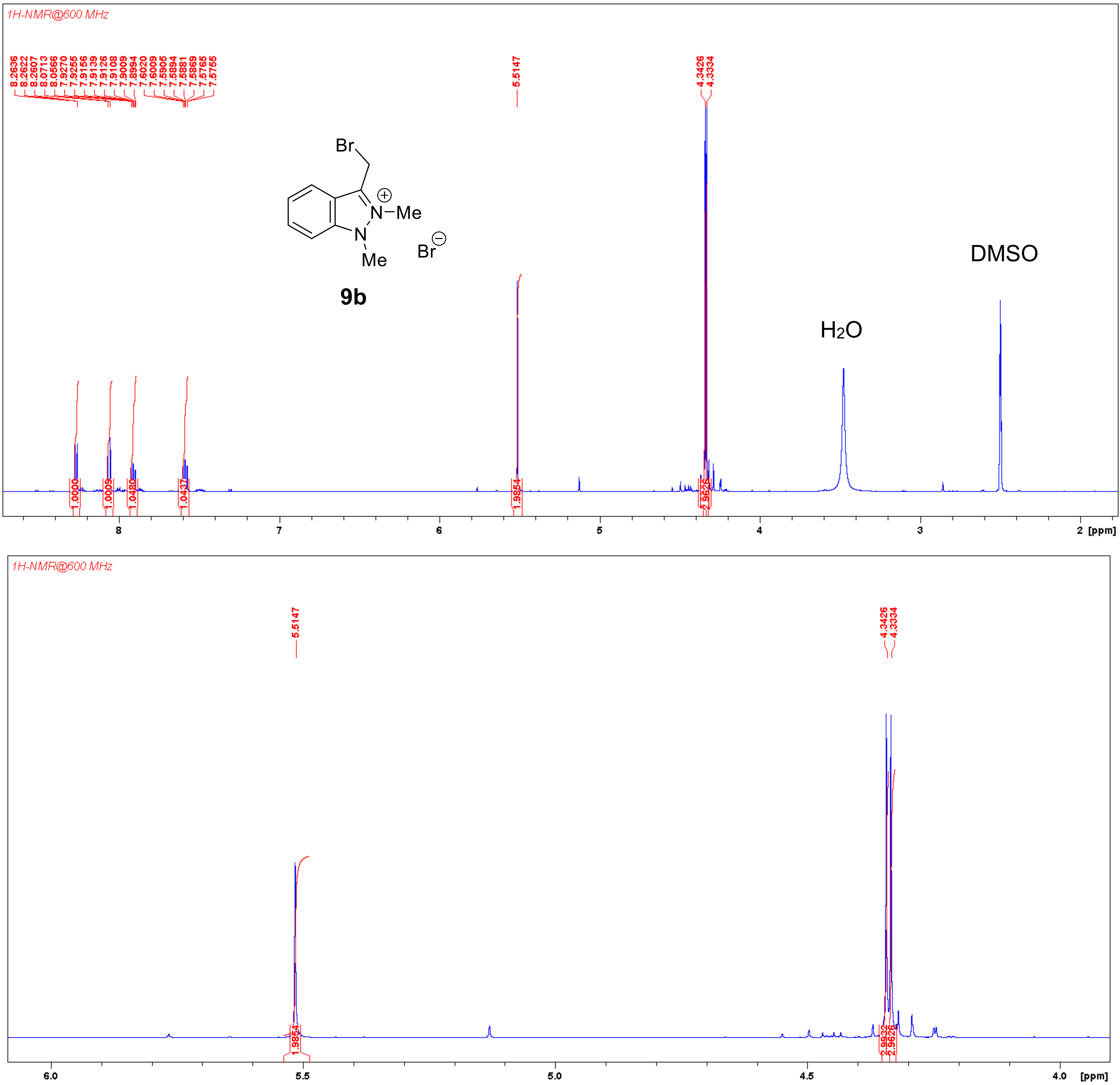

$^{13}C\{^1H\}$-NMR

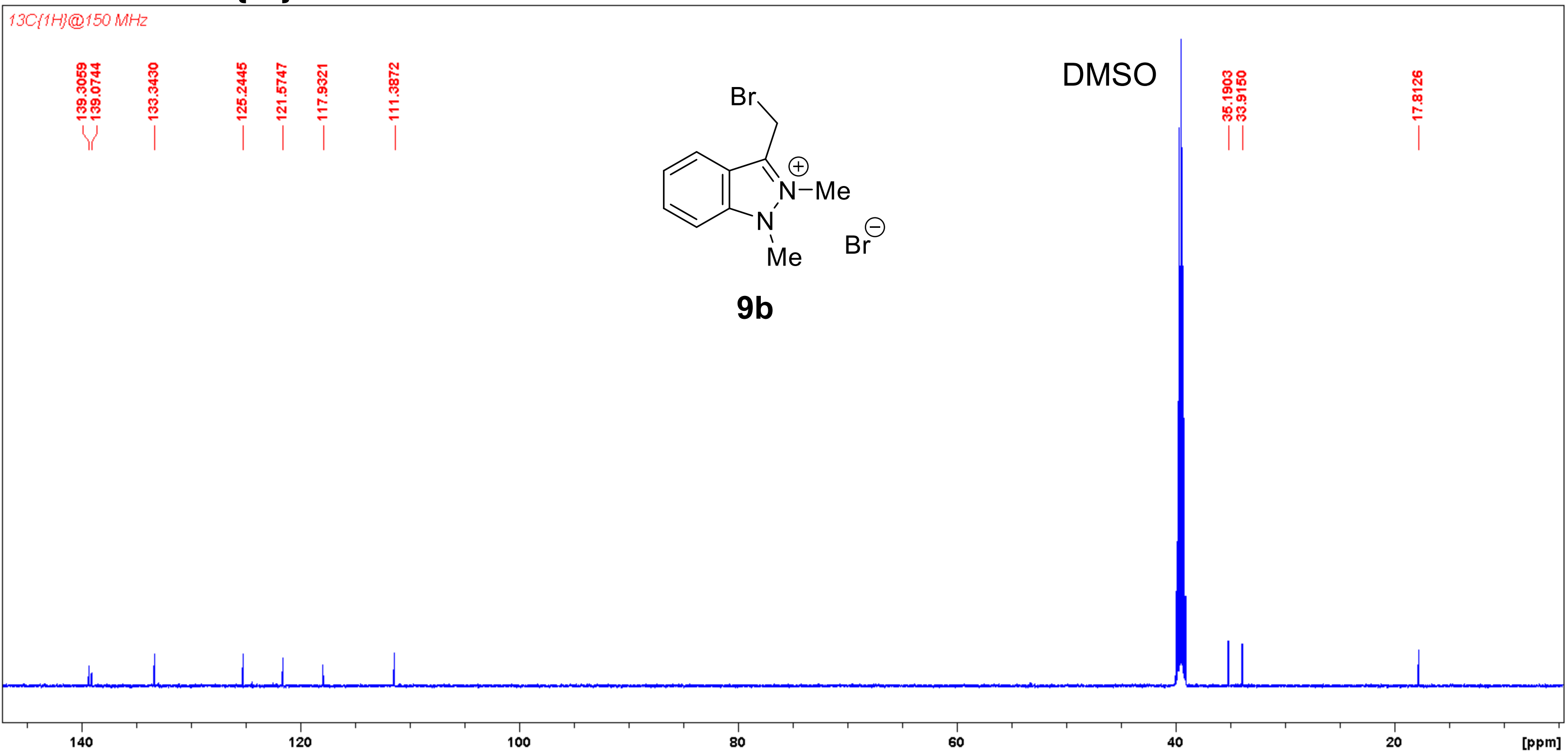

^{13}C-DEPT

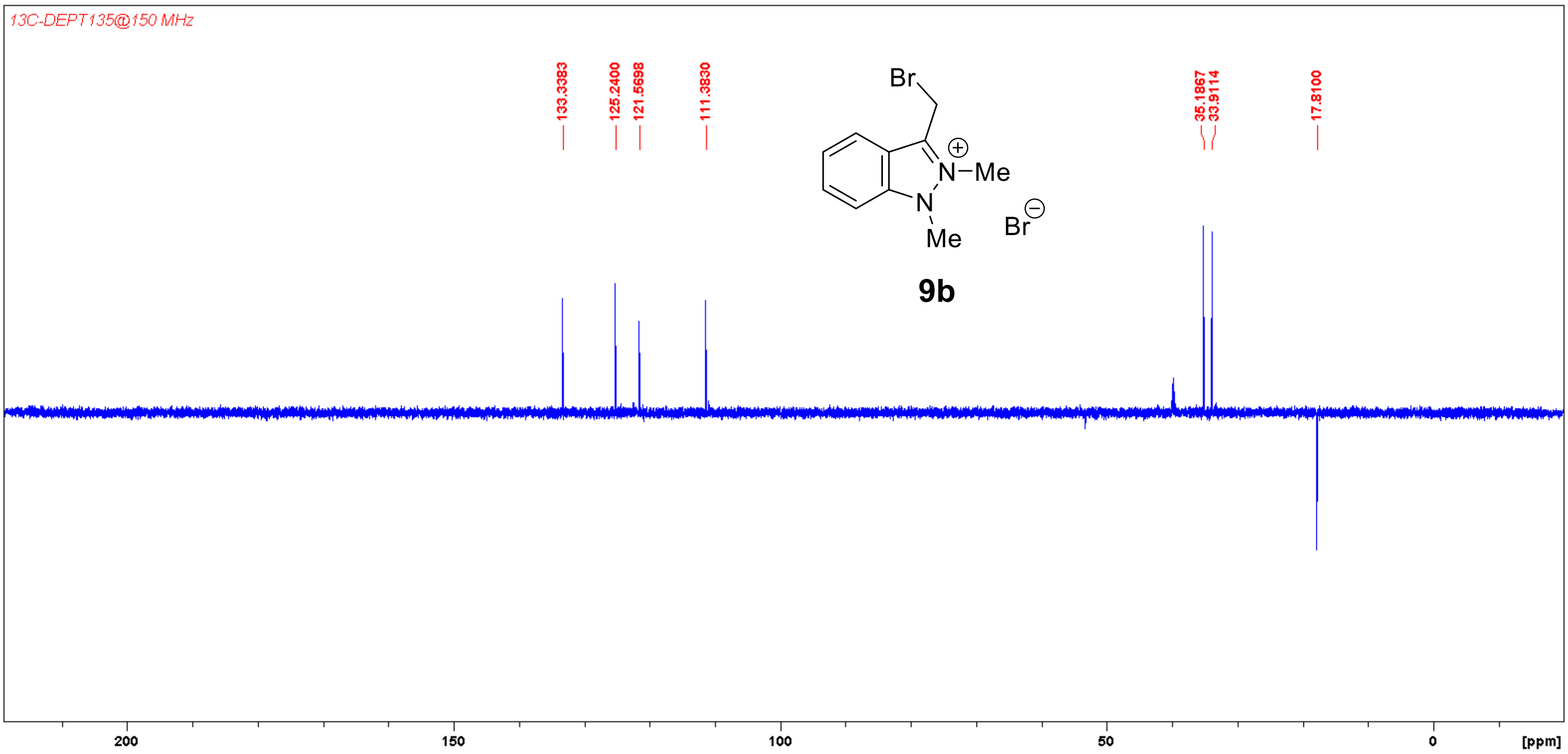

H,H-COSY

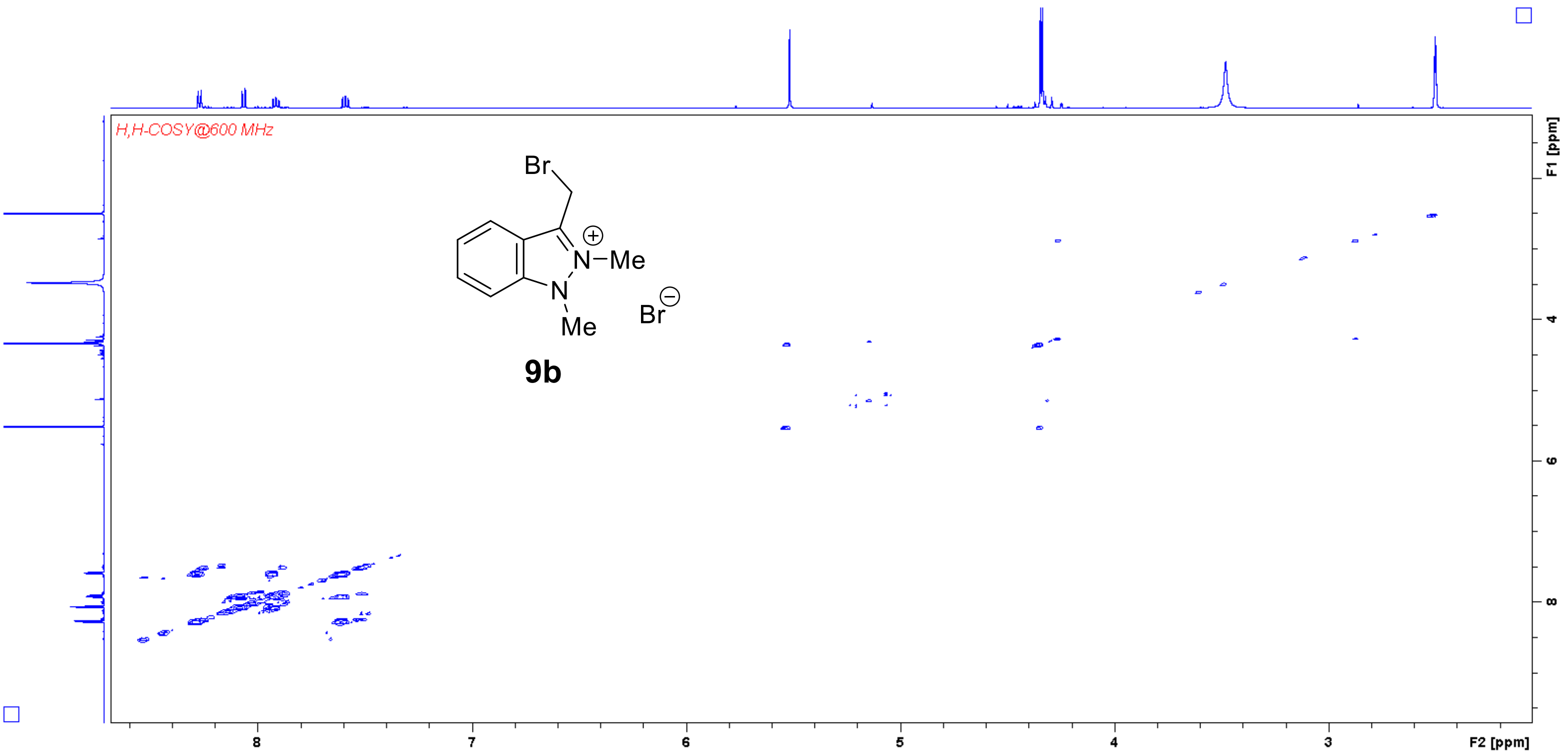

H,H-COSY@600 MHz
Br
N−Me
N
Me
Br
9b
F1 [ppm]
F2 [ppm]

HMBC

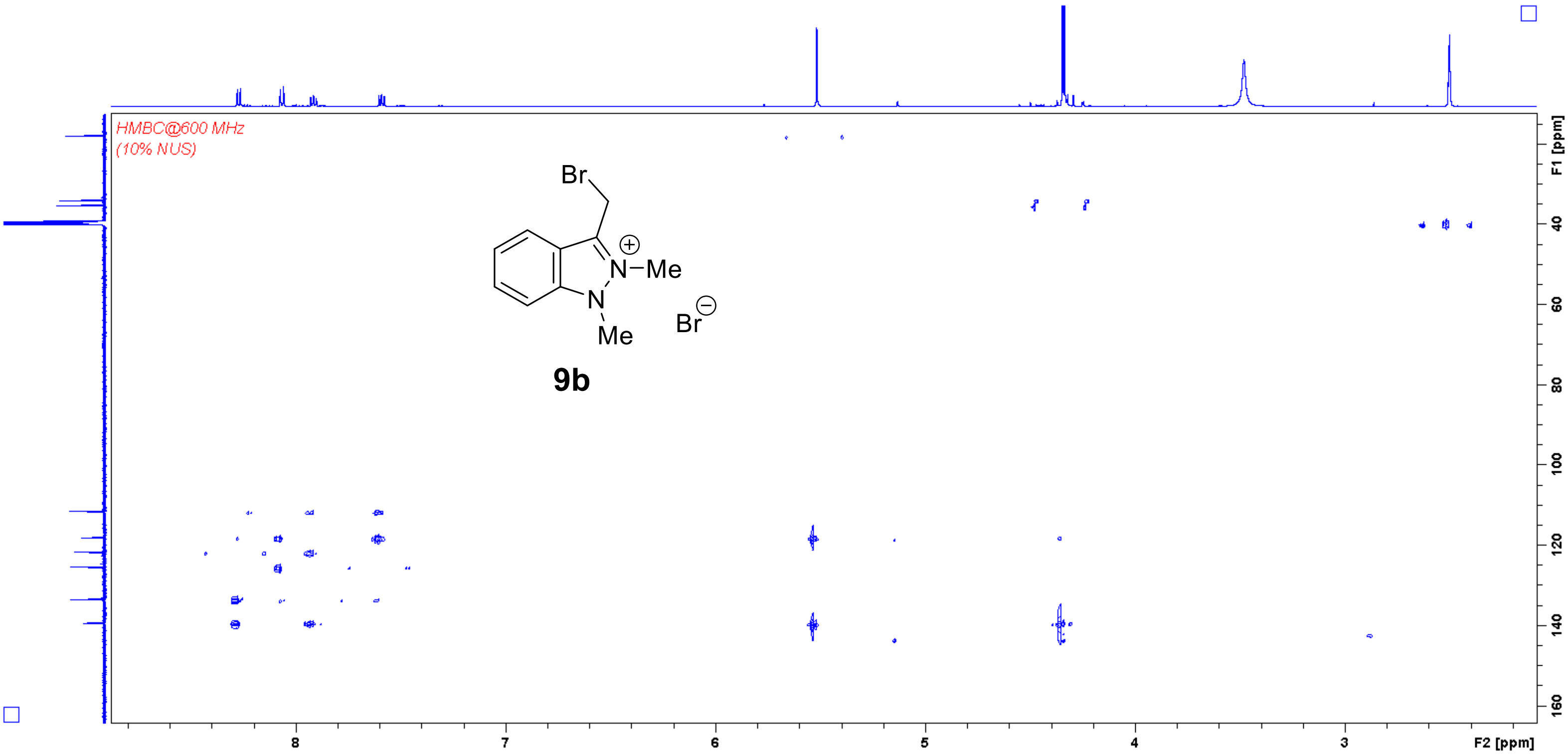

HMBC@600 MHz
(10% NUS)
Br
N−Me
N
Me
Br
9b
F1 [ppm]
F2 [ppm]

HSQC

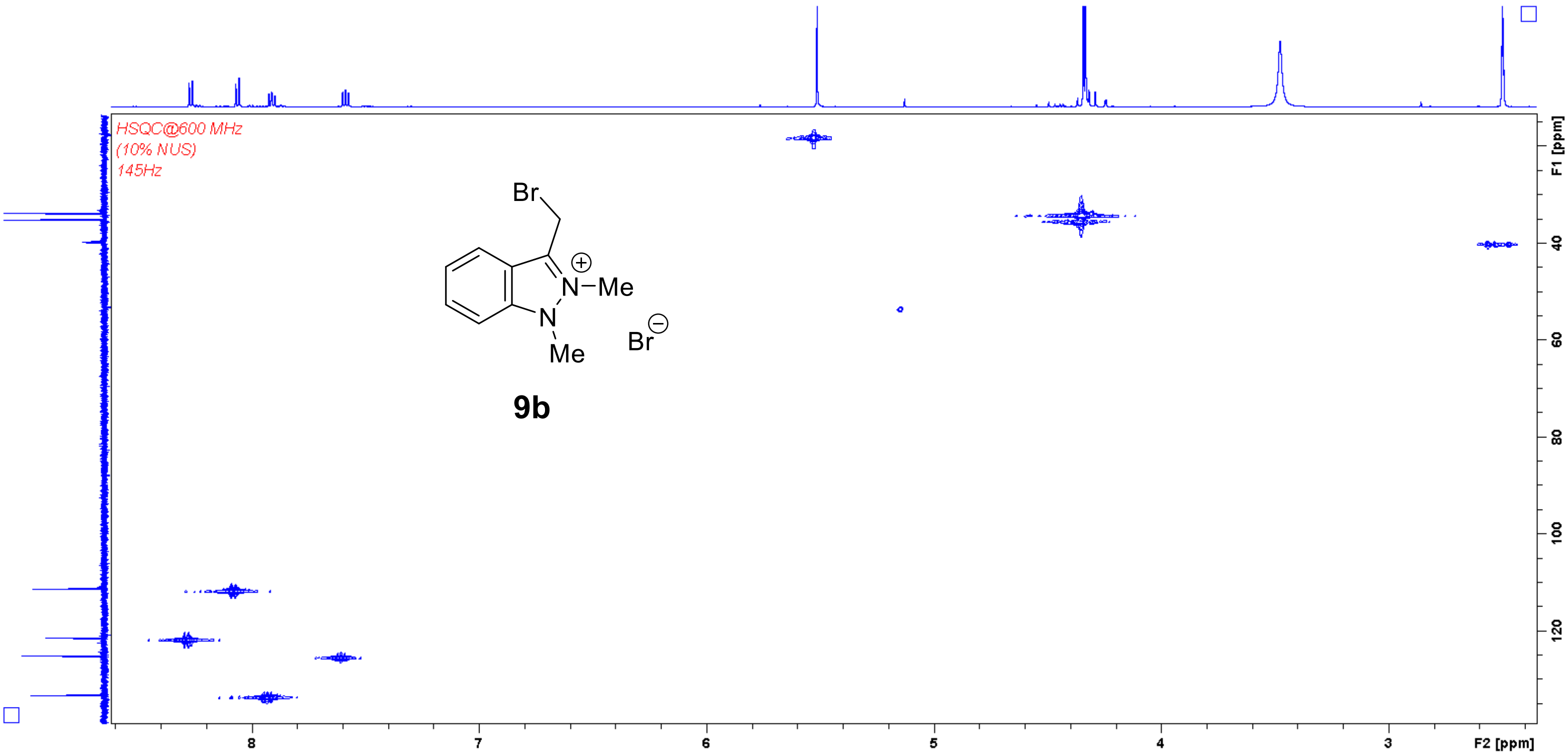

HSQC@600 MHz
(10% NUS)
145Hz
Br
N−Me
N
Me
Br
9b
F1 [ppm]
40
60
80
100
120
8
7
6
5
4
3
F2 [ppm]

Synthesis of 3-bromomethyl-1,2-dimethyl-1*H*-indazol-2-ium bromide **9c**

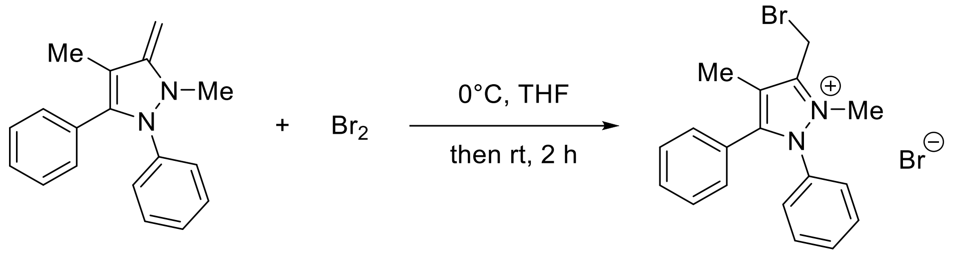

Followed by the general procedure of NHO, **9c** is prepared from 1,5-diphenyl-3,4,5-trimethyl-1*H*-pyrazolium iodide **3b** (1.0 Eq, 100 mg, 0.256 mmol) and bromine (1 Eq, 41 mg, 0.256 mmol) as yellow solid (99 mg, 92%).

^{1}H-NMR (DMSO-d$_6$, 600 MHz): 7.70-7.68 (m, 2H, Ar-H), 7.64-7.62 (m, 1H, Ar-H), 7.60-7.58 (m, 2H, Ar-H), 7.45-7.41 (m, 3H, Ar-H), 7.36-7.34 (m, 2H, Ar-H), 5.19 (s, 2H, CH_2Br), 3.81 (s, 3H, N-Me), 2.19 (s, 3H, Me) ppm.

^{13}C{^{1}H}-NMR (DMSO-d$_6$, 150 MHz): 146.7 (o, Ar-C), 144.0 (o, Ar-C), 132.3 (+, Ar-C), 131.4 (o, Ar-C), 130.6 (+, Ar-C), 130.1 (+, Ar-C), 129.8 (+, Ar-C), 129.5 (+, Ar-C), 128.9 (+, Ar-C), 125.1 (o, Ar-C), 116.3 (o, Ar-C), 35.3 (+, N-Me), 17.9 (-, CH_2Br), 7.8 (+, Me) ppm.

ESI-MS (m/z): calculated for $[C_{18}H_{18}N_2Br]^+$: 341.0648, found 341.0643.

IR (ATR): $\tilde{\nu}$ = 690 (C-Br stretching) cm^{-1}.

Melting point: 94-95 °C.

^{1}H-NMR

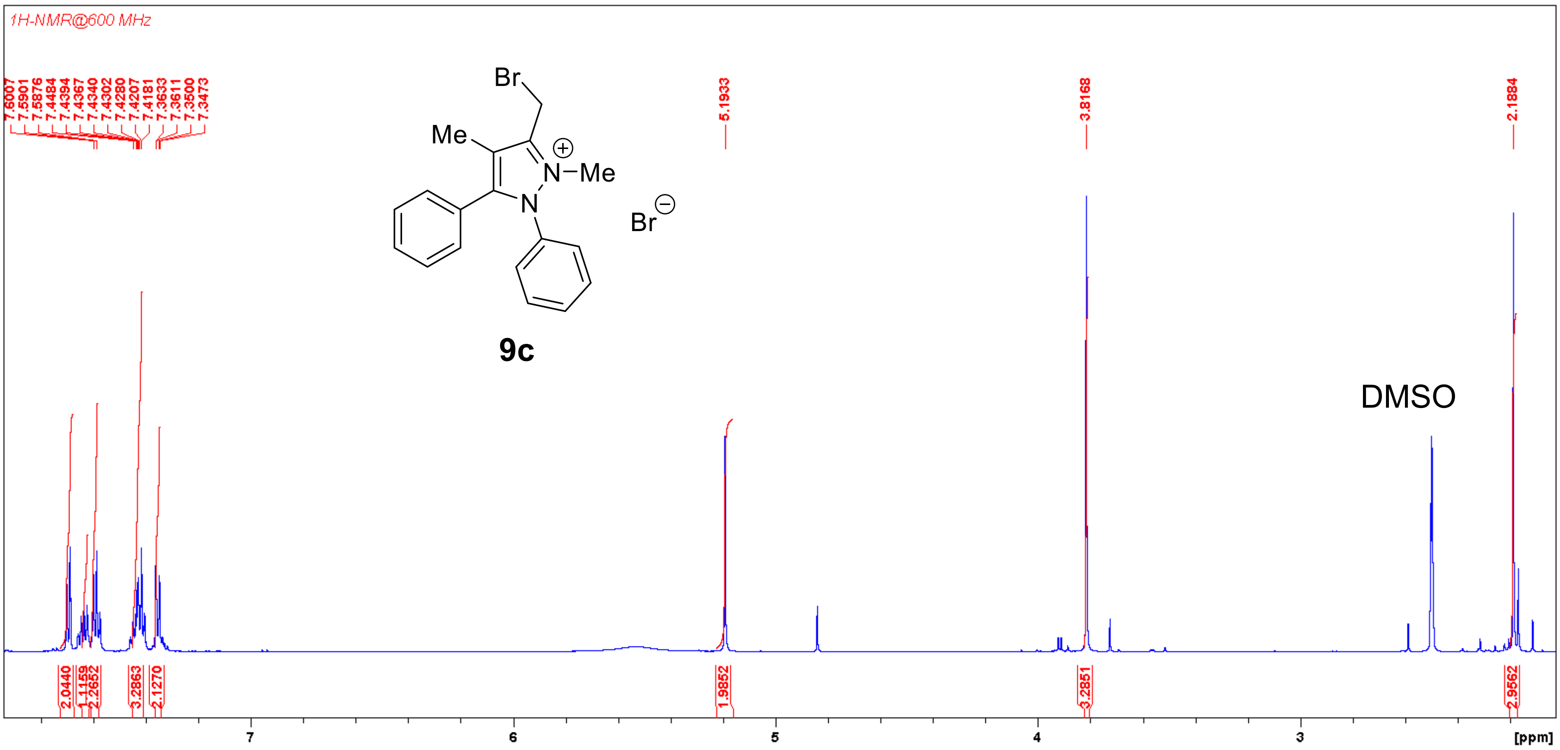

^{13}C{^{1}H}-NMR

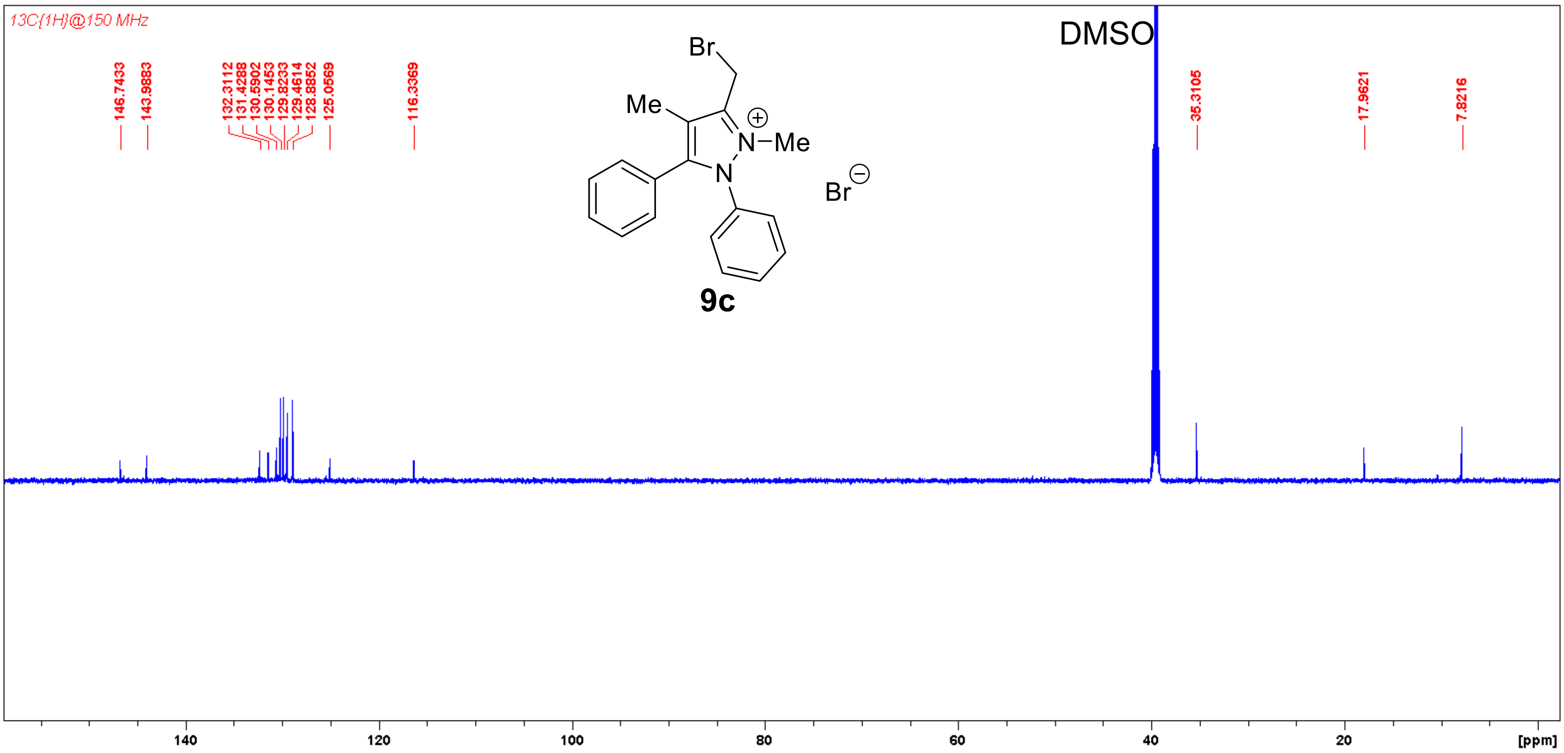

^{13}C-DEPT

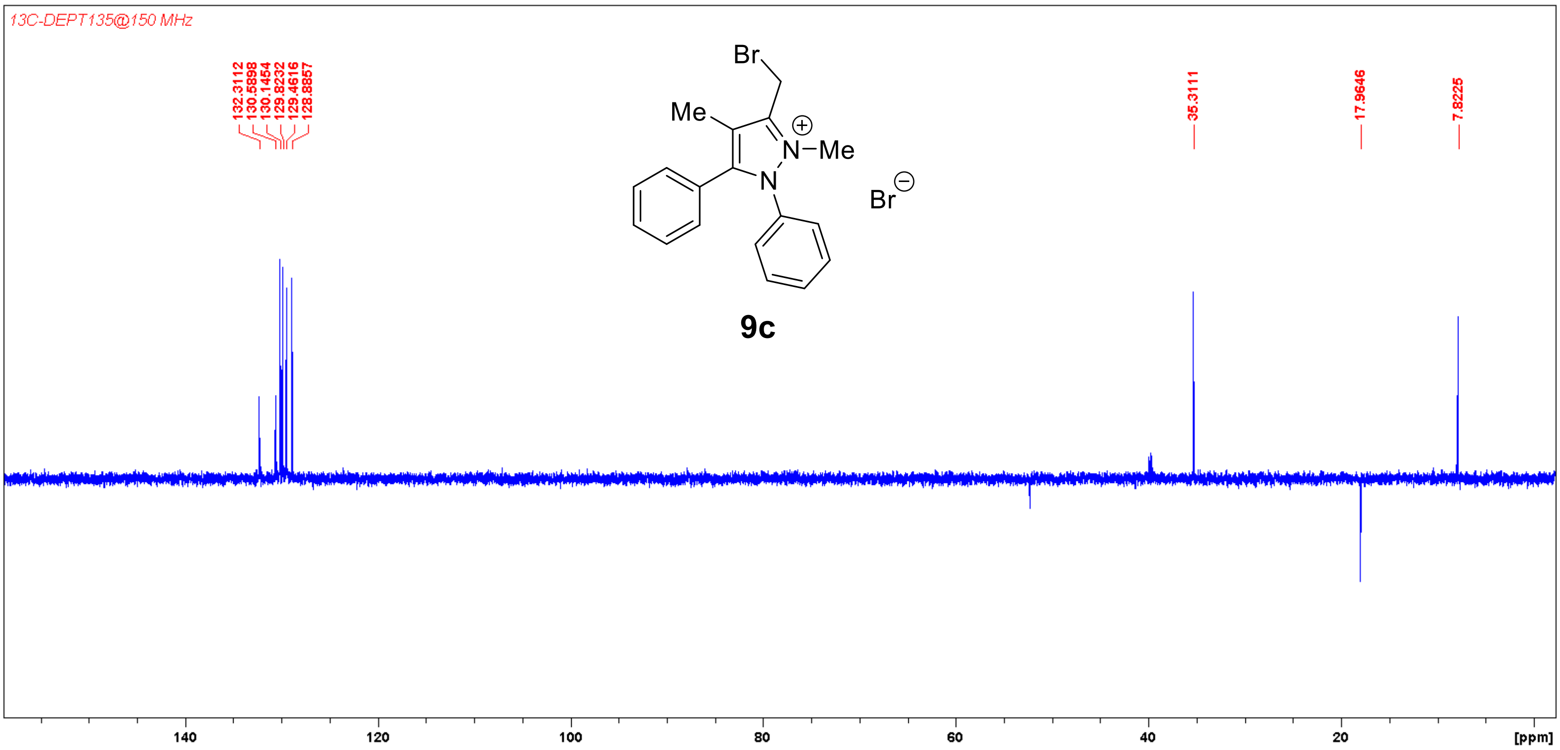

H,H-COSY

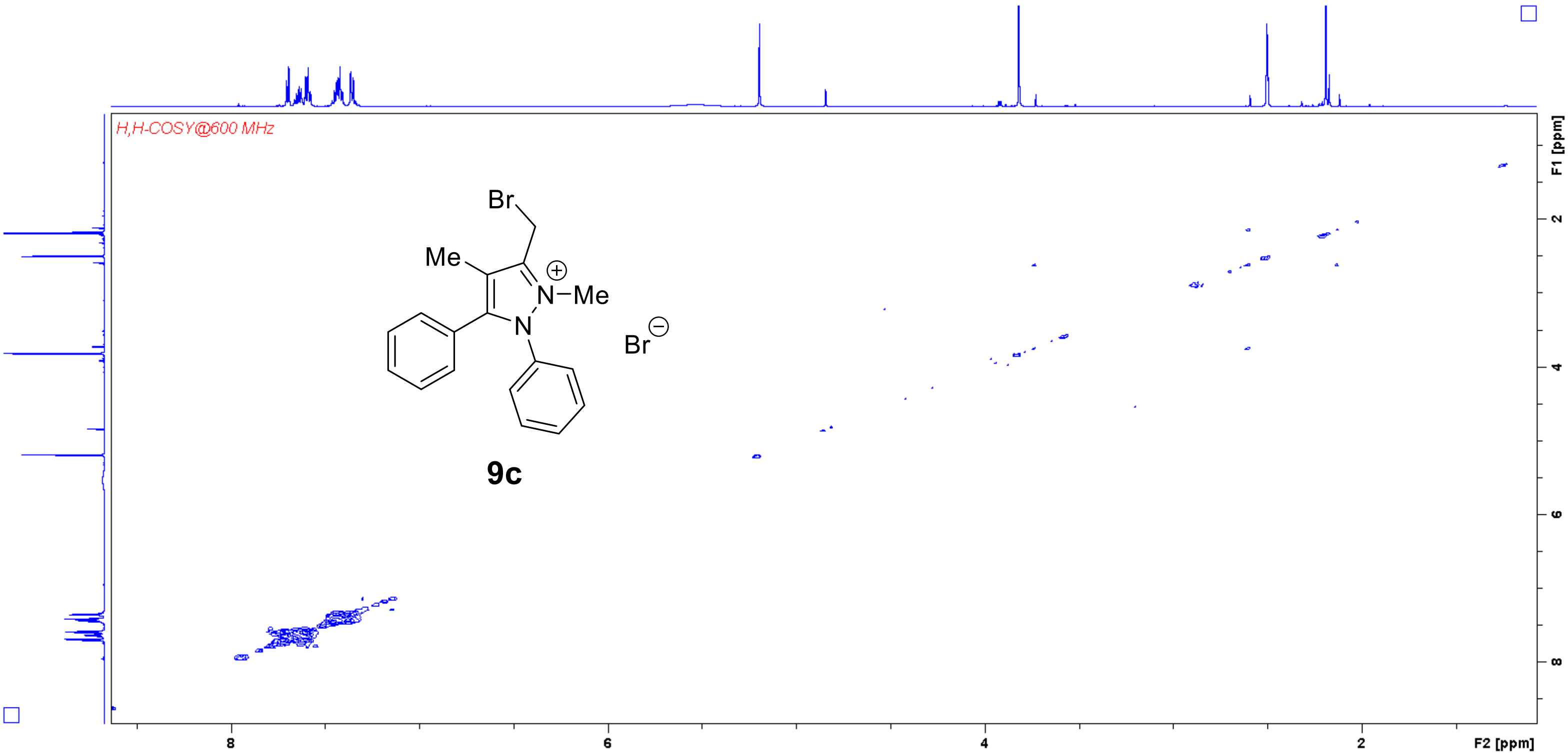

HSQC

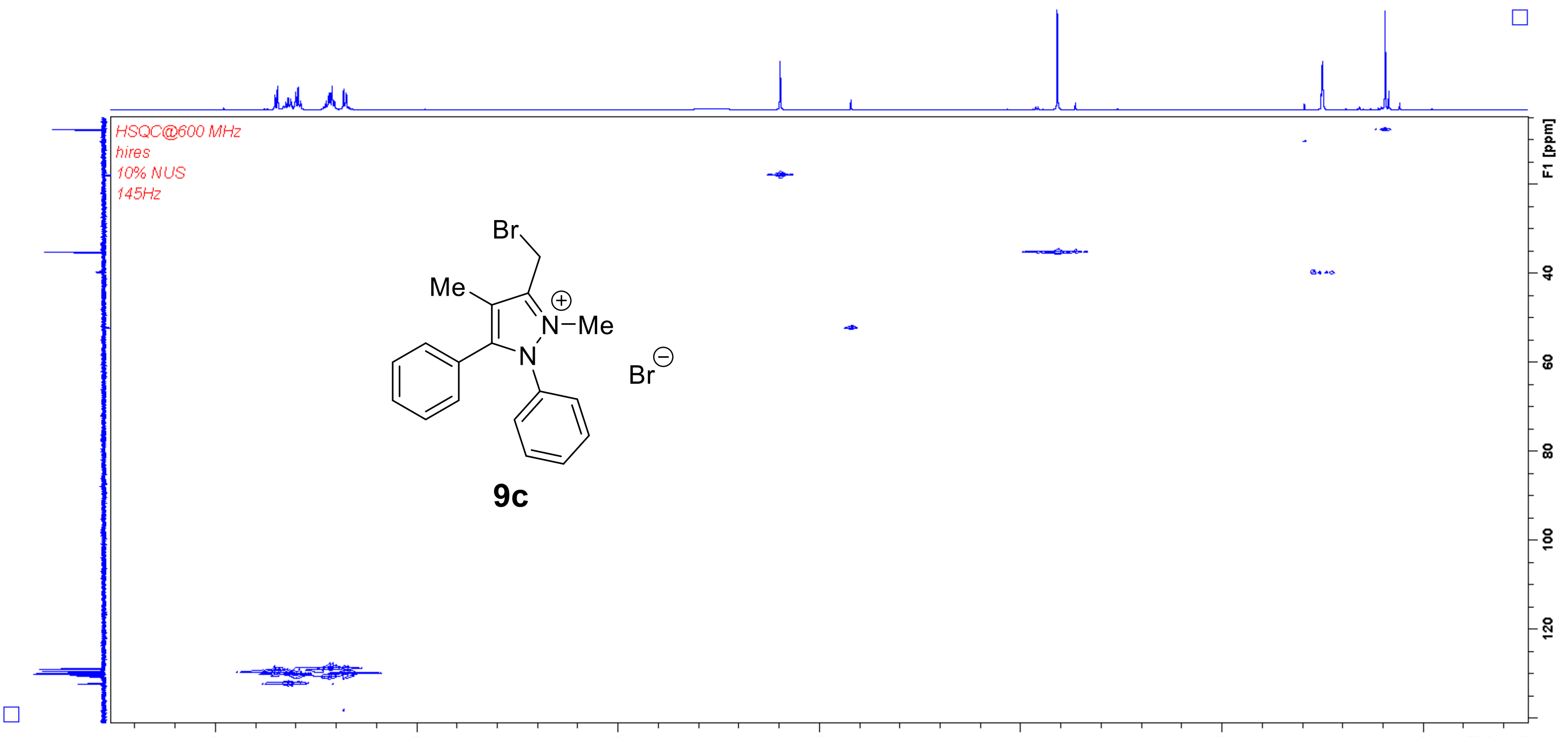
HSQC@600 MHz
hires
10% NUS
145Hz
Br
Me
N−Me
N
Br
9c
F1 [ppm]
F2 [ppm]

HMBC

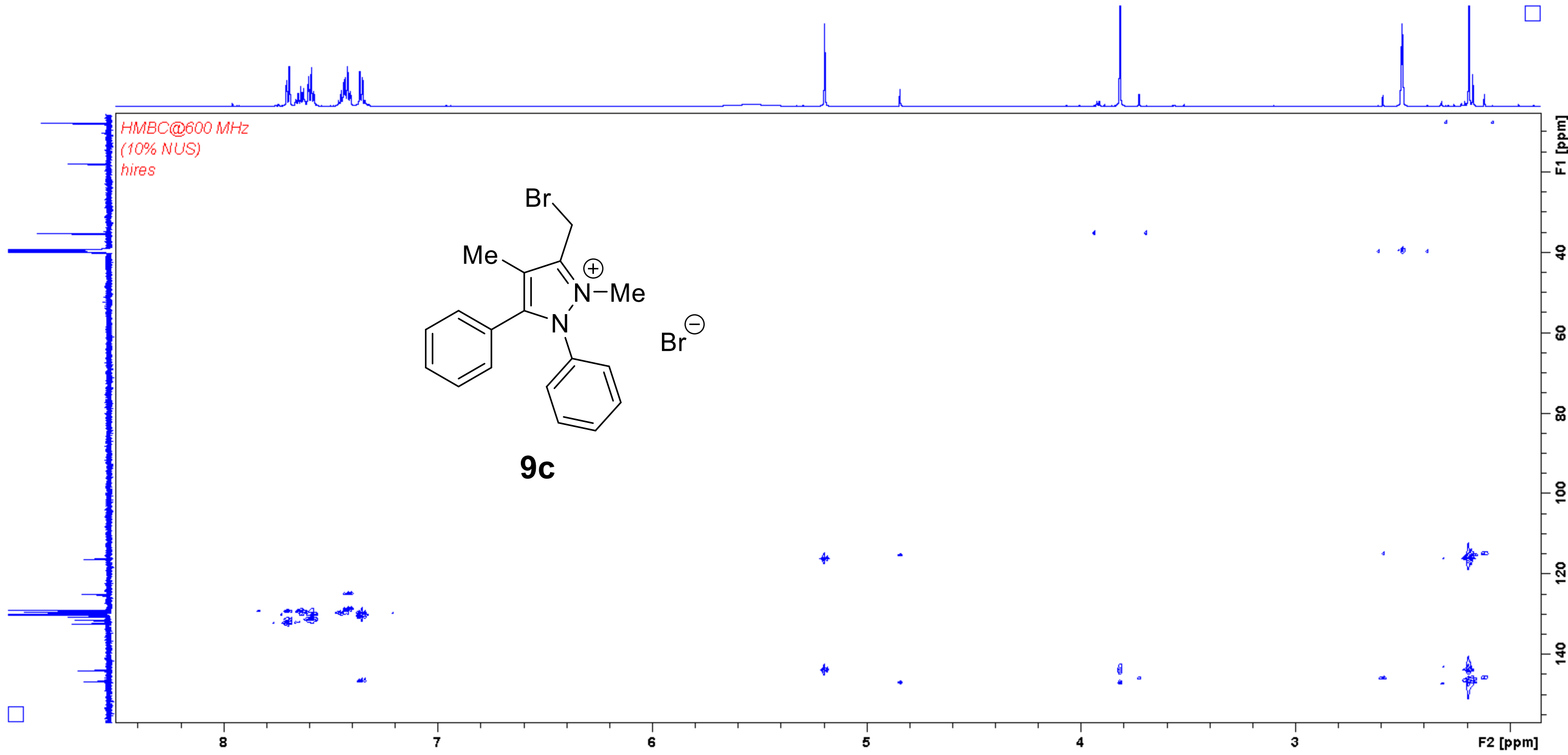
HMBC@600 MHz
(10% NUS)
hires
Br
Me
N−Me
N
Br
9c
F1 [ppm]
F2 [ppm]

Synthesis of 2-(1,2-dimethyl-1*H*-indazol-2-ium-3-yl)ethanedithioate **10a**

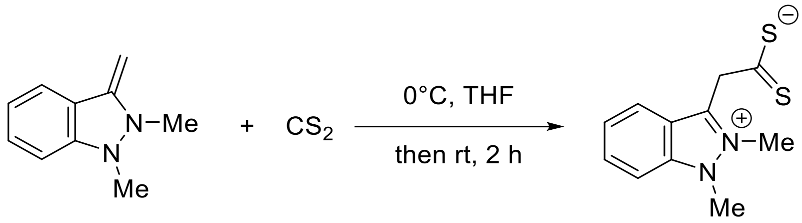

Followed by the general procedure of NHO adduct synthesis, **10a** is prepared from 1,2,3-trimethyl-1*H*-indazolium tetrafluoroborate **1a** (1.0 Eq, 100 mg, 0.403 mmol) and carbon disulfide (1 Eq, 31mg, 0.403 mmol) as orange-red solid (73 mg, 77%).

^{1}H-NMR (DMSO-d$_6$, 600 MHz): 8.14 (d, J = 8.1 Hz, 1H, Ar-H), 7.91 (d, J = 8.1 Hz, 1H, Ar-H), 7.82 (t, J = 8.1 Hz, 1H, Ar-H), 7.44 (t, J = 8.1Hz, 1H, Ar-H), 4.94 (s, 2H, CH_2), 4.30 (s, 3H, N-Me), 4.24 (s, 3H, N-Me) ppm.

^{13}C{^{1}H}-NMR (DMSO-d$_6$, 150 MHz): 246.4 (o, CS_2), 143.1 (o, Ar-C), 139.2 (o, Ar-C), 132.6 (+, Ar-C), 123.9 (+, Ar-C), 123.0 (+, Ar-C), 119.4 (o, Ar-C), 110.6 (+, Ar-C), 55.3 (-, CH_2), 35.1 (+, N-Me), 33.2 (+, N-Me) ppm.

ESI-MS (m/z): calculated for $[C_{11}H_{12}N_2S_2+Na]^+$: 259.0334, found 259.0335.

IR (ATR): 1214 (C=S stretching) cm^{-1}.

Melting point: 157-158 °C, decomposed.

^{1}H-NMR

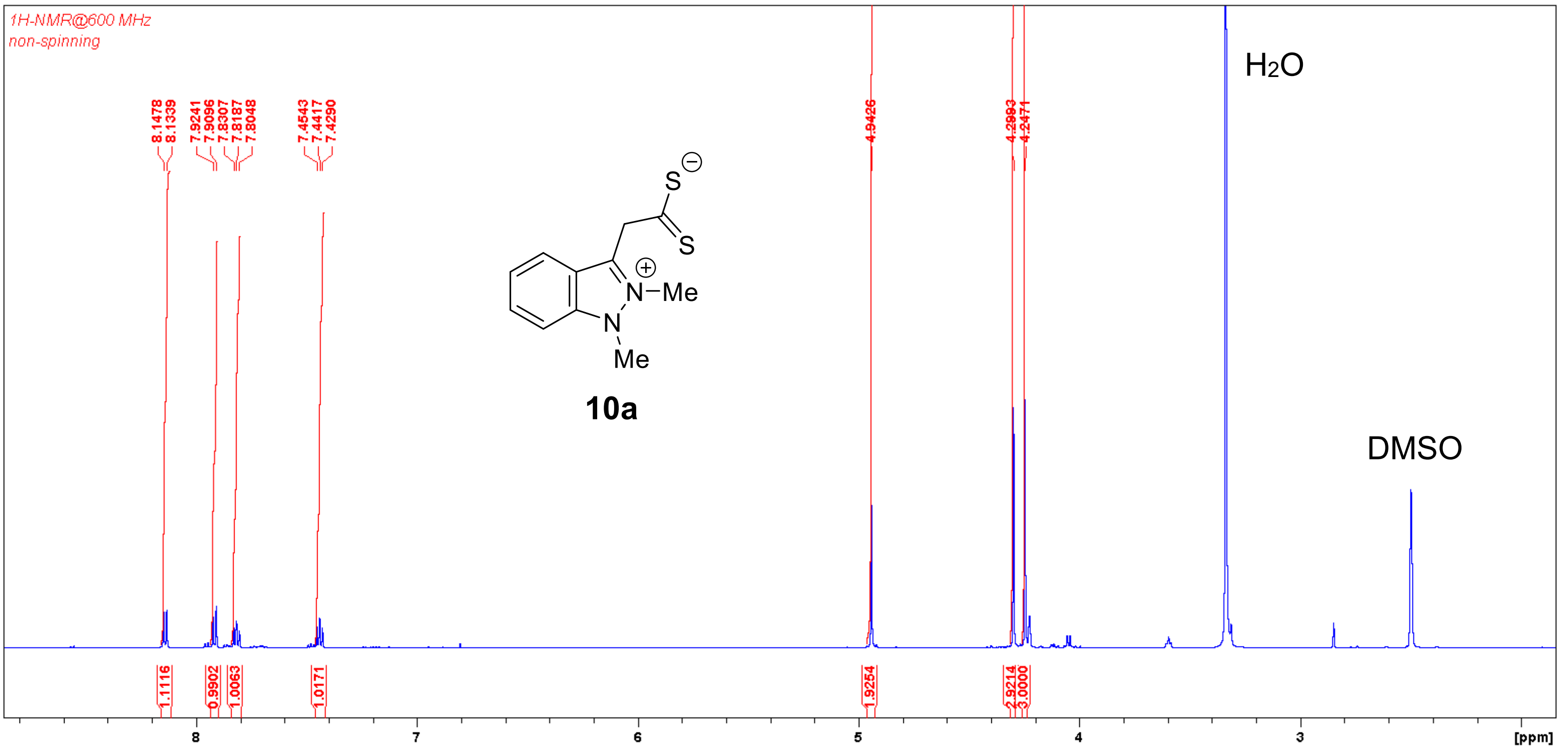
1H-NMR@600 MHz
non-spinning
8.1478
8.1339
7.9241
7.9096
7.8307
7.8187
7.8048
7.4543
7.4417
7.4290
4.9426
4.2993
4.2471
H2O
DMSO
S
S
N
N
Me
Me
10a
1.1116
0.9902
1.0063
1.0171
1.9254
2.9214
3.0000
8
7
6
5
4
3
[ppm]

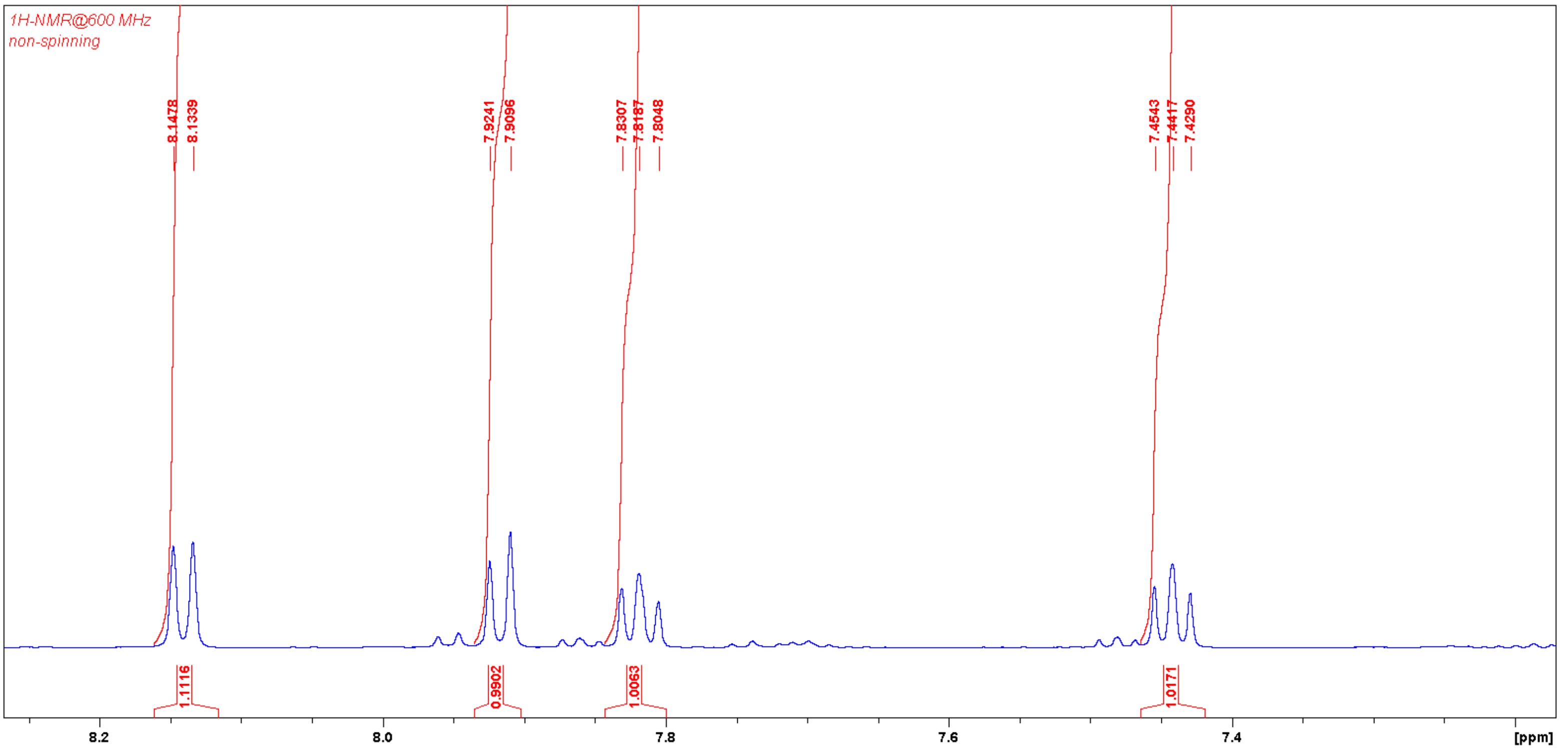
1H-NMR@600 MHz
non-spinning
8.1478
8.1339
7.9241
7.9096
7.8307
7.8187
7.8048
7.4543
7.4417
7.4290
1.1116
0.9902
1.0063
1.0171
8.2
8.0
7.8
7.6
7.4
[ppm]

$^{13}C\{^{1}H\}$-NMR

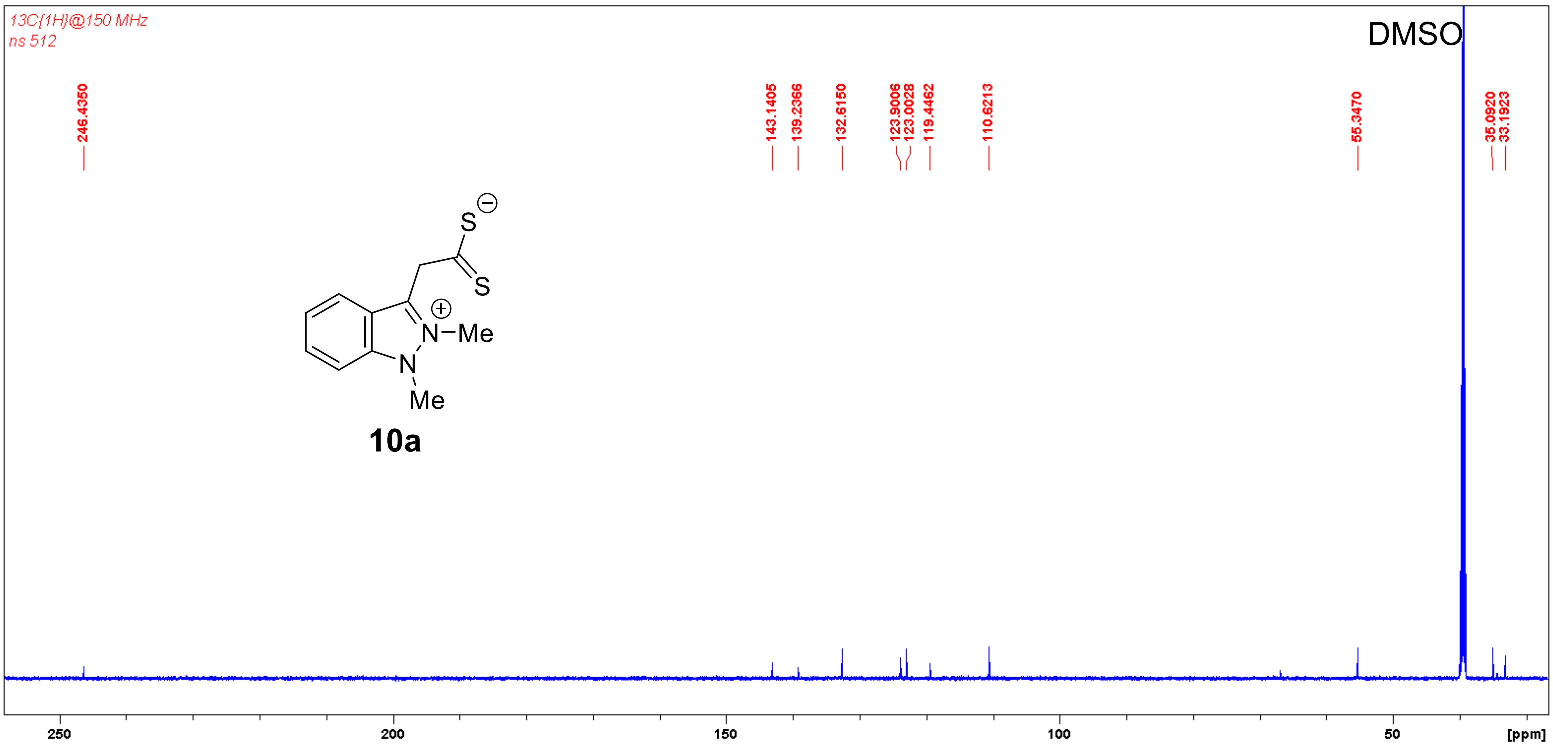

^{13}C-DEPT

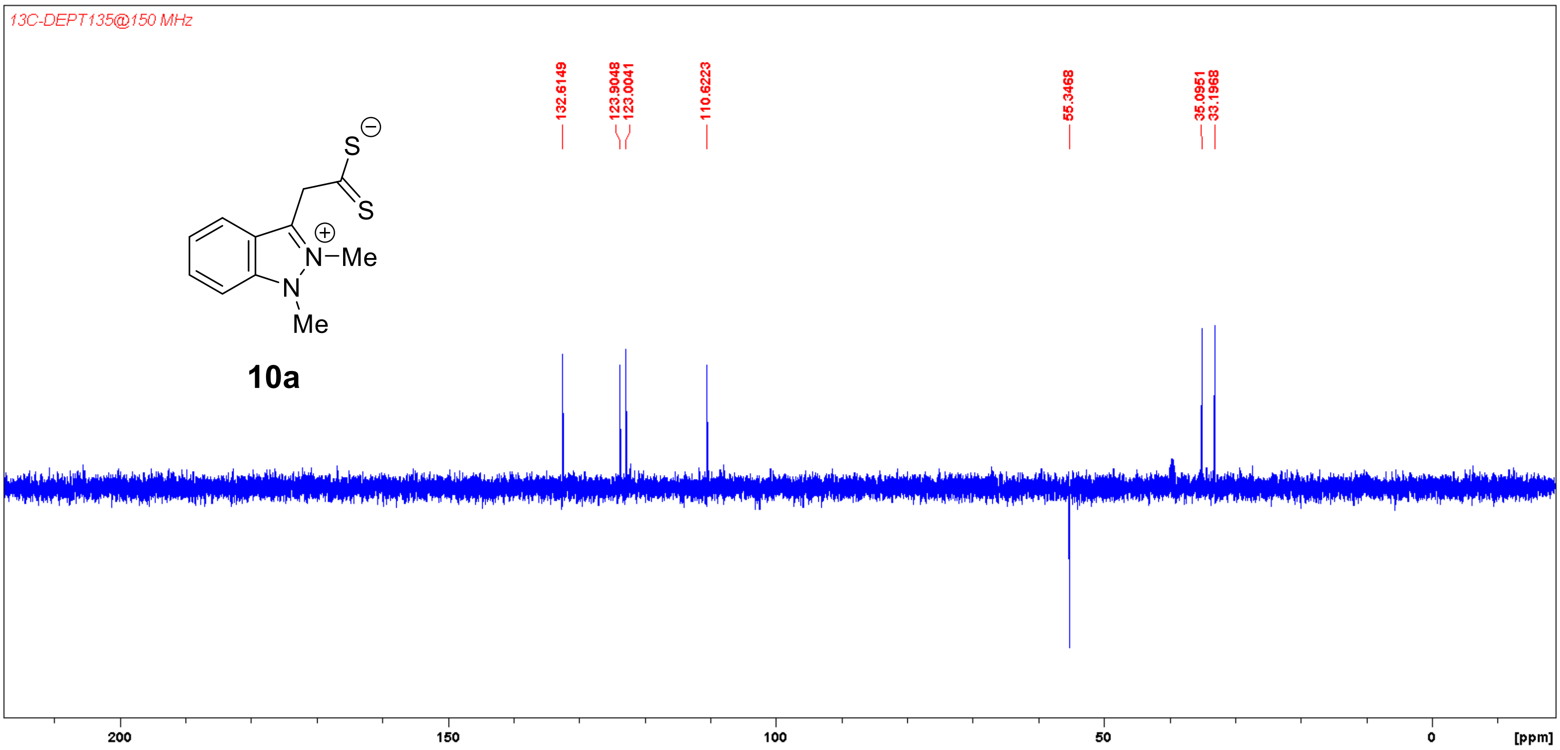

H,H-COSY

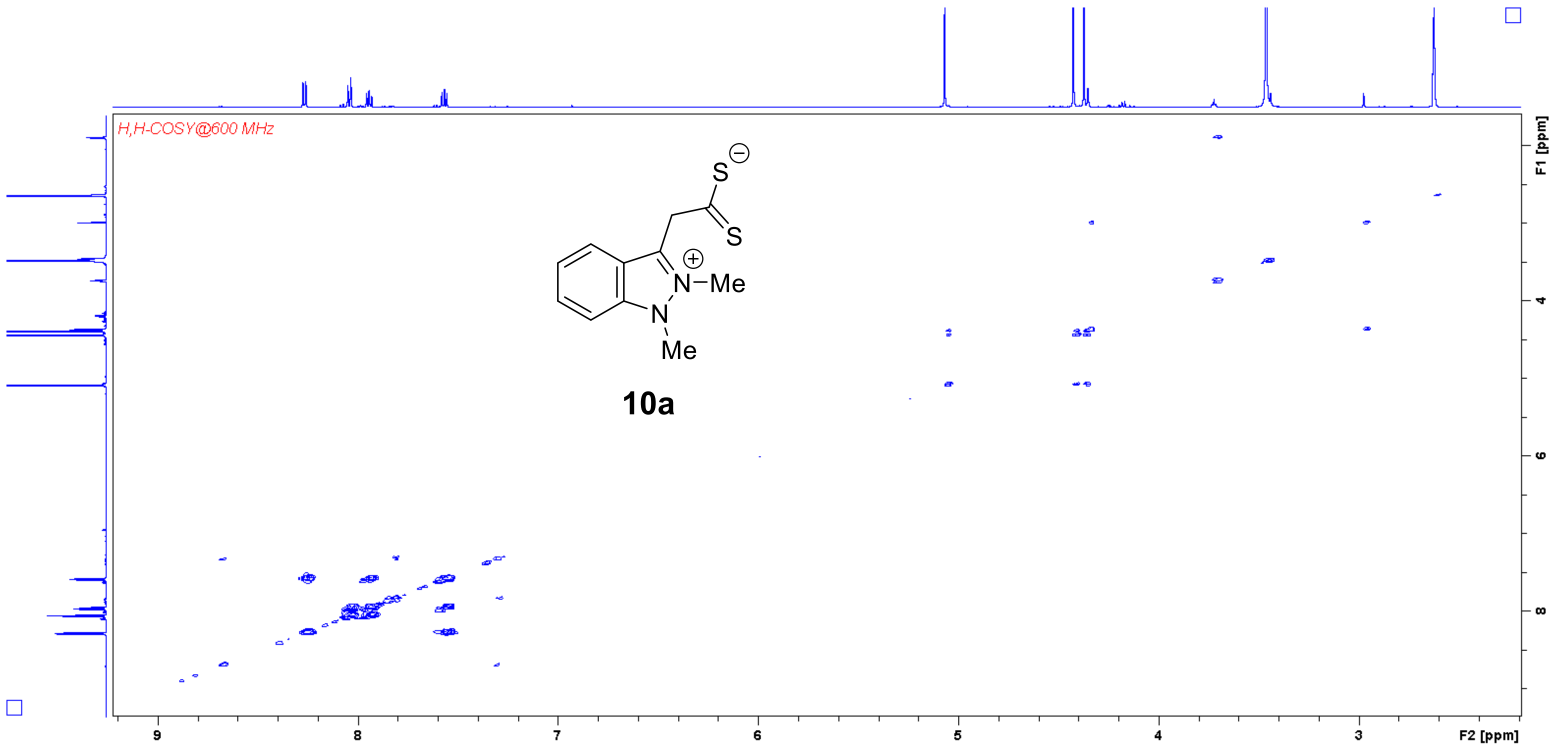
H,H-COSY@600 MHz
10a
F1 [ppm]
F2 [ppm]

HSQC

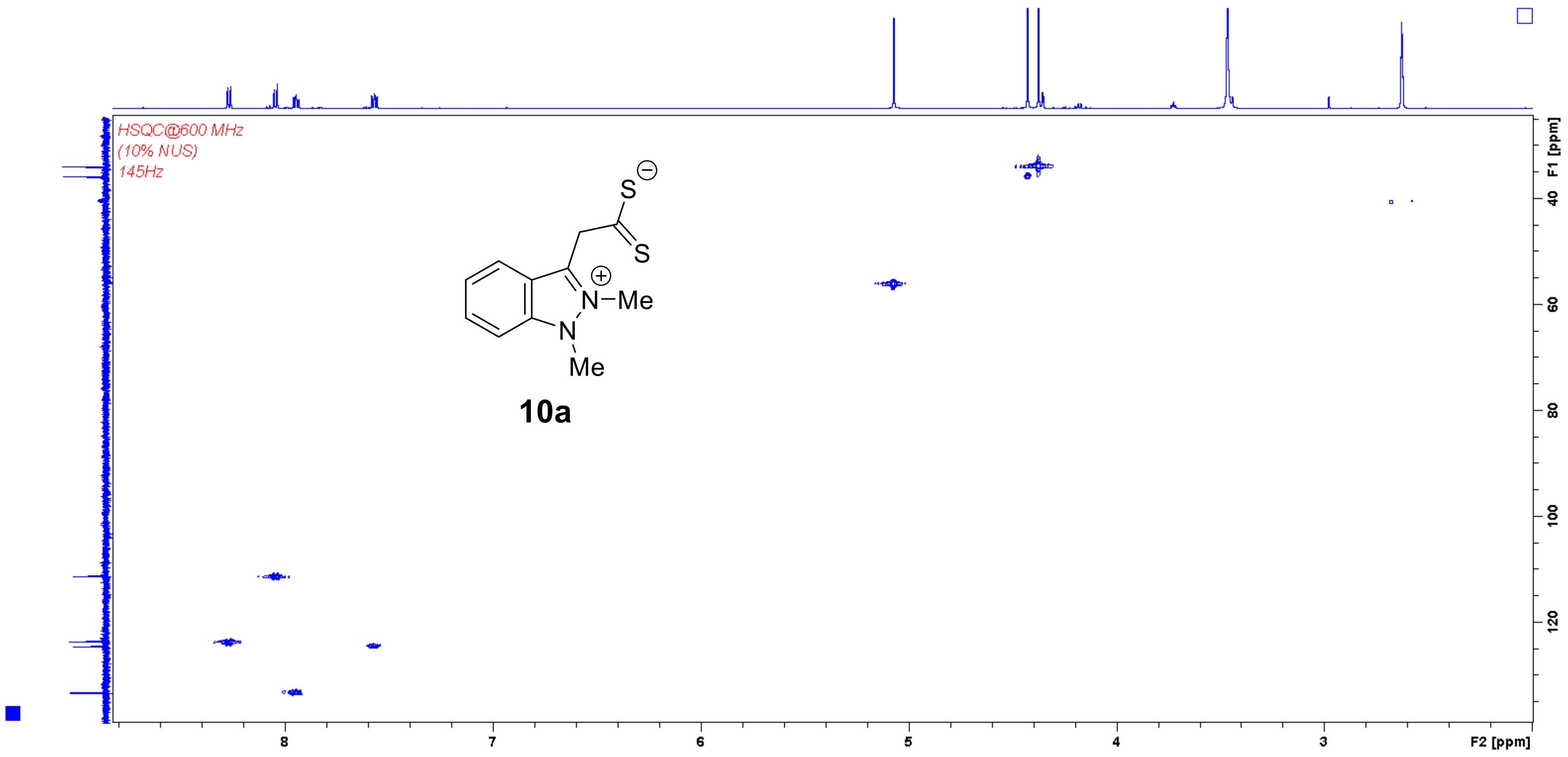
HSQC@600 MHz
(10% NUS)
145Hz
10a
F1 [ppm]
F2 [ppm]

HMBC

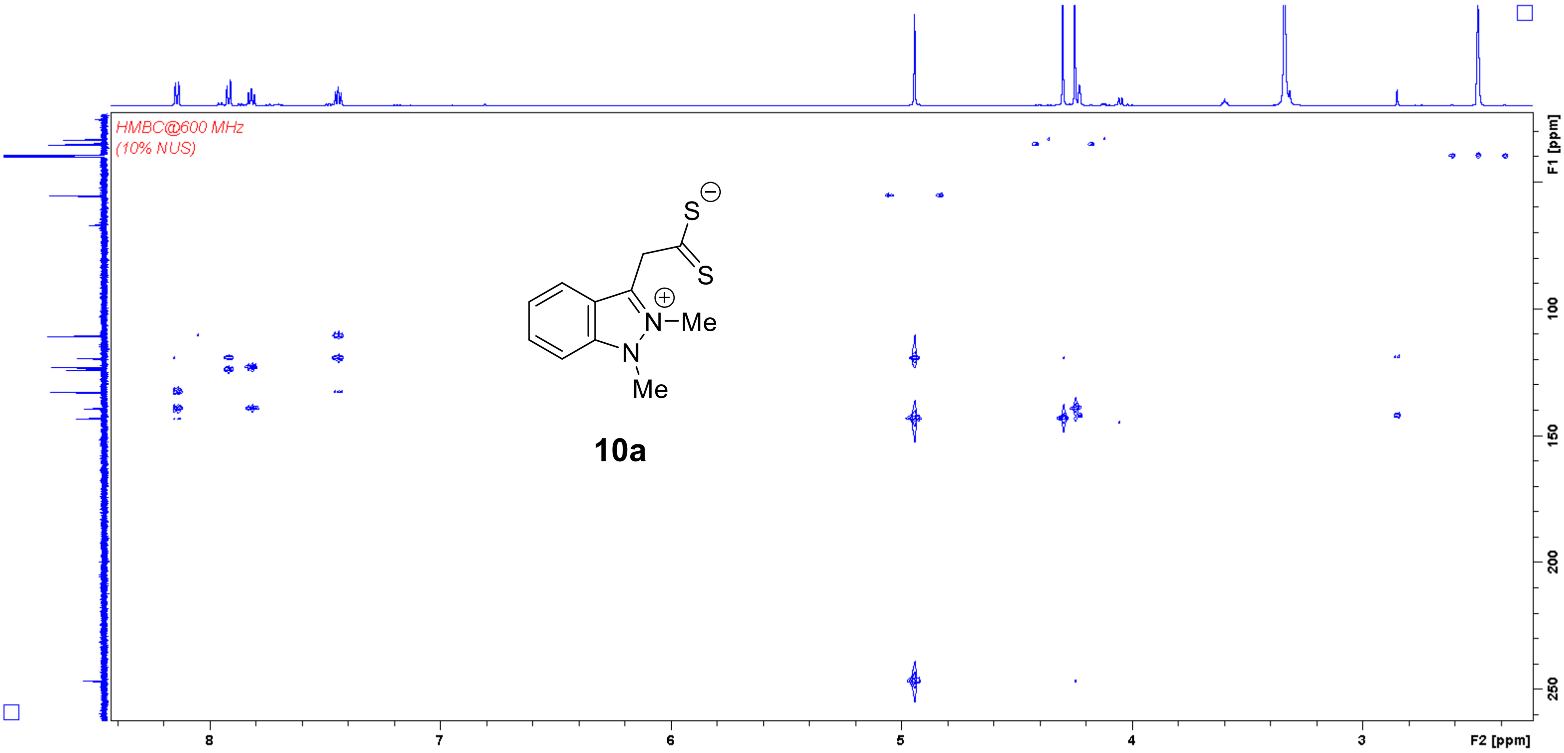
HMBC@600 MHz
(10% NUS)
S
S
N−Me
N
Me
10a
F1 [ppm]
100
150
200
250
8
7
6
5
4
3
F2 [ppm]

Synthesis of 2-(1,2,4,5-tetramethyl-1*H*-pyrazol-2-ium-3-yl)ethanedithioate **10b**

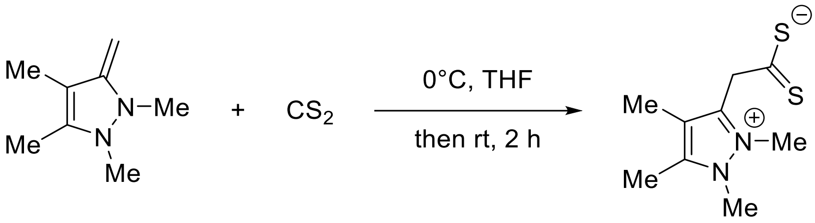

Followed by the general procedure of NHO adduct synthesis, **10b** is prepared from 1,2,3,4,5-pentamethyl-1*H*-pyrazolium iodide **3a** (200 mg, 1 Eq, 0.752 mmol) and carbon disulfide (1 Eq, 57mg, 0.752 mmol) as orange-red solid (80 mg, 50%).

^{1}H-NMR (DMSO-d_6, 600 MHz): 4.39 (s, 2H, CH_2), 3.98 (s, 3H, N-Me), 3.91 (s, 3H, N-Me), 2.35 (s, 3H, Me), 2.02 (s, 3H, Me) ppm.

^{13}C{^{1}H}-NMR (DMSO-d_6, 150 MHz): 247.7 (o, CS_2), 145.2 (o, Ar-C), 142.6 (o, Ar-C), 114.0 (o, Ar-C), 54.7 (-, CH_2), 34.2 (+, N-Me), 33.3 (+, N-Me), 9.6 (+, Me), 7.7 (+, Me) ppm.

ESI-MS (m/z): calculated for $[C_9H_{14}N_2S_2+Na]^+$: 237.0496, found 237.0490.

IR (ATR): $\tilde{\nu}$ = 1218 (C=S stretching) cm^{-1}.

Melting point: 172-174 °C decomposed.

^{1}H-NMR

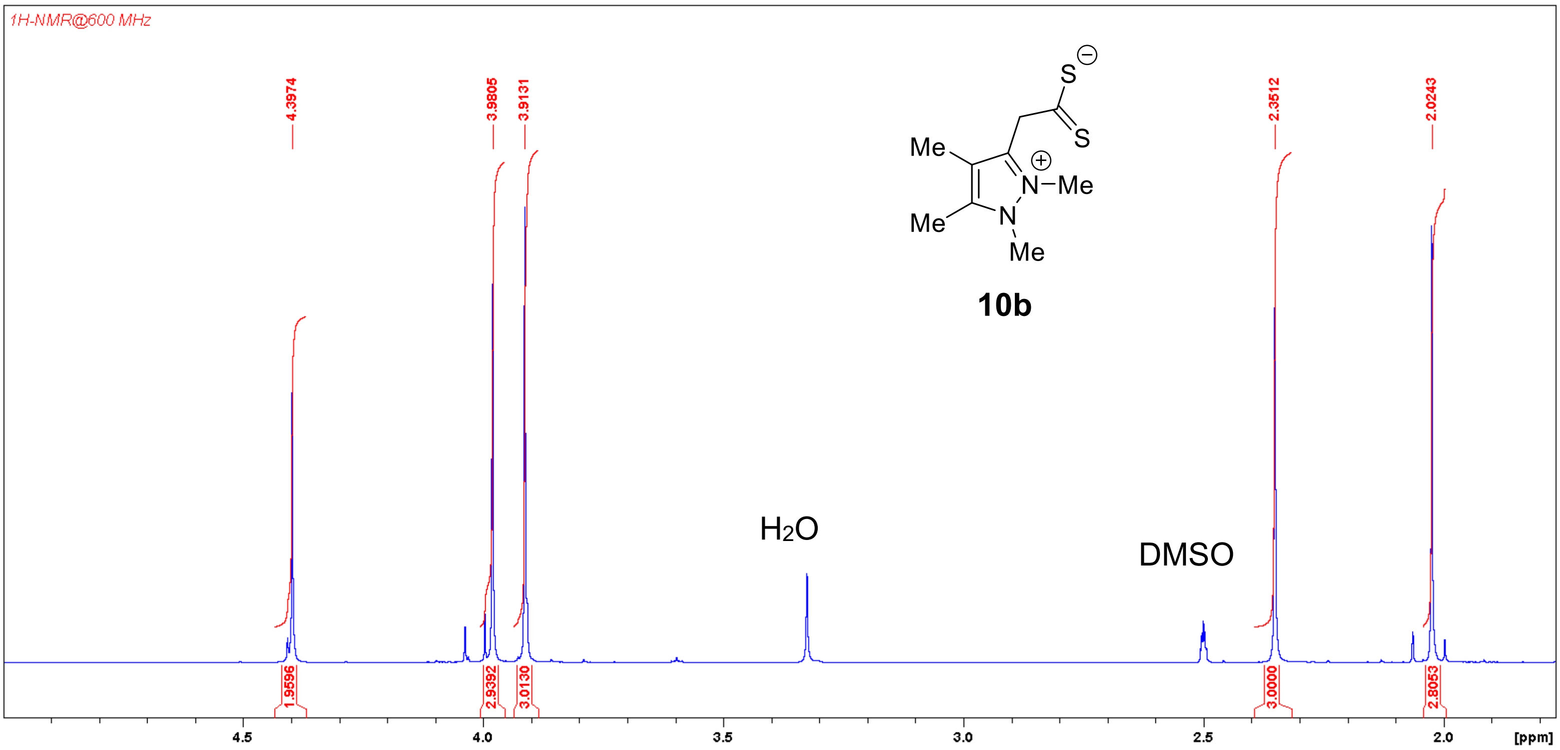

^{13}C{^{1}H}-NMR

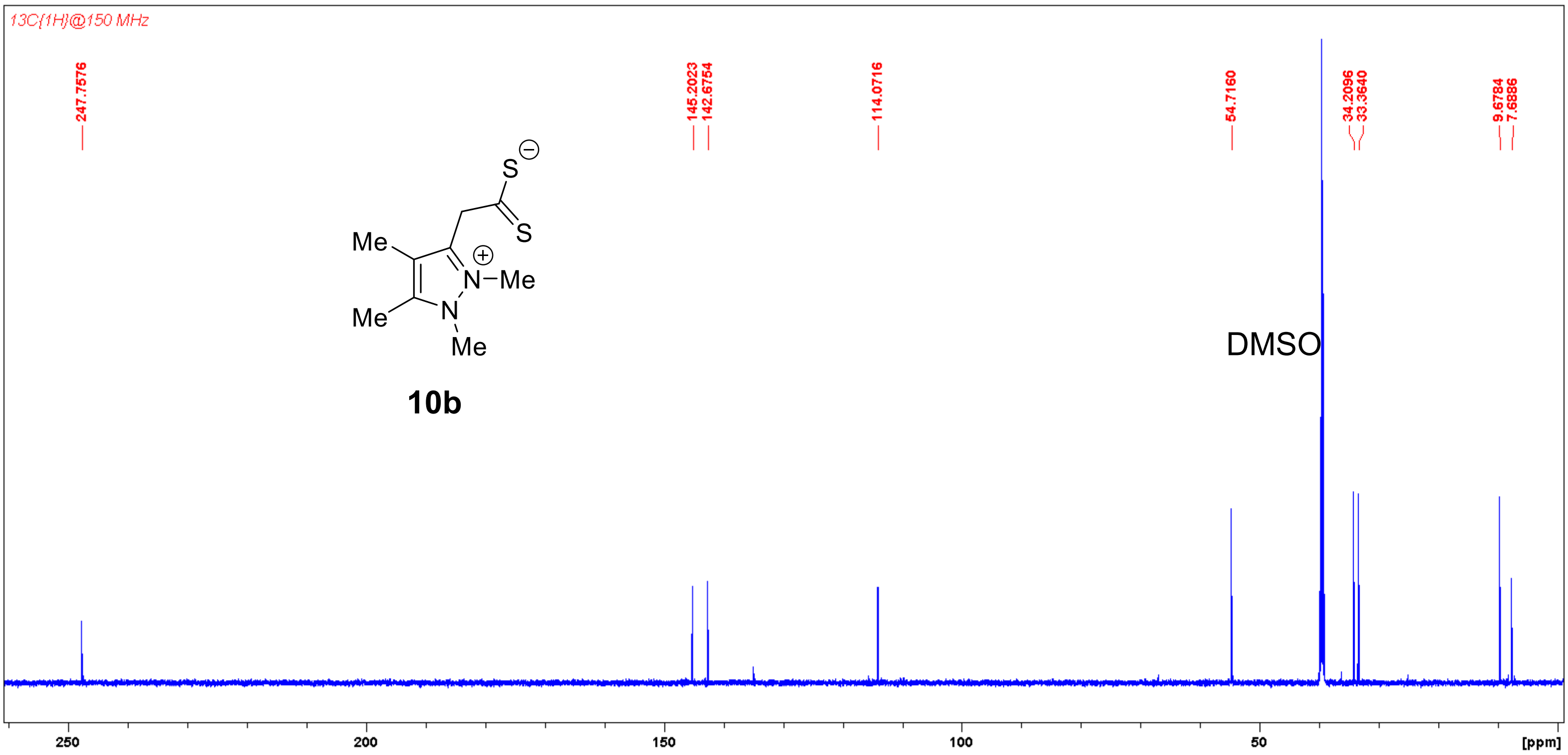

^{13}C-DEPT

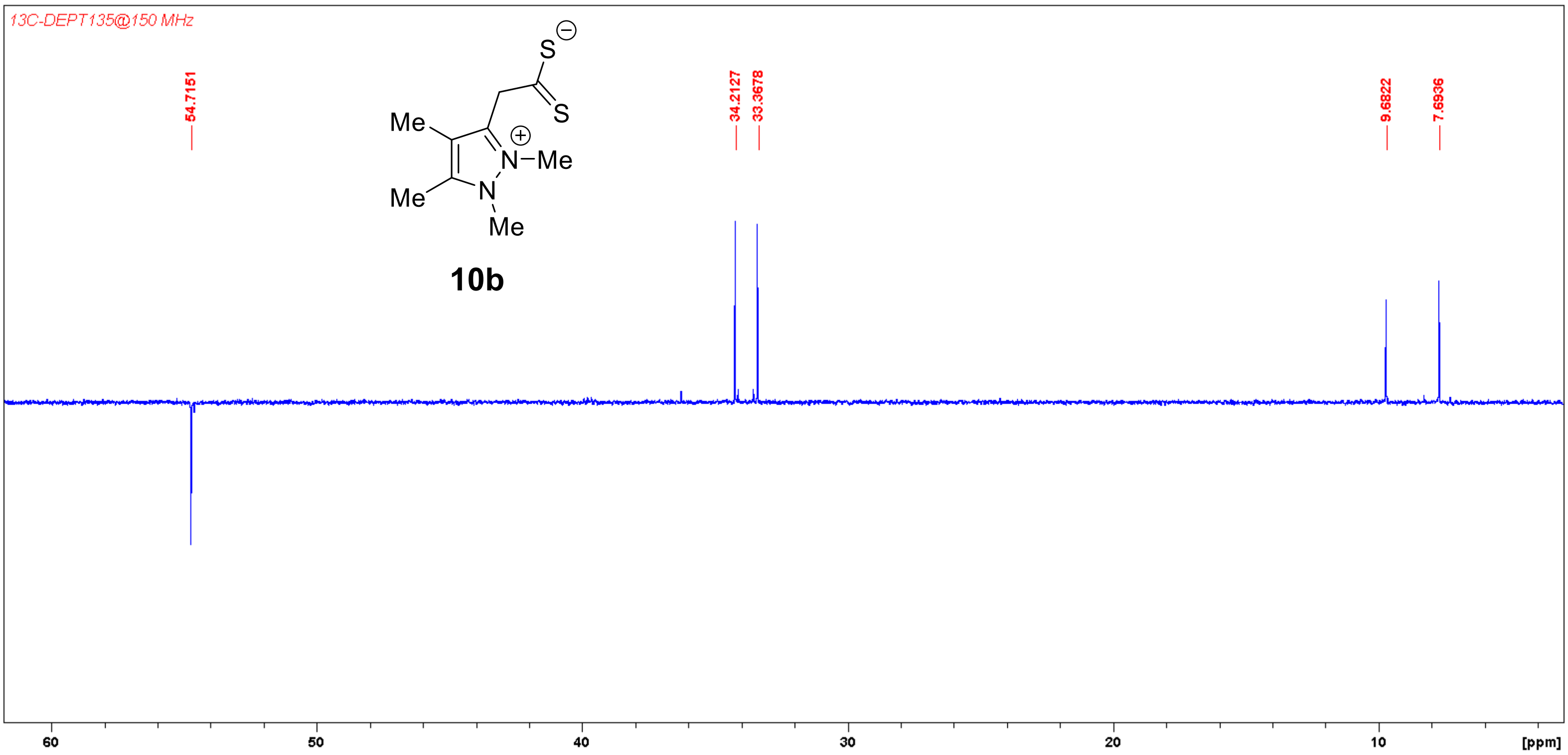

H,H-COSY

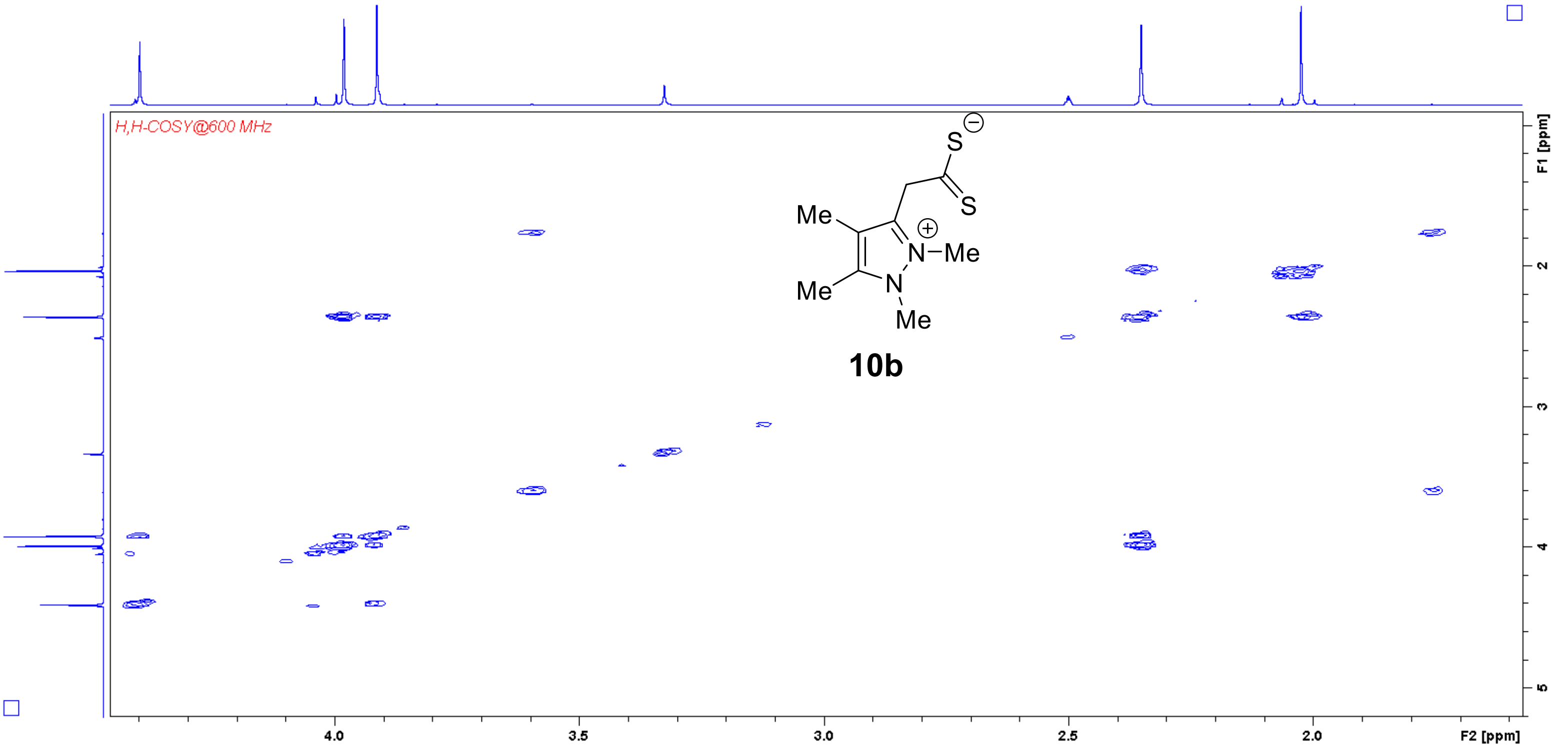

HMBC

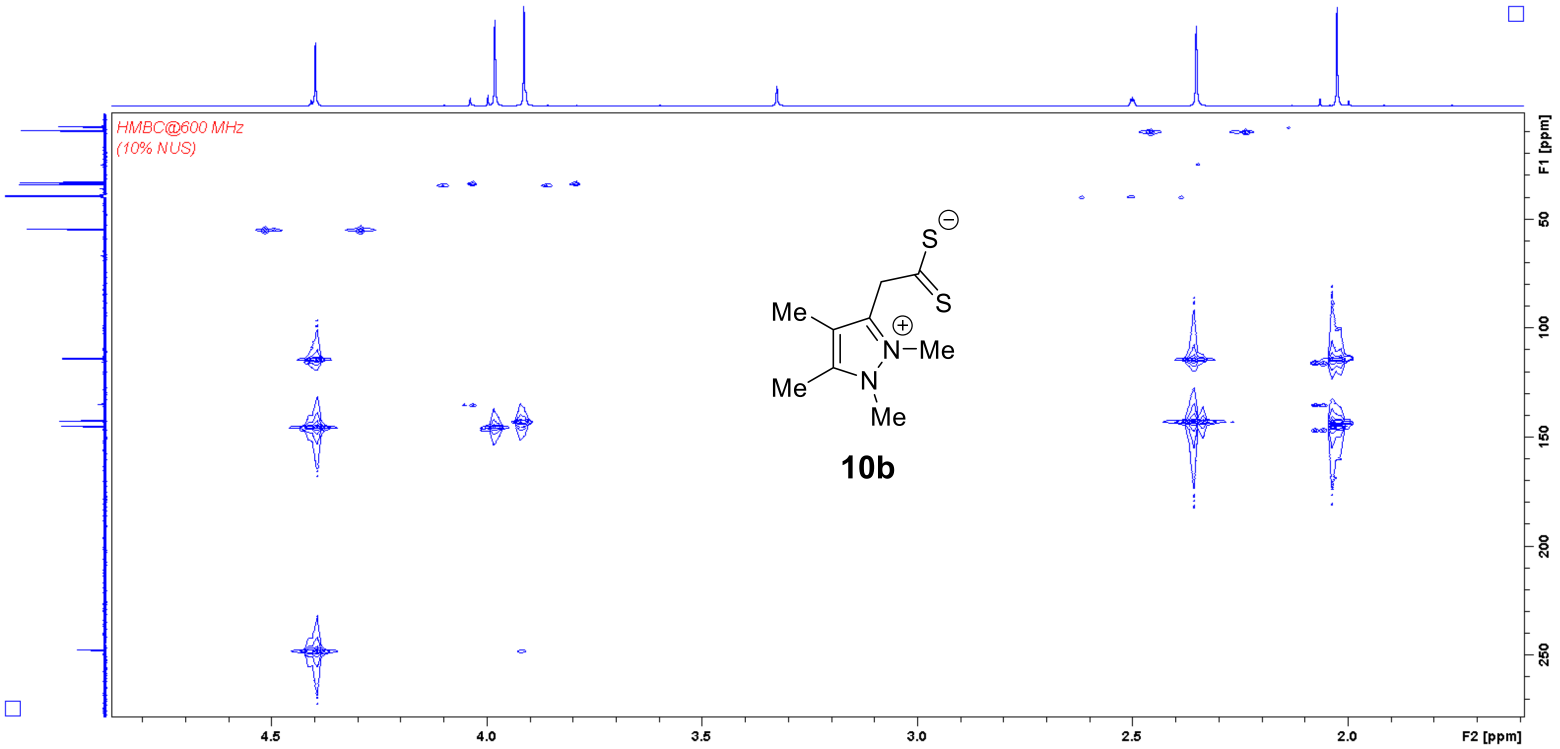
HMBC@600 MHz
(10% NUS)
S
S
Me
N
Me
N
Me
Me
10b
4.5
4.0
3.5
3.0
2.5
2.0
F2 [ppm]
50
100
150
200
250
F1 [ppm]

Synthesis of 2-(1,2,4,5-tetramethyl-1*H*-pyrazol-2-ium-3-yl)ethanedithioate **10c**

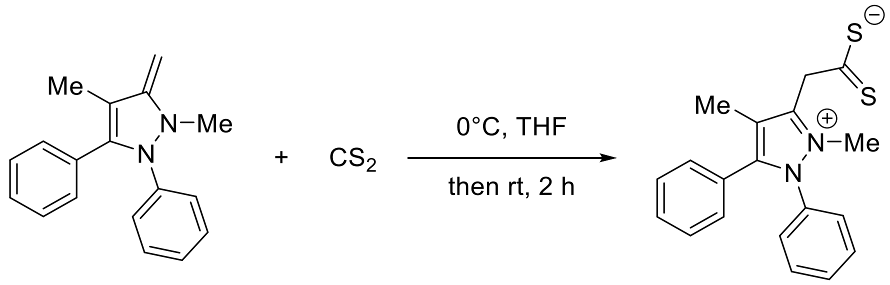

Followed by the general procedure of NHO adduct synthesis, **10c** is prepared from 1,5-diphenyl-2,3,4-trimethyl-1*H*-pyrazolium iodide **3b** (150 mg, 1 Eq, 0.384 mmol) and carbon disulfide (1 Eq, 29 mg, 0.384 mmol) as orange-red solid (65 mg, 50%).

^{1}H-NMR (DMSO-d_6, 600 MHz): 7.62-7.54 (m, 5H, Ar-H), 7.43-7.38 (m, 3H, Ar-H), 7.35-7.32 (m, 2H, Ar-H), 4.66 (s, 2H, 2-H), 3.82 (s, 3H, 1-H), 2.15 (s, 3H, 3-H) ppm.

^{13}C{^{1}H}-NMR (DMSO-d_6, 150 MHz): 246.5 (o, 7-C), 147.8 (o, 6-C), 145.9 (o, 5-C), 132.0 (+, Ar-C), 131.9 (o, Ar-C), 130.3 (+, Ar-C), 130.2 (+, Ar-C), 129.8 (+, Ar-C), 129.5 (+, Ar-C), 128.8 (+, Ar-C), 125.7 (o, Ar-C), 115.9 (o, 4-C), 55.2 (-, 2-C), 35.6 (+, 1-C), 8.4 (+, 3-C) ppm.

ESI-MS (m/z): calculated for $[C_{19}H_{18}N_2S_2+Na]^+$: 361.0809, found 361.0805.

IR (ATR): 1211 (C=S stretching) cm^{-1}.

Melting point: 161-163 °C decomposed.

^{1}H-NMR

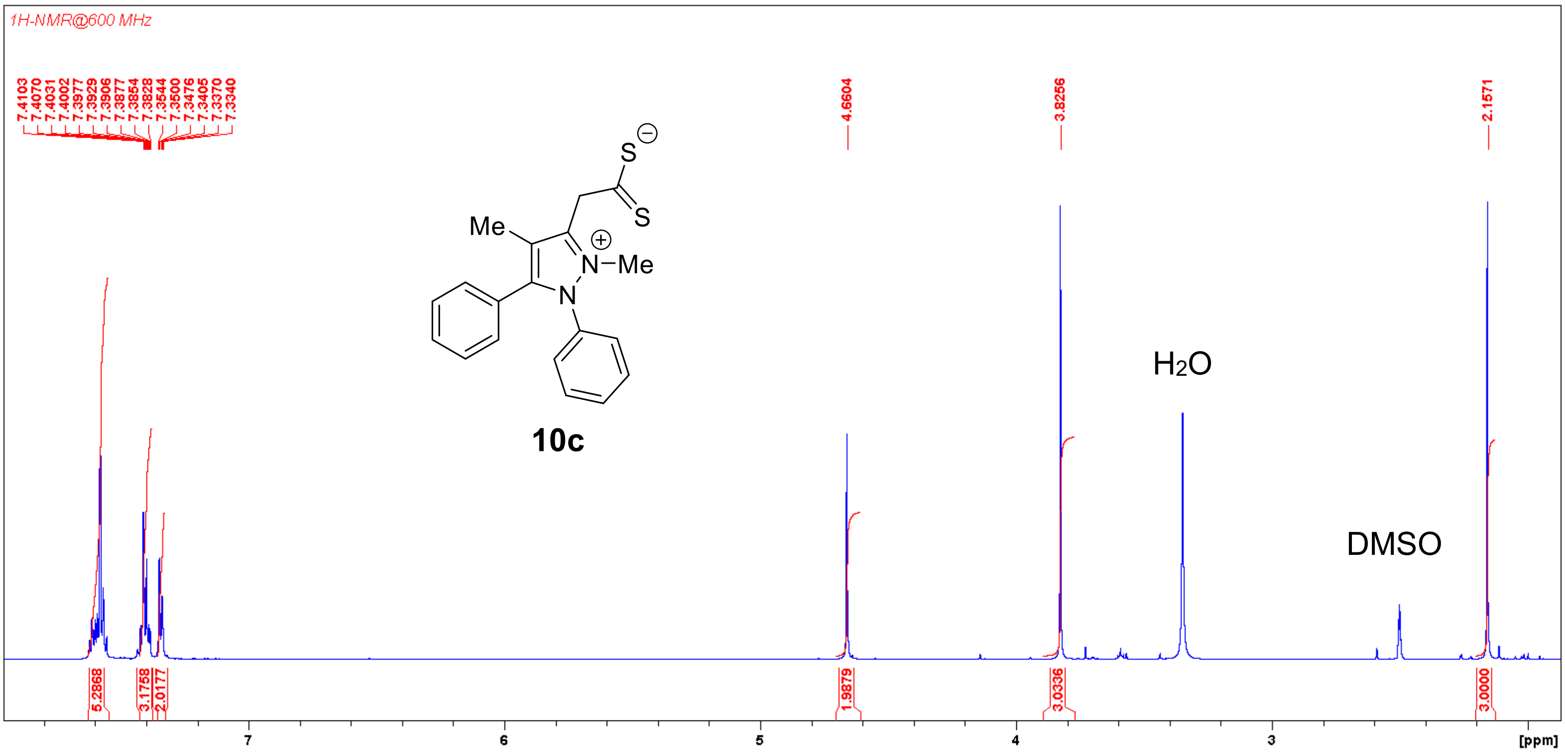

^{13}C{^{1}H}-NMR

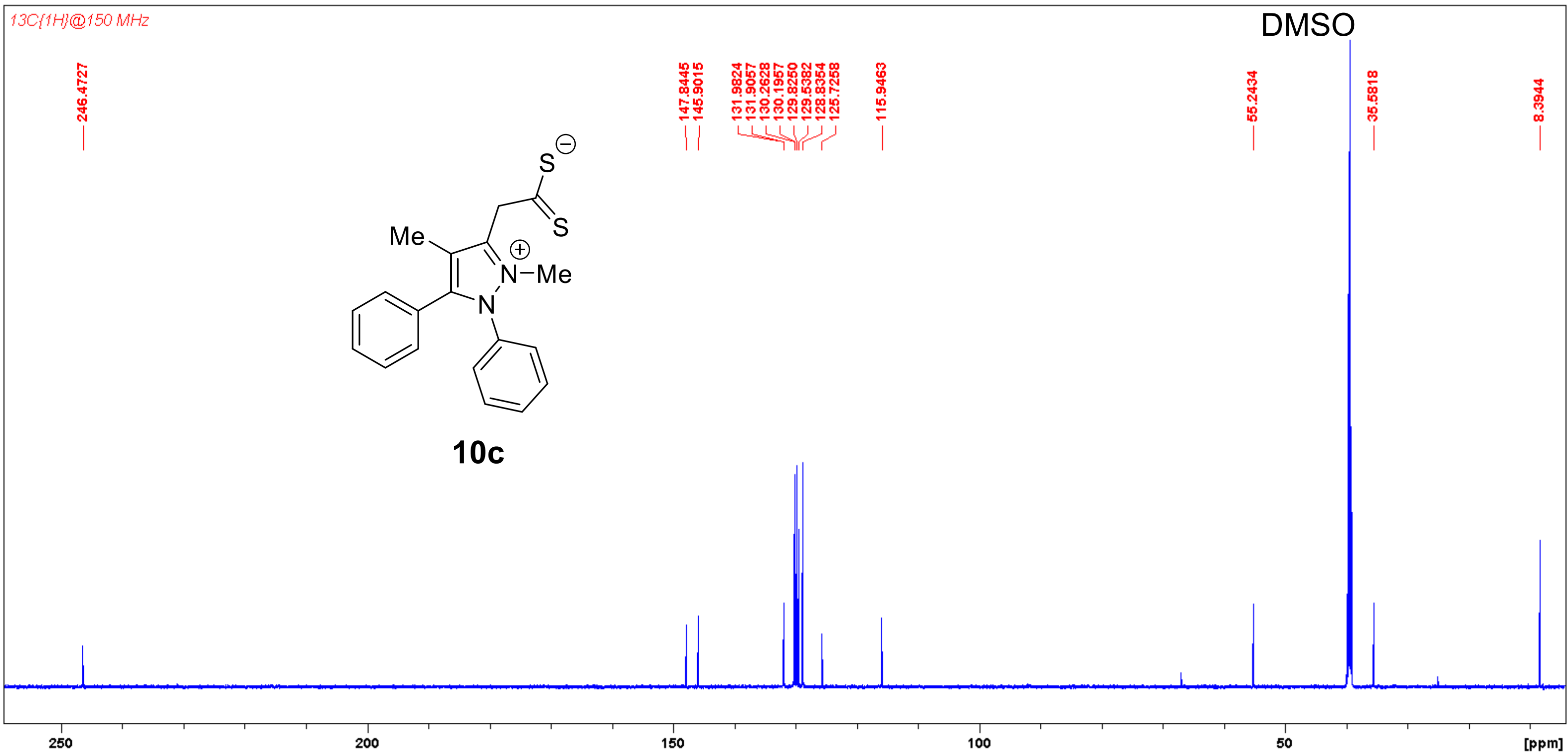

^{13}C-DEPT

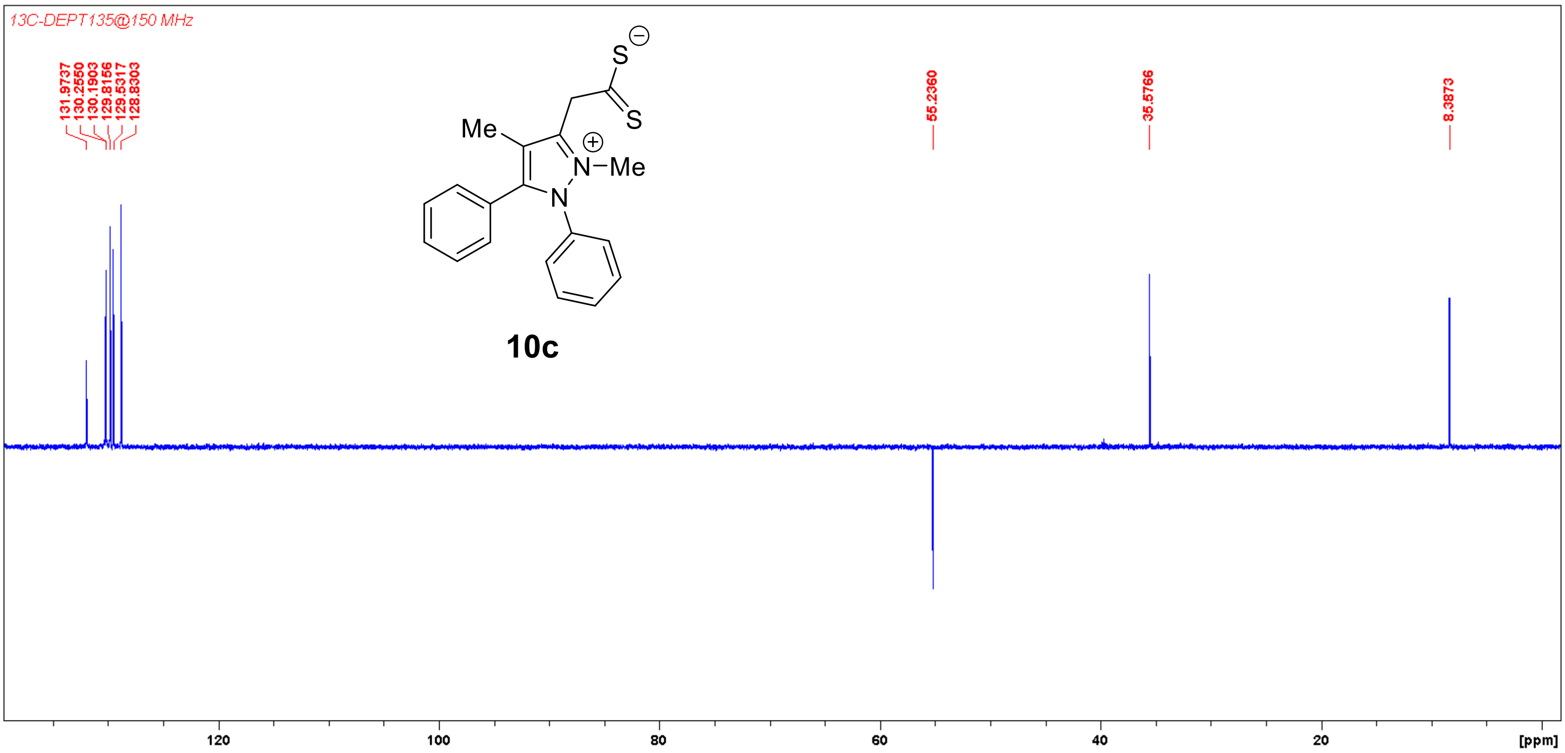

H,H-COSY

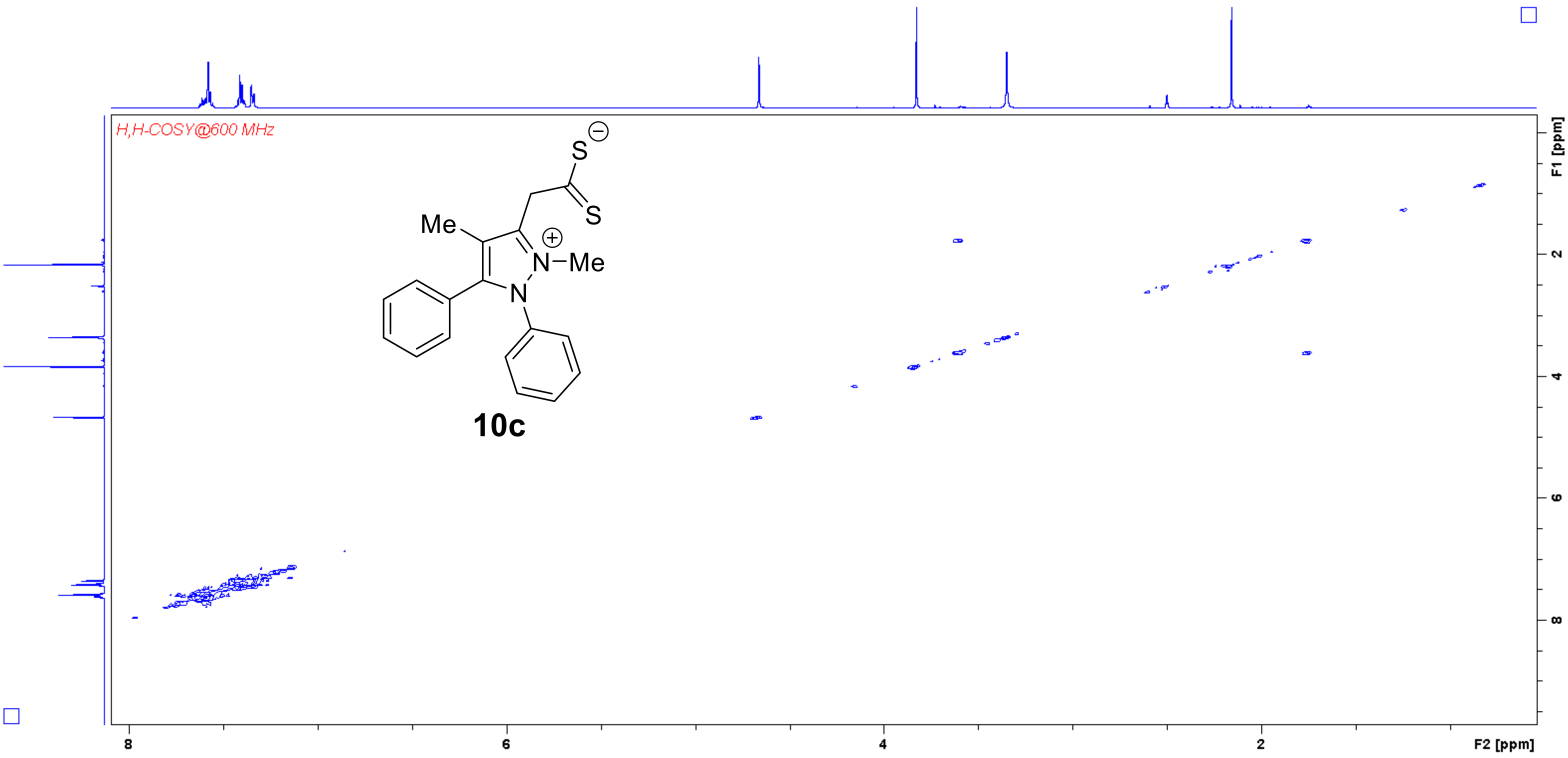

HSQC

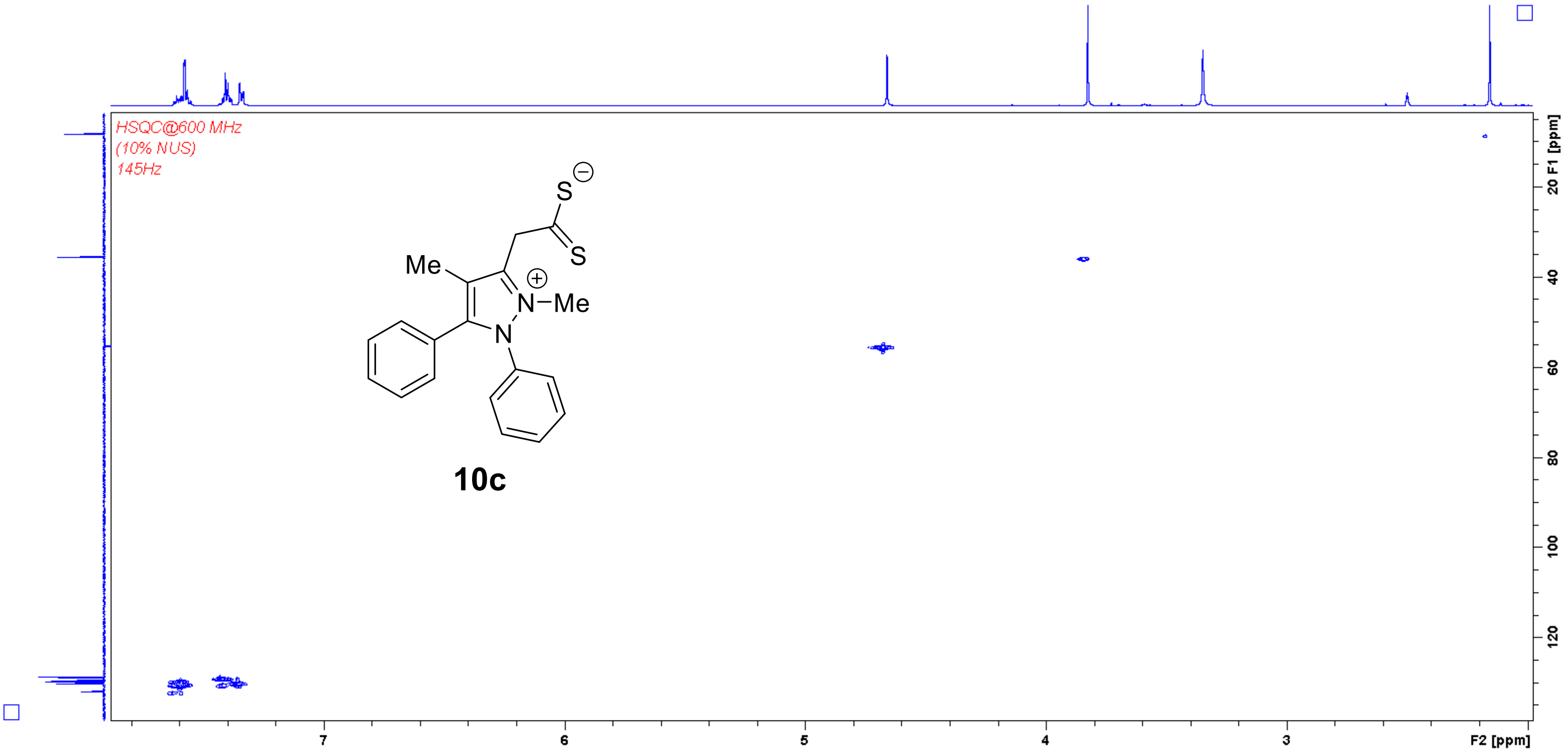
HSQC@600 MHz
(10% NUS)
145Hz
Me
N−Me
N
S
S
10c
F1 [ppm]
F2 [ppm]

HMBC

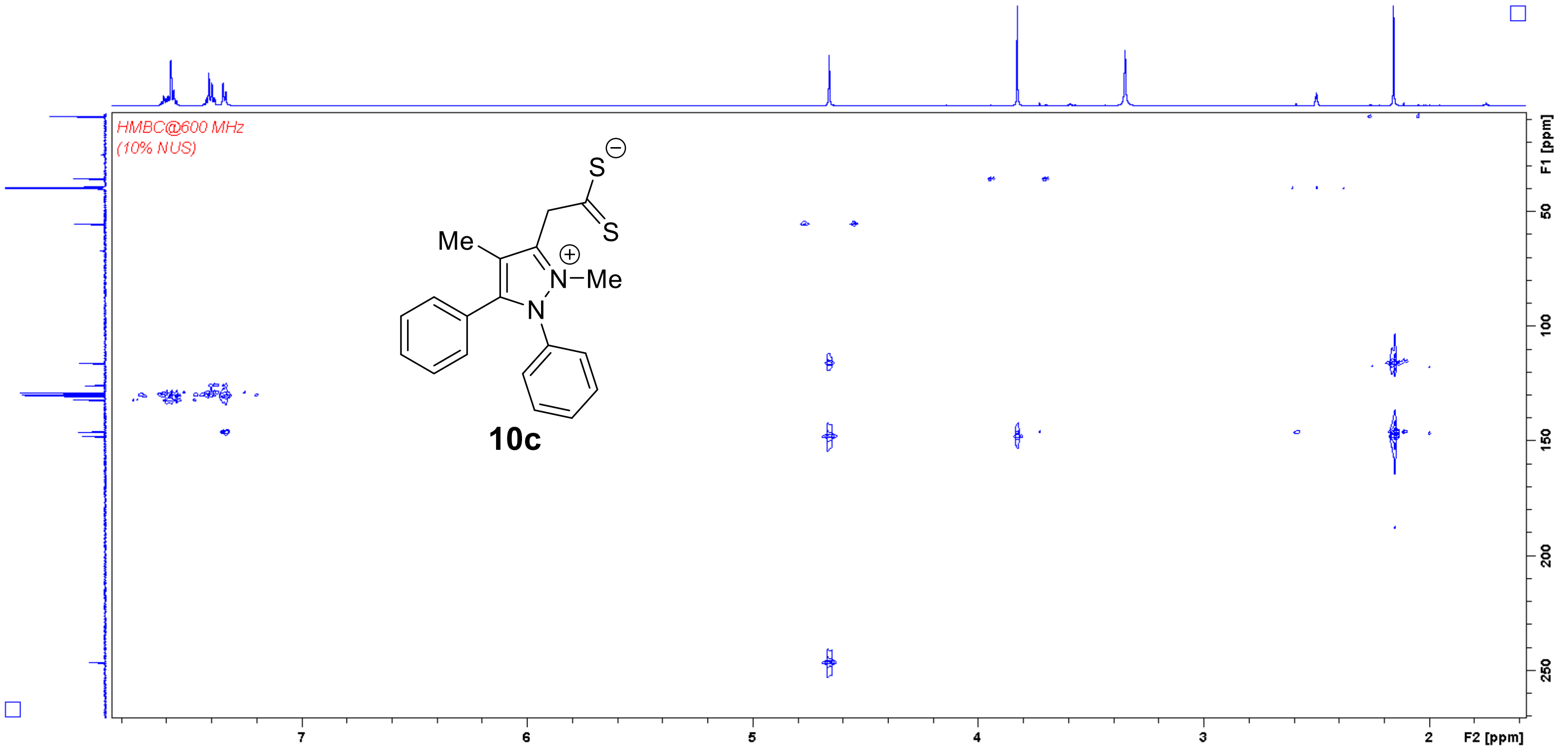
HMBC@600 MHz
(10% NUS)
Me
N−Me
N
S
S
10c
F1 [ppm]
F2 [ppm]

Synthesis of N[1],N[3]-bichlorophenyl-2-(1,2-dimethyl-2,3-dihydro-1*H*-indazol-3-ylidene)malonamide **11a**.

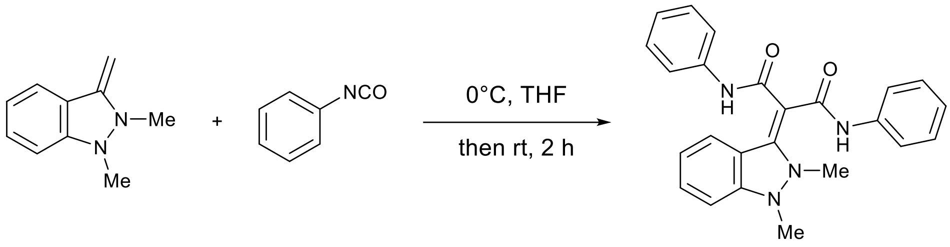

Followed by the general procedure of NHO adduct synthesis, **11a** is prepared from NHO **2a** (54 mg, 1 Eq, 0.34 mmol) and phenyl isocyanate (81 mg, 2 Eq, 0.68 mmol) as light yellow solid (55 mg, 41%).

^{1}H-NMR (DMSO-d$_6$, 600 MHz): 7.82 (d, J = 8.8 Hz, 1H, Ar-H), 7.69-7.66 (m, 2H, Ar-H), 7.43 (d, J = 7.8 Hz, 4H, Ar-H), 7.22 (td, J = 7.8, 1.0 Hz, 1H, Ar-H), 7.17 (t, J = 7.8 Hz, 4H, Ar-H), 6.84 (tt, J = 7.8, 1.0 Hz, 2H, Ar-H), 4.16 (s, 3H, N-Me), 3.95 (s, 3H, N-Me) ppm.

^{13}C{^{1}H}-NMR (DMSO-d$_6$, 150 MHz): 166.4 (o, C=O), 147.8 (o, Ar-C), 141.3 (o, Ar-C), 140.1 (o, Ar-C), 131.2 (+, Ar-C), 128.3 (+, Ar-C), 124.0 (+, Ar-C), 122.3 (+, Ar-C), 120.9 (o, Ar-C), 120.5 (+, Ar-C), 118.7 (+, Ar-C), 110.2 (+, Ar-C), 73.5 (o, C=indazole), 35.8 (+, N-Me), 33.5 (+, N-Me) ppm.

ESI-MS (m/z): calculated for $[C_{24}H_{22}N_4O_2+Na]^+$: 421.1635, found 421.1638.

IR (ATR): $\tilde{\nu}$ = 1508 (amide) cm^{-1}.

Melting point: 197-199 °C, decomposed.

^{1}H-NMR

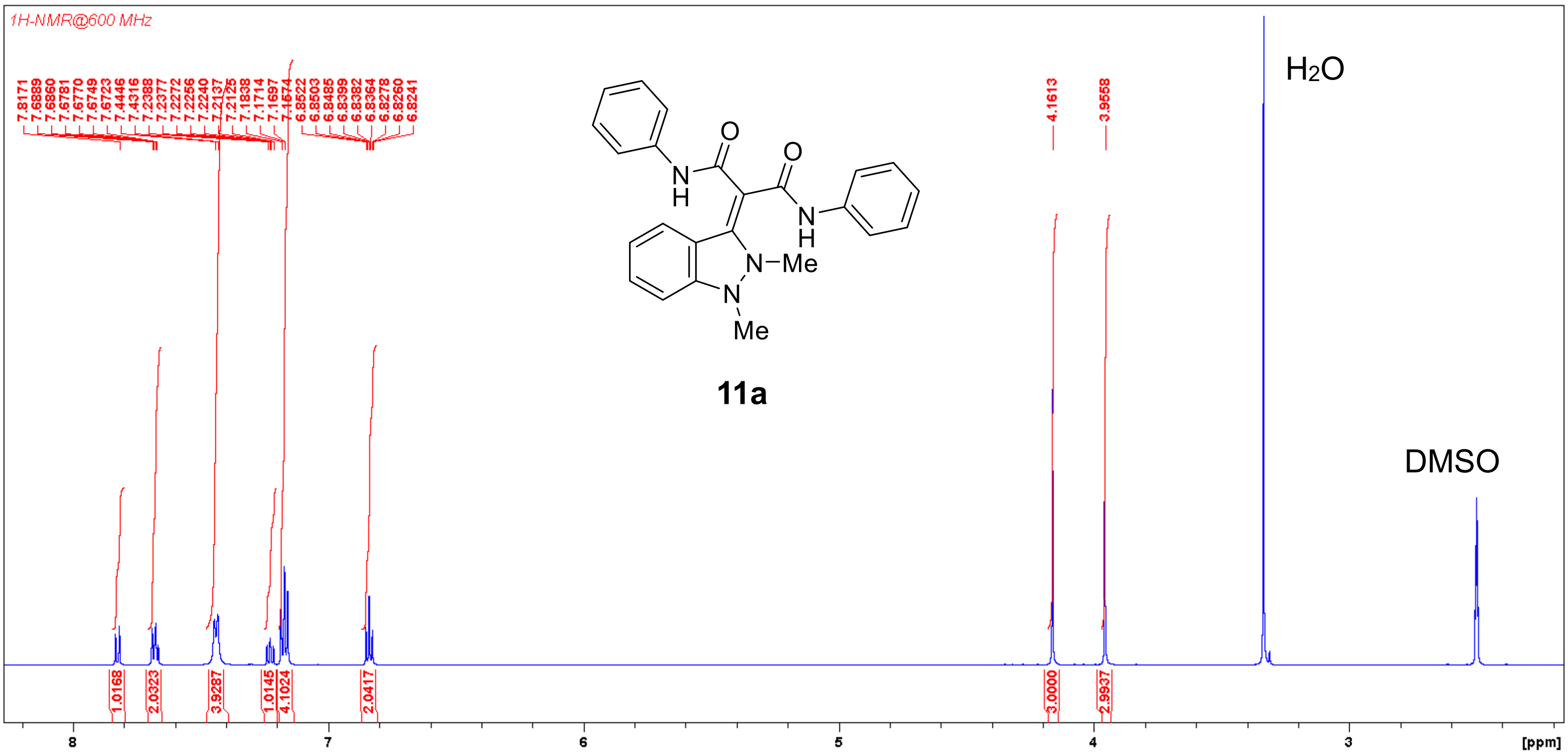
1H-NMR@600 MHz
H2O
DMSO
O
O
N
H
N
H
N−Me
N
Me
11a
[ppm]

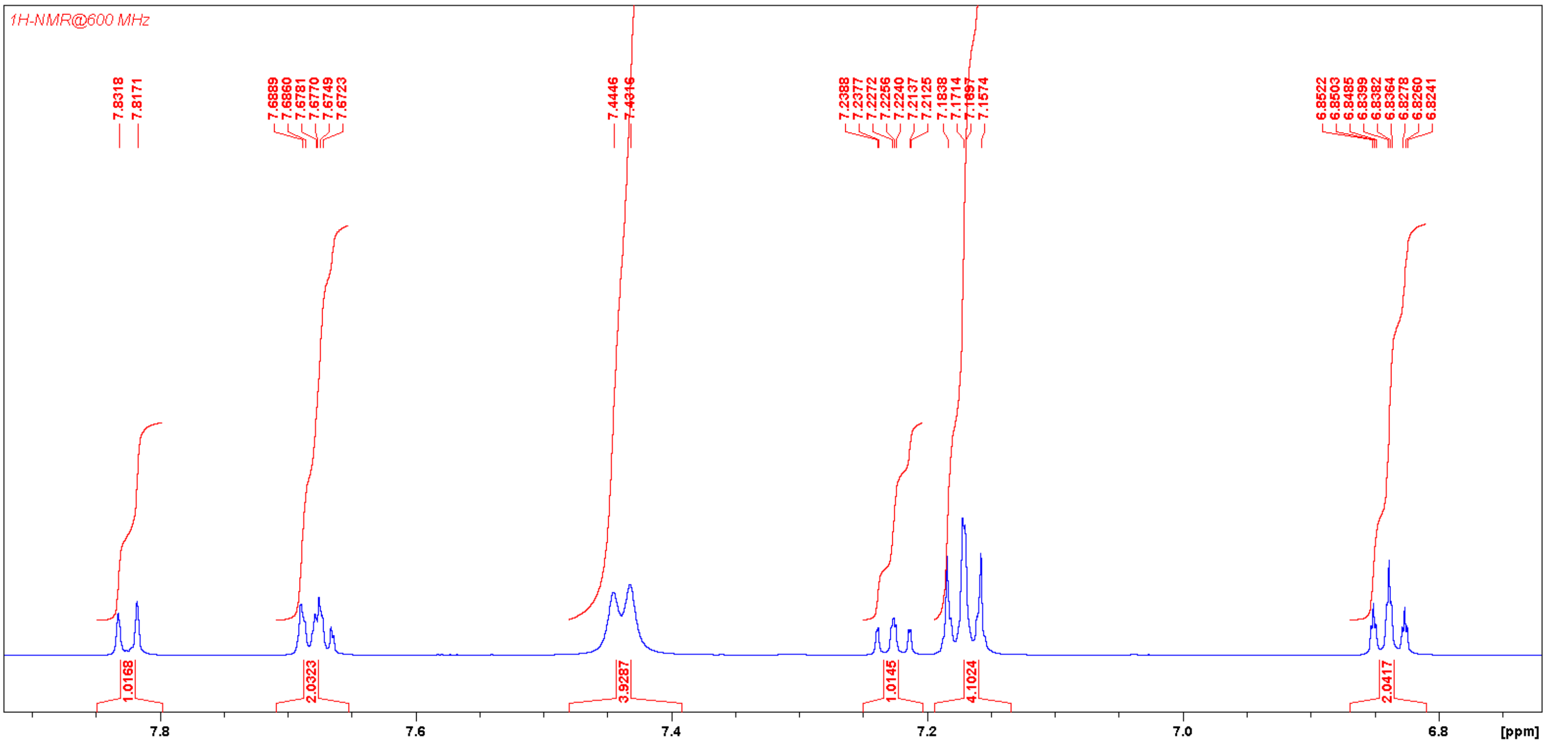
1H-NMR@600 MHz
[ppm]

$^{13}C\{^{1}H\}$-NMR

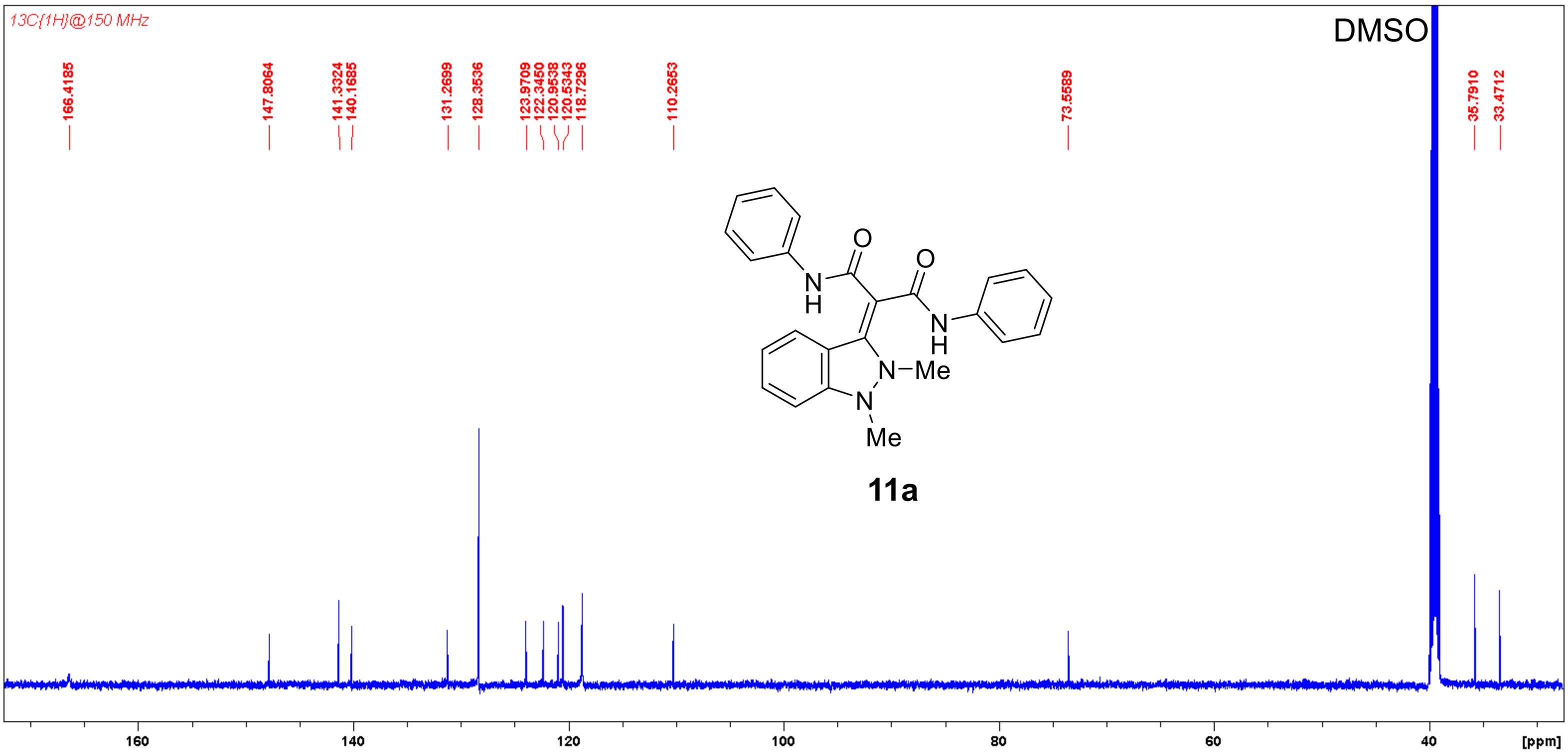

^{13}C-DEPT

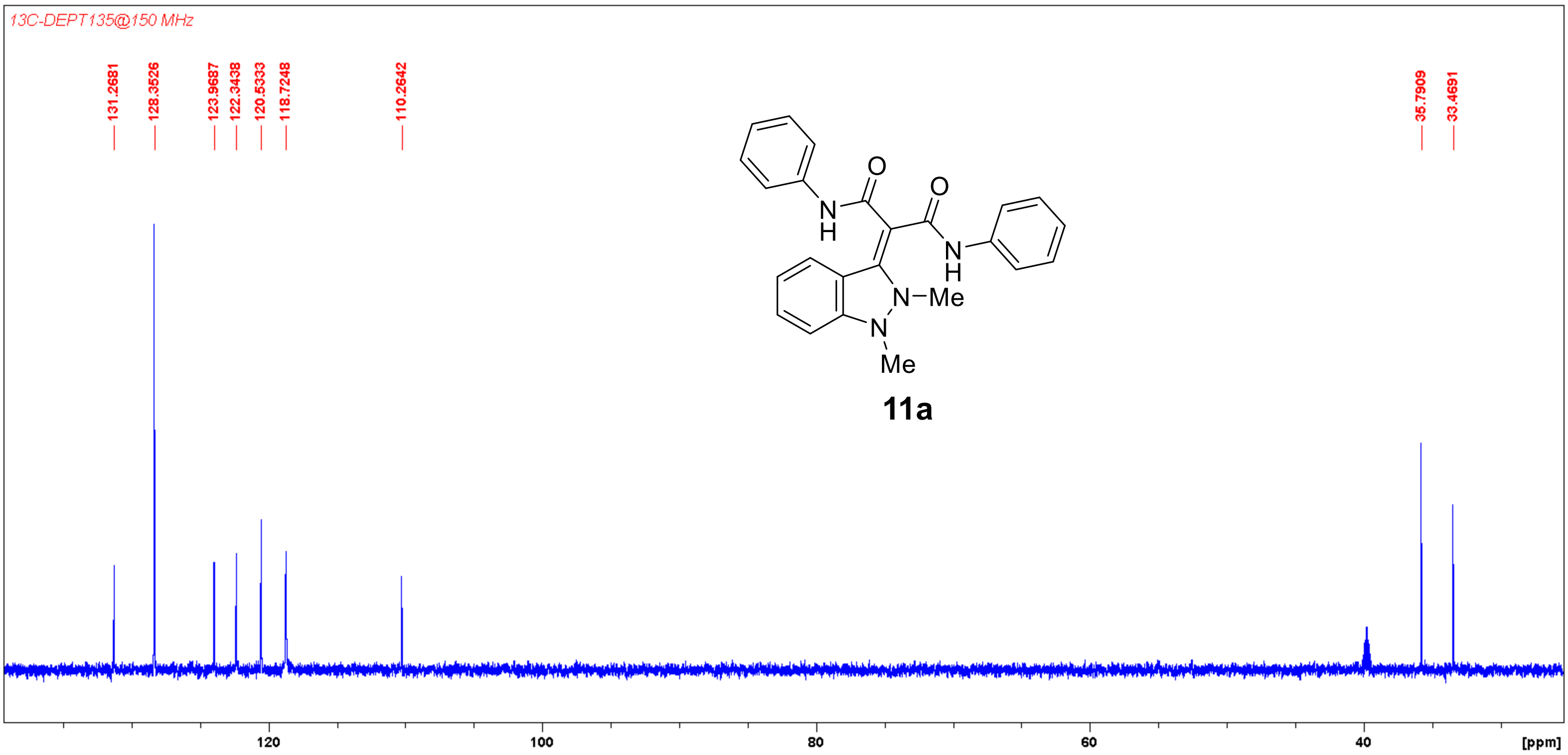

H,H-COSY

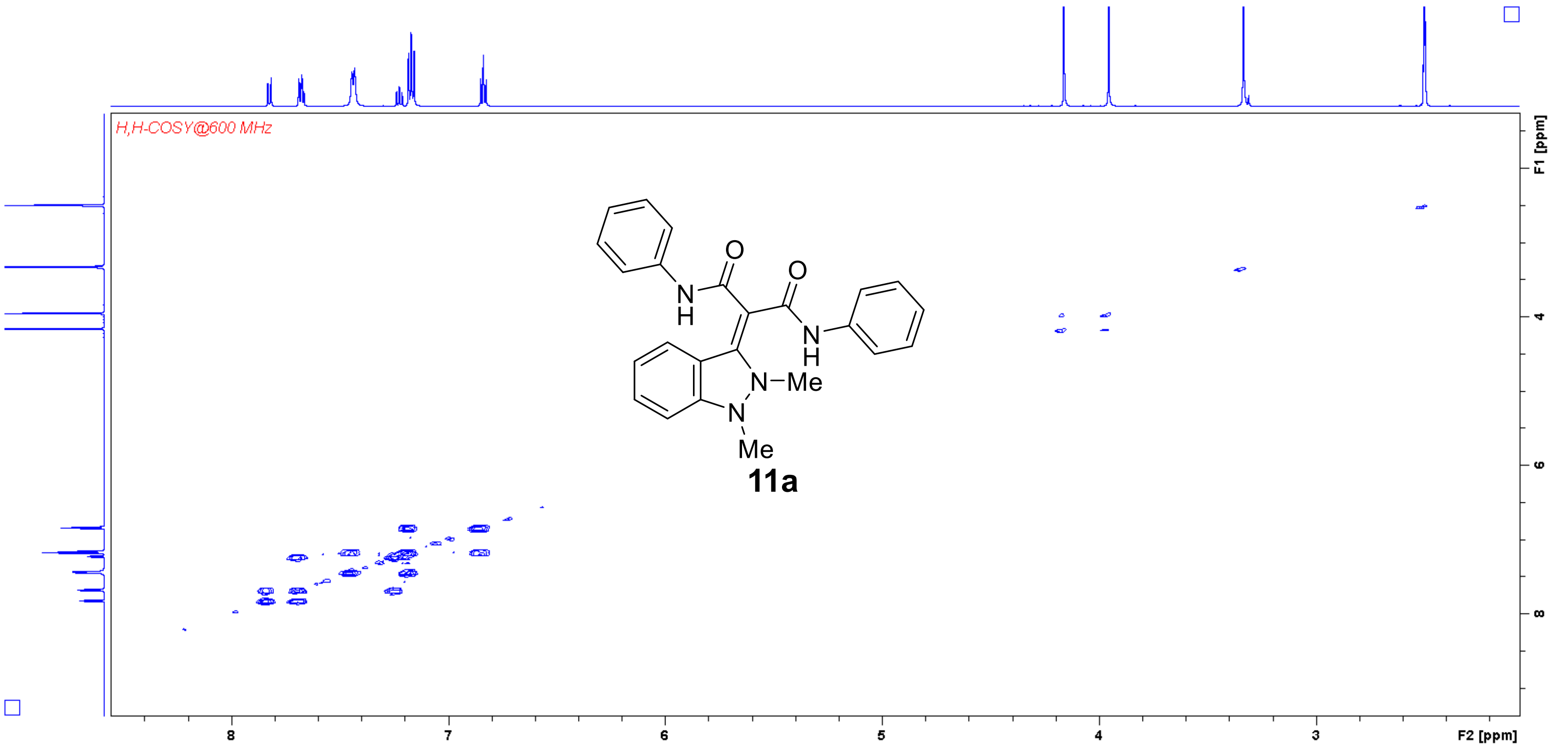
H,H-COSY@600 MHz
O
N
H
O
N
H
N−Me
N
Me
11a
F1 [ppm]
F2 [ppm]
8
7
6
5
4
3
4
6
8

HSQC

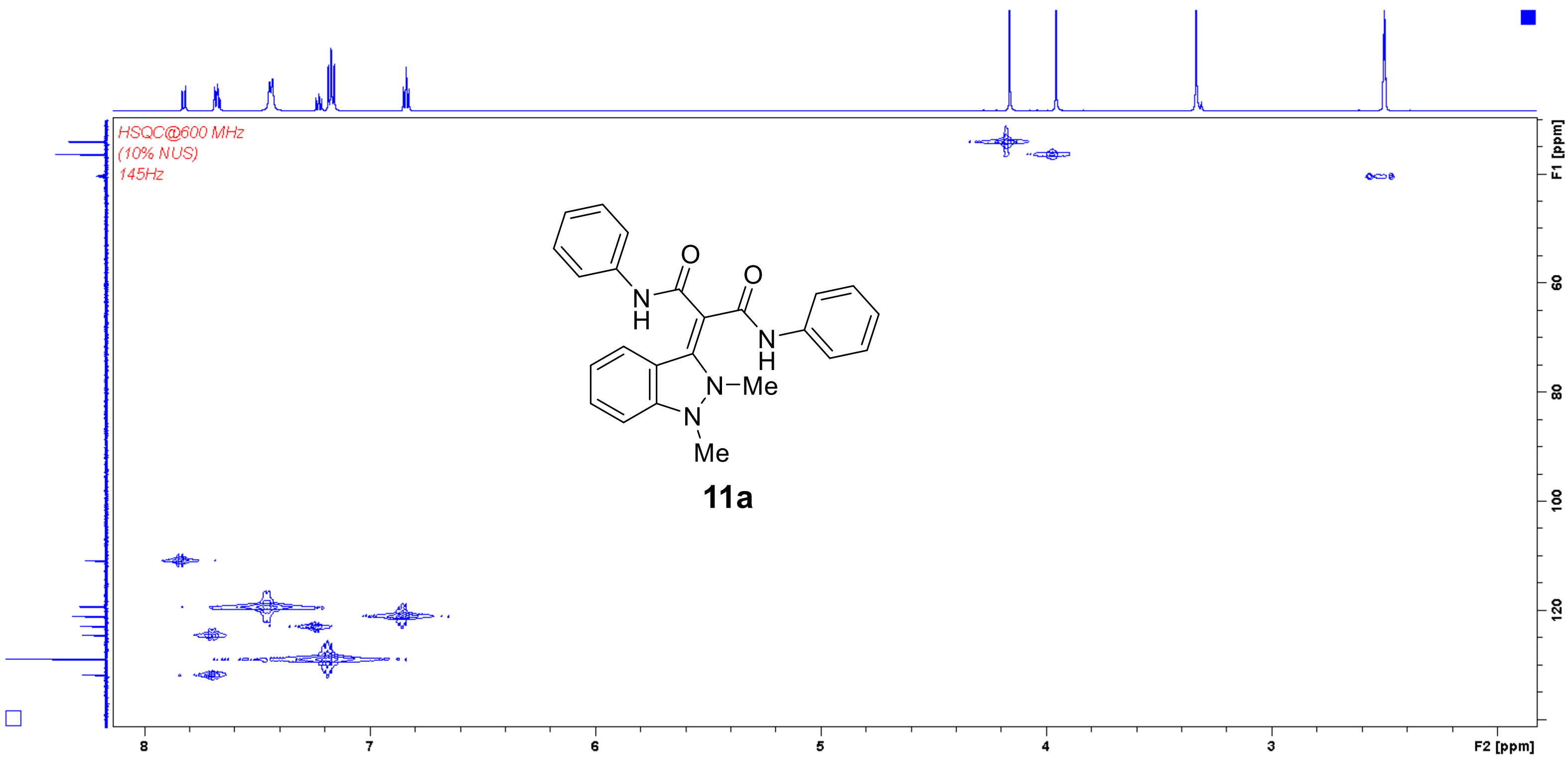
HSQC@600 MHz
(10% NUS)
145Hz
O
N
H
O
N
H
N−Me
N
Me
11a
F1 [ppm]
F2 [ppm]
8
7
6
5
4
3
60
80
100
120

HMBC

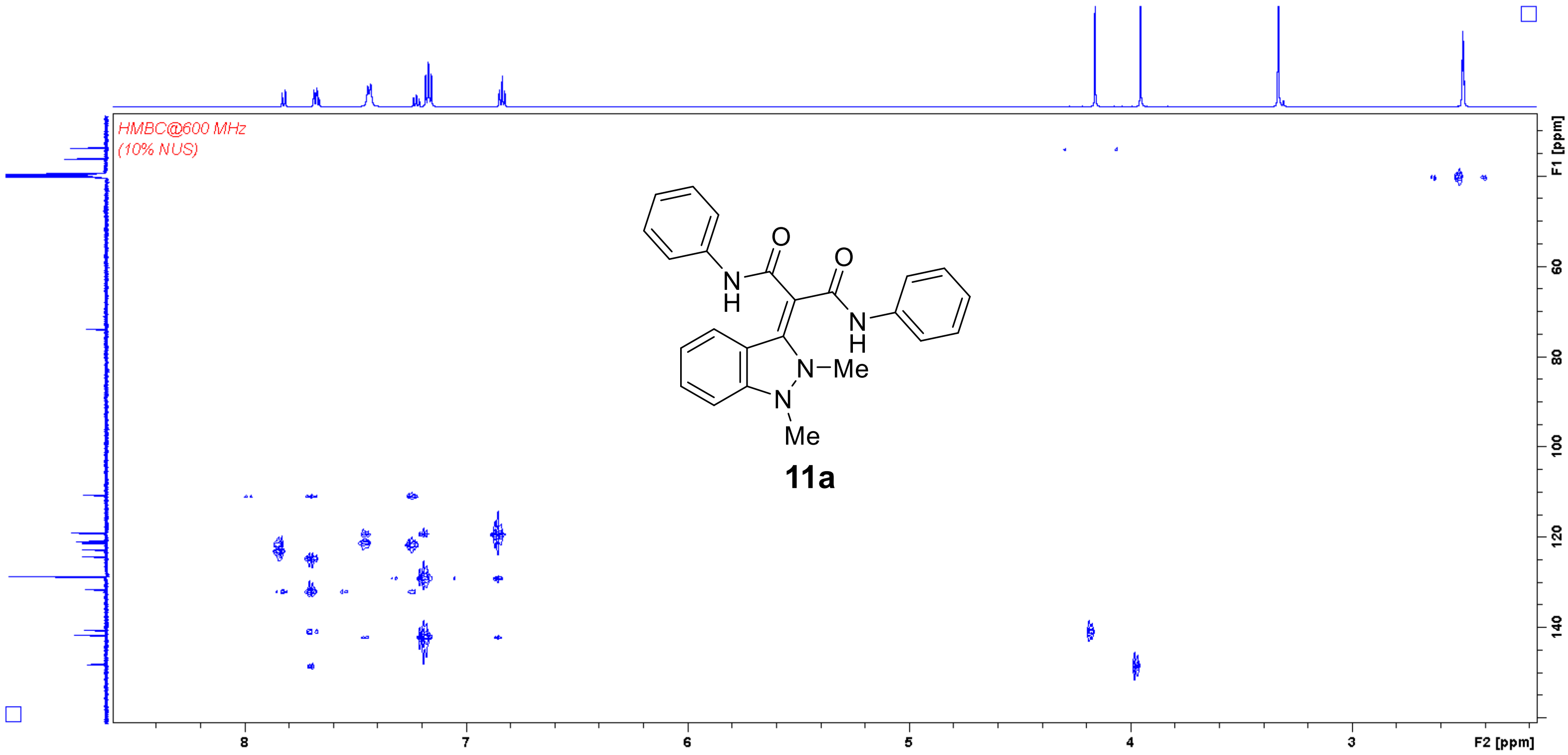
HMBC@600 MHz
(10% NUS)
O
O
N
H
N
H
N–Me
N
Me
11a
F1 [ppm]
60
80
100
120
140
8
7
6
5
4
3
F2 [ppm]

Synthesis of N^1,N^3-bis(4-chlorophenyl)-2-(1,2-dimethyl-2,3-dihydro-1*H*-indazol-3-ylidene)malonamide **11b**

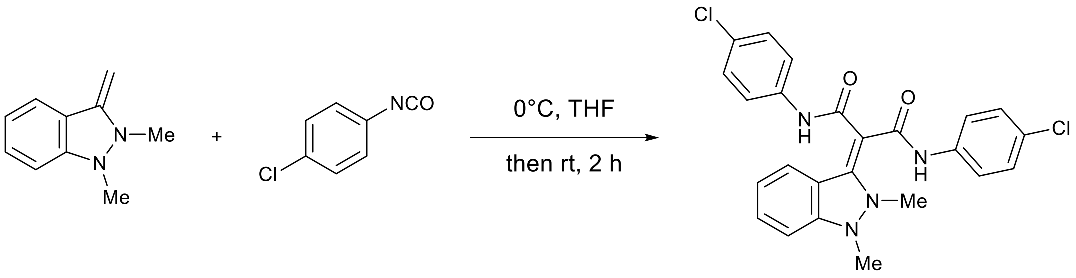

Followed by the general procedure of NHO adduct synthesis, **11b** is prepared from NHO **2a** (80 mg, 1 Eq, 0.499 mmol) and 4-chlorophenyl isocyanate (154 mg, 2 Eq, 1.000 mmol) as light yellow solid (108 mg, 46%).

^{1}H-NMR (DMSO-d$_6$, 600 MHz): 7.84 (d, J = 8.2 Hz, 1H, Ar-H), 7.69 (t, J = 8.2 Hz, 1H, Ar-H), 7.66 (d, J = 8.2 Hz, 1H, Ar-H), 7.48 (d, J = 7.7 Hz, 4H, Ar-H), 7.24 (t, J = 8.2 Hz, 1H, Ar-H), 7.21 (d, J = 7.7 Hz, 1H, Ar-H), 4.18 (s, 3H, N-Me), 3.96 (s, 3H, N-Me) ppm.

^{13}C{^{1}H}-NMR (DMSO-d$_6$, 150 MHz): 166.2 (o, C=O), 147.1 (o, Ar-C), 140.2 (o, Ar-C), 140.0 (o, Ar-C), 131.3 (+, Ar-C), 128.1 (+, Ar-C), 123.9 (o, Ar-C), 123.8 (+, Ar-C), 122.5 (+, Ar-C), 120.8 (o, Ar-C), 120.1 (+, Ar-C), 110.3 (+, Ar-C), 73.4 (o, C=indazole), 35.6 (+, Ar-C), 33.4 (+, Ar-C) ppm.

ESI-MS (m/z): calculated for $[C_{24}H_{20}Cl_2N_4O_2+Na]^+$: 489.0856, found 489.0854.

IR (ATR): $\tilde{\nu}$ = 1501 (amide) cm^{-1}.

Melting point: 198-199 °C, decomposed.

^{1}H-NMR

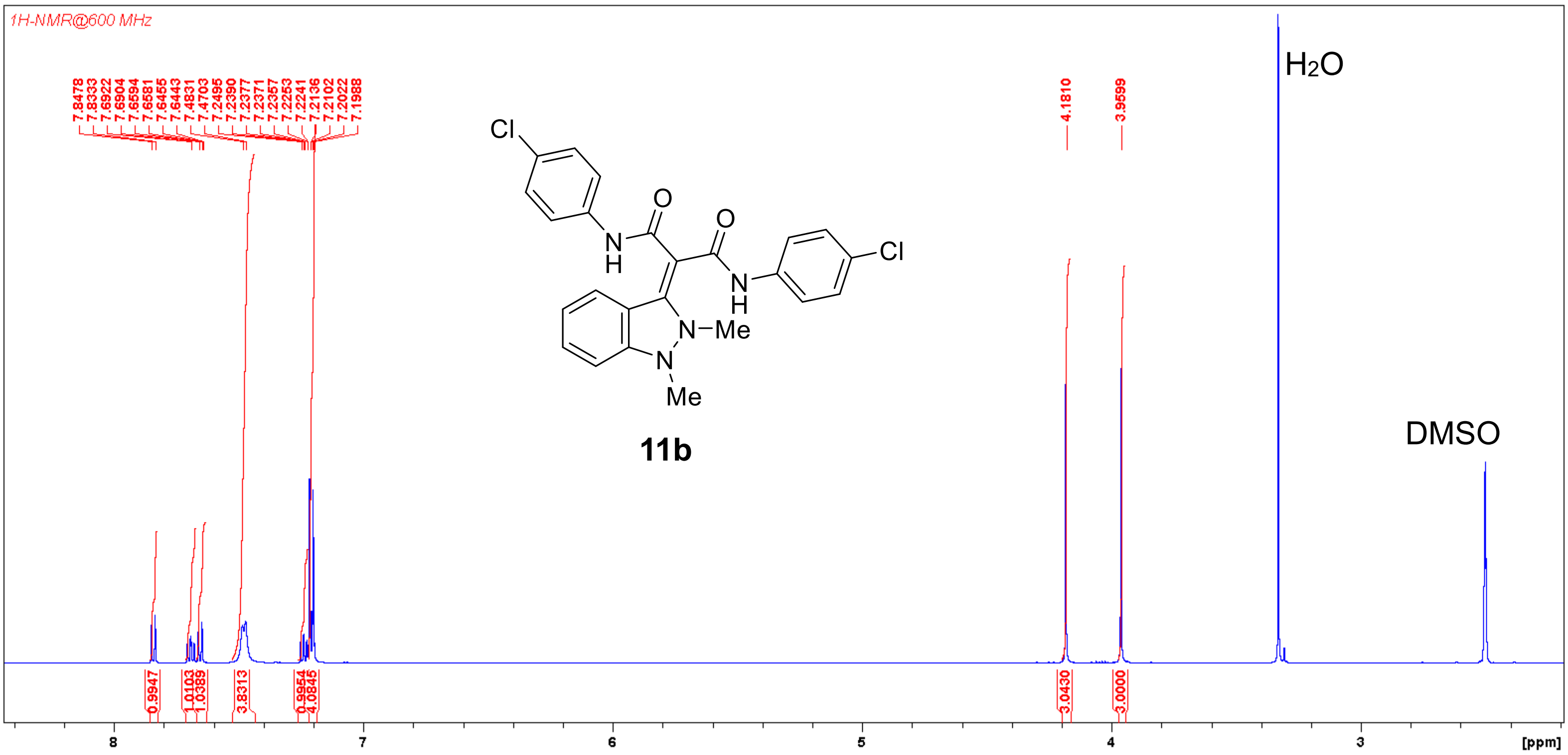
1H-NMR@600 MHz
7.8478 7.8333 7.6922 7.6904 7.6594 7.6581 7.6455 7.6443 7.4831 7.4703 7.2495 7.2390 7.2377 7.2371 7.2357 7.2253 7.2241 7.2136 7.2102 7.2022 7.1988
4.1810
3.9599
H2O
DMSO
Cl
O
N
H
Me
11b
0.9947
1.0103
1.0389
3.8313
0.9954
4.0845
3.0430
3.0000
[ppm]

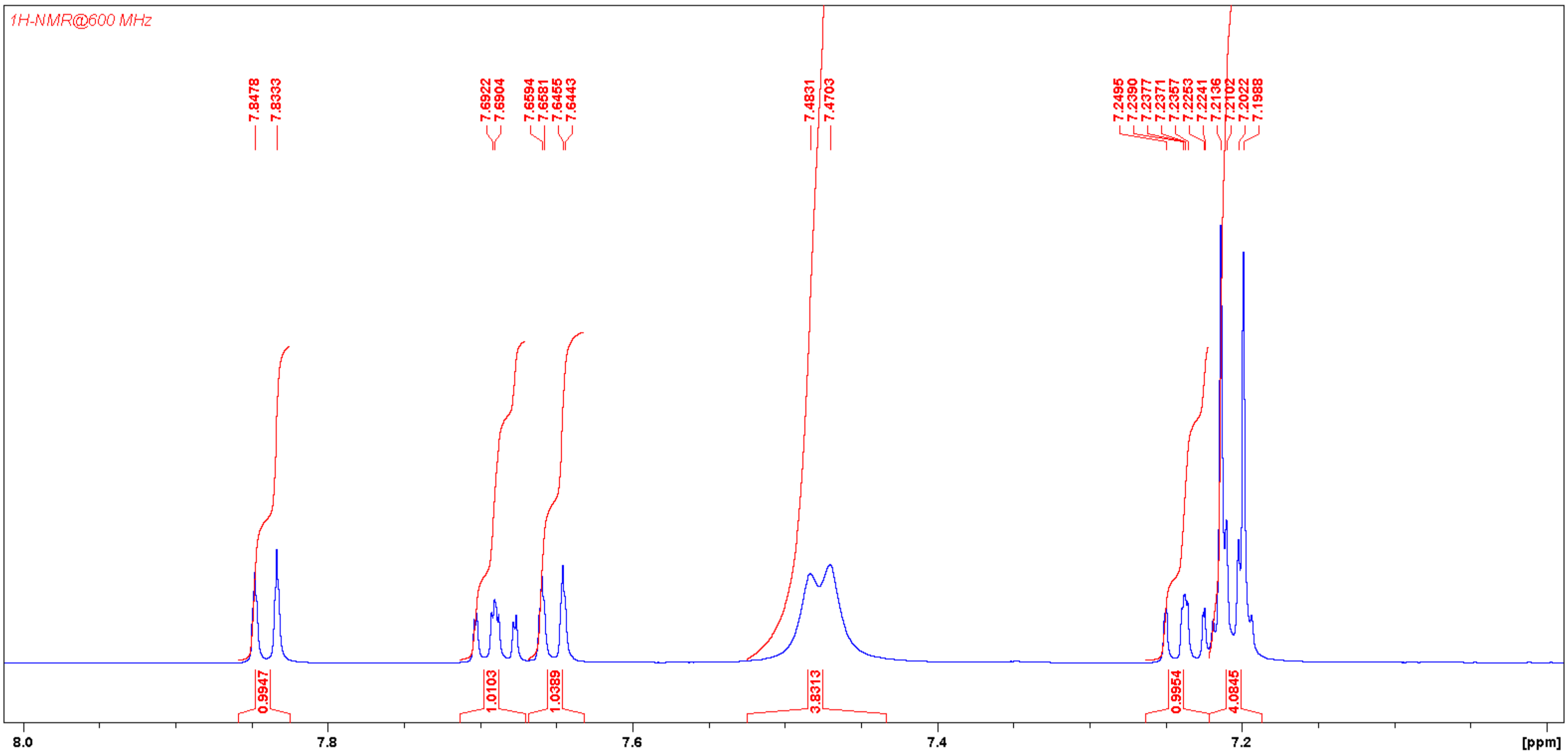
1H-NMR@600 MHz
7.8478 7.8333
7.6922 7.6904
7.6594 7.6581 7.6455 7.6443
7.4831 7.4703
7.2495 7.2390 7.2377 7.2371 7.2357 7.2253 7.2241 7.2136 7.2102 7.2022 7.1988
0.9947
1.0103
1.0389
3.8313
0.9954
4.0845
[ppm]

$^{13}C\{^1H\}$-NMR

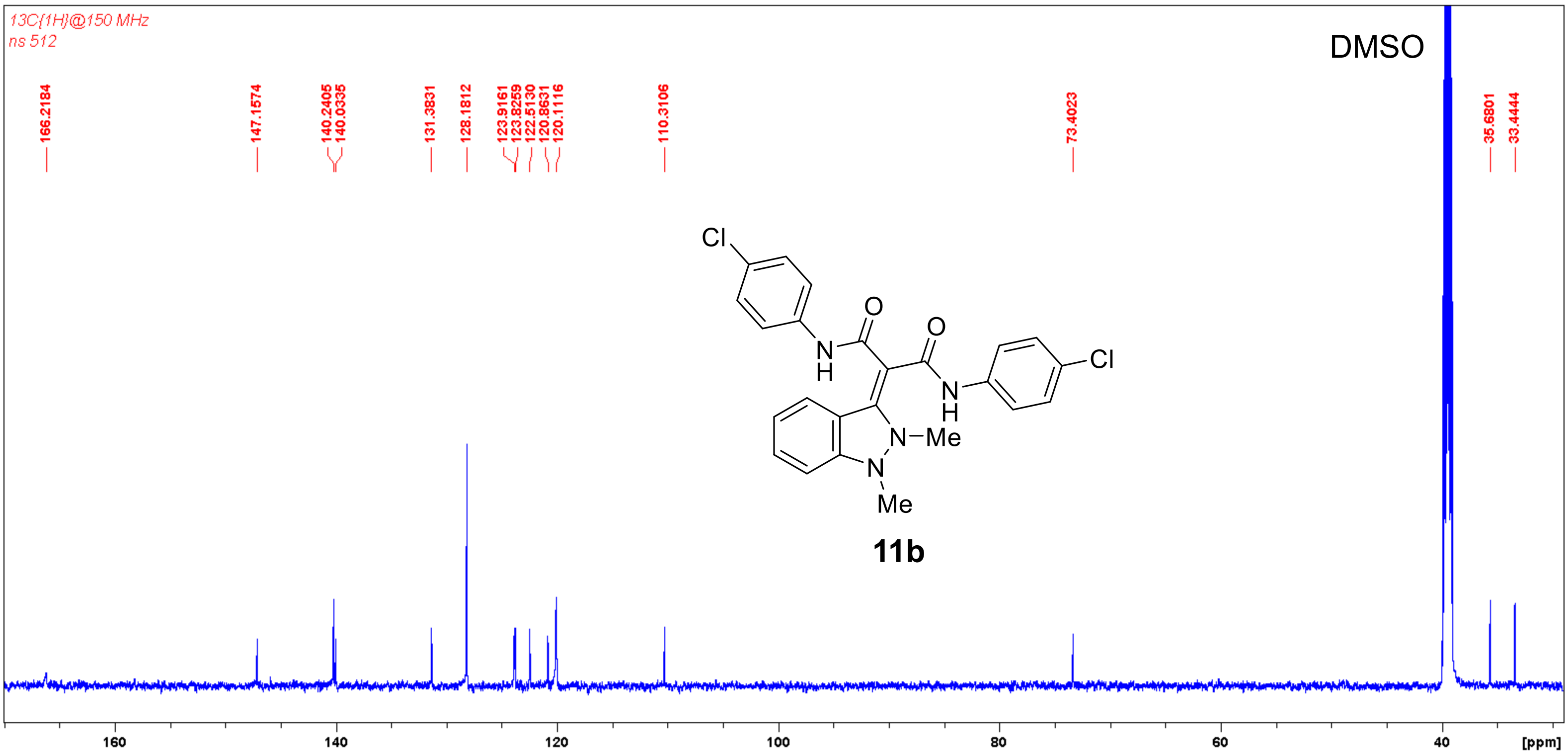

^{13}C-DEPT

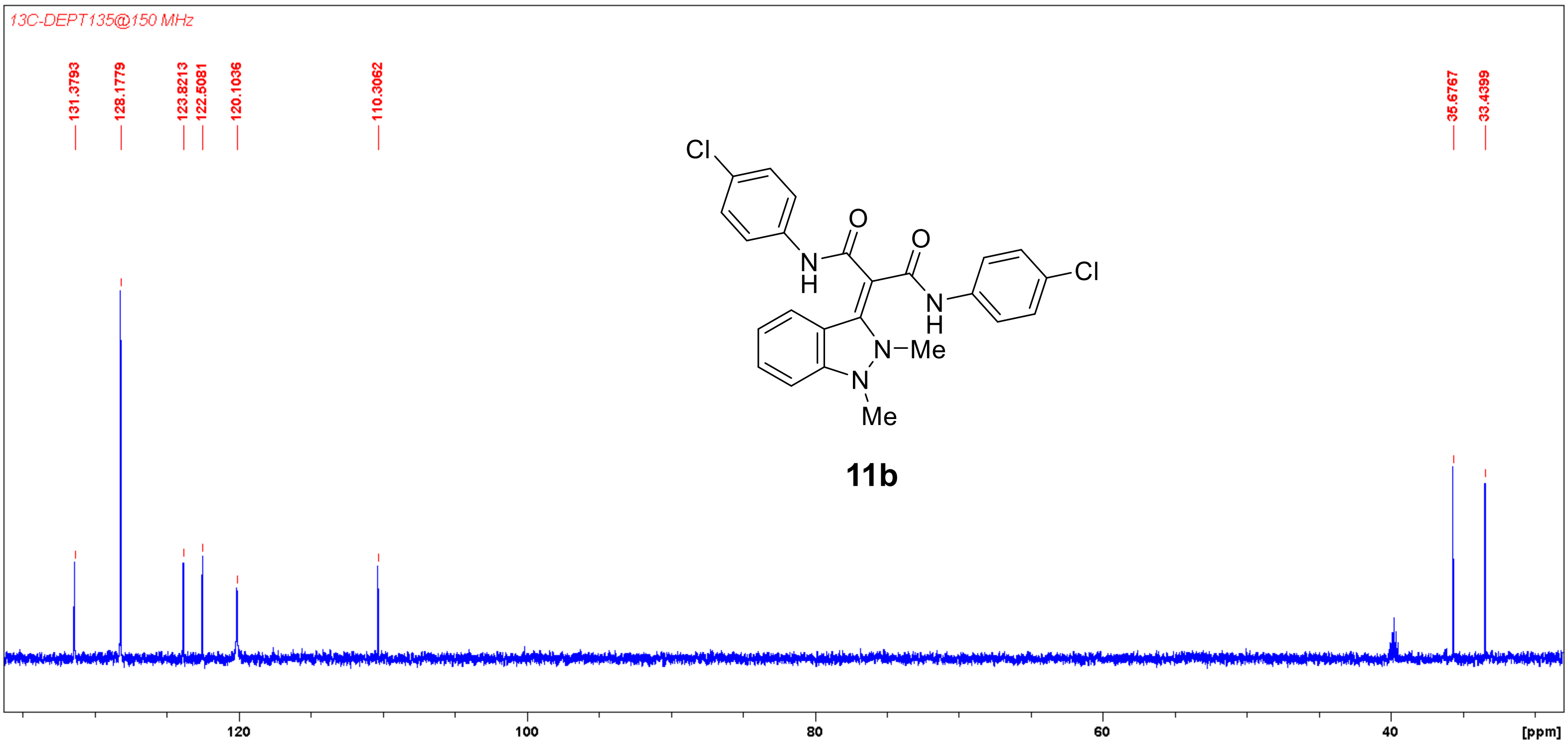

H,H-COSY

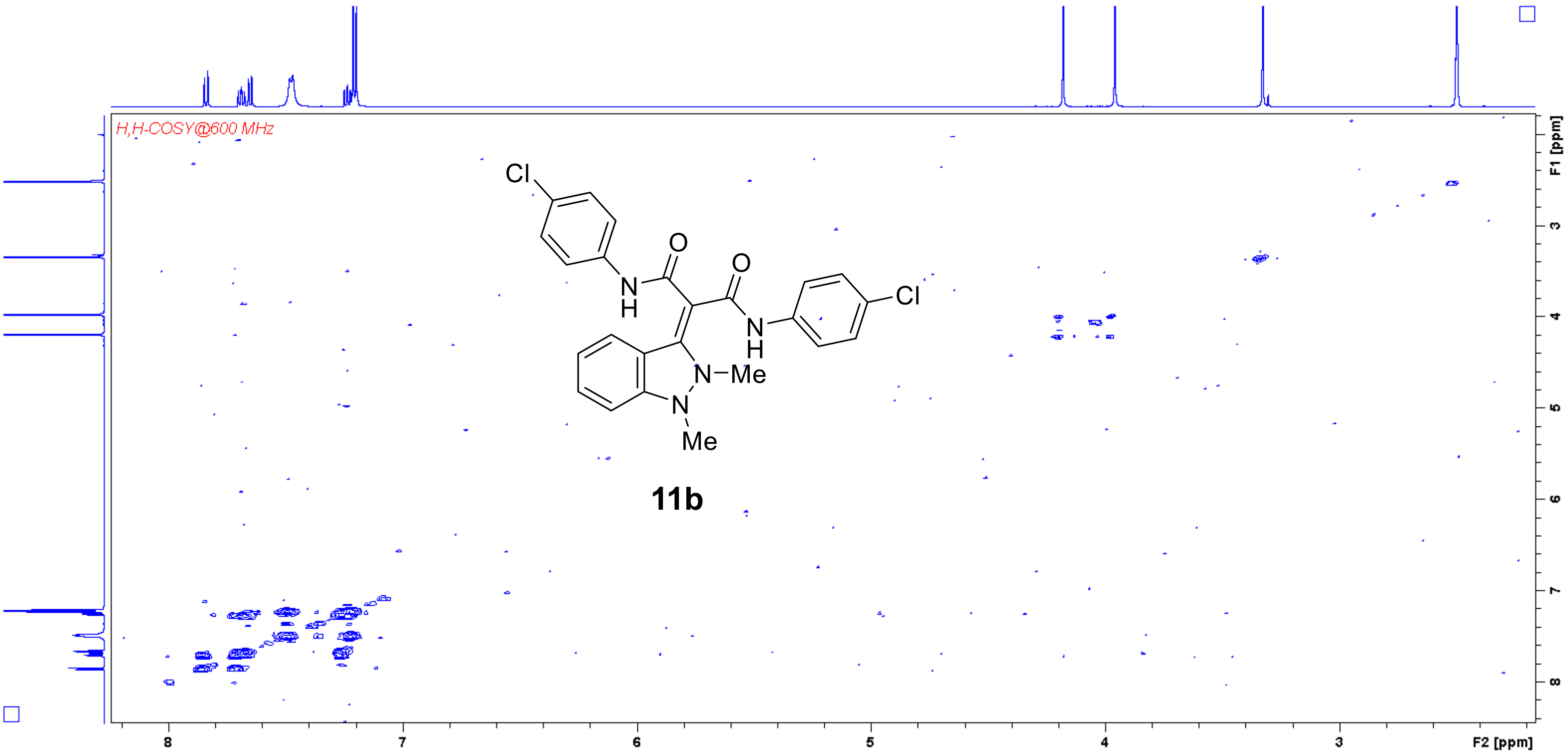
H,H-COSY@600 MHz
Cl
O
O
N
H
N
H
Cl
N−Me
N
Me
11b
F1 [ppm]
F2 [ppm]

HSQC

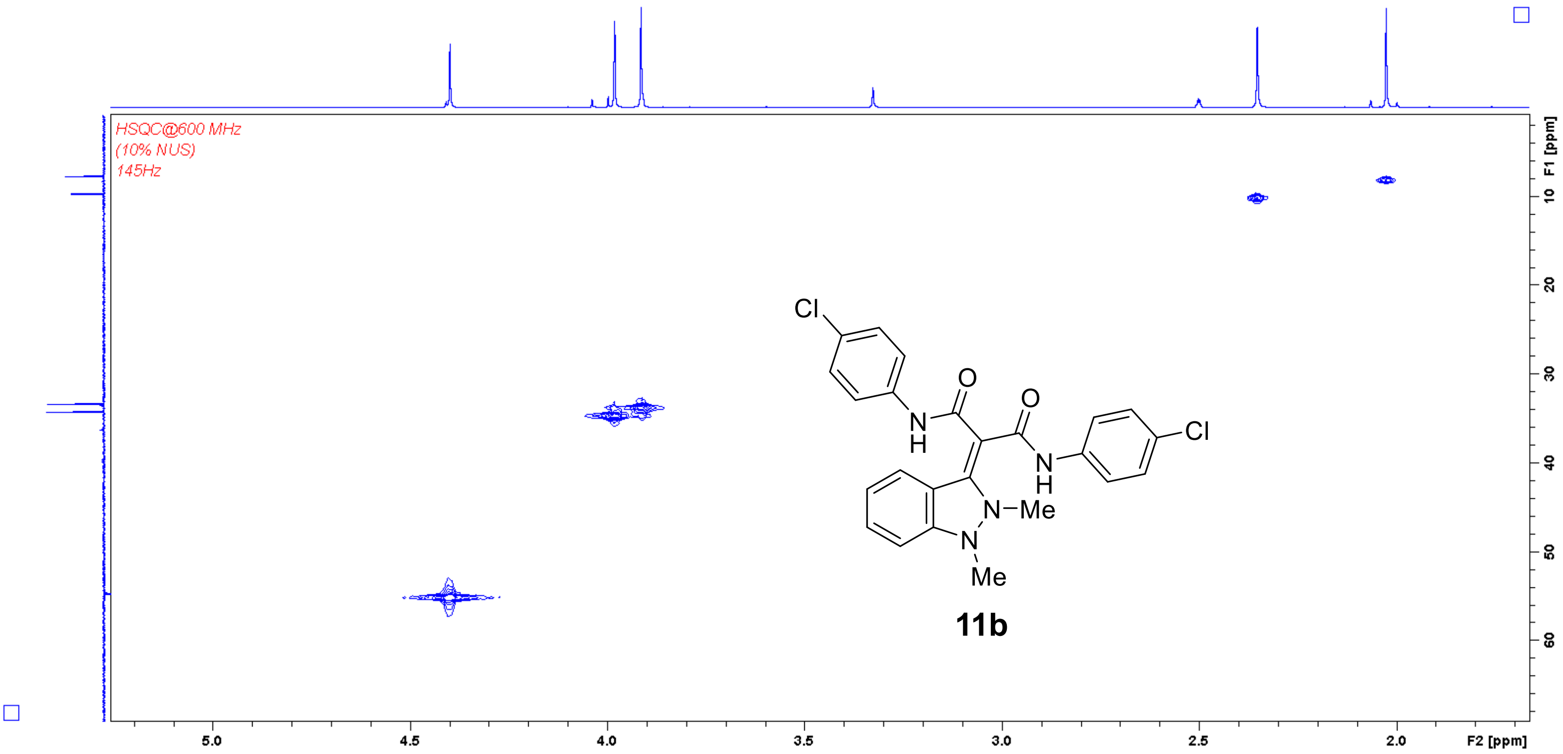
HSQC@600 MHz
(10% NUS)
145Hz
Cl
O
O
N
H
N
H
Cl
N−Me
N
Me
11b
F1 [ppm]
F2 [ppm]

HMBC

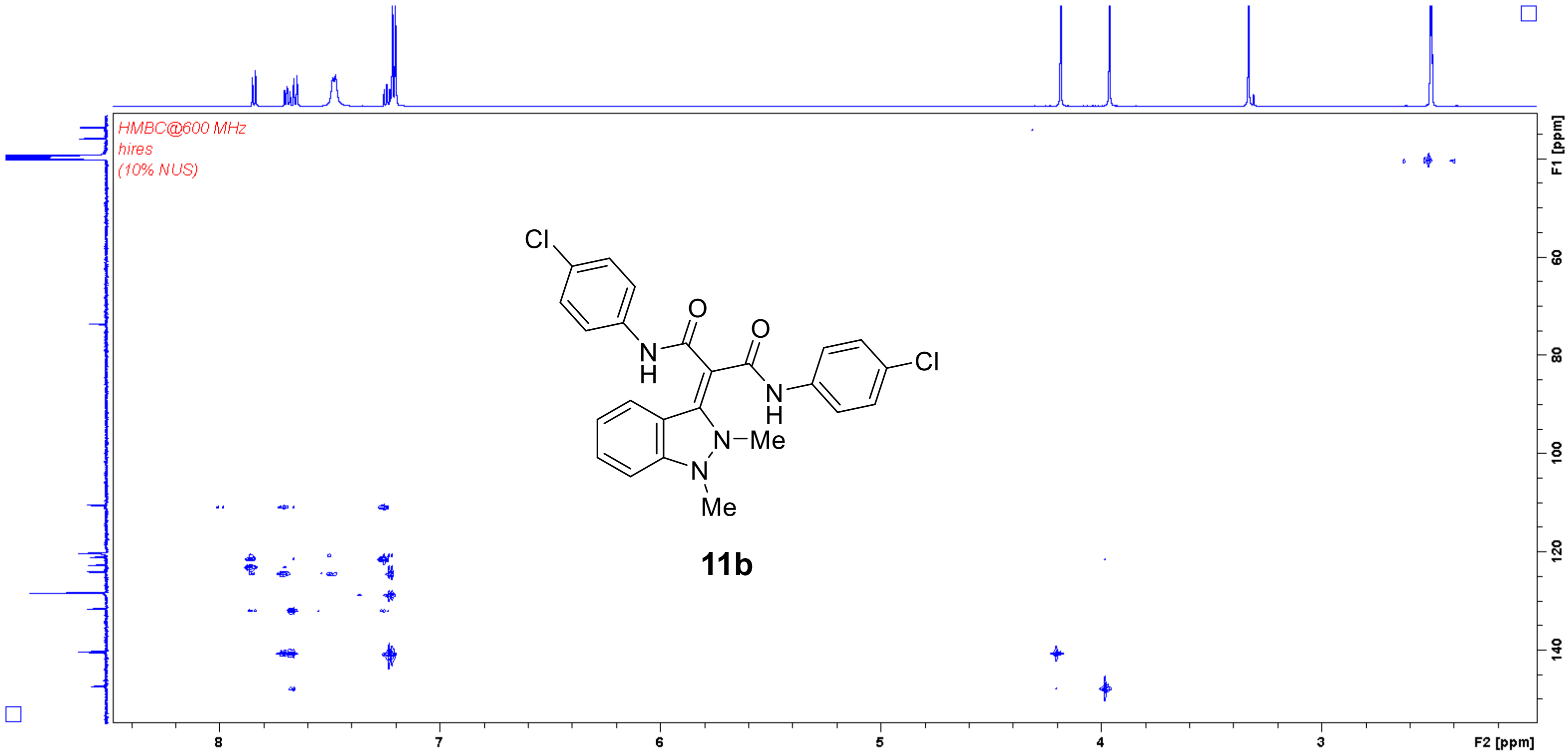
HMBC@600 MHz
hires
(10% NUS)
Cl
O
O
N
H
Cl
N
H
N−Me
N
Me
11b
F1 [ppm]
60
80
100
120
140
8
7
6
5
4
3
F2 [ppm]

Synthesis of N[1],N[3]-bis(2-methoxylphenyl)-2-(1,2-dimethyl-2,3-dihydro-1*H*-indazol-3-ylidene)malonamide **11c**

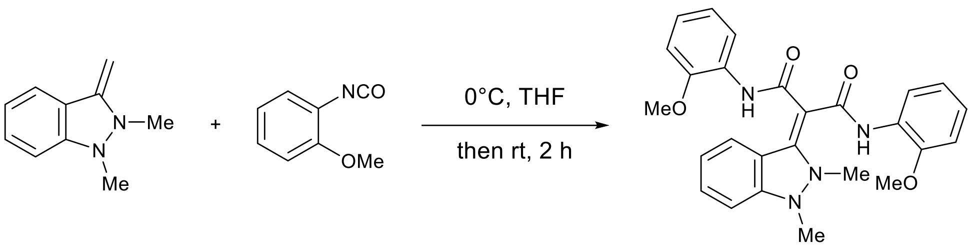

Followed by the general procedure of NHO adduct synthesis, **11c** is prepared from NHO **2a** (80 mg, 1 Eq, 0.499 mmol) and 2-methoxylphenyl isocyanate (149 mg, 2 Eq, 0.998 mmol) as light yellow solid (187 mg, 82%).

^{1}H-NMR (DMSO-d$_6$, 600 MHz): 8.26 (s, 2H, Ar-H), 7.88 (d, J = 8.2 Hz, 1H, Ar-H), 7.79 (d, J = 8.0 Hz, 1H, Ar-H), 7.75 (t, J = 8.2 Hz, 1H, 6-H), 7.31 (t, J = 8.0 Hz, 1H, Ar-H), 6.90-6.87 (m, 2H, Ar-H), 6.85-6.81 (m, 4H, Ar-H), 4.20 (s, 3H, N-Me), 3.99 (s, 3H, N-Me), 3.60 (s, 6H, OMe) ppm.

^{13}C{^{1}H}-NMR (DMSO-d$_6$, 150 MHz): 165.8 (o, C=O), 148.0 (o, Ar-C), 147.1 (o, Ar-C), 139.7 (o, Ar-C), 131.6 (+, Ar-C), 130.4 (o, Ar-C), 124.1 (+, Ar-C), 122.4 (+, Ar-C), 120.5 (+, Ar-C), 120.3 (+, Ar-C), 119.9 (o, Ar-C), 119.2 (+, Ar-C), 110.4 (+, Ar-C), 73.5 (o, C=indazole), 55.4 (+, OMe), 35.7 (+, N-Me), 33.4 (+, N-Me) ppm.

IR (ATR): $\tilde{\nu}$ = 1501 (amide), 505 (C-O-C deformation) cm^{-1}.

ESI-MS (m/z): calculated for $[C_{26}H_{26}N_4O_4+Na]^+$: 481.1846, found 481.1841.

Melting point: 138-140 °C.

^{1}H-NMR

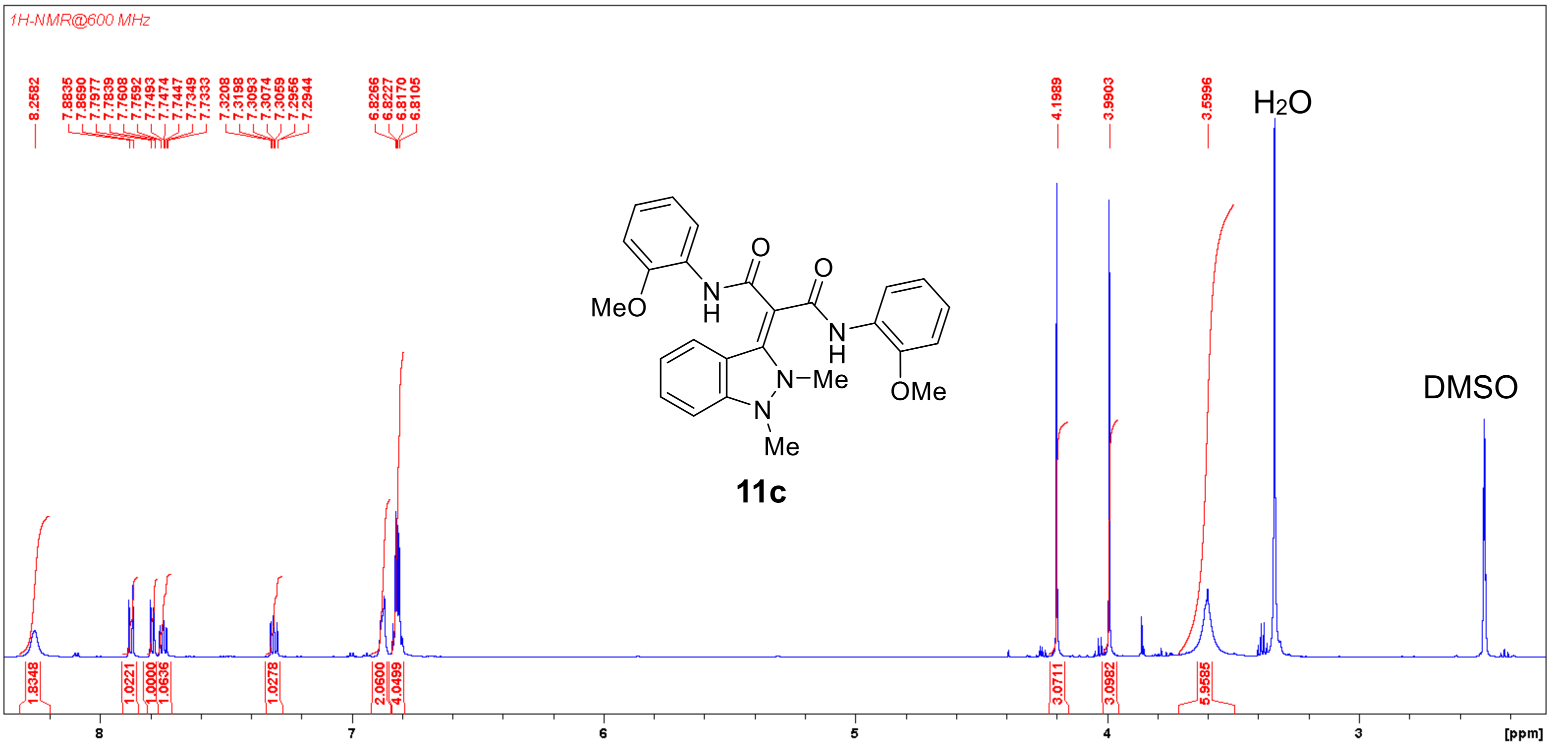

^{13}C{^{1}H}-NMR

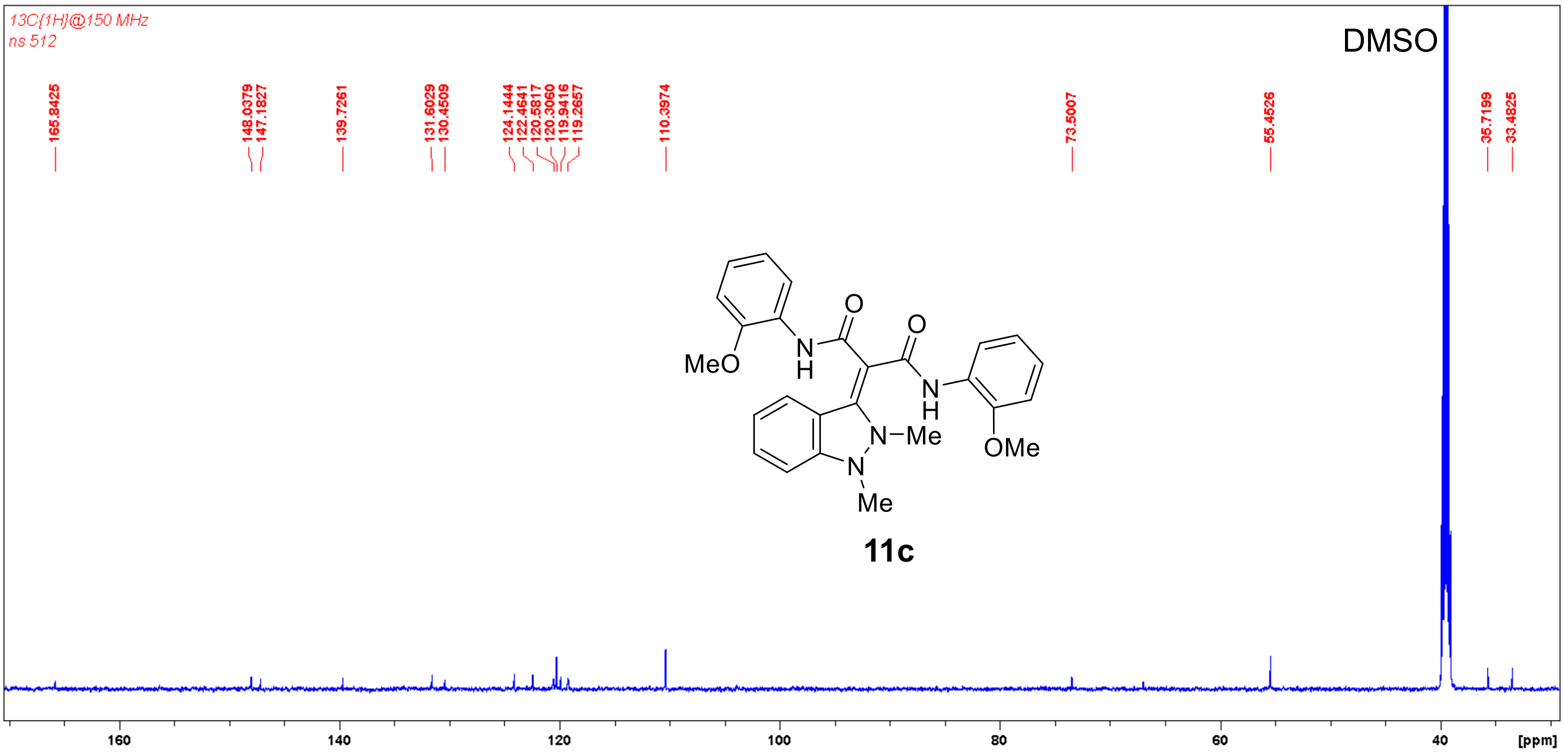

^{13}C-DEPT

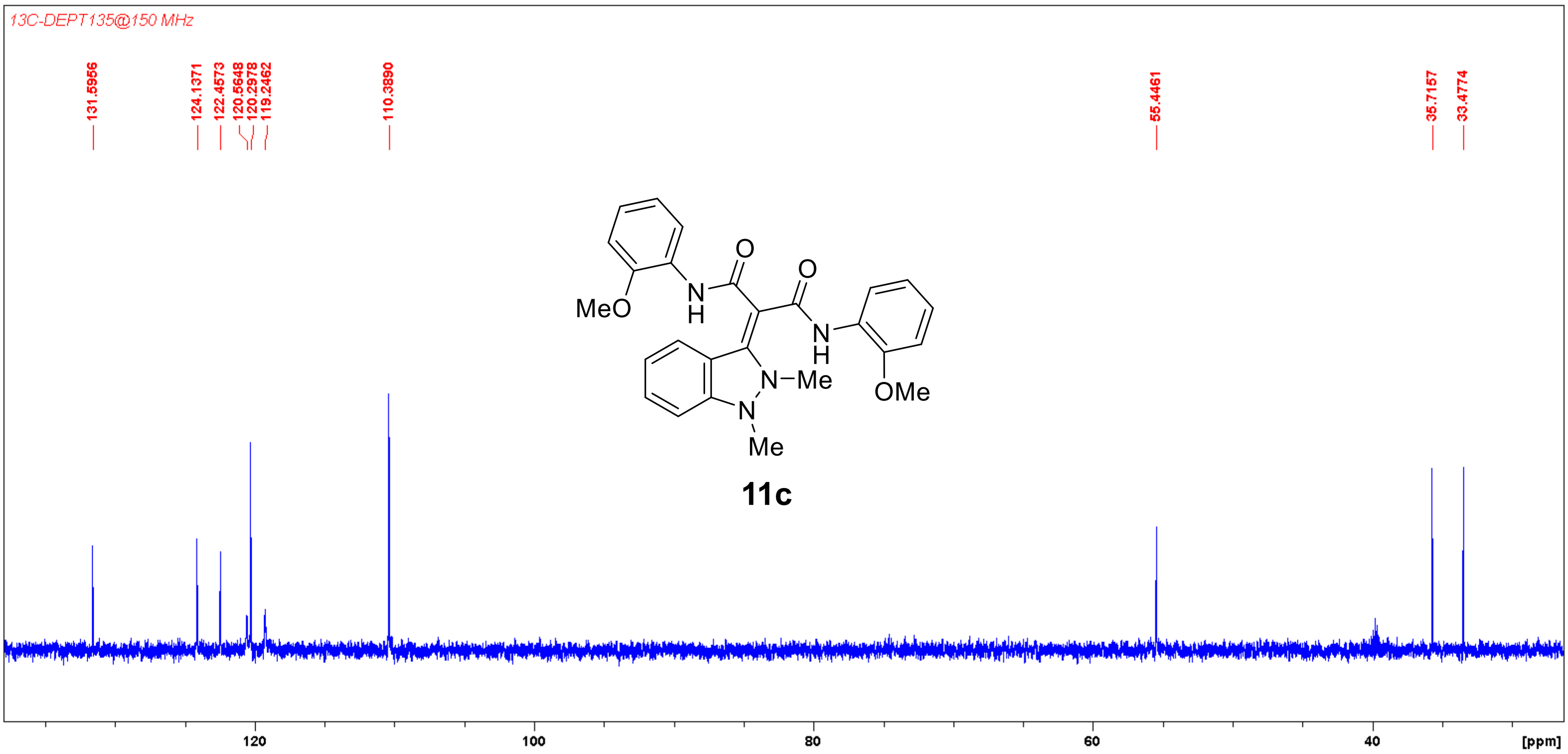

H,H-COSY

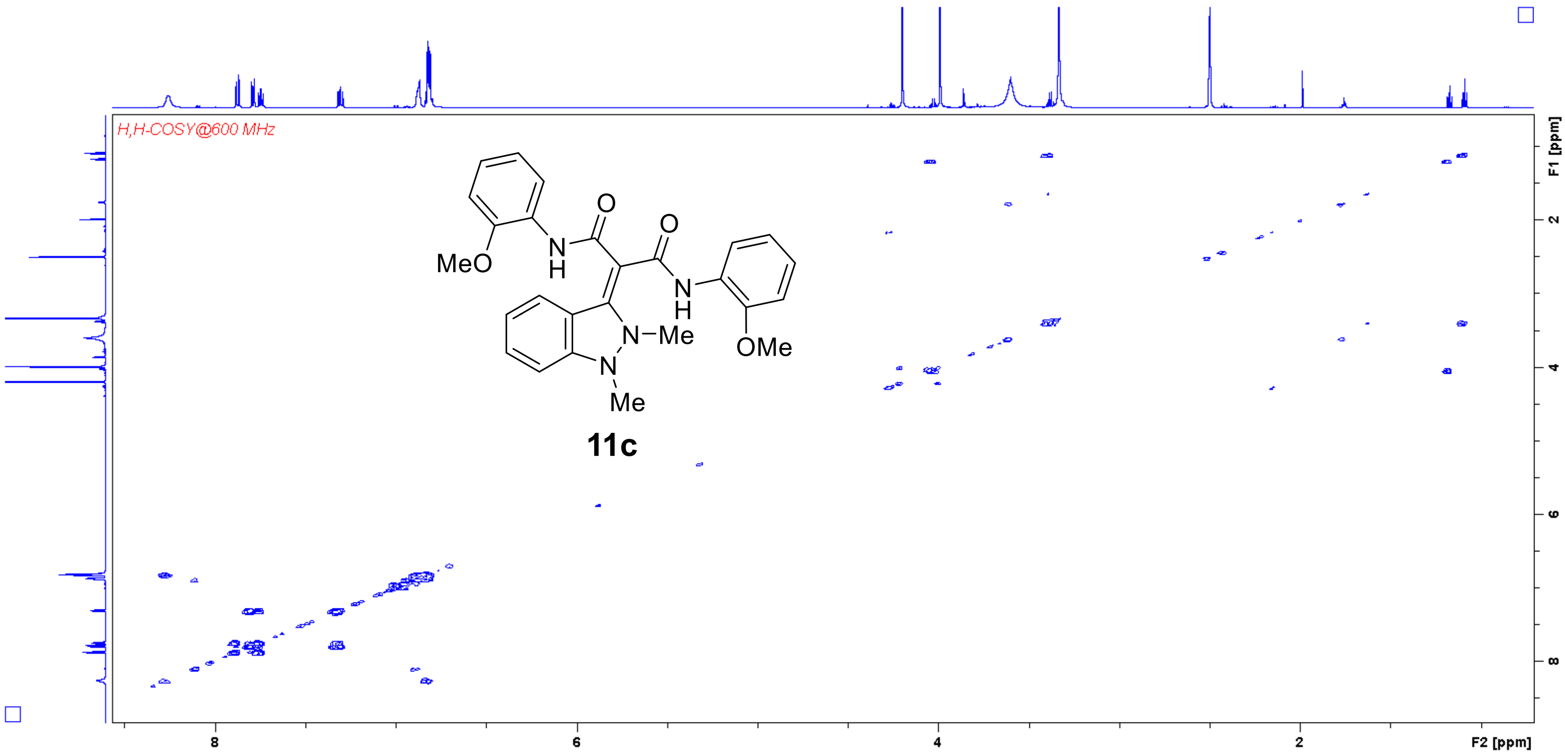

HSQC

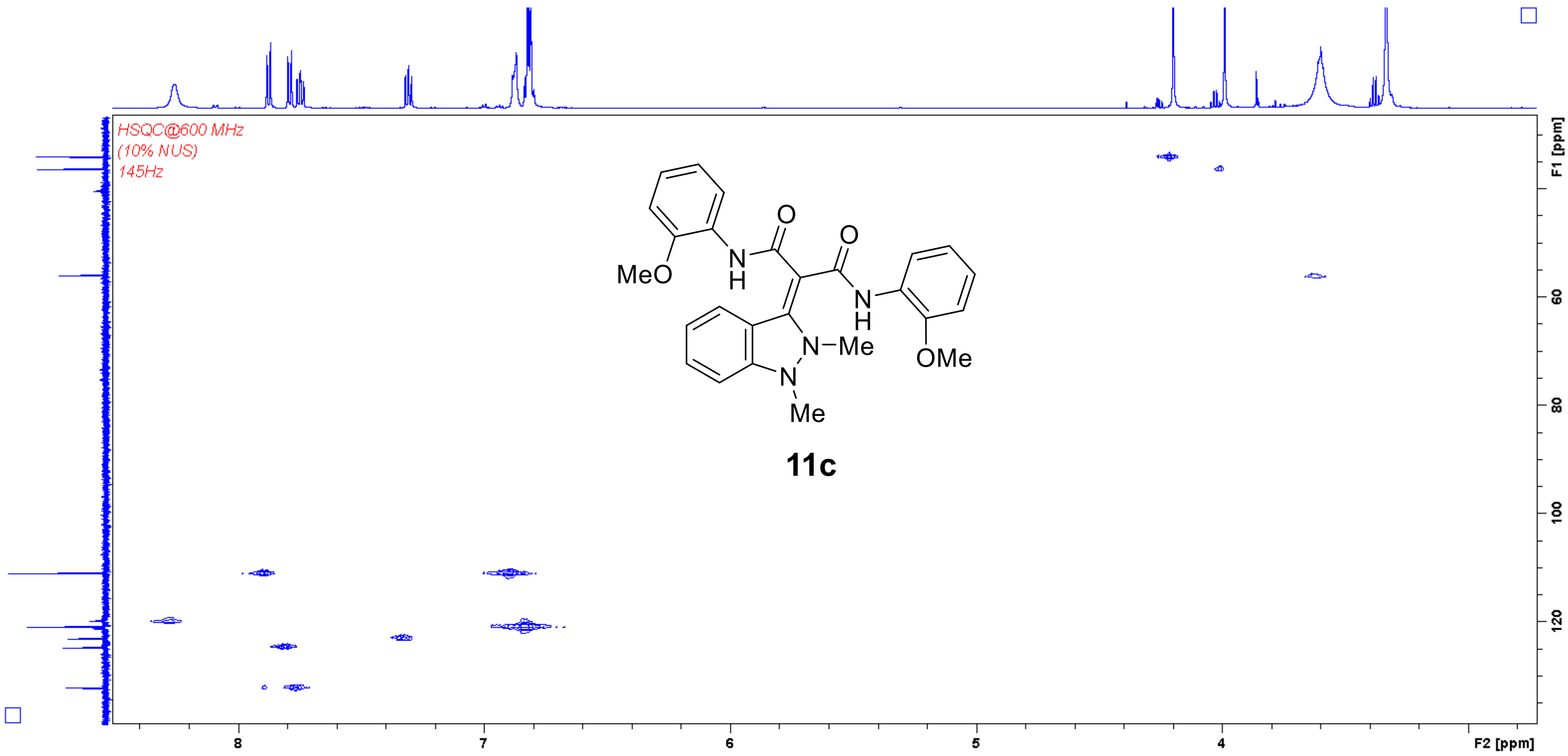

HSQC@600 MHz
(10% NUS)
145Hz
MeO
N
H
O
O
N
H
N−Me
N
Me
OMe
11c
F1 [ppm]
60
80
100
120
8
7
6
5
4
F2 [ppm]

HMBC

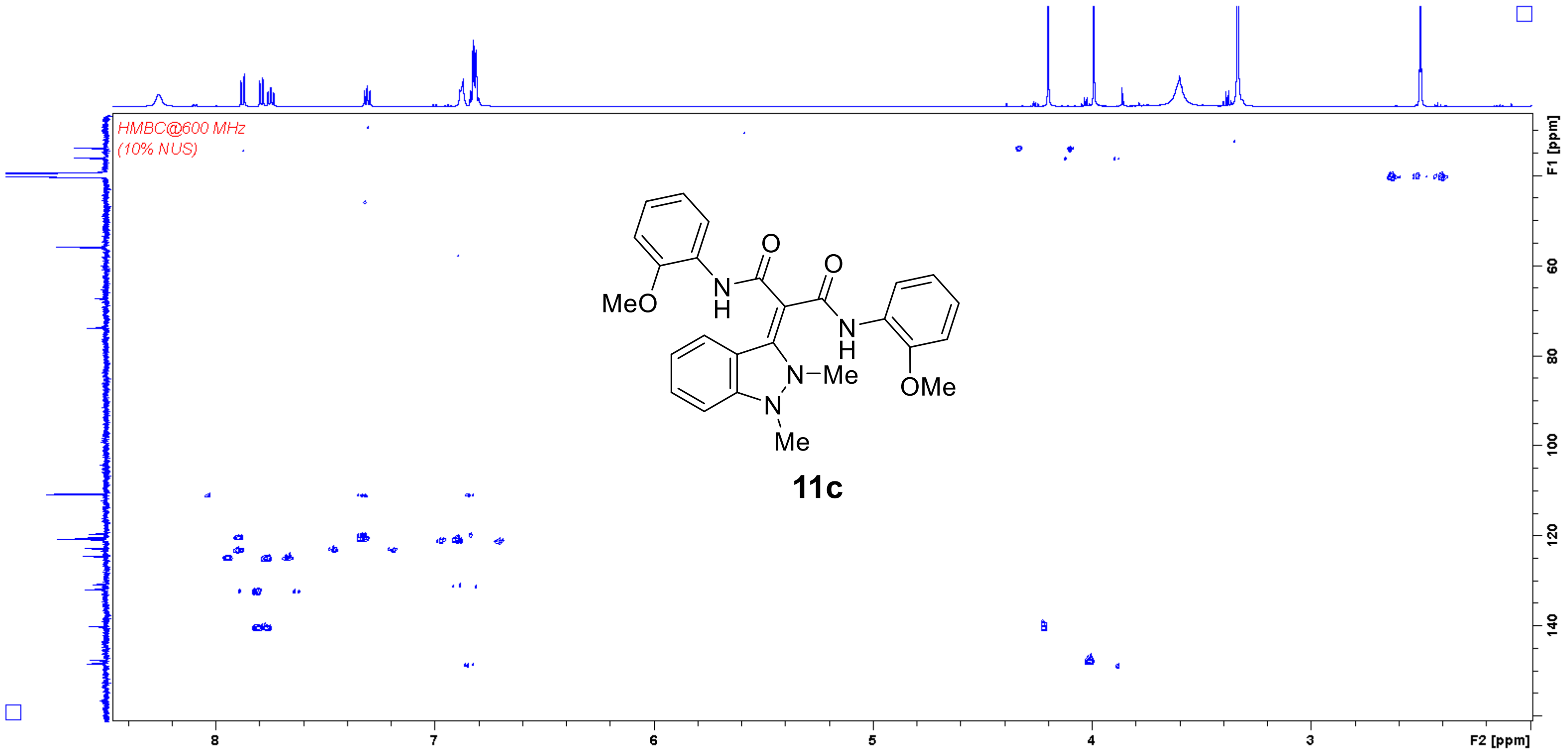

HMBC@600 MHz
(10% NUS)
MeO
N
H
O
O
N
H
N−Me
N
Me
OMe
11c
F1 [ppm]
60
80
100
120
140
8
7
6
5
4
3
F2 [ppm]

Synthesis of N^1,N^3-bis(4-methylphenyl)-2-(1,2-dimethyl-2,3-dihydro-1*H*-indazol-3-ylidene)malonamide **11d**

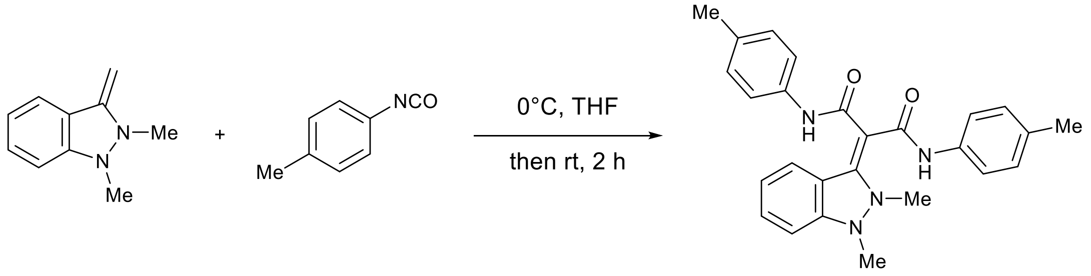

Followed by the general procedure of NHO adduct synthesis, **11d** is prepared from NHO **2a** (80 mg, 1 Eq, 0.499 mmol) and 4-methylphenyl isocyanate (133 mg, 2 Eq, 0.998 mmol) as light yellow solid (160 mg, 75%).

^{1}H-NMR (DMSO-d$_6$, 600 MHz): 7.80 (d, J = 8.9 Hz, 1H, Ar-H), 7.68-7.65 (m, 2H, Ar-H), 7.32 (d, J = 8.2 Hz, 4H, Ar-H), 7.21 (t, J = 7.5 Hz, 1H, Ar-H), 6.97 (d, J = 8.2 Hz, 4H, Ar-H), 4.13 (s, 3H, N-Me), 3.93 (s, 3H, N-Me), 2.20 (s, 6H, Me) ppm.

^{13}C{^{1}H}-NMR (DMSO-d$_6$, 150 MHz): 166.3 (o, Ar-C), 148.1 (o, Ar-C), 140.2 (o, Ar-C), 138.8 (o, Ar-C), 131.1 (+, Ar-C), 129.1 (o, Ar-C), 128.7 (+, Ar-C), 124.0 (+, Ar-C), 122.2 (+, Ar-C), 121.0 (o, Ar-C), 118.7 (+, Ar-C), 110.2 (+, Ar-C), 73.5 (o, C=indazole), 35.8 (+, N-Me), 33.5 (+, N-Me), 20.3 (+, Me) ppm.

ESI-MS (m/z): calculated for $[C_{26}H_{26}N_4O_2+Na]^+$: 449.1948 , found 449.1950.

IR (ATR): $\tilde{\nu}$ = 1508 (amide) cm^{-1}.

Melting point: 201-203 °C, decomposed.

^{1}H-NMR

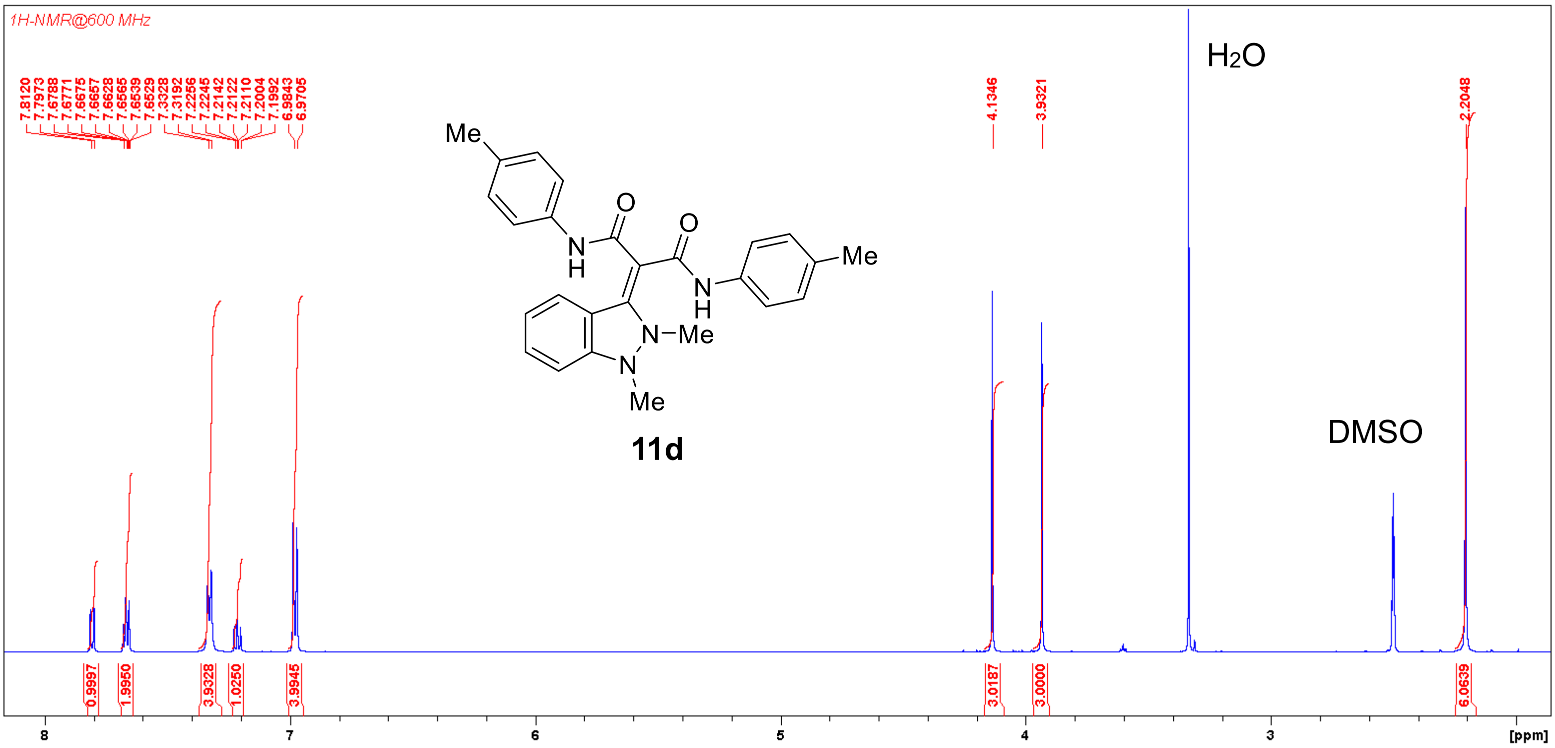
1H-NMR@600 MHz
7.8120
7.7973
7.6788
7.6771
7.6675
7.6657
7.6628
7.6565
7.6539
7.6529
7.3328
7.3192
7.2256
7.2245
7.2142
7.2122
7.2110
7.2004
7.1992
6.9843
6.9705
4.1346
3.9321
2.2048
Me
O
O
N
H
N
H
Me
N−Me
N
Me
11d
H2O
DMSO
0.9997
1.9950
3.9328
1.0250
3.9945
3.0187
3.0000
6.0639
[ppm]

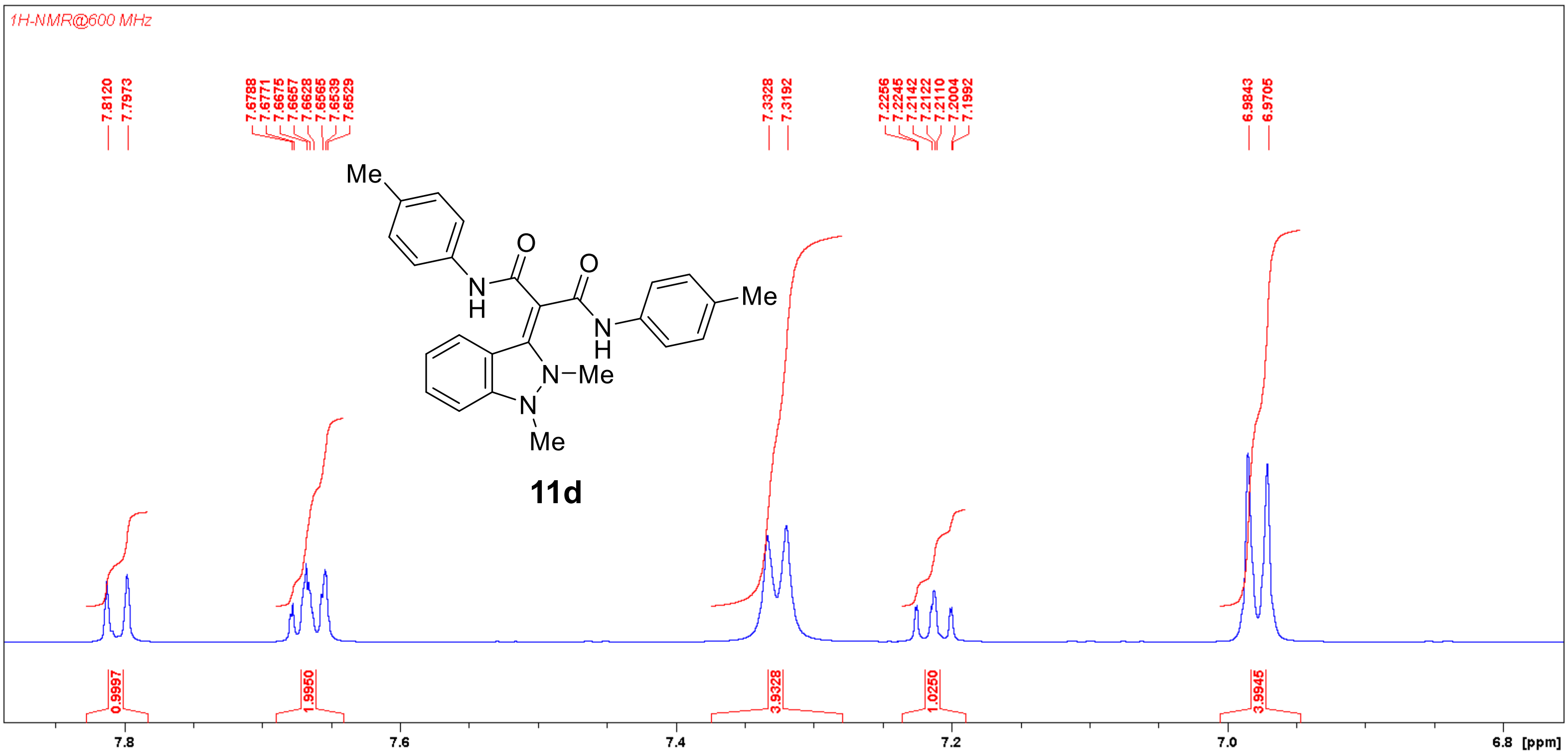
1H-NMR@600 MHz
7.8120
7.7973
7.6788
7.6771
7.6675
7.6657
7.6628
7.6565
7.6539
7.6529
7.3328
7.3192
7.2256
7.2245
7.2142
7.2122
7.2110
7.2004
7.1992
6.9843
6.9705
Me
O
O
N
H
N
H
Me
N−Me
N
Me
11d
0.9997
1.9950
3.9328
1.0250
3.9945
[ppm]

$^{13}C\{^1H\}$-NMR

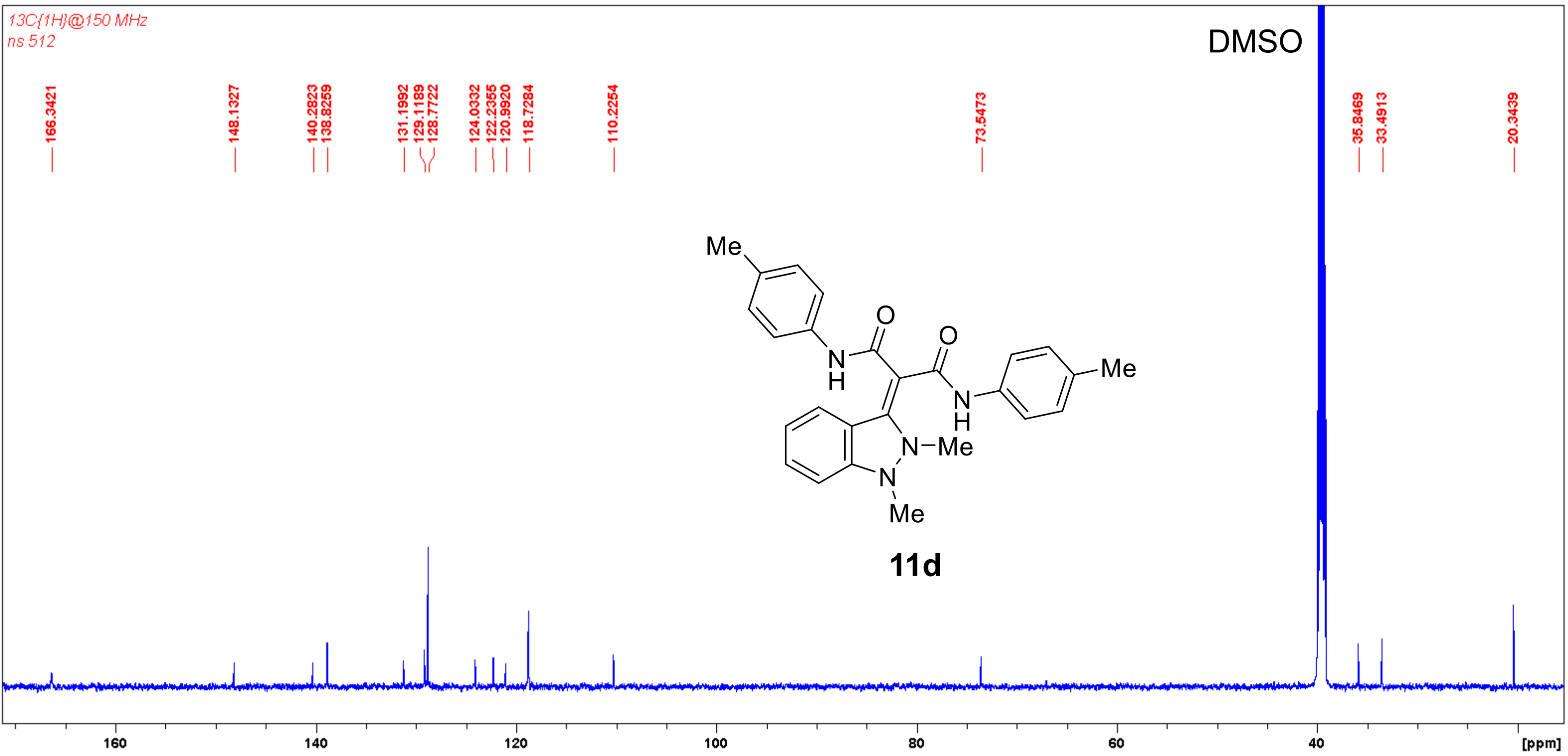

^{13}C-DEPT

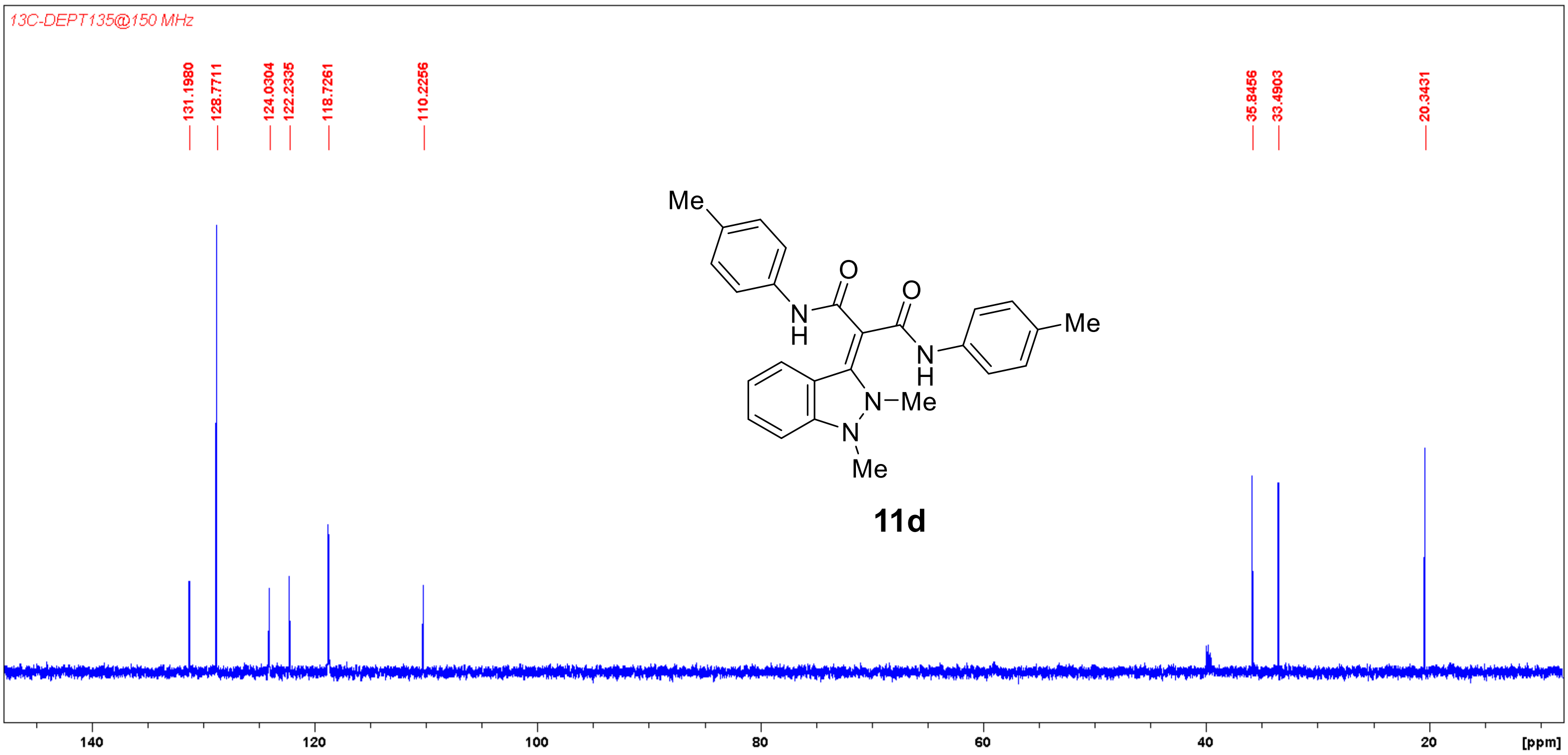

H,H-COSY

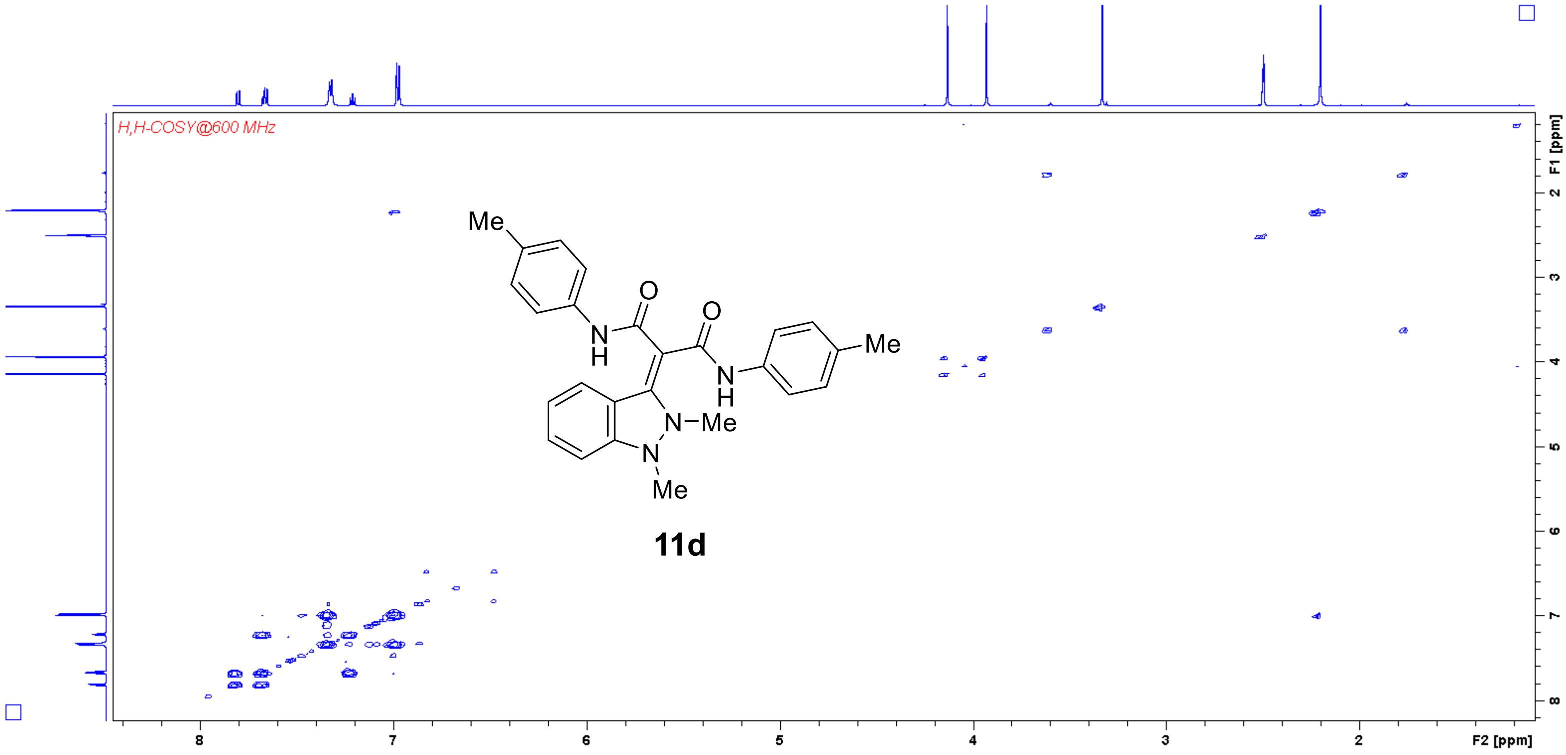
H,H-COSY@600 MHz
Me
O
O
N
H
N
H
Me
N−Me
N
Me
11d
F1 [ppm]
F2 [ppm]
8
7
6
5
4
3
2

HSQC

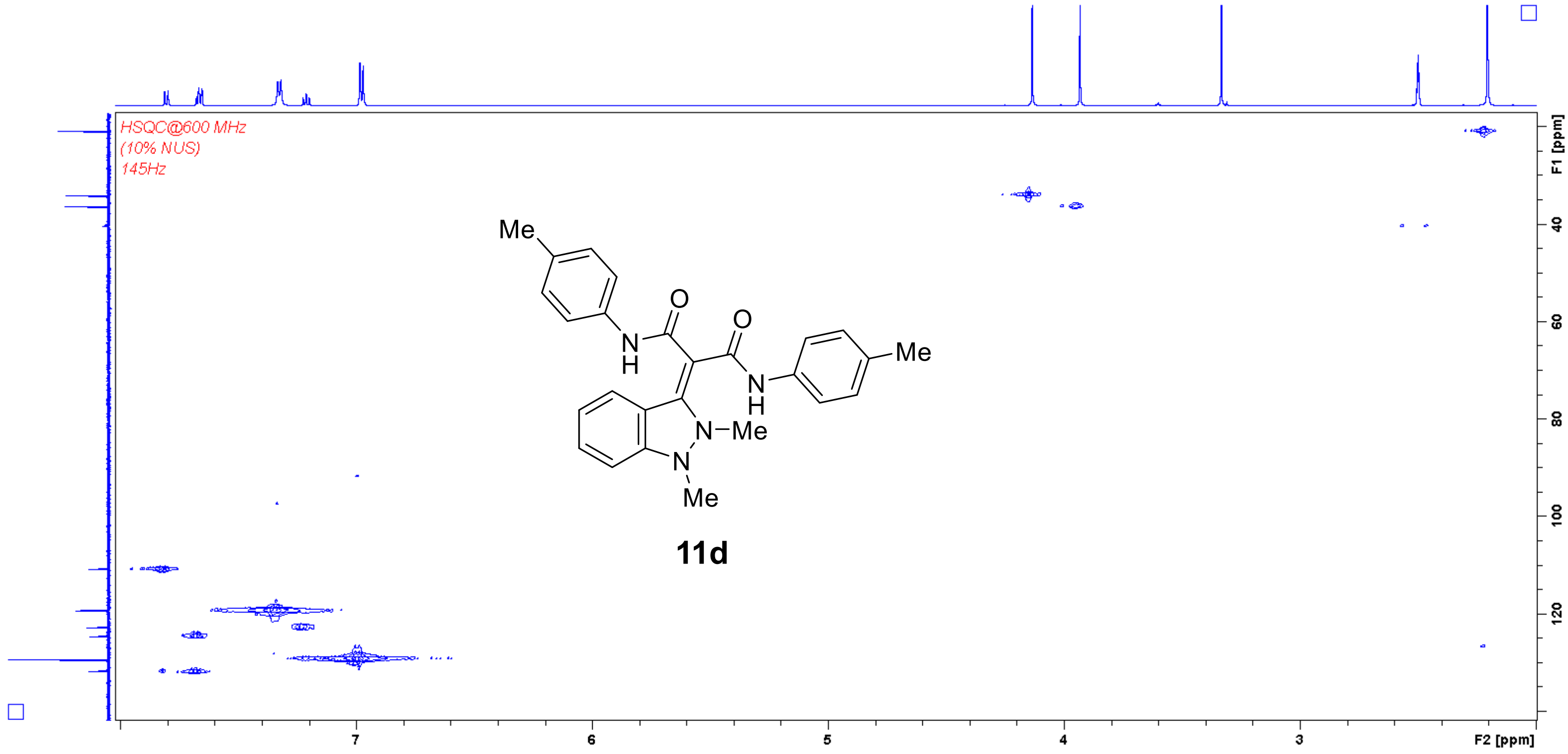
HSQC@600 MHz
(10% NUS)
145Hz
Me
O
O
N
H
N
H
Me
N−Me
N
Me
11d
F1 [ppm]
F2 [ppm]
7
6
5
4
3
40
60
80
100
120

HMBC

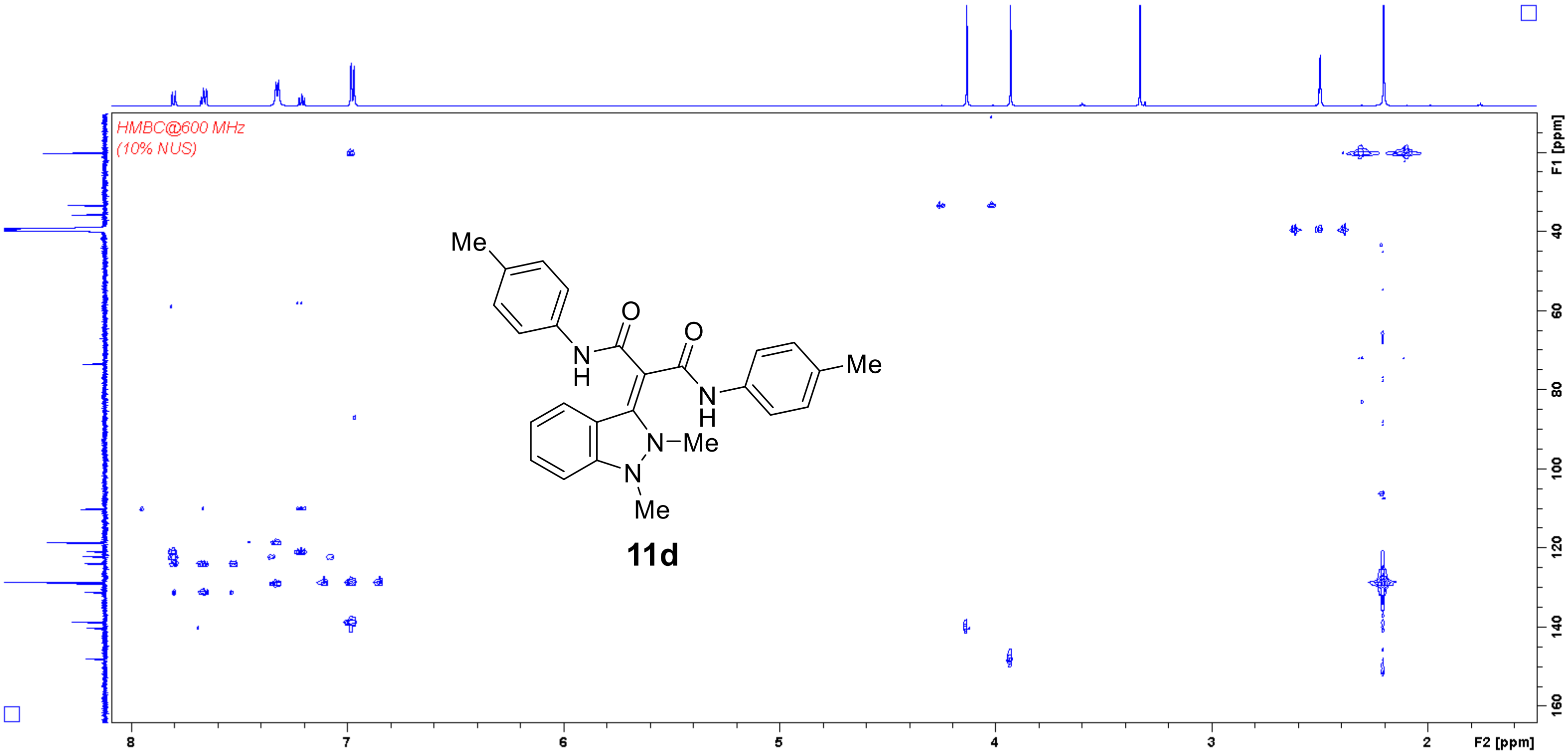

11d

Synthesis of N[1],N[3]-bis(4-chlorophenyl)-2-(2,4-dimethyl-1,5-diphenyl-1,2-dihydro-3*H*-pyrazol-3-ylidene)malonamide **11e**

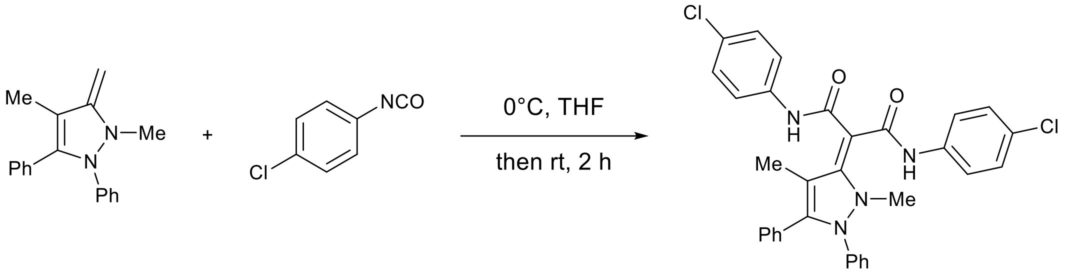

Followed by the general procedure of NHO adduct synthesis, **11e** is prepared from NHO **4b** (100 mg, 1 Eq, 0.403 mmol) and 4-methylphenyl isocyanate (124 mg, 2 Eq, 0.805 mmol) as light yellow solid (107 mg, 48%).

^{1}H-NMR (DMSO-d$_6$, 600 MHz): 12.17 (s, 1H, N-H), 8.06 (s, 1H, Ar-H), 7.65-7.56 (m, 8H, Ar-H), 7.41-7.39 (m, 3H, Ar-H), 7.32-7.29 (m, 2H, Ar-H), 7.25 (s, 4H, Ar-H), 3.51 (s, 3H, N-Me), 1.89 (s, 3H, Me) ppm.

^{13}C{^{1}H}-NMR (DMSO-d$_6$, 150 MHz): 151.1 (o, Ar-C), 144.6 (o, Ar-C), 140.5 (o, Ar-C), 133.0 (o, Ar-C), 131.1 (+, Ar-C), 130.1 (+, Ar-C), 129.8 (+, Ar-C), 129.7 (+, Ar-C), 128.9 (+, Ar-C), 128.7 (+, Ar-C), 128.2 (+, Ar-C), 126.8 (o, Ar-C), 116.0 (o, Ar-C), 72.5 (o, Ar-C), 36.2 (+, N-Me), 9.7 (+, Me) ppm.

ESI-MS (m/z): calculated for $[C_{32}H_{26}N_4O_2Cl+Na]^+$: 591.1330, found 591.1305.

IR (ATR): $\tilde{\nu}$ = 1485 (amide) cm^{-1}.

Melting point: 203-204 °C, decomposed.

^{1}H-NMR

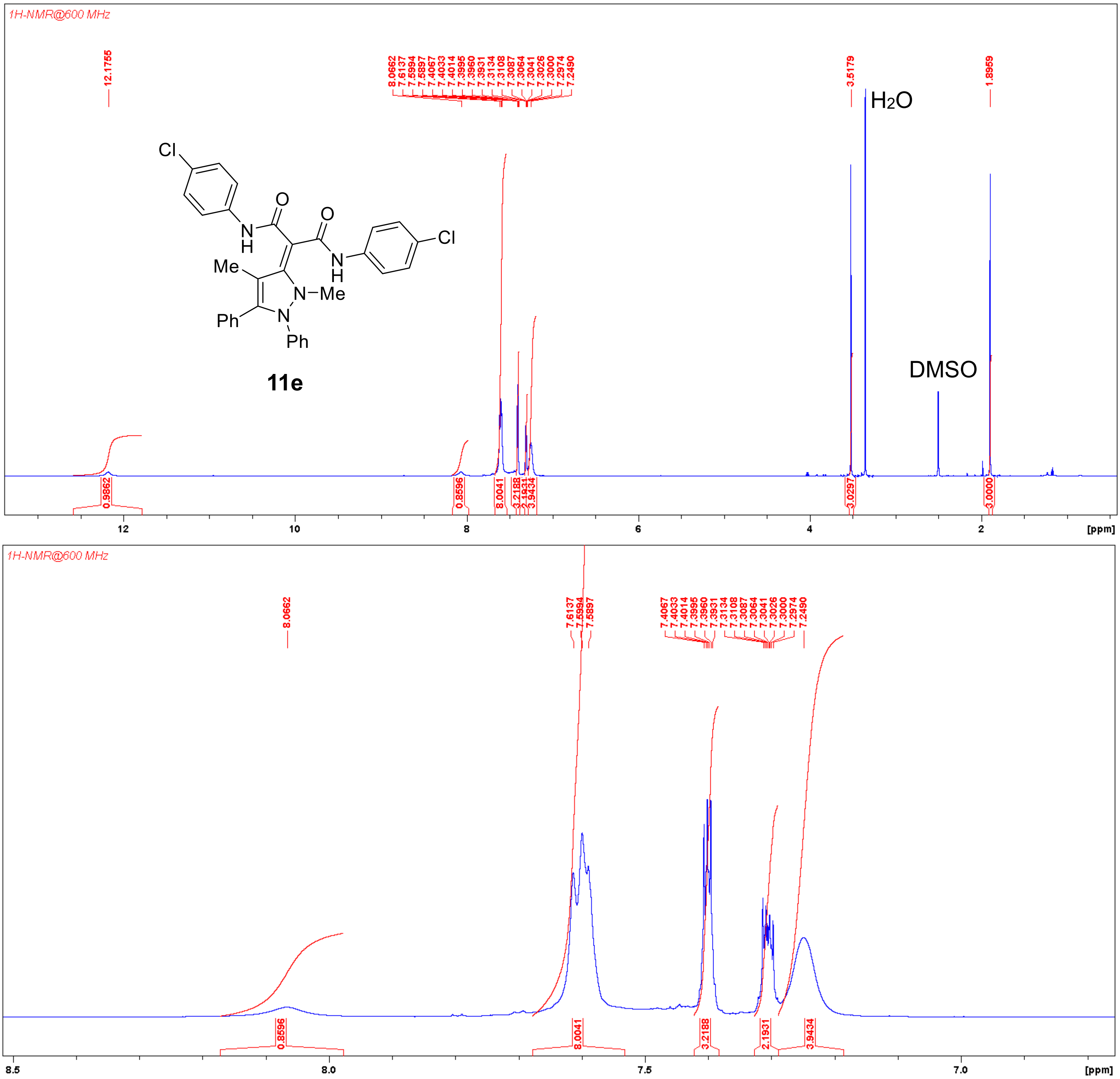
1H-NMR@600 MHz
11e
12.1755
8.0662
7.6137
7.5994
7.5897
7.4067
7.4033
7.4014
7.3995
7.3960
7.3931
7.3134
7.3108
7.3087
7.3064
7.3041
7.3026
7.3000
7.2974
7.2490
3.5179
1.8959
H2O
DMSO
0.9862
0.8596
8.0041
3.2188
2.1931
3.9434
3.0297
3.0000
[ppm]

$^{13}C\{^{1}H\}$-NMR

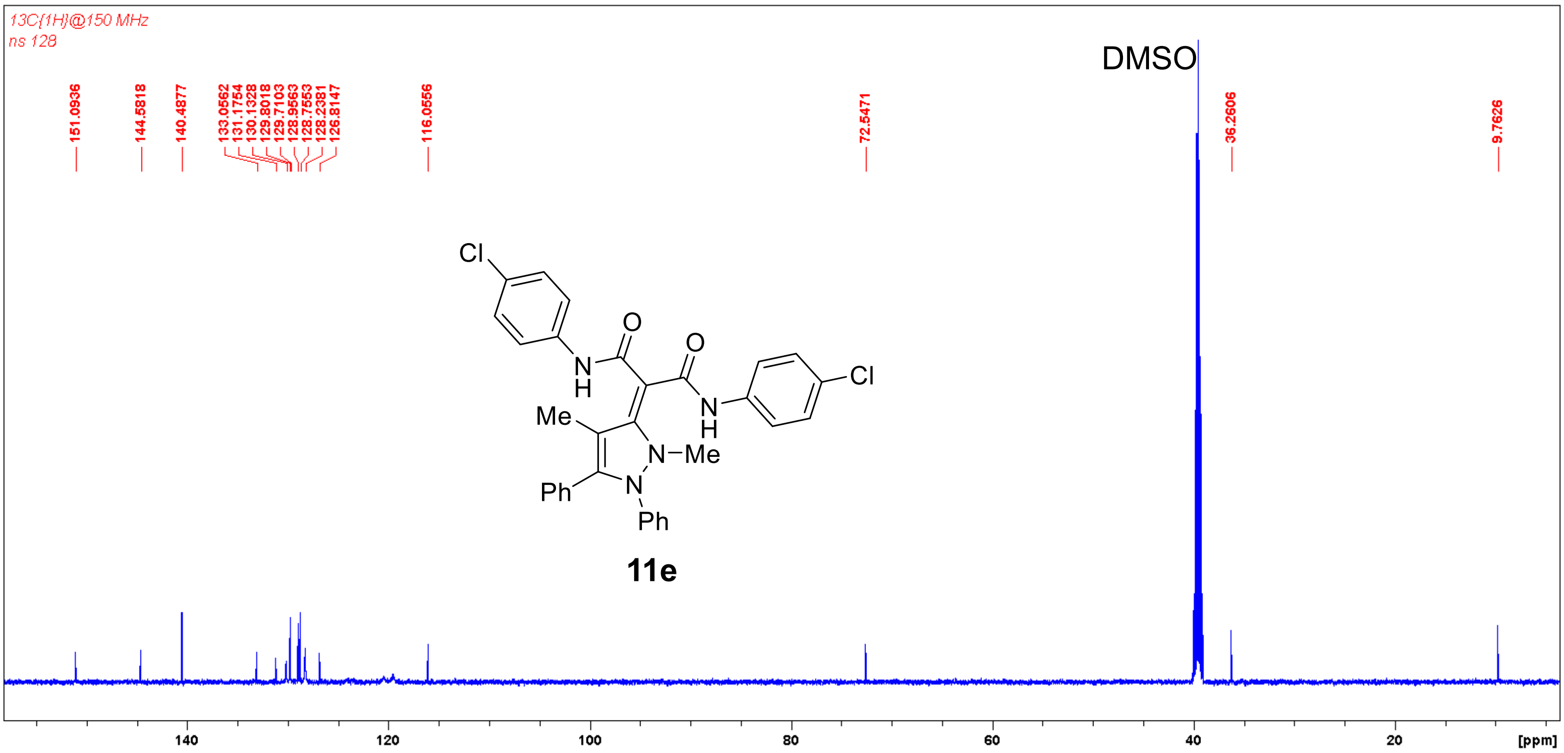

^{13}C-DEPT

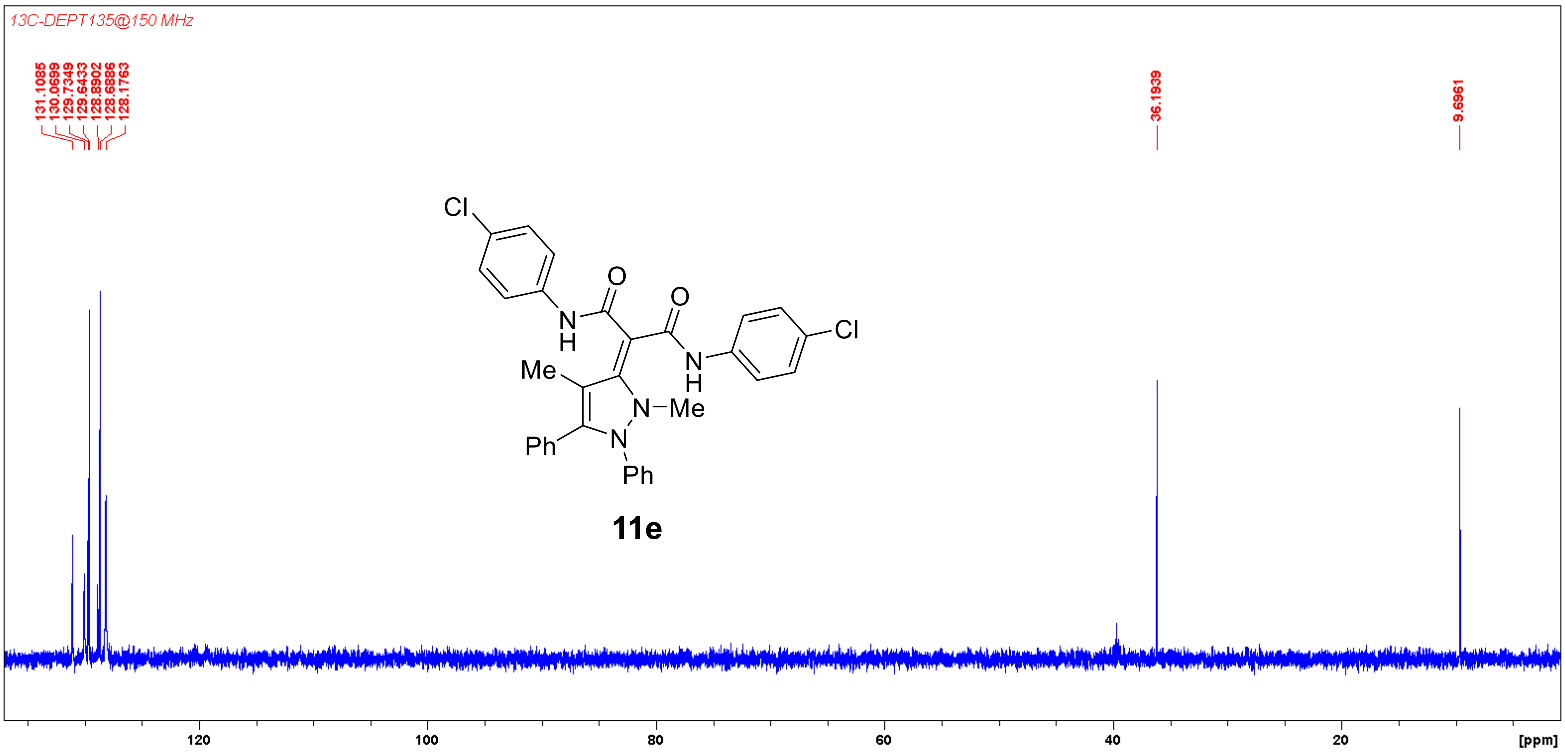

H,H-COSY

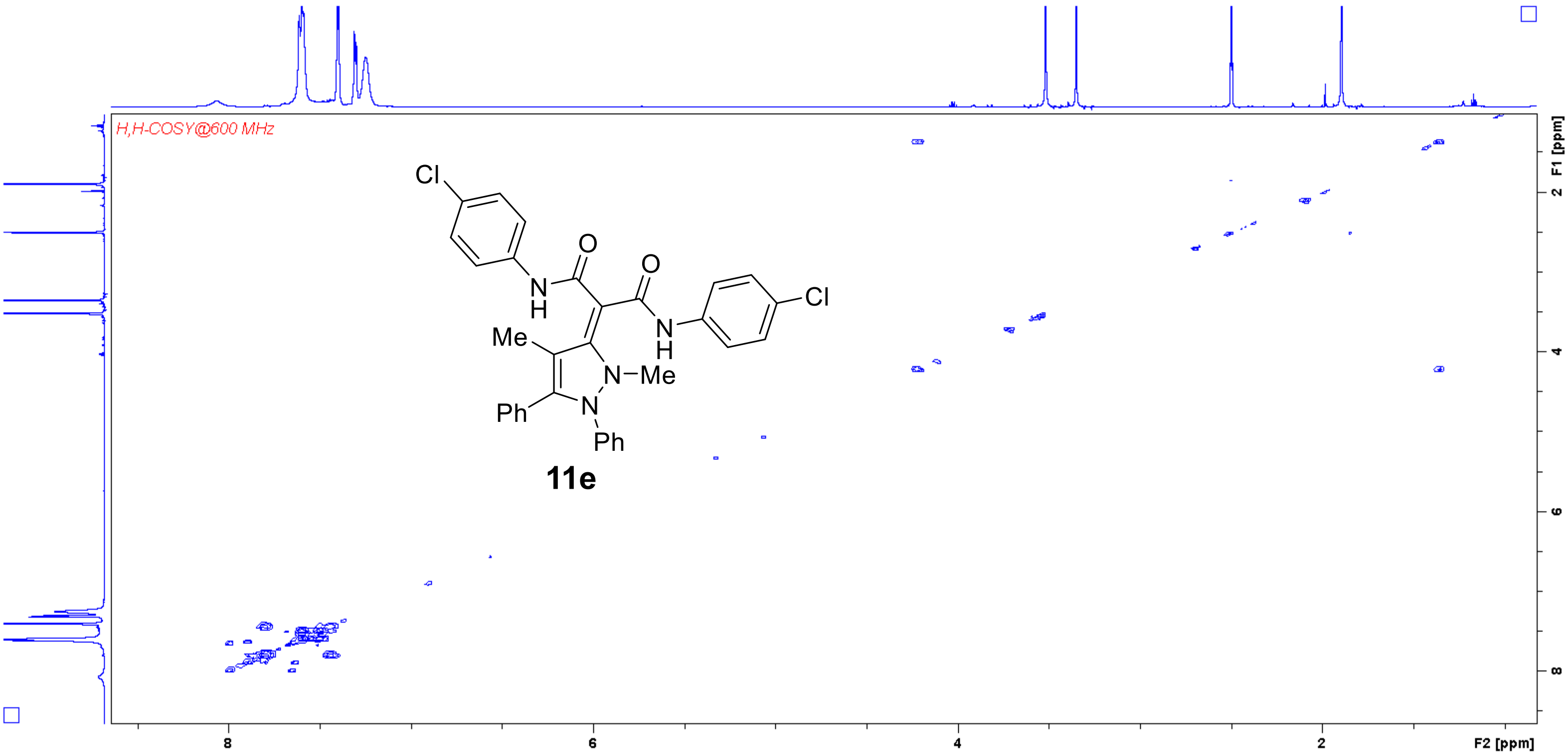
H,H-COSY@600 MHz
Cl
O
O
N
H
N
H
Cl
Me
N−Me
Ph
N
Ph
11e
F1 [ppm]
F2 [ppm]
8
6
4
2

HSQC

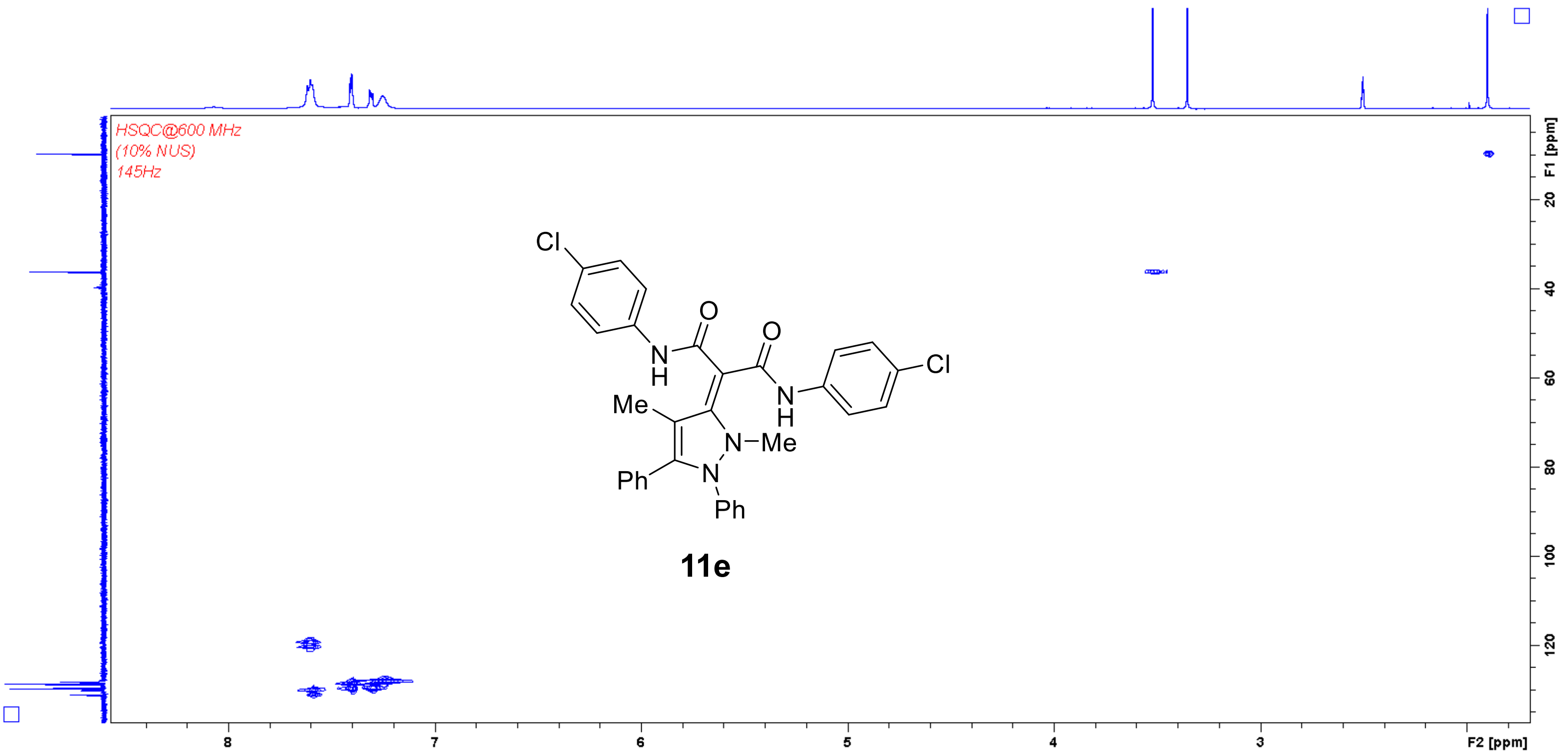
HSQC@600 MHz
(10% NUS)
145Hz
Cl
O
O
N
H
N
H
Cl
Me
N−Me
Ph
N
Ph
11e
F1 [ppm]
F2 [ppm]
20
40
60
80
100
120
8
7
6
5
4
3

HMBC

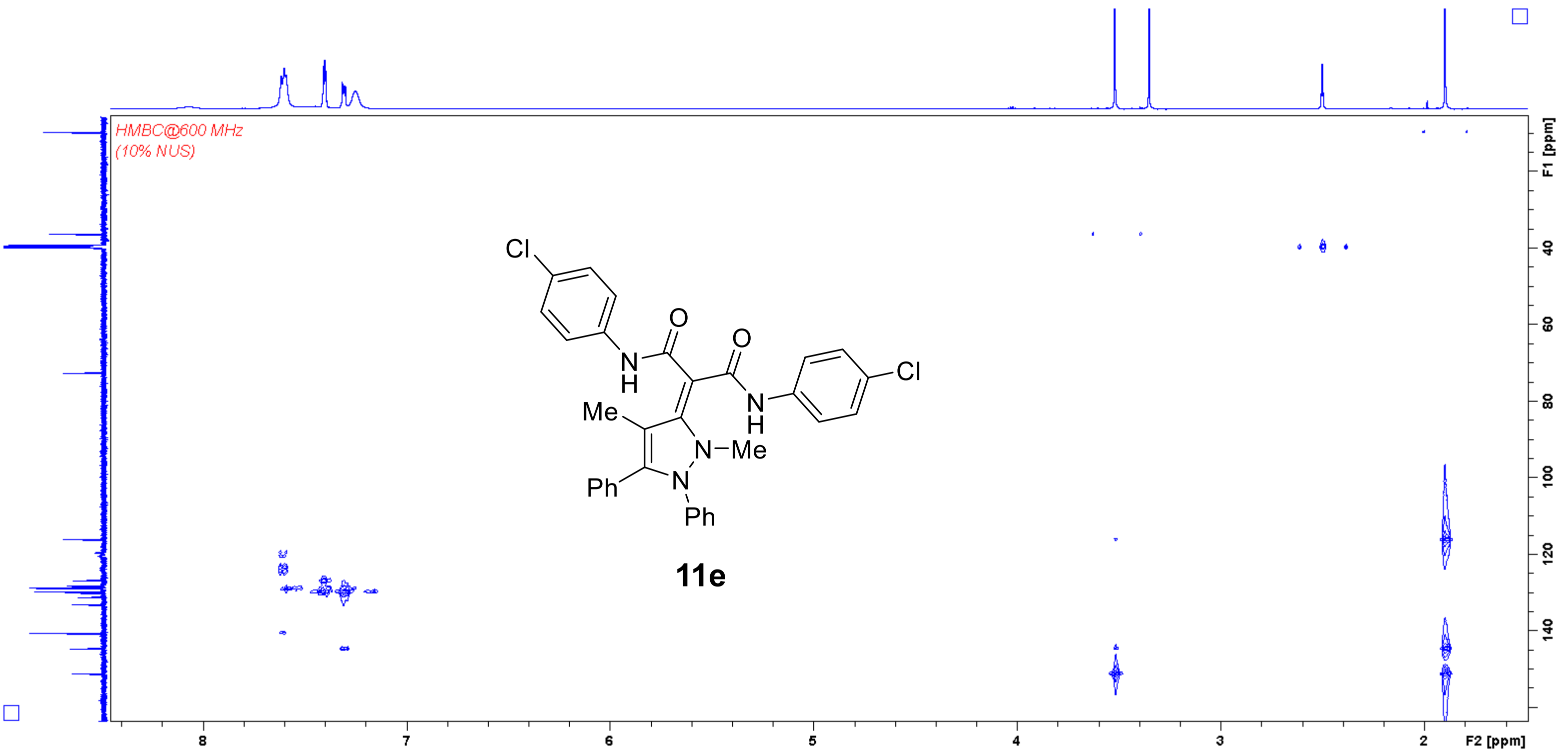
HMBC@600 MHz
(10% NUS)
Cl
O
O
N
H
Cl
Me
N
H
N−Me
Ph
N
Ph
11e
F1 [ppm]
40
60
80
100
120
140
8
7
6
5
4
3
2
F2 [ppm]

DFT calculated structures

Table S1. Calculated HOMO-LUMO energies of some NHOs and their isocyanate adducts

NHO	HOMO	LUMO	HOMO/LUMO gaps [eV]
**5**	-3.7742	1.8449	-5.6192
**6**	-4.5116	0.4408	-4.9525
**2a**	-4.9062	-0.4245	-4.4817
**2b**	-4.8763	-0.4708	-4.4055
**4a**	-4.5443	0.5061	-5.0504
**4b**	-4.6341	-0.6123	-4.0218
**12a**	-4.9117	-0.6748	-4.2368
**12b**	-5.3416	-1.3279	-4.0137

1,2-Dimethyl-3-methylene-2,3-dihydro-1*H*-indazole **2a**

vacuum

6-31G(d)/PBE0-D3

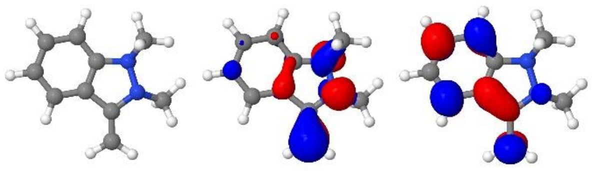

HOMO LUMO

Charge: 0
Number of imaginary frequencies: 0
E: -497.1665 H
H: -0.1803 a.u.
L: -0.0156 a.u.
HL-Gap: -4.4817 eV

ATOM	ATOMIC CHARGE	COORDINATES (BOHR) X	Y	Z
C	6.0	-14.7867611118	-3.0347811047	-0.1697994513
C	6.0	-15.3707923093	-0.7371651540	0.9986730142
C	6.0	-13.5396684807	1.1124080707	1.3804430449
C	6.0	-11.0957933970	0.6085560631	0.5403917520
C	6.0	-10.4863264859	-1.6977677456	-0.5922004835
C	6.0	-12.3323186797	-3.5282245009	-0.9730974608
N	7.0	-8.9735723766	2.1680468789	0.6590060815
N	7.0	-6.8770648596	0.6332207685	-0.1263111840
C	6.0	-7.7985614588	-1.6253458816	-1.1972378765
C	6.0	-6.4559034877	-3.2862261733	-2.5736992685
C	6.0	-5.0393307690	2.0455378240	-1.5797467902
C	6.0	-8.4797812708	3.3714943955	3.0799474489
H	1.0	-16.2551009880	-4.4432393395	-0.4380233981
H	1.0	-17.2938721008	-0.3819419958	1.6255310748
H	1.0	-14.0182869754	2.8945501981	2.2772012394
H	1.0	-11.8547848878	-5.3194221959	-1.8561759278

H	1.0	-7.3735488251	-4.9556725935	-3.3239677834
H	1.0	-4.4559817627	-3.0587711779	-2.9472773365
H	1.0	-4.5748001826	3.7953335093	-0.5866673654
H	1.0	-3.3087630470	0.9300910736	-1.7286666575
H	1.0	-5.7267148677	2.5002399448	-3.4873251584
H	1.0	-6.8255376973	4.5973805201	2.9150404985
H	1.0	-10.0952193184	4.5622920854	3.5659169782
H	1.0	-8.1532044710	1.9844732139	4.5910801732

6-311++G(2df,2p)/M06-2X//6-31G(d)/PBE0-D3

Charge: 0
E: -497.6926 H
G_{korr}: 440 kJ/mol
G: -1306251.7442 kJ/mol

DMSO

PCM/6-31G(d)/PBE0-D3

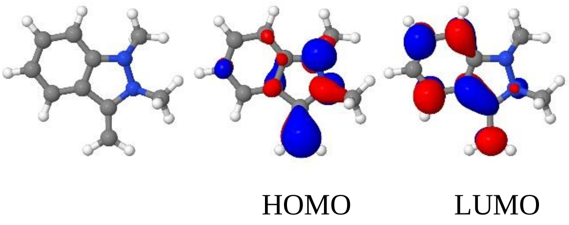

Charge: 0
Number of imaginary frequencies: 0
E: -497.1749 H
H: -0.1888 a.u.
L: -0.0255 a.u.
HL-Gap: -4.436 eV

ATOM	ATOMIC CHARGE	COORDINATES (BOHR) X	Y	Z
C	6.0	-14.7937134142	-3.0437006121	-0.1863383343
C	6.0	-15.1954124967	-1.0904607915	1.5547665005
C	6.0	-13.2990836682	0.6362159844	2.1487660033
C	6.0	-10.9627906928	0.3445424261	0.9601509471
C	6.0	-10.5611294049	-1.5702036733	-0.8194967418
C	6.0	-12.4663815202	-3.2930745407	-1.3898916755
N	7.0	-8.7728381087	1.7654766327	1.2910325430
N	7.0	-7.0587053348	0.9507552288	-0.6500450002
C	6.0	-7.9685574416	-1.3065037308	-1.7234831387
C	6.0	-6.6525597267	-2.9058507604	-3.2038322245
C	6.0	-4.4399625544	1.0067893879	0.1521361812
C	6.0	-9.0600330160	4.5019266740	1.3385005736
H	1.0	-16.3129341664	-4.3577197837	-0.6104193330
H	1.0	-17.0279441713	-0.9096215602	2.4646526224
H	1.0	-13.6341510075	2.1418685029	3.5022407667
H	1.0	-12.1492854763	-4.7845030907	-2.7668103812
H	1.0	-7.5643998254	-4.5935443760	-3.9247079393
H	1.0	-4.7071169162	-2.5650669995	-3.7465710163
H	1.0	-4.0193586517	2.8367680424	1.0089285578
H	1.0	-4.0055806585	-0.5079961770	1.5059321980
H	1.0	-3.2308250724	0.8006580621	-1.5074647659
H	1.0	-7.2275277976	5.3802335826	1.7020423061
H	1.0	-9.8346657699	5.2348720696	-0.4434072278
H	1.0	-10.3159676757	5.0013321561	2.8986830292

PCM/6-311++G(2df,2p)/M06-2X//6-31G(d)/PBE0-D3

Charge: 0
E: -497.6996 H
G_{korr}: 439 kJ/mol
G: -1306271.0450 kJ/mol

1,2,3-Trimethyl-1H-indazol-2-ium **1a**

vacuum

6-31G(d)/PBE0-D3

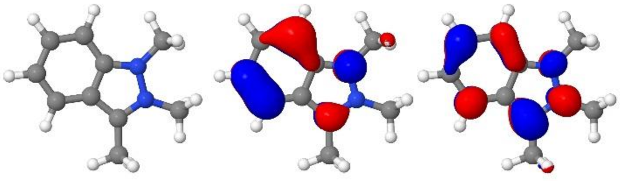

Charge: 1
Number of imaginary frequencies: 0
E: -497.5976 H
H: -0.3826 a.u.
L: -0.1987 a.u.
HL-Gap: -5.0042 eV

ATOM	ATOMIC CHARGE	COORDINATES (BOHR) X	Y	Z
C	6.0	-4.1175885054	-4.0758463451	1.8816229798
C	6.0	-4.3397201423	-2.0906644838	3.6673990506
C	6.0	-2.8905496479	0.0705396968	3.5454021114
C	6.0	-1.1640353885	0.2182444702	1.5396902655
C	6.0	-0.9065866601	-1.7487053133	-0.2533310152
C	6.0	-2.4198132009	-3.9354793782	-0.0770195677
N	7.0	0.4919637406	2.0746452755	0.9001086789
N	7.0	1.7932745040	1.2996118996	-1.1832368956
C	6.0	0.9914032377	-0.9747774273	-1.9480544120
C	6.0	2.0137337333	-2.2872564721	-4.2076982060
C	6.0	3.6729624043	2.9194208837	-2.3441901408
C	6.0	1.1266830619	4.3637177744	2.2510946730
H	1.0	-5.3136282956	-5.7291431658	2.0814312820
H	1.0	-5.7020066986	-2.2823583020	5.1898285577
H	1.0	-3.0907453439	1.5643889860	4.9341202670
H	1.0	-2.2452365220	-5.4520356165	-1.4468007778
H	1.0	1.7173150727	-1.1812980366	-5.9324814763
H	1.0	4.0396127363	-2.6701338824	-4.0165637468
H	1.0	1.0528760286	-4.0962081441	-4.4467447814
H	1.0	4.5626227874	1.8705113902	-3.8744827983
H	1.0	2.7827426622	4.6169920978	-3.1158522452
H	1.0	5.1186520231	3.4377009503	-0.9647127460
H	1.0	-0.3253995004	4.6531406689	3.6817723075
H	1.0	2.9640165305	4.1999389905	3.1867509900
H	1.0	1.1110058940	5.9919247013	0.9820642112

6-311++G(2df,2p)/M06-2X//6-31G(d)/PBE0-D3

Charge: 1
E: -498.1079 H
G_{korr}: 475 kJ/mol
G: -1307307.3311 kJ/mol

DMSO

PCM/6-31G(d)/PBE0-D3

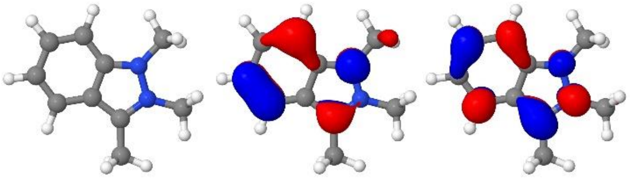

Charge: 1
Number of imaginary frequencies: 0
E: -497.6682 H
H: -0.2585 a.u.
L: -0.0700 a.u.
HL-Gap: -5.1293 eV

ATOM	ATOMIC CHARGE	COORDINATES (BOHR) X	Y	Z
C	6.0	-4.0635820225	-4.0985797504	1.9287678672
C	6.0	-4.3045901336	-2.0960388649	3.6953556589
C	6.0	-2.8708341353	0.0766888656	3.5466682279
C	6.0	-1.1433409977	0.2079019991	1.5377381784
C	6.0	-0.8717703459	-1.7686457034	-0.2369716561
C	6.0	-2.3652946022	-3.9652501236	-0.0383973451
N	7.0	0.4913439104	2.0671109374	0.8831559458
N	7.0	1.7792376184	1.3051223410	-1.2045492269
C	6.0	1.0118840895	-0.9804900694	-1.9546854610
C	6.0	1.9866275017	-2.3397133796	-4.1999824542
C	6.0	3.6504217511	2.9710935549	-2.3084232944
C	6.0	1.0025677397	4.4427801360	2.1278108303
H	1.0	-5.2470551339	-5.7581353440	2.1439887756
H	1.0	-5.6637397446	-2.2815192636	5.2198978798
H	1.0	-3.0622331562	1.5947663334	4.9076792190
H	1.0	-2.1721324668	-5.4893161335	-1.3939583662
H	1.0	3.5304468188	-1.3449350910	-5.1278871147
H	1.0	2.6567999749	-4.2118291473	-3.6435658247
H	1.0	0.4624084240	-2.5928062893	-5.5724980981
H	1.0	5.0128991697	3.5216765999	-0.8655682648
H	1.0	4.6206241514	1.9511252170	-3.8004168726
H	1.0	2.7315745479	4.6388883544	-3.0974727690
H	1.0	-0.4720403581	4.7114084842	3.5356076609
H	1.0	2.8441738791	4.4079033507	3.0575523046
H	1.0	0.9122048158	5.9918831274	0.7715033674

PCM/6-311++G(2df,2p)/M06-2X//6-31G(d)/PBE0-D3

Charge: 1
E: -498.1770 H
G_{korr}: 471 kJ/mol
G: -1307492.2649 kJ/mol

2-Methyl-3-methylene-1-phenyl-2,3-dihydro-1*H*-indazole **2b**

vacuum

6-31G(d)/PBE0-D3

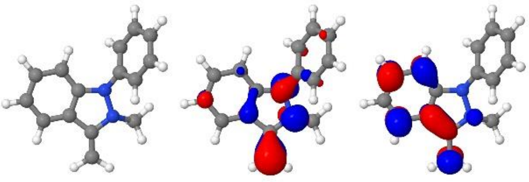

Charge: 0
Number of imaginary frequencies: 0
E: -688.6895 H
H: -0.1792 a.u.
L: -0.0173 a.u.
HL-Gap: -4.4055 eV

ATOM	ATOMIC CHARGE	COORDINATES (BOHR) X	Y	Z
C	6.0	-5.5578300439	-4.7790663487	-0.8771277195
C	6.0	-6.0806908066	-2.4946935986	0.3444876240
C	6.0	-4.2896745253	-0.5754064874	0.5060818845
C	6.0	-1.9445489701	-1.0010861944	-0.6108369625
C	6.0	-1.3805413107	-3.2994712637	-1.7862371639
C	6.0	-3.1923351307	-5.1952123874	-1.9452708455
N	7.0	0.1494603290	0.5962142618	-0.7521223363
N	7.0	2.2005785142	-0.8785204476	-1.7076604619
C	6.0	1.2281065528	-3.1681277391	-2.6635103923
C	6.0	2.4743110126	-4.8104773763	-4.1473422433
C	6.0	3.8606311058	0.5836891570	-3.3207949333
C	6.0	0.7708551914	2.2372675469	1.2757862326
C	6.0	-0.7098132581	4.3906917251	1.6484591218
C	6.0	-0.1678927176	6.0259473305	3.6338129482
C	6.0	1.8861054102	5.5651470634	5.2129852616
C	6.0	3.3914347856	3.4451408021	4.7975799955
C	6.0	2.8412051194	1.7739010318	2.8461864374
H	1.0	2.9596644913	1.0782474916	-5.1258499900
H	1.0	4.4196479985	2.3048498223	-2.3261054618
H	1.0	5.5642702305	-0.5212091421	-3.6872600722
H	1.0	-2.7481682330	-6.9802382369	-2.8575096764
H	1.0	-6.9967771199	-6.2390630840	-0.9707050675
H	1.0	-7.9258364031	-2.2045866233	1.1980069950
H	1.0	-4.7039327277	1.1769875713	1.4845575058
H	1.0	3.9835445622	0.1036495883	2.5129218968
H	1.0	5.0020540315	3.0745919650	6.0170599485
H	1.0	2.3239511740	6.8598116601	6.7445893890
H	1.0	-1.3356451974	7.6909453354	3.9182696424
H	1.0	-2.2495243106	4.7862983306	0.3503042011
H	1.0	1.5319442781	-6.5111571899	-4.7881464827
H	1.0	4.4196839033	-4.5349855429	-4.7219210306

6-311++G(2df,2p)/M06-2X//6-31G(d)/PBE0-D3

Charge: 0
E: -689.4197 H
G_{korr}: 567 kJ/mol
G: -1809504.1921 kJ/mol

DMSO

PCM/6-31G(d)/PBE0-D3

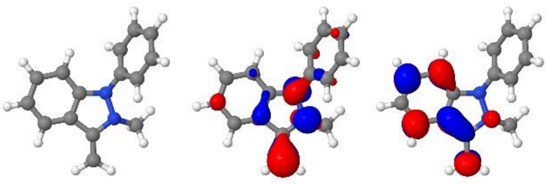

HOMO LUMO

Charge: 0
Number of imaginary frequencies: 0
E: -688.6985 H
H: -0.1885 a.u.
L: -0.0279 a.u.
HL-Gap: -4.3701 eV

ATOM	ATOMIC CHARGE	COORDINATES (BOHR) X	Y	Z
C	6.0	-5.5874231550	-4.7603448320	-0.9035196346
C	6.0	-6.0956517684	-2.4832758734	0.3441890473
C	6.0	-4.2939264091	-0.5714437317	0.5225395093
C	6.0	-1.9481923621	-0.9973350881	-0.6029152306
C	6.0	-1.4019235618	-3.2904837262	-1.8054783553
C	6.0	-3.2253259694	-5.1782558749	-1.9846187228
N	7.0	0.1582702322	0.5834567207	-0.7340112011
N	7.0	2.1967631571	-0.8987480761	-1.7047276070
C	6.0	1.2048629214	-3.1616421990	-2.6941390734
C	6.0	2.4323212981	-4.7951365796	-4.2097277719
C	6.0	3.9079800836	0.5564071809	-3.2767567557
C	6.0	0.7857292257	2.2304361870	1.2894432833
C	6.0	-0.7386183534	4.3481086366	1.7087357161
C	6.0	-0.1914481538	5.9835834502	3.6953632178
C	6.0	1.9094189614	5.5633574928	5.2280690555
C	6.0	3.4574296911	3.4821510882	4.7646326205
C	6.0	2.9027912938	1.8083450699	2.8124529364
H	1.0	3.0438687977	1.0759136798	-5.0924793163
H	1.0	4.4748525678	2.2620059516	-2.2601105563
H	1.0	5.6033025236	-0.5673732616	-3.6205036071
H	1.0	-2.7959367300	-6.9536819156	-2.9238825271
H	1.0	-7.0357546110	-6.2106019189	-1.0108504093
H	1.0	-7.9373882989	-2.1910524048	1.2048251269
H	1.0	-4.7110210904	1.1759614501	1.5101500667
H	1.0	4.0906599082	0.1754327249	2.4485578250
H	1.0	5.1069395006	3.1431965823	5.9404731281
H	1.0	2.3482095883	6.8585323155	6.7588643802
H	1.0	-1.3949410238	7.6158003760	4.0169454715
H	1.0	-2.3231650479	4.7205509792	0.4574932463
H	1.0	1.4688784481	-6.4825864206	-4.8598483611
H	1.0	4.3715431303	-4.5109709033	-4.8043376562

PCM/6-311++G(2df,2p)/M06-2X//6-31G(d)/PBE0-D3

Charge: 0
E: -689.4269 H
G_{korr}: 565 kJ/mol
G: -1809525.0134 kJ/mol

2,3-Dimethyl-1-phenyl-1H-indazol-2-ium**1b**

vacuum

6-31G(d)/PBE0-D3

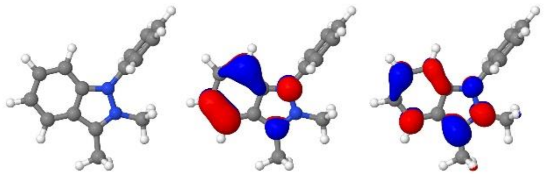

HOMO LUMO

Charge: 1
Number of imaginary frequencies: 0
E: -689.1238 H
H: -0.3746 a.u.
L: -0.1897 a.u.
HL-Gap: -5.0314 eV

ATOM	ATOMIC CHARGE	COORDINATES (BOHR) X	Y	Z
C	6.0	-5.7377111840	-4.6799710004	-0.7537021473
C	6.0	-6.0240878400	-2.7185618938	1.0511204730
C	6.0	-4.1528205593	-0.9673848187	1.5176352719
C	6.0	-1.9313889173	-1.2325209530	0.1017674210
C	6.0	-1.6024102756	-3.1712212207	-1.7061808063
C	6.0	-3.5552343564	-4.9324856539	-2.1392814679
N	7.0	0.2159484530	0.1754365043	0.1383279524
N	7.0	1.8548247736	-0.8235029612	-1.5905465750
C	6.0	0.8345767563	-2.8346080854	-2.7292993177
C	6.0	2.1690692208	-4.3328056344	-4.6921408366
C	6.0	4.3273405465	0.2997332402	-1.9546419973
C	6.0	0.7964137372	2.3584103299	1.6113959233
C	6.0	0.0615937333	4.7287429420	0.7290261036
C	6.0	0.6164400005	6.8489854191	2.1806173371
C	6.0	1.8869312205	6.5814852374	4.4723203348
C	6.0	2.6008395151	4.1978395048	5.3371818420
C	6.0	2.0520422613	2.0615797090	3.9075530055
H	1.0	-3.3385300130	-6.4335574763	-3.5203575710
H	1.0	-7.2816760100	-5.9997141524	-1.0348556003
H	1.0	-7.7825441401	-2.5967822731	2.1016948152
H	1.0	-4.3713749446	0.5336567681	2.8958484400
H	1.0	2.5659627297	0.1844051445	4.5608105401
H	1.0	3.5742015375	3.9999114802	7.1319567886
H	1.0	2.3154814215	8.2423239551	5.5985800981
H	1.0	0.0530710685	8.7084589190	1.5226846201
H	1.0	-0.9371699877	4.8945607403	-1.0568368946
H	1.0	4.1227228913	2.2922888128	-2.4517268957
H	1.0	5.4429384749	0.1316931240	-0.2256597555
H	1.0	5.2650302088	-0.7035166906	-3.4868073734
H	1.0	2.6595759825	-3.1732432277	-6.3355222644
H	1.0	3.9061886232	-5.1815251011	-3.9520711736
H	1.0	0.9463672847	-5.8610441596	-5.3422746539

6-311++G(2df,2p)/M06-2X//6-31G(d)/PBE0-D3

Charge: 1
E: -689.8374 H
G_{korr}: 601 kJ/mol
G: -1810567.4750 kJ/mol

DMSO

PCM/6-31G(d)/PBE0-D3

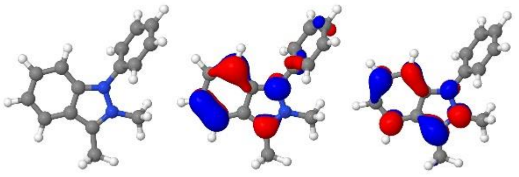	Charge: 1<br>Number of imaginary frequencies: 0<br>E: -689.1878 H<br>H: -0.2594 a.u.<br>L: -0.0743 a.u.<br>HL-Gap: -5.0368 eV

ATOM ATOMIC CHARGE COORDINATES (BOHR) X Y Z

C	6.0	-5.7776127512	-4.5777084711	-0.8111347037
C	6.0	-6.1083734046	-2.5283609593	0.8838740418
C	6.0	-4.2265255474	-0.7804039775	1.3432607937
C	6.0	-1.9556000885	-1.1351112404	0.0254508315
C	6.0	-1.5819010780	-3.1705012351	-1.6563090442
C	6.0	-3.5353884526	-4.9275912632	-2.0876106864
N	7.0	0.2145406070	0.2417110893	0.0775675883
N	7.0	1.9090807004	-0.8753948406	-1.5192698850
C	6.0	0.9105966589	-2.9283082657	-2.5870539630
C	6.0	2.2397279703	-4.5754086744	-4.4176902423
C	6.0	4.3650916053	0.2691518023	-1.9389440424
C	6.0	0.8030296684	2.3776515213	1.5998024536
C	6.0	-0.4992675321	4.6272684285	1.1791040646
C	6.0	0.0123380219	6.6963730269	2.7219955262
C	6.0	1.8238143679	6.5104485426	4.6260665627
C	6.0	3.1193482386	4.2477093773	5.0178559213
C	6.0	2.5976515471	2.1537265344	3.5163778078
H	1.0	-3.2686347128	-6.4987681454	-3.3749582635
H	1.0	-7.3282369721	-5.8874077288	-1.0961280802
H	1.0	-7.9032655142	-2.3297753099	1.8556071203
H	1.0	-4.4798981366	0.7882784662	2.6354574080
H	1.0	3.5556198605	0.3675800773	3.8234942080
H	1.0	4.5191156229	4.1020303903	6.5087288919
H	1.0	2.2277225403	8.1342448488	5.8118375810
H	1.0	-0.9933515454	8.4567511786	2.4184110241
H	1.0	-1.8780759639	4.7448396291	-0.3332399741
H	1.0	5.6282280112	-0.1364363365	-0.3622510496
H	1.0	5.1361527768	-0.5074349284	-3.6761598209
H	1.0	4.1267763539	2.3035459113	-2.1485789204
H	1.0	1.3463051425	-4.4306499840	-6.2756954250
H	1.0	4.2362122835	-4.1101354256	-4.6013092607
H	1.0	2.0872743152	-6.5401040147	-3.7995967314

PCM/6-311++G(2df,2p)/M06-2X//6-31G(d)/PBE0-D3

Charge: 1
E: -689.9006 H
G_{korr}: 604 kJ/mol
G: -1810730.0459 kJ/mol

1,2,4,5-Tetramethyl-3-methylene-2,3-dihydro-1*H*-pyrazole **4a**

vacuum

6-31G(d)/PBE0-D3

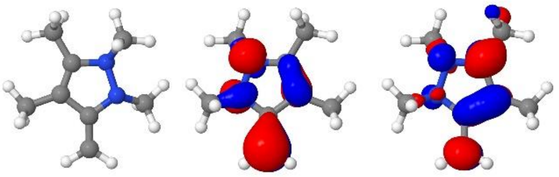

HOMO LUMO

Charge: 0
Number of imaginary frequencies: 0
E: -422.2327 H
H: -0.1670 a.u.
L: 0.0186 a.u.
HL-Gap: -5.0504 eV

ATOM	ATOMIC CHARGE	COORDINATES (BOHR) X	Y	Z
C	6.0	-1.8636290077	0.0113345773	0.6235434810
C	6.0	-1.0075112632	-1.7474656530	-1.0166839939
C	6.0	-2.4025411041	-3.8241707299	-2.2972493439
N	7.0	0.0256662602	1.7680806753	1.2174220413
N	7.0	2.2882901523	0.8299828321	0.0884920950
C	6.0	1.6340064964	-1.1820274709	-1.5259330593
C	6.0	3.1474106715	-2.2697878438	-3.2625497946
C	6.0	3.8506873669	2.8213970101	-0.9482437828
C	6.0	-4.4106585713	0.2998655210	1.7821648041
H	1.0	-1.5013892964	-5.6532271981	-1.9223106730
H	1.0	-4.3705529138	-3.9425979765	-1.6779142830
H	1.0	-2.3936329352	-3.5618710746	-4.3557223431
C	6.0	0.4070526766	2.3086765177	3.8884648819
H	1.0	2.4255012765	-3.7935968510	-4.4233764282
H	1.0	5.0979198378	-1.7121693484	-3.5406154351
H	1.0	4.1399458552	4.2866169485	0.4794367461
H	1.0	5.6977699327	2.0342504898	-1.4270758165
H	1.0	3.0145610352	3.6748256743	-2.6485040772
H	1.0	-5.7748915456	-0.9421078420	0.8567319054
H	1.0	-4.3978197720	-0.1747070700	3.8008817452
H	1.0	-5.0830628110	2.2506335798	1.6025652332
H	1.0	1.9022965836	3.7198011560	4.0866914833
H	1.0	-1.3270790689	3.1199397229	4.6640897420
H	1.0	0.9380694973	0.6136677723	4.9680597482

6-311++G(2df,2p)/M06-2X//6-31G(d)/PBE0-D3

Charge: 0
E: -422.6769 H
G_{korr}: 464 kJ/mol
G: -1109273.9400 kJ/mol

DMSO

PCM/6-31G(d)/PBE0-D3

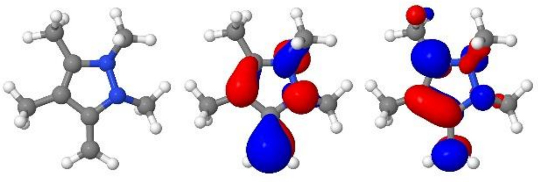

Charge: 0
Number of imaginary frequencies: 0
E: -422.2402 H
H: 0.0094 a.u.
L: -0.1779 a.u.
HL-Gap: -5.0967 eV

ATOM	ATOMIC CHARGE	COORDINATES (BOHR) X	Y	Z
C	6.0	-1.8659117968	-0.0022544433	0.6321889780
C	6.0	-1.0130065868	-1.7562453206	-1.0262214416
C	6.0	-2.4127059410	-3.8350876778	-2.3031320613
N	7.0	0.0174232749	1.7634527360	1.1995924753
N	7.0	2.2813567471	0.8129072668	0.0871541689
C	6.0	1.6216287903	-1.1738411774	-1.5483962338
C	6.0	3.1402353814	-2.2468768043	-3.3011890247
C	6.0	3.9164762923	2.7848194712	-0.8811962998
C	6.0	-4.4007148325	0.2598089964	1.8227239960
H	1.0	-1.5040651486	-5.6651079063	-1.9477482764
H	1.0	-4.3723821687	-3.9584508890	-1.6594252025
H	1.0	-2.4281676801	-3.5664593296	-4.3611723133
C	6.0	0.3765543867	2.4032705384	3.8549808247
H	1.0	2.4156217884	-3.7712376114	-4.4628376891
H	1.0	5.0850148981	-1.6666288385	-3.5874031642
H	1.0	4.2192519915	4.2128533789	0.5803651287
H	1.0	5.7528819054	1.9611388757	-1.3410951675
H	1.0	3.1327993091	3.6917387231	-2.5787334989
H	1.0	-5.7589120214	-1.0069859194	0.9224055575
H	1.0	-4.3422996185	-0.2036066517	3.8426087878
H	1.0	-5.1014819716	2.2012871615	1.6492943808
H	1.0	1.8599648287	3.8314934187	4.0160950947
H	1.0	-1.3714233821	3.2247118084	4.5844774701
H	1.0	0.8995927837	0.7484562676	4.9980723785

PCM/6-311++G(2df,2p)/M06-2X//6-31G(d)/PBE0-D3

Charge: 0
E: -422.6837 H
G_{korr}: 463 kJ/mol
G: -1109293.3257 kJ/mol

1,2,3,4,5-Pentamethyl-1*H*-pyrazol-2-ium **3a**

vacuum

6-31G(d)/PBE0-D3

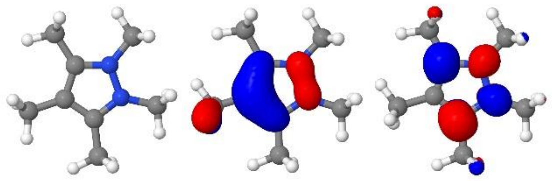

HOMO LUMO

Charge: 1
Number of imaginary frequencies: 0
E: -422.6797 H
H: -0.4112 a.u.
L: -0.1719 a.u.
HL-Gap: -6.5117 eV

ATOM	ATOMIC CHARGE	COORDINATES (BOHR) X	Y	Z
C	6.0	-1.9144872069	-0.1799189347	0.9097141568
C	6.0	-1.1365833371	-1.7308511809	-1.0791205450
C	6.0	-2.6617472778	-3.7816273257	-2.2753474181
N	7.0	-0.0173609139	1.4308325921	1.4437923338
N	7.0	1.9429937255	0.9573692702	-0.1315986377
C	6.0	1.3098409871	-0.9578813860	-1.6927845378
C	6.0	3.0720918574	-1.9152771124	-3.6595566872
C	6.0	4.2587094730	2.4125604320	-0.0221494799
C	6.0	-4.3516046299	-0.1666870723	2.3044321929
C	6.0	0.1109420414	3.3820485159	3.3593510154
H	1.0	-1.5386452470	-4.8730915618	-3.6192639467
H	1.0	-3.3771616841	-5.1063650239	-0.8570323719
H	1.0	-4.2852204409	-3.0119210878	-3.3026724597
H	1.0	3.5517383631	-0.4426267709	-5.0335406482
H	1.0	4.8284203259	-2.6300943652	-2.8286403303
H	1.0	2.1930725220	-3.4672146929	-4.6924885462
H	1.0	3.8948578254	4.3990594324	-0.4584003149
H	1.0	5.1390157119	2.2424208301	1.8387129687
H	1.0	5.5481659845	1.6509970240	-1.4332873463
H	1.0	-5.5869847385	-1.6336587868	1.5459849432
H	1.0	-4.0823564516	-0.5476634181	4.3215901099
H	1.0	-5.3341696186	1.6453958758	2.1091327773
H	1.0	1.5992808892	2.9637878737	4.7299863817
H	1.0	0.4377663953	5.2296715433	2.4957178302
H	1.0	-1.6989696114	3.4222845646	4.3380590731

6-311++G(2df,2p)/M06-2X//6-31G(d)/PBE0-D3

Charge: 1
E: -423.10874 H
G_{korr}: 498 kJ/mol
G: -1110373.6721 kJ/mol

DMSO

PCM/6-31G(d)/PBE0-D3

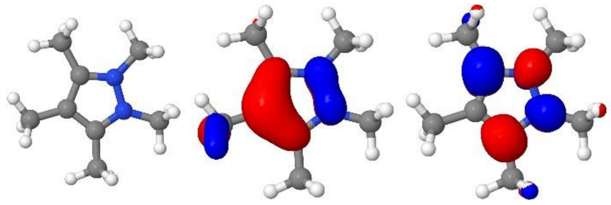

Charge: 1
Number of imaginary frequencies: 0
E: -422.7508 H
H: -0.2785 a.u.
L: -0.0353 a.u.
HL-Gap: -6.6178 eV

ATOM	ATOMIC CHARGE	COORDINATES (BOHR) X	Y	Z
C	6.0	-1.9315098598	-0.1802364087	0.8605378138
C	6.0	-1.1706532094	-1.6997878629	-1.1612688294
C	6.0	-2.6944149734	-3.7167057846	-2.4130498711
N	7.0	-0.0023886138	1.3680785669	1.4451548264
N	7.0	1.9527277048	0.8970473226	-0.1251905764
C	6.0	1.3014298162	-0.9665722365	-1.7311044042
C	6.0	3.0723261834	-1.9394164739	-3.6771953908
C	6.0	4.3145187547	2.2535796628	0.0972018427
C	6.0	-4.3635288018	-0.1377119017	2.2568186534
C	6.0	0.1589807692	3.2939154689	3.3780404068
H	1.0	-1.9138409205	-4.1925709484	-4.2640007062
H	1.0	-2.7298737944	-5.4485207259	-1.2811946288
H	1.0	-4.6491646851	-3.1064565270	-2.6909492157
H	1.0	3.8640458409	-0.4094375110	-4.8188753192
H	1.0	4.6361539207	-2.9772786285	-2.8077399594
H	1.0	2.0721565062	-3.2306285409	-4.9339464122
H	1.0	4.0076272319	4.2751784362	-0.1710901342
H	1.0	5.1663165852	1.9047607865	1.9432204926
H	1.0	5.5699753137	1.5570889740	-1.3727424110
H	1.0	-5.6436217203	-1.5336015679	1.4443176777
H	1.0	-4.0844124736	-0.6036163190	4.2519820480
H	1.0	-5.2692688645	1.7178196296	2.1479005087
H	1.0	1.7128156348	2.8758608068	4.6668203963
H	1.0	0.4291284572	5.1475383767	2.5161306518
H	1.0	-1.6106381432	3.2785198701	4.4232611549

PCM/6-311++G(2df,2p)/M06-2X//6-31G(d)/PBE0-D3

Charge: 1
E: -423.1781 H
G_{korr}: 496 kJ/mol
G: -1110557.7685 kJ/mol

2,4-Dimethyl-3-methylene-1,5-diphenyl-2,3-dihydro-1*H*-pyrazole **4b**

vacuum

6-31G(d)/PBE0-D3

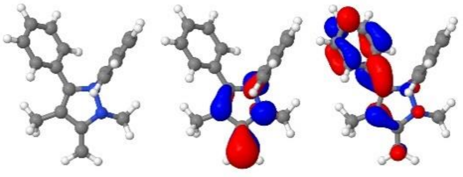

HOMO LUMO

Charge: 0
Number of imaginary frequencies: 0
E: -805.2745 H
H: -0.1703 a.u.
L: -0.0225 a.u.
HL-Gap: -4.0218 eV

ATOM	ATOMIC CHARGE	COORDINATES (BOHR) X	Y	Z
C	6.0	-0.7750957368	-1.0724876063	-1.1573098532
C	6.0	-0.3409217108	-3.3920187109	-2.1498544855
C	6.0	-2.1809102447	-5.4375074020	-2.7297131677
N	7.0	1.4208642867	0.4312506196	-1.2440690694
N	7.0	3.3927197993	-1.2338210846	-2.0312231486
C	6.0	2.3007888009	-3.4823476197	-2.9100497318
C	6.0	3.4679800320	-5.2880773087	-4.2779827898
C	6.0	5.3202404473	0.0014569788	-3.5138550234
C	6.0	2.0955909998	1.7383911882	1.0528703594
C	6.0	2.8065834470	0.3967782357	3.2075285288
C	6.0	3.4465562069	1.6974956251	5.3950093512
C	6.0	3.3843558715	4.3341322222	5.4332876436
C	6.0	2.6911665342	5.6641063515	3.2748008891
C	6.0	2.0545385895	4.3632585709	1.0740485201
C	6.0	-3.1228139963	0.0102460951	-0.1648842736
H	1.0	-2.0552094423	-5.9647504397	-4.7319025640
H	1.0	-1.7800124061	-7.1500509162	-1.6312607244
H	1.0	-4.1218555070	-4.8497647825	-2.3387363912
H	1.0	2.4354658024	-6.9639336799	-4.8385511477
H	1.0	5.4395728727	-5.1654813263	-4.8164337931
H	1.0	4.6741752104	0.4375396282	-5.4408559967
H	1.0	5.8813398182	1.7441548528	-2.5568920443
H	1.0	6.9724601241	-1.2323263112	-3.6169962754
H	1.0	2.8601174984	-1.6539979091	3.1252989861
H	1.0	3.9895179865	0.6580971232	7.0810248900
H	1.0	3.8847723571	5.3470235357	7.1480251298
H	1.0	2.6531433548	7.7168704881	3.2946354545
H	1.0	1.5232156332	5.3621281166	-0.6381831893
C	6.0	-3.8606764592	2.4618614969	-0.8349490324
C	6.0	-6.0984655706	3.4773474044	0.0889380704
C	6.0	-7.6300545802	2.0629894447	1.6982137210
C	6.0	-6.9062119954	-0.3721796707	2.3837439984
C	6.0	-4.6633376310	-1.3899653749	1.4663972377
H	1.0	-2.6723108469	3.5443344161	-2.1102137006
H	1.0	-6.6549710173	5.3781888989	-0.4536967866
H	1.0	-9.3777413311	2.8614818807	2.4226496798

H	1.0	-8.0802194686	-1.4773122958	3.6554729890
H	1.0	-4.0655945808	-3.2675197744	2.0422421416

6-311++G(2df,2p)/M06-2X//6-31G(d)/PBE0-D3

Charge: 0
E: -806.1274 H
G_{korr}: 718 kJ/mol
G: -2115769.6836 kJ/mol

DMSO

PCM/6-31G(d)/PBE0-D3

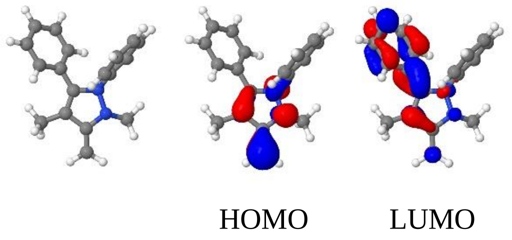

Charge: 0
Number of imaginary frequencies: 0
E: -805.2849 H
H: -0.1827 a.u.
L: -0.0335 a.u.
HL-Gap: -4.0599 eV

ATOM	ATOMIC CHARGE	COORDINATES (BOHR) X	Y	Z
C	6.0	-0.7760028053	-1.1049663293	-1.1451853704
C	6.0	-0.3218052414	-3.4104794455	-2.1731170141
C	6.0	-2.1329849004	-5.4796520741	-2.7672865922
N	7.0	1.3953057409	0.4249351549	-1.2349700381
N	7.0	3.3938498556	-1.2024629692	-2.0502508009
C	6.0	2.3200980224	-3.4561333389	-2.9395785923
C	6.0	3.5098280171	-5.2438237022	-4.3210213023
C	6.0	5.2767861951	0.0763317074	-3.5641085103
C	6.0	2.0690006635	1.7585224406	1.0451753946
C	6.0	2.2905332572	0.4849793128	3.3440763591
C	6.0	2.9499021661	1.8175007929	5.5105717729
C	6.0	3.3998629641	4.4152317086	5.3795740682
C	6.0	3.1874879832	5.6757054904	3.0791783303
C	6.0	2.5186742232	4.3443915453	0.9038465572
C	6.0	-3.1231749340	-0.0453156325	-0.1251149873
H	1.0	-1.9384621622	-6.0550925765	-4.7502593636
H	1.0	-1.7705940111	-7.1621357148	-1.6115282042
H	1.0	-4.0891991499	-4.8935950902	-2.4546106178
H	1.0	2.4919950697	-6.9279362869	-4.8890238427
H	1.0	5.4787147699	-5.0851830837	-4.8670519971
H	1.0	4.5631538005	0.5694916446	-5.4524456870
H	1.0	5.8745594809	1.7932574965	-2.5818798929
H	1.0	6.9232478764	-1.1535247318	-3.7588353389
H	1.0	1.9394693863	-1.5387321744	3.4091131735
H	1.0	3.1124507382	0.8339059035	7.3062896928
H	1.0	3.9121847242	5.4531316576	7.0760605795
H	1.0	3.5327692922	7.6971568652	2.9724617166
H	1.0	2.3274811824	5.2947971748	-0.9066679183
C	6.0	-3.7854332341	2.4705088837	-0.6311912026
C	6.0	-6.0243675195	3.4735698419	0.3105405839
C	6.0	-7.6303720542	1.9838193687	1.7769907340
C	6.0	-6.9768348401	-0.5123803417	2.3069511978
C	6.0	-4.7339869320	-1.5198878254	1.3723984808
H	1.0	-2.5390624783	3.6235403969	-1.7865508585
H	1.0	-6.5197327671	5.4232928821	-0.1042221753
H	1.0	-9.3795101148	2.7693955266	2.5126686735
H	1.0	-8.2072808738	-1.6760906972	3.4685450601
H	1.0	-4.1990961727	-3.4464277056	1.8364566357

PCM/6-311++G(2df,2p)/M06-2X//6-31G(d)/PBE0-D3

Charge: 0
E: -806.1355 H
G_{korr}: 713 kJ/mol
G: -2115795.8497 kJ/mol

2,3,4-Trimethyl-1,5-diphenyl-1H-pyrazol-2-ium **3b**

vacuum

6-31G(d)/PBE0-D3

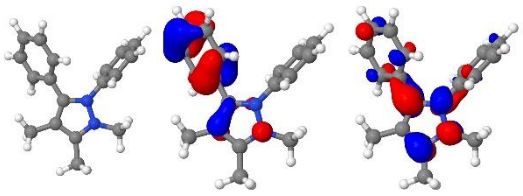

HOMO LUMO

Charge: 1
Number of imaginary frequencies: 0
E: -805.7265 H
H: -0.3673 a.u.
L: -0.1693 a.u.
HL-Gap: -5.3879 eV

ATOM	ATOMIC CHARGE	COORDINATES (BOHR) X	Y	Z
C	6.0	-0.7250009869	-1.1486945918	-1.0961828822
C	6.0	-0.2553152276	-2.7636772153	-3.1375179553
C	6.0	-2.1474280772	-4.2932706742	-4.5655575321
N	7.0	1.5238940448	-0.2219615615	-0.2968476284
N	7.0	3.3893617560	-1.1754474446	-1.7825692055
C	6.0	2.3563958819	-2.7300835540	-3.5116138083
C	6.0	3.9044330677	-4.1104302229	-5.4063911716
C	6.0	6.0029058999	-0.4211594821	-1.4655260740
C	6.0	2.0286380032	1.6547141153	1.5811187314
C	6.0	3.2071997164	0.9491054979	3.8269316198
C	6.0	3.6897261648	2.7914127257	5.6377805768
C	6.0	2.9747010420	5.2885459607	5.2001048883
C	6.0	1.7790051820	5.9600450216	2.9522151909
C	6.0	1.3047632930	4.1403672642	1.1156848558
C	6.0	-3.1277253945	-0.4816647332	0.1030392067
C	6.0	-5.1852951047	0.1735751241	-1.4278354864
C	6.0	-7.4943609060	0.7871824251	-0.3370345442
C	6.0	-7.7691686586	0.7379739568	2.2800093724
C	6.0	-5.7307437638	0.0745062319	3.8098787313
C	6.0	-3.4148938457	-0.5306067501	2.7340765453
H	1.0	-3.8709490105	-4.5338025743	-3.4534839429
H	1.0	-2.6716059790	-3.3918788712	-6.3546897565
H	1.0	-1.4116915561	-6.1750769574	-5.0031651904
H	1.0	4.8226963455	-2.8081538094	-6.7294734697
H	1.0	5.3697267054	-5.2627549786	-4.5075410503
H	1.0	2.6953579467	-5.3662703963	-6.5078237130
H	1.0	6.1514837267	1.6374439083	-1.4965950612
H	1.0	6.7658620360	-1.1417857531	0.3117953621
H	1.0	7.0737872390	-1.2049347310	-3.0385813440
H	1.0	3.7065730732	-1.0152855965	4.1565924527
H	1.0	4.6064058520	2.2723314152	7.3979300716
H	1.0	3.3449286256	6.7183618801	6.6243650129
H	1.0	1.2151071268	7.9046280068	2.6232743437
H	1.0	0.3685570657	4.6230751262	-0.6450372259
H	1.0	-4.9616138924	0.2381036021	-3.4676342121
H	1.0	-9.0783746978	1.3084104645	-1.5323751357
H	1.0	-9.5758526592	1.2123821417	3.1298532261
H	1.0	-5.9491696478	0.0163725872	5.8491747898

H 1.0 -1.8404307297 -1.0713197556 3.9329830500

6-311++G(2df,2p)/M06-2X//6-31G(d)/PBE0-D3

Charge: 1
E: -806.5631 H
G_{korr}: 750 kJ/mol
G: -2116881.2130 kJ/mol

DMSO

PCM/6-31G(d)/PBE0-D3

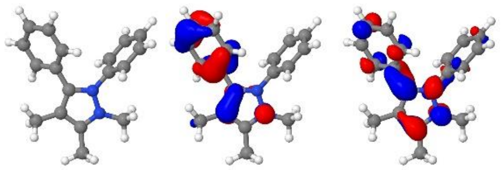

HOMO LUMO

Charge: 1
Number of imaginary frequencies: 0
E: -805.7893 H
H: -0.2661 a.u.
L: -0.0601 a.u.
HL-Gap: -5.6055 eV

ATOM	ATOMIC CHARGE	COORDINATES (BOHR) X	Y	Z
C	6.0	-0.7302279694	-1.1169585312	-1.1141012653
C	6.0	-0.2660734385	-2.7255501010	-3.1569726857
C	6.0	-2.1549548563	-4.2241482719	-4.6184755328
N	7.0	1.5207325330	-0.1998083021	-0.3083125968
N	7.0	3.3796410048	-1.1591164314	-1.7875788695
C	6.0	2.3536444406	-2.7092890077	-3.5153120023
C	6.0	3.9127478626	-4.1169875725	-5.3736289898
C	6.0	6.0034784869	-0.4689317586	-1.4271381775
C	6.0	2.0333207446	1.6692253222	1.5708008267
C	6.0	3.2024867395	0.9494683253	3.8161337247
C	6.0	3.6909922813	2.7872628871	5.6323759601
C	6.0	2.9968486322	5.2923669870	5.1965899977
C	6.0	1.8164822306	5.9770903513	2.9420125595
C	6.0	1.3337214562	4.1611504721	1.1018615092
C	6.0	-3.1350707599	-0.4668190448	0.0965366591
C	6.0	-5.2160503974	0.1343255124	-1.4259778856
C	6.0	-7.5322329073	0.7161910837	-0.3250744676
C	6.0	-7.7905981529	0.6934425606	2.2967466767
C	6.0	-5.7254336334	0.0876568361	3.8193122441
C	6.0	-3.4045778308	-0.4901118090	2.7316293500
H	1.0	-3.8437426234	-4.5760511813	-3.4824496650
H	1.0	-2.7432492759	-3.2334744685	-6.3370964062
H	1.0	-1.3710284294	-6.0487374276	-5.1877687562
H	1.0	4.7772654397	-2.8424069852	-6.7530837080
H	1.0	5.4184816394	-5.1794293948	-4.4384148687
H	1.0	2.7219785186	-5.4475607450	-6.4025168350
H	1.0	6.1833728551	1.5839892253	-1.3831736992
H	1.0	6.7310532808	-1.2793559253	0.3233774935
H	1.0	7.0597919273	-1.2078467990	-3.0287831140
H	1.0	3.6934564841	-1.0150815061	4.1375950359
H	1.0	4.6010144634	2.2535721039	7.3904505356
H	1.0	3.3735069538	6.7160847602	6.6243498951
H	1.0	1.2787247568	7.9270703942	2.6075820579
H	1.0	0.4304815011	4.6505366263	-0.6717258280
H	1.0	-5.0049887762	0.1751398173	-3.4658862155
H	1.0	-9.1316744232	1.1935321236	-1.5176201541
H	1.0	-9.5996707673	1.1423262148	3.1546294253
H	1.0	-5.9229137930	0.0577991633	5.8607002295
H	1.0	-1.8107336837	-0.9825971140	3.9245605406

PCM/6-311++G(2df,2p)/M06-2X//6-31G(d)/PBE0-D3

Charge: 1
E: -806.6251 H
G_{korr}: 753 kJ/mol
G: -2117040.9290 kJ/mol

1,3,4,5-Tetramethyl-2-methylene-2,3-dihydro-1H-imidazole **5**

vacuum

6-31G(d)/PBE0-D3

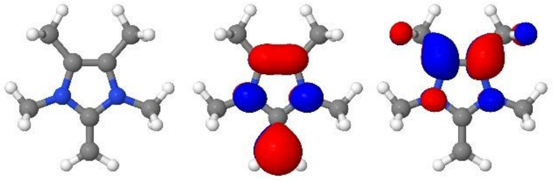

Charge: 0
Number of imaginary frequencies: 0
E: -422.2627 H
H: -0.1387 a.u.
L: 0.0678 a.u.
HL-Gap: -5.6192 eV

ATOM	ATOMIC CHARGE	COORDINATES (BOHR) X	Y	Z
C	6.0	-1.0911826669	-11.3277875145	1.6727987943
C	6.0	-2.4868701325	-7.2450795739	1.6063522443
C	6.0	-4.5521366972	-8.7510042130	1.6273527707
N	7.0	-0.3589478084	-8.8181575206	1.6210939978
N	7.0	-3.7046890162	-11.2577807205	1.6586844299
C	6.0	-7.2805497333	-8.1056570720	1.6019831975
H	1.0	-7.5300710606	-6.0559694095	1.5748127153
H	1.0	-8.2473298396	-8.8735264961	-0.0667640240
H	1.0	-8.2694112894	-8.8295903637	3.2773425708
C	6.0	-2.2661350032	-4.4506036022	1.5513593243
H	1.0	-1.2477275791	-3.7837098039	-0.1300207163
H	1.0	-4.1404806477	-3.5841396072	1.5247576497
H	1.0	-1.2640755998	-3.7137011201	3.2132714065
C	6.0	2.2458601316	-8.0940314769	1.6722280970
H	1.0	3.2548170353	-8.8658749950	0.0300050714
H	1.0	2.4030267637	-6.0380869311	1.6374476877
H	1.0	3.1767713464	-8.8069249885	3.3864969312
C	6.0	-5.1890613289	-13.5167101980	1.7346533099
H	1.0	-7.1951284432	-13.0384772074	1.7420610363
H	1.0	-4.7961570318	-14.7114460771	0.0826131570
H	1.0	-4.7597760244	-14.6226365081	3.4391711572
C	6.0	0.4200653307	-13.3983793275	1.7308662987
H	1.0	-0.3781493155	-15.2794334997	1.7623340182
H	1.0	2.4551907637	-13.2116365917	1.7412673513

6-311++G(2df,2p)/M06-2X//6-31G(d)/PBE0-D3

Charge: 0
E: -422.7096 H
G_{korr}: 461 kJ/mol
G: -1109362.7665 kJ/mol

DMSO

PCM/6-31G(d)/PBE0-D3

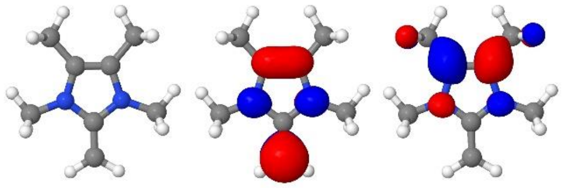

HOMO LUMO

Charge: 0
Number of imaginary frequencies: 0
E: -422.2709 H
H: -0.1525 a.u.
L: 0.0616 a.u.
HL-Gap: -5.8260 eV

ATOM	ATOMIC CHARGE	COORDINATES (BOHR) X	Y	Z
C	6.0	-1.0938509602	-11.3239589294	1.6906170220
C	6.0	-2.4864619516	-7.2420390046	1.6044927538
C	6.0	-4.5552925398	-8.7507075260	1.6252948590
N	7.0	-0.3573623281	-8.8212547817	1.6456774450
N	7.0	-3.7020830839	-11.2602298055	1.6811570530
C	6.0	-7.2871599953	-8.1091814112	1.5967240897
H	1.0	-7.5349484437	-6.0593482398	1.5536496724
H	1.0	-8.2506217425	-8.8949748876	-0.0647703630
H	1.0	-8.2708474812	-8.8277422115	3.2765507755
C	6.0	-2.2604374789	-4.4455183492	1.5524723730
H	1.0	-1.2240795464	-3.7826666751	-0.1184914972
H	1.0	-4.1354350790	-3.5803242502	1.5115579127
H	1.0	-1.2665020082	-3.7164960250	3.2221247734
C	6.0	2.2533585649	-8.0860379353	1.6448157299
H	1.0	3.2307268067	-8.8407076225	-0.0234779574
H	1.0	2.3983383532	-6.0299554396	1.6240627576
H	1.0	3.2192769561	-8.8067719207	3.3343934025
C	6.0	-5.1993471082	-13.5216801777	1.7128704368
H	1.0	-7.2017972867	-13.0325755927	1.7206277626
H	1.0	-4.8032321664	-14.6910483733	0.0444123434
H	1.0	-4.7832766585	-14.6529911789	3.4023366156
C	6.0	0.4248727940	-13.4044642456	1.7351106236
H	1.0	-0.3841038425	-15.2832601951	1.7656599361
H	1.0	2.4610848195	-13.2066817298	1.7314521138

311-6G++(2df,2p)/M06-2X/DMSO

Charge: 0
E: -422.7169 H
G_{korr}: 460 kJ/mol
G: -1109383.6090 kJ/mol

1,2,3,4,5-Pentamethyl-1H-imidazol-3-ium **5 cation**

vacuum

6-31G(d)/PBE0-D3

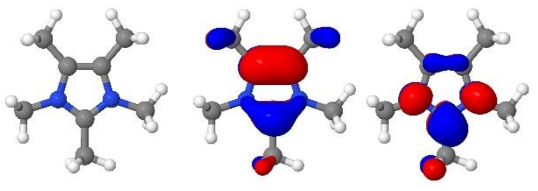

Charge: 1
Number of imaginary frequencies: 0
E: -422.7136 H
H: -0.3943 a.u.
L: -0.1536 a.u.
HL-Gap: -6.5498 eV

ATOM	ATOMIC CHARGE	COORDINATES (BOHR) X	Y	Z
C	6.0	-1.0597905365	-11.2465822035	1.7633544703
C	6.0	-2.4183864577	-7.2534208250	1.6539922399
C	6.0	-4.4891748021	-8.7956489927	1.6819356202
N	7.0	-0.3170261240	-8.8260225607	1.7066475687
N	7.0	-3.5896179233	-11.2561120923	1.7507840121
C	6.0	-7.2333405952	-8.2136190153	1.6528395070
H	1.0	-7.5304395572	-6.1745346060	1.5697614773
H	1.0	-8.1736796536	-9.0502725806	0.0083053463
H	1.0	-8.1812328889	-8.9149738592	3.3555677837
C	6.0	-2.2340928070	-4.4535383469	1.5835508089
H	1.0	-1.2301550159	-3.7901669980	-0.1014858518
H	1.0	-4.1237131078	-3.6274691375	1.5397715237
H	1.0	-1.2697542268	-3.7026405531	3.2550362436
C	6.0	2.3079508629	-7.9930955353	1.6990074060
H	1.0	3.2760613364	-8.6914116996	0.0149345056
H	1.0	2.3511518919	-5.9365708437	1.6788345796
H	1.0	3.2738654747	-8.6562571245	3.3981867770
C	6.0	-5.1924061441	-13.4869961444	1.7925375108
H	1.0	-6.3309264504	-13.5628818764	0.0718095928
H	1.0	-4.0255456151	-15.1789907767	1.9032584542
H	1.0	-6.4368418203	-13.4189943498	3.4380751161
C	6.0	0.6038582039	-13.5002336759	1.8259516482
H	1.0	0.2695751010	-14.7180827952	0.1860227501
H	1.0	2.5889455789	-12.9452418998	1.8017933894
H	1.0	0.2816145461	-14.6175682626	3.5385990973

6-311++G(2df,2p)/M06-2X//6-31G(d)/PBE0-D3

Charge: 1
E: -423.1450 H
G_{korr}: 501 kJ/mol
G: -1110466.2740 kJ/mol

DMSO

PCM/6-31G(d)/PBE0-D3

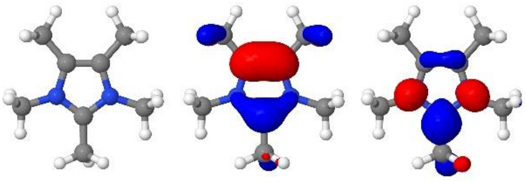

HOMO LUMO

Charge: 1
Number of imaginary frequencies: 0
E: -422.7836 H
H: -0.2612 a.u.
L: -0.0133 a.u.
HL-Gap: -6.7457 eV

ATOM	ATOMIC CHARGE	COORDINATES (BOHR) X	Y	Z
C	6.0	-1.0603291084	-11.2710749438	1.7767072751
C	6.0	-2.4037713158	-7.2734027891	1.6667176557
C	6.0	-4.4797318407	-8.8022857109	1.7734739537
N	7.0	-0.3156787493	-8.8536673642	1.6644632124
N	7.0	-3.5924109385	-11.2684935779	1.8289015107
C	6.0	-7.2172231211	-8.2019159414	1.8079425582
H	1.0	-7.4820625684	-6.1574231360	1.7958029576
H	1.0	-8.1832548959	-8.9843305874	0.1527219963
H	1.0	-8.1380224113	-8.9608847554	3.4979869932
C	6.0	-2.1643997075	-4.4838514436	1.5542373772
H	1.0	-1.2412080240	-3.8747416908	-0.1948969039
H	1.0	-4.0348922005	-3.6195511851	1.6247279412
H	1.0	-1.0573773562	-3.7554111551	3.1433458706
C	6.0	2.2793933217	-7.9685253162	1.5631908996
H	1.0	3.5404472495	-9.5705009548	1.3014846182
H	1.0	2.4950639850	-6.6775532445	-0.0286047844
H	1.0	2.7578549691	-6.9909397559	3.3149613487
C	6.0	-5.2415560309	-13.4606458033	1.9110743615
H	1.0	-6.2765646990	-13.4832148024	3.6946318938
H	1.0	-6.5696895669	-13.3715981288	0.3386313628
H	1.0	-4.1277004299	-15.1788018041	1.7419873370
C	6.0	0.6359476433	-13.4952769243	1.8078008287
H	1.0	1.3419795594	-13.9109505314	-0.0912284184
H	1.0	2.2523494511	-13.1492605118	3.0418808058
H	1.0	-0.3468441125	-15.1585231530	2.5192883842

PCM/6-311++G(2df,2p)/M06-2X//6-31G(d)/PBE0-D3

Charge: 1
E: -423.2135 H
G_{korr}: 496 kJ/mol
G: -1110651.0617 kJ/mol

1,3-Dimethyl-2-methylene-2,3-dihydro-1H-benzo[*d*]imidazole **6**

vacuum

6-31G(d)/PBE0-D3

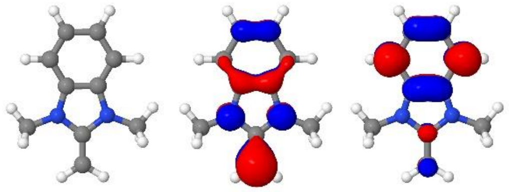

Charge: 0
Number of imaginary frequencies: 0
E: -497.2070 H
H: -0.1658 a.u.
L: 0.0162 a.u.
HL-Gap: -4.9525 eV

ATOM	ATOMIC CHARGE	COORDINATES (BOHR) X	Y	Z
C	6.0	-1.1056315129	-11.3129758412	1.3079266946
C	6.0	-2.4352125791	-7.2241451879	1.2324812687
C	6.0	-4.5883079449	-8.7905731883	1.2611918778
N	7.0	-0.3466494707	-8.7973081723	1.2612958127
N	7.0	-3.7329801060	-11.2613352953	1.3078038624
C	6.0	-6.9978618227	-7.7719654533	1.2421321001
H	1.0	-8.6658380360	-8.9673003756	1.2654891150
C	6.0	-2.6629396950	-4.6186739541	1.1854327574
H	1.0	-1.0122847114	-3.3995832846	1.1646552187
C	6.0	2.2616544626	-8.0704665921	1.2507624793
H	1.0	3.2287123586	-8.8302593267	-0.4189560615
H	1.0	2.4007704307	-6.0140420559	1.2169401611
H	1.0	3.2264900407	-8.7750925519	2.9457145330
C	6.0	-5.2264023171	-13.5196846269	1.3454169712
H	1.0	-7.2263448291	-13.0201147386	1.3383248290
H	1.0	-4.8272316882	-14.6911636466	-0.3184887720
H	1.0	-4.8256140826	-14.6362689924	3.0462252862
C	6.0	0.3952078732	-13.3743627981	1.3443341581
H	1.0	-0.4040971449	-15.2549237519	1.3799309291
H	1.0	2.4307528255	-13.1915884873	1.3406340744
C	6.0	-7.2269892257	-5.1347071363	1.1940517983
H	1.0	-9.0998665530	-4.2955156688	1.1780118029
C	6.0	-5.1022548696	-3.5890736821	1.1662879420
H	1.0	-5.3246000455	-1.5491370064	1.1288694750

6-311++G(2df,2p)/M06-2X//6-31G(d)/PBE0-D3

Charge: 0
E: -497.7356 H
G_{korr}: 439 kJ/mol
G: -1306365.8607 kJ/mol

DMSO

PCM/6-31G(d)/PBE0-D3

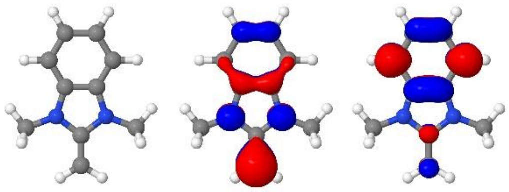

Charge: 0
Number of imaginary frequencies: 0
E: -497.2070 H
H: -0.1755 a.u.
L: 0.0050 a.u.
HL-Gap: -4.9117 eV

ATOM	ATOMIC CHARGE	COORDINATES (BOHR) X	Y	Z
C	6.0	-1.1065990526	-11.3114111479	1.3050864362
C	6.0	-2.4312498234	-7.2255738208	1.2299074618
C	6.0	-4.5881624360	-8.7949422351	1.2619383196
N	7.0	-0.3429172616	-8.7966788935	1.2570779440
N	7.0	-3.7347035363	-11.2648426270	1.3059821664
C	6.0	-7.0025710202	-7.7762419035	1.2486214196
H	1.0	-8.6682606649	-8.9753430500	1.2768501484
C	6.0	-2.6575142913	-4.6156428333	1.1766512001
H	1.0	-1.0039302322	-3.4001709895	1.1470486403
C	6.0	2.2719950439	-8.0584214778	1.2613279381
H	1.0	3.2438282779	-8.7976861175	-0.4125839050
H	1.0	2.4014526219	-6.0015679738	1.2500217066
H	1.0	3.2289788100	-8.7774584890	2.9523947148
C	6.0	-5.2414955597	-13.5254615197	1.3452828006
H	1.0	-7.2386658434	-13.0164656775	1.3352899289
H	1.0	-4.8425535876	-14.6970066798	-0.3164932212
H	1.0	-4.8452936905	-14.6381606082	3.0476520294
C	6.0	0.3971637398	-13.3764622838	1.3425351388
H	1.0	-0.4115936885	-15.2544267539	1.3851881472
H	1.0	2.4330582913	-13.1831433012	1.3347683645
C	6.0	-7.2290414683	-5.1353496432	1.1990236677
H	1.0	-9.1027540545	-4.2953909469	1.1892405555
C	6.0	-5.1010435551	-3.5872614348	1.1629620240
H	1.0	-5.3239121852	-1.5464951693	1.1224066117

PCM/6-311++G(2df,2p)/M06-2X//6-31G(d)/PBE0-D3

Charge: 0
E: -497.7428 H
G_{korr}: 439 kJ/mol
G: -1306385.0049 kJ/mol

1,2,3-Trimethyl-1H-benzo[d]imidazol-3-ium **6 cation**

vacuum

6-31G(d)/PBE0-D3

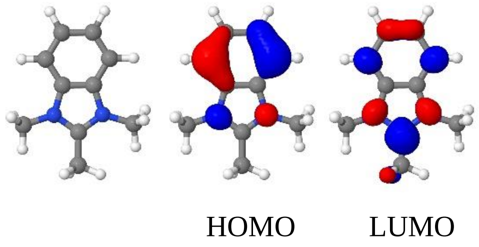

Charge: 1
Number of imaginary frequencies: 0
E: -497.6378 H
H: -0.3955 a.u.
L: -0.1803 a.u.
HL-Gap: -5.8559 eV

ATOM	ATOMIC CHARGE	COORDINATES (BOHR) X	Y	Z
C	6.0	-1.1858503869	-11.2531905757	1.3225588440
C	6.0	-2.4342129140	-7.2397864510	1.2390556259
C	6.0	-4.5817430364	-8.7732803046	1.2612523490
N	7.0	-0.3610227276	-8.8545989992	1.2653851800
N	7.0	-3.7221481959	-11.2515975366	1.3113017454
C	6.0	-7.0120782324	-7.7677589229	1.2322299352
H	1.0	-8.6823731396	-8.9565799593	1.2481603264
C	6.0	-2.6039896885	-4.6149493039	1.1968089087
H	1.0	-0.9432813620	-3.4130267963	1.1948190271
C	6.0	2.2383125655	-7.9633342386	1.2659388698
H	1.0	3.5213307800	-9.4857278409	0.7495825444
H	1.0	2.4186453502	-6.4613666860	-0.1373339564
H	1.0	2.7369678263	-7.2335220089	3.1329769434
C	6.0	-5.3735949747	-13.4448590312	1.3269411189
H	1.0	-6.5854461034	-13.3995641857	-0.3447370679
H	1.0	-4.2551246626	-15.1719704441	1.2963747988
H	1.0	-6.5409052586	-13.4189149813	3.0306690607
C	6.0	0.3929534300	-13.5665290478	1.3919533668
H	1.0	0.1648162435	-14.6639194650	-0.3490494229
H	1.0	2.3902163103	-13.1151585141	1.6069418389
C	6.0	-7.1856741434	-5.1521096242	1.1849584361
H	1.0	-9.0387452512	-4.2740238136	1.1620020432
C	6.0	-5.0242242984	-3.6066556940	1.1688050572
H	1.0	-5.2560918043	-1.5693778629	1.1367515227
H	1.0	-0.1491088399	-14.7654506703	2.9882995112

6-311++G(2df,2p)/M06-2X//6-31G(d)/PBE0-D3

Charge: 1
E: -498.1509 H
G_{korr}: 477 kJ/mol
G: -1307418.5830 kJ/mol

DMSO

PCM/6-31G(d)/PBE0-D3

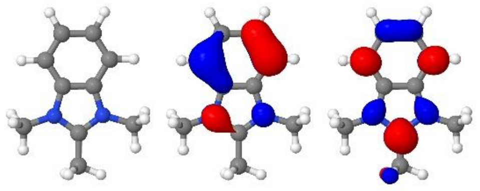

Charge: 1
Number of imaginary frequencies: 0
E: -497.7094 H
H: -0.2730 a.u.
L: -0.0519 a.u.
HL-Gap: -6.0164 eV

ATOM	ATOMIC CHARGE	COORDINATES (BOHR) X	Y	Z
C	6.0	-1.1912701214	-11.2455504130	1.2943093281
C	6.0	-2.4330923064	-7.2397392079	1.2246181183
C	6.0	-4.5833379652	-8.7747278348	1.2234445984
N	7.0	-0.3639064497	-8.8493436708	1.2633102608
N	7.0	-3.7255534824	-11.2476687960	1.2521892225
C	6.0	-7.0134029304	-7.7662150167	1.2036799529
H	1.0	-8.6745137686	-8.9630636096	1.2239302580
C	6.0	-2.5990820698	-4.6140743607	1.1973285834
H	1.0	-0.9292274688	-3.4291934033	1.1957298751
C	6.0	2.2318119076	-7.9630035365	1.3156216594
H	1.0	3.5166631565	-9.5384001772	1.0282453383
H	1.0	2.4960485323	-6.5948985135	-0.2023235276
H	1.0	2.6239848807	-7.0757979076	3.1345548647
C	6.0	-5.3508218852	-13.4555359838	1.3013391093
H	1.0	-6.7825577663	-13.2642597952	-0.1676357149
H	1.0	-4.2286042461	-15.1371975937	0.9365199219
H	1.0	-6.2542167978	-13.6151932750	3.1478963312
C	6.0	0.3765959606	-13.5529816012	1.4013453056
H	1.0	-0.0783650527	-14.8106982727	-0.1705969157
H	1.0	2.3775664836	-13.0917655944	1.3099052378
C	6.0	-7.1860010660	-5.1457620341	1.1816892099
H	1.0	-9.0388151711	-4.2685832921	1.1741076287
C	6.0	-5.0217752133	-3.6002344046	1.1765094706
H	1.0	-5.2499350765	-1.5630756263	1.1580279491
H	1.0	0.0247610814	-14.5663850305	3.1667293417

PCM/6-311++G(2df,2p)/M06-2X//6-31G(d)/PBE0-D3

Charge: 1
E: -498.2209 H
G_{korr}: 475 kJ/mol
G: -1307603.8807 kJ/mol

1,3-Dimethyl-2-methylene-1,2-dihydropyridine **7**

vacuum

6-31G(d)/PBE0-D3

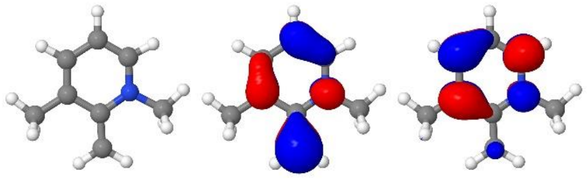

HOMO LUMO

Charge: 0
Number of imaginary frequencies: 0
E: -365.7596 H
H: -0.1674 a.u.
L: -0.0051 a.u.
HL-Gap: -4.4164 eV

ATOM	ATOMIC CHARGE	COORDINATES (BOHR) X	Y	Z
C	6.0	-3.7036024237	2.0773267968	0.2624300465
C	6.0	-3.6229829277	-0.4791457283	0.2960803996
N	7.0	-1.4064532353	-1.7936335520	0.1402271271
C	6.0	0.9600829169	-0.5930036171	-0.0677995939
C	6.0	0.8789059518	2.1703599037	-0.1035739992
C	6.0	-1.3631161461	3.4086747570	0.0556392063
C	6.0	3.1399689301	-1.9358637888	-0.2221807697
C	6.0	-1.4452039592	-4.5183219379	0.1866236830
C	6.0	3.3285843844	3.5621035109	-0.3197341015
H	1.0	-5.4954312872	3.0580209566	0.3898334921
H	1.0	-5.3086885515	-1.6362760575	0.4483110671
H	1.0	-1.3760702187	5.4637349109	0.0248442294
H	1.0	4.9297984633	-0.9640872466	-0.3809782355
H	1.0	3.2091895980	-3.9780927023	-0.1969963897
H	1.0	-3.3903236266	-5.1826268114	0.3542386108
H	1.0	-0.6153137237	-5.2845265133	-1.5525082778
H	1.0	-0.3451490282	-5.2300721652	1.7939548054
H	1.0	3.0226320555	5.6060161704	-0.3234625312
H	1.0	4.5960255864	3.0960535846	1.2556606494
H	1.0	4.3296441226	3.0503033151	-2.0637396660

6-311++G(2df,2p)/M06-2X//6-31G(d)/PBE0-D3

Charge: 0
E: -366.1518 H
G_{korr}: 372 kJ/mol
G: -960959.2257 kJ/mol

DMSO

PCM/6-31G(d)/PBE0-D3

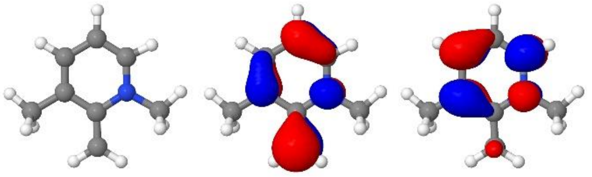

HOMO LUMO

Charge: 0
Number of imaginary frequencies: 0
E: -365.7664 H
H: -0.1770 a.u.
L: -0.0143 a.u.
HL-Gap: -4.4273 eV

ATOM	ATOMIC CHARGE	COORDINATES (BOHR) X	Y	Z
C	6.0	-3.7044376826	2.0793280168	0.2623128835
C	6.0	-3.6226333284	-0.4838794922	0.2962013421
N	7.0	-1.4044822510	-1.7944121191	0.1402063402
C	6.0	0.9592306505	-0.5915258512	-0.0677523508
C	6.0	0.8825984766	2.1734382676	-0.1038291122
C	6.0	-1.3651778373	3.4125940490	0.0555617275
C	6.0	3.1435934248	-1.9378801266	-0.2229007554
C	6.0	-1.4525247583	-4.5280483583	0.1874948467
C	6.0	3.3320558113	3.5688706201	-0.3199703173
H	1.0	-5.4991748347	3.0578338737	0.3901963196
H	1.0	-5.3064794617	-1.6438481901	0.4492294740
H	1.0	-1.3788046524	5.4687596927	0.0248177732
H	1.0	4.9336856300	-0.9620179965	-0.3792491361
H	1.0	3.2021125737	-3.9818135731	-0.1976388966
H	1.0	-3.4007152306	-5.1784996495	0.3544937239
H	1.0	-0.6257147763	-5.2940696302	-1.5515369586
H	1.0	-0.3567954103	-5.2405620350	1.7956442206
H	1.0	3.0193269245	5.6115341706	-0.3234341853
H	1.0	4.6030988313	3.1044647556	1.2534345520
H	1.0	4.3375790826	3.0583535484	-2.0621239502

PCM/6-311++G(2df,2p)/M06-2X//6-31G(d)/PBE0-D3

Charge: 0
E: -366.1578 H
G_{korr}: 372 kJ/mol
G: -962000.6955 kJ/mol

1,2,3-Trimethylpyridin-1-ium **7 cation**

vacuum

6-31G(d)/PBE0-D3

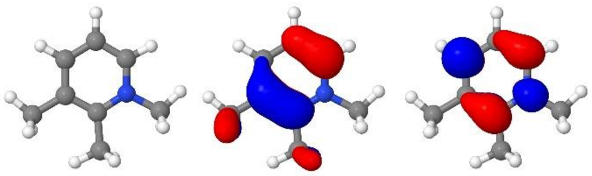

HOMO LUMO

Charge: 1
Number of imaginary frequencies: 0
E: -366.1848 H
H: -0.4417 a.u.
L: -0.2216 a.u.
HL-Gap: -5.9892 eV

ATOM	ATOMIC CHARGE	COORDINATES (BOHR) X	Y	Z
C	6.0	-12.5551551844	-0.5904676046	3.9983448972
C	6.0	-10.1741588478	-1.4624647177	3.4353047776
N	7.0	-9.1675829990	-1.1048000334	1.1152804544
C	6.0	-10.4425642083	0.1200694186	-0.7564876036
C	6.0	-12.8786630690	1.0457385330	-0.2675058509
C	6.0	-13.9063603867	0.6695469738	2.1306170736
C	6.0	-9.1621783823	0.4191299163	-3.2525871585
C	6.0	-6.6117492013	-2.0844567335	0.6315559198
C	6.0	-14.3584698033	2.4118725717	-2.2655435189
H	1.0	-13.3288411855	-0.8967071719	5.8702395669
H	1.0	-9.0086891572	-2.4584165262	4.7911624856
H	1.0	-15.7907480358	1.3751102378	2.5366058344
H	1.0	-7.4202023837	1.5278775875	-3.0825571606
H	1.0	-8.6578255972	-1.4136379740	-4.0747692012
H	1.0	-10.3863618636	1.3865015069	-4.5929774582
H	1.0	-6.6715514743	-3.4919606565	-0.8757085352
H	1.0	-5.3561716999	-0.5390179211	0.0931521596
H	1.0	-5.8977426410	-2.9652826470	2.3482643903
H	1.0	-14.6967251106	1.2090089805	-3.9158810285
H	1.0	-16.1967367966	2.9966747774	-1.5326321384
H	1.0	-13.3725694480	4.1131646566	-2.9119356785

6-311++G(2df,2p)/M06-2X//6-31G(d)/PBE0-D3

Charge: 1
E: -366.5614 H
G_{korr}: 406 kJ/mol
G: -962000.6955 kJ/mol

DMSO

PCM/6-31G(d)/PBE0-D3

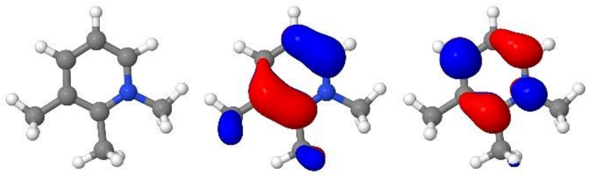

Charge: 1
Number of imaginary frequencies: 0
E: -366.2589 H
H: -0.3008 a.u.
L: -0.0792 a.u.
HL-Gap: -6.0300 eV

ATOM	ATOMIC CHARGE	COORDINATES (BOHR) X	Y	Z
C	6.0	-12.5573945098	-0.6018815504	3.9946467032
C	6.0	-10.1735673635	-1.4694264687	3.4300154342
N	7.0	-9.1690607648	-1.0955271473	1.1150971510
C	6.0	-10.4378569005	0.1316402116	-0.7487926389
C	6.0	-12.8722417796	1.0552268479	-0.2608199998
C	6.0	-13.9049166359	0.6722171568	2.1323915264
C	6.0	-9.1735242979	0.3944236369	-3.2550664792
C	6.0	-6.6149371693	-2.0555363649	0.6320340205
C	6.0	-14.3495087220	2.4032818768	-2.2682325992
H	1.0	-13.3324146576	-0.9258826535	5.8592602582
H	1.0	-9.0016801630	-2.4777087402	4.7644927808
H	1.0	-15.7884652467	1.3753502330	2.5320478150
H	1.0	-7.3251850644	1.3016433552	-3.0729668006
H	1.0	-8.8666063190	-1.4611683655	-4.1175620493
H	1.0	-10.3222585740	1.5186028117	-4.5341767401
H	1.0	-6.6501011930	-3.3494053868	-0.9689608503
H	1.0	-5.3478531255	-0.4815059962	0.2308319360
H	1.0	-5.9706539441	-3.0531190071	2.3064995532
H	1.0	-14.6930760494	1.1723690807	-3.8939734336
H	1.0	-16.1767359353	3.0049839032	-1.5257081819
H	1.0	-13.3519827716	4.0786752651	-2.9538687012

PCM/6-311++G(2df,2p)/M06-2X//6-31G(d)/PBE0-D3

Charge: 1
E: -366.6339 H
G_{korr}: 404 kJ/mol
G: -960974.9313 kJ/mol

2-(1,2-Dimethyl-1,2-dihydro-3*H*-indazol-3-ylidene)-*N*^{1},*N*^{3}-dimethylmalonamide **12b**

vacuum

6-31G(d)/PBE0-D3

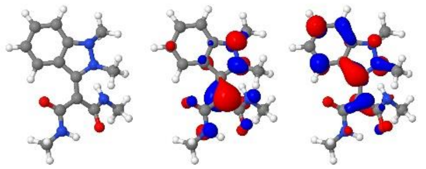

HOMO LUMO

Charge: 0
Number of imaginary frequencies: 0
E: - -912.7591 H
H: -0.1963 a.u.
L: -0.0488 a.u.
HL-Gap: -4.0137 eV

ATOM	ATOMIC CHARGE	COORDINATES (BOHR) X	Y	Z
C	6.0	-7.8132918739	2.3857546670	2.9441706267
C	6.0	-7.6743799962	0.2527376412	1.3689742971
N	7.0	-5.4095980369	3.2668186860	3.4864710022
N	7.0	-3.7661523584	1.7909822662	2.0304691478
C	6.0	-5.0163195740	-0.1103789030	0.7596547846
C	6.0	-3.7246520830	-1.8430385518	-0.7602991812
C	6.0	-4.7792591007	-4.4308805306	-1.0603763516
C	6.0	-1.3713099986	-1.1313601341	-2.0905454311
O	8.0	-6.7949468108	-5.0946921856	-0.0867270908
N	7.0	-1.0318792816	1.3839050232	-2.6180076769
N	7.0	-3.3977767065	-6.0802353826	-2.4471178537
O	8.0	0.2444681997	-2.6521096746	-2.8338238491
C	6.0	-9.9150736166	-0.8940974601	0.5445926132
C	6.0	-12.2027533873	0.1172934109	1.3148808867
C	6.0	-12.2990935149	2.2504899606	2.8859897388
C	6.0	-10.1101897287	3.4264533004	3.7138069448
C	6.0	-4.9189778916	5.9664398548	3.3493827101
C	6.0	-1.2046947358	1.5839703281	2.9673594560
C	6.0	1.0297854651	2.1763427766	-4.2161698482
C	6.0	-4.1868885419	-8.6755606769	-2.6368992690
H	1.0	-2.5396879777	2.5381913146	-2.4559655514
H	1.0	-1.6175356435	-5.5381428771	-2.9220608311
H	1.0	-9.8077560699	-2.5729036938	-0.6161735491
H	1.0	-13.9515683048	-0.7618147416	0.6986978890
H	1.0	-14.1202301409	2.9979144378	3.4709979246
H	1.0	-10.1849151689	5.0739392133	4.9332774491
H	1.0	-2.9674029197	6.3365370473	3.9121450400
H	1.0	-5.2476371696	6.7234357948	1.4427303077
H	1.0	-6.1584511470	6.9246160381	4.6910315674
H	1.0	-1.2143493466	2.0217839666	4.9871516513
H	1.0	-0.5684730822	-0.3627442681	2.6858923086
H	1.0	0.0919162787	2.8391774432	1.9587975051
H	1.0	2.7607122350	1.2335073900	-3.6057486255
H	1.0	0.7213519258	1.6931152399	-6.2110422250
H	1.0	1.2716949756	4.2219977636	-4.0477952505
H	1.0	-4.0475817114	-9.6727691533	-0.8215735509
H	1.0	-6.1590407415	-8.7783277631	-3.2477343418
H	1.0	-2.9950401643	-9.6356719397	-4.0238788766

DMSO

PCM/6-31G(d)/PBE0-D3

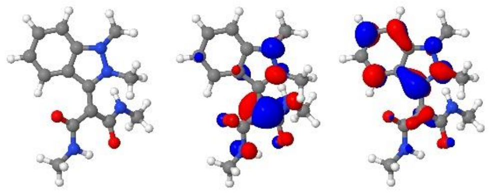

HOMO LUMO

Charge: 0
Number of imaginary frequencies: 0
E: -912.7851 H
H: -0.2017 a.u.
L: -0.0566 a.u.
HL-Gap: -3.9484 eV

ATOM	ATOMIC CHARGE	COORDINATES (BOHR) X	Y	Z
C	6.0	-7.8234056882	2.3805579201	2.8872426272
C	6.0	-7.6314586467	0.2736304532	1.2756671799
N	7.0	-5.4438360948	3.1703084830	3.5614307684
N	7.0	-3.7702587333	1.6750853629	2.2135609327
C	6.0	-4.9702593894	-0.1259729230	0.8249750579
C	6.0	-3.6724238323	-1.8791871229	-0.7301599393
C	6.0	-4.7042407530	-4.4573102402	-0.8529278867
C	6.0	-1.5065954919	-1.1084226383	-2.2695554075
O	8.0	-6.5167715662	-5.1573139299	0.4775829248
N	7.0	-1.0945161438	1.4143995337	-2.5932125805
N	7.0	-3.5868305772	-6.0899070009	-2.4833382343
O	8.0	-0.0568146160	-2.6250261198	-3.3637332903
C	6.0	-9.8323735321	-0.8324205788	0.2913447459
C	6.0	-12.1431268589	0.1988558802	0.9437330065
C	6.0	-12.2957940531	2.3198731450	2.5486981918
C	6.0	-10.1536496502	3.4523009744	3.5342754039
C	6.0	-4.8201716714	5.8332878623	3.8351878333
C	6.0	-1.1549628134	1.6343201910	3.0084043075
C	6.0	0.8889649639	2.3187468683	-4.2296398161
C	6.0	-4.2481780293	-8.7292137811	-2.4823650254
H	1.0	-2.4569085248	2.6456581496	-2.0842790993
H	1.0	-1.9281896110	-5.4849546456	-3.2525833791
H	1.0	-9.6909785542	-2.4830944597	-0.9106042178
H	1.0	-13.8709355807	-0.6280977209	0.2072840587
H	1.0	-14.1385812713	3.0916316255	3.0197672307
H	1.0	-10.2782600806	5.0932295376	4.7566239612
H	1.0	-2.9361298421	6.0238251681	4.6472844076
H	1.0	-4.9108199439	6.8234287630	2.0197222754
H	1.0	-6.1698424161	6.6622918163	5.1526122913
H	1.0	-1.0822990644	1.9930072171	5.0402227198
H	1.0	-0.4149422832	-0.2532251905	2.6126710904
H	1.0	-0.0168979310	3.0227341007	1.9908850547
H	1.0	2.6412135138	1.3041113375	-3.8247055224
H	1.0	0.4412132558	2.0483232803	-6.2381824717
H	1.0	1.1789434379	4.3297518370	-3.8778238341
H	1.0	-3.5122827713	-9.7337543948	-0.8182381842
H	1.0	-6.3026391401	-8.9405437434	-2.4866187989
H	1.0	-3.4756372023	-9.6163287031	-4.1792975121

3-(1,3-Bis(methylamino)-1,3-dioxopropan-2-yl)-1,2-dimethyl-1*H*-indazol-2-ium **12b cation**

vacuum

6-31G(d)/PBE0-D3

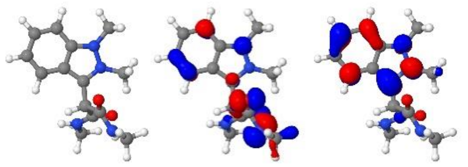

HOMO LUMO

Charge: 1
Number of imaginary frequencies: 0
E: -913.1765 H
H: -0.3717 a.u.
L: -0.1911 a.u.
HL-Gap: -4.9144 eV

ATOM	ATOMIC CHARGE	COORDINATES (BOHR) X	Y	Z
C	6.0	-3.2079404891	1.8365624604	3.5001242734
C	6.0	-3.0956246168	-0.1804499477	1.7512186491
N	7.0	-0.9204742574	2.9991824440	3.4850272514
N	7.0	0.5776061286	1.8198912965	1.7618956017
C	6.0	-0.6483725925	-0.1228473160	0.7146245008
C	6.0	0.5068283263	-1.8785427262	-1.1715772855
C	6.0	0.6851013095	-0.5914502622	-3.7678587811
C	6.0	3.0196821930	-2.9427306554	-0.1012118416
O	8.0	2.4248625491	0.8759674276	-4.2202459875
N	7.0	4.8510403476	-3.4418564581	-1.7860519708
N	7.0	-1.1639144460	-1.1636668919	-5.4087193142
O	8.0	3.1422214836	-3.3593415668	2.1650875678
C	6.0	-5.2093550977	-1.7612417565	1.3936295538
C	6.0	-7.3505017253	-1.2576996639	2.7726175096
C	6.0	-7.4340068330	0.7877550121	4.5011481069
C	6.0	-5.3976285116	2.3600865170	4.9010916338
C	6.0	-0.0851775154	5.1742250890	4.9124904618
C	6.0	3.2125778770	2.5377132139	1.4475831244
C	6.0	7.2497982200	-4.5076374264	-1.0008669862
C	6.0	-1.3025031806	-0.0837318749	-7.9238446317
H	1.0	-0.7543219775	-3.5206788245	-1.2712830153
H	1.0	4.7461133045	-2.6229417519	-3.5081121457
H	1.0	-2.4928624540	-2.4333020660	-4.9013448571
H	1.0	-5.1411019695	-3.3402572226	0.0850735804
H	1.0	-9.0113139868	-2.4399482328	2.5531484969
H	1.0	-9.1645991215	1.1210649061	5.5517111107
H	1.0	-5.5011458190	3.9129065996	6.2346656913
H	1.0	1.6735055519	4.7573628441	5.9101165676
H	1.0	0.1505223551	6.8268794029	3.6927176012
H	1.0	-1.5403176546	5.5816462622	6.3106912631
H	1.0	3.3818009618	4.5886575443	1.5444542651
H	1.0	4.3516877779	1.6217213869	2.9079729228
H	1.0	3.7991942197	1.9369031382	-0.4348278712
H	1.0	8.6566369595	-3.0295390045	-0.6471518295
H	1.0	6.9535175095	-5.5714304028	0.7389490555
H	1.0	7.9543089066	-5.7735857448	-2.4710739118
H	1.0	0.2328331559	1.2769692012	-8.1150131060
H	1.0	-1.0916910032	-1.5547041396	-9.3613970899

H	1.0	-3.1102359792	0.8810375628	-8.1945119944

DMSO

PCM/6-31G(d)/PBE0-D3

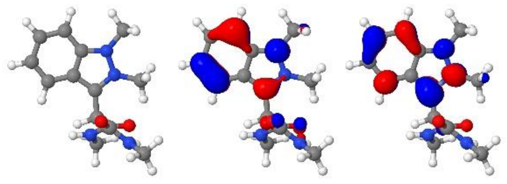

Charge: 1
Number of imaginary frequencies: 0
E: -913.2532 H
H: -0.2619 a.u.
L: -0.0795 a.u.
HL-Gap: -4.9634 eV

ATOM	ATOMIC CHARGE	COORDINATES (BOHR) X	Y	Z
C	6.0	-3.1593272845	1.8779776981	3.5126248117
C	6.0	-3.0530528666	-0.1718214582	1.8004856989
N	7.0	-0.8996910494	3.0746807822	3.4241837393
N	7.0	0.5874912860	1.8829193320	1.7134524725
C	6.0	-0.6337895760	-0.0855894757	0.7007822569
C	6.0	0.4387055892	-1.8568052066	-1.2232990895
C	6.0	0.7047695790	-0.5820828898	-3.8118723923
C	6.0	2.8307265880	-3.1640837251	-0.1672955642
O	8.0	2.4750782414	0.8752001988	-4.2647244713
N	7.0	4.9298891702	-3.0292555455	-1.5677923827
N	7.0	-1.1037021025	-1.1697499203	-5.4670476008
O	8.0	2.6563010872	-4.2863882916	1.8575988916
C	6.0	-5.1449966951	-1.8006047517	1.5268268998
C	6.0	-7.2605488720	-1.2981549208	2.9496451633
C	6.0	-7.3415009597	0.7882482306	4.6351731529
C	6.0	-5.3227329961	2.4019231637	4.9570105196
C	6.0	-0.0638708533	5.2764573827	4.8070437440
C	6.0	3.1838748269	2.6974895578	1.3578211335
C	6.0	7.2869143309	-4.1497440852	-0.7518426568
C	6.0	-1.2051822851	-0.1668231326	-8.0095210348
H	1.0	-0.9468926286	-3.3857939531	-1.3750006337
H	1.0	4.9334191786	-1.8401075866	-3.0669140078
H	1.0	-2.4261664601	-2.4407664842	-4.9553853551
H	1.0	-5.0648628587	-3.4015164745	0.2507968925
H	1.0	-8.9062111991	-2.5088911110	2.7946573854
H	1.0	-9.0536835362	1.1195871403	5.7127951451
H	1.0	-5.4053310353	3.9903551351	6.2461042036
H	1.0	1.6730104437	4.8587806658	5.8338226548
H	1.0	0.2113072856	6.8778982288	3.5374425849
H	1.0	-1.5459414795	5.7282776712	6.1575176223
H	1.0	3.2328716458	4.7502045616	1.2120665574
H	1.0	4.3311067706	2.0614814433	2.9498114592
H	1.0	3.8570897591	1.8991388513	-0.4122966666
H	1.0	8.3445014467	-2.8711988524	0.4845541245
H	1.0	6.8901568822	-5.8915707954	0.2783037459
H	1.0	8.4305236697	-4.5843697557	-2.4104949614
H	1.0	0.0658475068	1.4495276526	-8.1300666643
H	1.0	-0.6177817060	-1.5969281801	-9.3820291197
H	1.0	-3.1294941781	0.4372127056	-8.4385096523

N^1,N^3-Dimethyl-2-(1,2,4,5-tetramethyl-1,2-dihydro-3*H*-pyrazol-3-ylidene)malonamide **12a**

vacuum

6-31G(d)/PBE0-D3

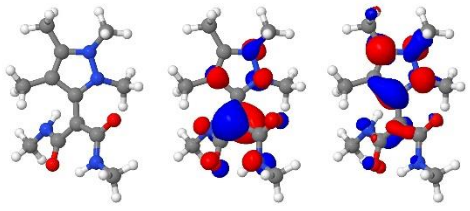

HOMO LUMO

Charge: 0
Number of imaginary frequencies: 0
E: -837.8330 H
H: -0.1805 a.u.
L: -0.0248 a.u.
HL-Gap: -4.2368 eV

ATOM	ATOMIC CHARGE	COORDINATES (BOHR) X	Y	Z
C	6.0	-10.1603940827	-4.3090423284	-0.4166713826
C	6.0	-10.2629514093	-1.8163764059	-1.1144584236
N	7.0	-8.0976786483	-4.7087893238	1.1069656595
N	7.0	-6.7935125087	-2.4604744380	1.2136860527
C	6.0	-8.0546703714	-0.6230011296	-0.0720344702
C	6.0	-7.1386672061	1.8735160548	-0.3334497337
C	6.0	-8.8792769327	4.0071907052	-0.3580105042
C	6.0	-4.4156682353	2.1667921008	-0.8960930109
O	8.0	-8.4332864493	6.1172815736	-1.2802478760
N	7.0	-3.6281418801	4.5432153001	-1.4659814980
N	7.0	-11.1774182273	3.6269078889	0.8209556104
O	8.0	-2.9264449957	0.3578801131	-0.8837719965
C	6.0	-6.6088031183	-7.0102036240	0.9862990875
C	6.0	-4.9566760380	-2.0849064883	3.1943684760
C	6.0	-1.0952644753	4.9899749021	-2.3377291672
C	6.0	-12.8948542424	5.7218261462	1.1236443823
C	6.0	-11.8113703198	-6.4550115370	-1.1759482220
C	6.0	-12.1212400509	-0.6208619596	-2.8613836149
H	1.0	-5.0200612317	5.8412549476	-1.7492249880
H	1.0	-11.2506734605	2.2419899725	2.1272231255
H	1.0	-5.5458775261	-7.1356776593	-0.7903080321
H	1.0	-5.2883399806	-7.0628457247	2.5700823327
H	1.0	-7.8821327070	-8.6230527468	1.1584172327
H	1.0	-5.5190604227	-3.1994083756	4.8451387329
H	1.0	-3.0540279653	-2.5591672746	2.5588743670
H	1.0	-4.9810005927	-0.0767134321	3.6707986679
H	1.0	0.1939236950	3.7218533986	-1.3420532587
H	1.0	-0.8741816365	4.6401582504	-4.3754227380
H	1.0	-0.5670350007	6.9516655779	-1.9501368902
H	1.0	-13.0662278356	6.7162113718	-0.6758265337
H	1.0	-14.7520770785	5.0057919098	1.6831696113
H	1.0	-12.2449093977	7.0980739992	2.5371425166
H	1.0	-13.5057611248	-5.7459900742	-2.1143692083
H	1.0	-10.8484849390	-7.7378955810	-2.4902187272
H	1.0	-12.4108651461	-7.5653125631	0.4655548180
H	1.0	-11.1628257621	0.8049685274	-4.0134967213

H	1.0	-12.9618677103	-2.0198205411	-4.1322244343
H	1.0	-13.6385616283	0.3588665501	-1.8528103256

6-311++G(2df,2p)/M06-2X//6-31G(d)/PBE0-D3

Charge: 0
E: -838.7091 H
G_{korr}: 734 kJ/mol
G: -2201297.1201 kJ/mol

DMSO

PCM/6-31G(d)/PBE0-D3

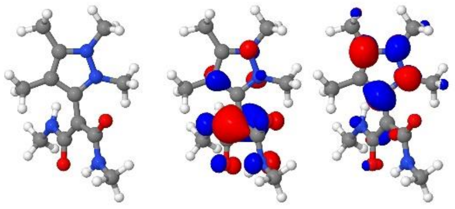

HOMO LUMO

Charge: 0
Number of imaginary frequencies: 0
E: -837.8627 H
H: -0.1895 a.u.
L: -0.0170 a.u.
HL-Gap: -4.6940 eV

ATOM	ATOMIC CHARGE	COORDINATES (BOHR) X	Y	Z
C	6.0	-10.0476983755	-4.3704414199	-0.6030664087
C	6.0	-10.1018617057	-1.8665694215	-1.3921139935
N	7.0	-8.0584517134	-4.6681753299	0.9679895311
N	7.0	-6.8817948441	-2.4106385806	1.2475178195
C	6.0	-8.0368691513	-0.6428848279	-0.2098465273
C	6.0	-7.1207318154	1.9144324048	-0.4285388626
C	6.0	-8.8301081486	4.0170418475	-0.1199314686
C	6.0	-4.4854652697	2.2130847217	-1.1591655643
O	8.0	-8.2825222092	6.2827516623	-0.5883208757
N	7.0	-3.5773649391	4.6195545664	-1.3293013868
N	7.0	-11.2077502213	3.5137718755	0.7771687664
O	8.0	-3.0753743116	0.3676764533	-1.6057645395
C	6.0	-7.1833082063	-6.9228737106	2.2329514124
C	6.0	-4.7781573904	-2.0737400967	2.9454858761
C	6.0	-1.1446486882	5.1174520471	-2.4347968394
C	6.0	-12.9498887364	5.5286942465	1.3383626236
C	6.0	-11.7212360528	-6.5177126499	-1.2960157508
C	6.0	-11.8596622709	-0.7112513396	-3.2661232667
H	1.0	-4.9368792671	5.9866221298	-1.2678493829
H	1.0	-11.5357084109	1.8114593385	1.5683479622
H	1.0	-5.1712808957	-7.2050872999	1.8667942989
H	1.0	-7.5201840135	-6.8115215987	4.2680201537
H	1.0	-8.2315770826	-8.5177307509	1.4660854329
H	1.0	-5.0626443202	-3.2033371162	4.6465190685
H	1.0	-2.9997077879	-2.5498055714	2.0121406941
H	1.0	-4.7463079463	-0.0713560585	3.4432132814
H	1.0	0.1986404514	3.6841722597	-1.7975207186
H	1.0	-1.1732194575	5.0812883582	-4.5152983761
H	1.0	-0.4801378345	6.9791251882	-1.8294249648
H	1.0	-13.0085779606	6.8566633763	-0.2419321872
H	1.0	-14.8327475971	4.7283971221	1.6098520171
H	1.0	-12.4274248161	6.5933621661	3.0449535005
H	1.0	-13.1626057577	-5.8714489917	-2.6207742360
H	1.0	-10.6613944936	-8.0523006548	-2.1939890392
H	1.0	-12.6815703033	-7.2902308002	0.3657734991
H	1.0	-10.8863304841	0.8080846858	-4.2803657344
H	1.0	-12.5049735071	-2.1091837999	-4.6456346767
H	1.0	-13.5251686121	0.1288377478	-2.3684466654

PCM/6-311++G(2df,2p)/M06-2X//6-31G(d)/PBE0-D3

Charge: 0
E: -838.7380 H
G_{korr}: 732 kJ/mol
G: -2201375.0477 kJ/mol

3-(1,3-Bis(methylamino)-1,3-dioxopropan-2-yl)-1,2,4,5-tetramethyl-1*H*-pyrazol-2-ium **12a cation**

vacuum

6-31G(d)/PBE0-D3

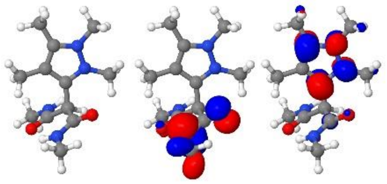

Charge: 1
Number of imaginary frequencies: 0
E: -838.2590 H
H: -0.3691 a.u.
L: -0.1674 a.u.
HL-Gap: -5.4885 eV

ATOM	ATOMIC CHARGE	COORDINATES (BOHR) X	Y	Z
C	6.0	-2.8007290755	-3.7907244673	0.6415128867
C	6.0	-1.5978976096	-1.9919489705	-0.8659254230
N	7.0	-2.5809293606	-3.0579434779	3.0724584642
N	7.0	-1.2638809581	-0.8695555869	3.1778862848
C	6.0	-0.6699740519	-0.1614411926	0.8020980334
C	6.0	0.8324243583	2.1810122899	0.2706087812
C	6.0	3.6681492719	1.5116070456	0.6824178984
C	6.0	0.0216713792	3.4154021820	-2.2408107834
O	8.0	4.2638192925	0.8443182945	2.8211362279
N	7.0	-2.2569471548	4.5440297721	-2.1432442235
N	7.0	5.1977710766	1.6751155986	-1.3075279624
O	8.0	1.3162509382	3.3450174428	-4.1597029419
C	6.0	-3.4224867653	-4.3523567409	5.3337727761
C	6.0	-0.6603421178	0.3265144389	5.5761282620
C	6.0	-3.3481184833	5.8658289462	-4.2822422325
C	6.0	7.8773932738	1.1454574909	-1.1446883724
C	6.0	-4.1297092088	-6.1562628441	-0.0856178216
C	6.0	-1.3444002985	-2.0386932359	-3.6766511497
H	1.0	0.4401380016	3.5656845419	1.7622489805
H	1.0	-3.1804676507	4.6173870506	-0.4761712994
H	1.0	4.4097855179	2.2823960965	-2.9557092945
H	1.0	-4.6874373083	-5.8688695155	4.7551915488
H	1.0	-1.8190352506	-5.1651241680	6.3547596763
H	1.0	-4.4624691925	-3.0581192224	6.5591864691
H	1.0	-2.2853913124	1.3800556511	6.2994020392
H	1.0	-0.0846389434	-1.1134946633	6.9342612002
H	1.0	0.9825555400	1.5272331909	5.2690232001
H	1.0	-2.2236331729	5.4168772619	-5.9493340539
H	1.0	-5.2976884557	5.2500579087	-4.5813083994
H	1.0	-3.3168113906	7.9120262845	-3.9824031676
H	1.0	8.9760139028	2.8424731256	-1.5799962340
H	1.0	8.3084889359	0.5302722686	0.7739940265
H	1.0	8.3943391938	-0.3476396872	-2.4780923546
H	1.0	-4.0701733872	-6.3949050181	-2.1319512202
H	1.0	-3.2462887013	-7.8209830593	0.7709043242
H	1.0	-6.1215939286	-6.1155203488	0.4794084002
H	1.0	0.5276795644	-1.4033276283	-4.2670223783
H	1.0	-1.6274680440	-3.9468498603	-4.4112179303

H	1.0	-2.7307128328	-0.7979387462	-4.5846040817

6-311++G(2df,2p)/M06-2X//6-31G(d)/PBE0-D3

Charge: 1
E: -839.1233 H
G_{korr}: 767 kJ/mol
G: -2202351.1762 kJ/mol

DMSO

PCM/6-31G(d)/PBE0-D3

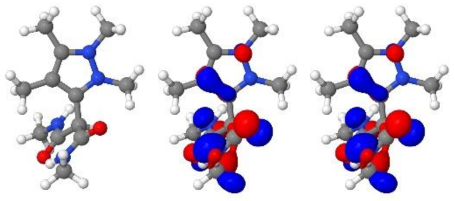

HOMO LUMO

Charge: 1
Number of imaginary frequencies: 0
E: -838.3365 H
H: -0.2762 a.u.
L: -0.0454 a.u.
HL-Gap: -6.2804 eV

ATOM	ATOMIC CHARGE	COORDINATES (BOHR) X	Y	Z
C	6.0	-2.7804031813	-3.7549443928	0.6822175875
C	6.0	-1.4358630430	-2.0433892053	-0.8136518189
N	7.0	-2.7076279384	-2.9216129660	3.0834169860
N	7.0	-1.3575225567	-0.7634965978	3.1878697079
C	6.0	-0.5924631554	-0.1700318876	0.8364475852
C	6.0	0.9725683375	2.1225611711	0.2956665496
C	6.0	3.7783902248	1.3510974879	0.5226264367
C	6.0	0.0616371970	3.5107785493	-2.0949749491
O	8.0	4.4643305728	0.3599399146	2.5167259155
N	7.0	-2.4222566168	3.9732720110	-2.1253768630
N	7.0	5.2792220520	1.7760723271	-1.4504687362
O	8.0	1.4924168768	4.2042645736	-3.8014146479
C	6.0	-3.8039071967	-4.0439364297	5.3238290372
C	6.0	-0.9122369412	0.5691685015	5.5407544786
C	6.0	-3.6295478363	5.3716731233	-4.1439898691
C	6.0	7.9357366782	1.1339282719	-1.3923804449
C	6.0	-4.1355163372	-6.0902093571	-0.0693623974
C	6.0	-1.0206297420	-2.1758117636	-3.5981972799
H	1.0	0.6872329206	3.4779861318	1.8392213050
H	1.0	-3.4733619720	3.4163413759	-0.6382852345
H	1.0	4.5048973235	2.7292142800	-2.9340870481
H	1.0	-4.9399349543	-5.6435782566	4.7177428462
H	1.0	-2.3187846628	-4.7063761435	6.5923171476
H	1.0	-5.0162402055	-2.6813399583	6.2797999101
H	1.0	-2.4038487946	1.9493526539	5.8902593255
H	1.0	-0.8285447505	-0.7911565191	7.0812459880
H	1.0	0.9304331140	1.4752959581	5.4007106549
H	1.0	-2.7931248175	4.8271277532	-5.9484572210
H	1.0	-5.6363916281	4.9168330524	-4.1594799542
H	1.0	-3.3962838228	7.4123033285	-3.8884667716
H	1.0	8.9574681306	2.2963252678	-0.0168922618
H	1.0	8.1847194336	-0.8530356011	-0.8832882267
H	1.0	8.7275923960	1.4575155249	-3.2662593270
H	1.0	-3.5325576429	-6.6507436999	-1.9582683818
H	1.0	-3.7557777620	-7.6535608835	1.2235107388
H	1.0	-6.1769969192	-5.7662460485	-0.1200127268
H	1.0	0.7747159018	-1.2983495626	-4.1214681132
H	1.0	-0.9733922580	-4.1376460585	-4.2392774193
H	1.0	-2.5283798565	-1.2083362380	-4.6314976355

PCM/6-311++G(2df,2p)/M06-2X//6-31G(d)/PBE0-D3

Charge: 1
E: -839.1982 H
G_{korr}: 763 kJ/mol
G: -2202552.4052 kJ/mol
